# Amazing grace: Vancouver's supervised injection facility granted six-month lease on life

**DOI:** 10.1186/1477-7517-5-3

**Published:** 2008-01-24

**Authors:** Dan Small

**Affiliations:** 1 Department of Medicine, University of British Columbia, Vancouver, Canada; 2 PHS Community Services Society, Vancouver, Canada

## Abstract

Addiction should be a matter, primarily, for the Chief of Medicine rather than the Chief of Police. While internationally renowned for its social kindness, Canada has not been without its share of disgraceful political mistakes in the not too distant past. Regrettably, there are many shameful events in Canada that have unfolded in the name of public policy including the banishment without medical treatment of Chinese Canadians living with leprosy to die on D'Arcy and Bentinck Islands in British Columbia while European Canadians stricken similarly enjoyed healthcare on the mainland as well as the eternally haunting treatment of people of aboriginal ancestry who were without full voting privileges in some parts of Canada until 1965 and abandoned to encampments, reserves, that paralleled South African apartheid. In due course, these public policies have come to be understood as horrific in retrospect. Many have all met with a remorseful fate where a future Prime Minister is held to public account for the sad excesses of an earlier generation. With respect to North America's only supervised injection facility (SIF), a medical program aimed at reducing fatal overdoses and infections (HIV, HCV) in injection drug users, Canada's Prime Minister Stephen Harper holds the ability to forestall a similarly heartrending fate in his political hands. The SIF currently has a temporary exemption from Canada's "Controlled Drugs and Substances Act" in order to operate until June of 2008. As such, the fate of the SIF is politically determined each time behind closed doors by the Prime Minister and his ministers. Sadly, the Prime Minister appears lost at present, content to ignore the scientific and medical evidence on the matter of population health. In light of the vast medical evidence accumulated on Vancouver's SIF, the fate of injection facilities needs to be taken out of the political realm entirely. I am hoping that the Prime Minister will be found, see the light of the scientific evidence, and lead the way towards to provision of a permanent medical exemption for injection facilities from Canada's Controlled Drugs and Substances Act (CDSA). In so doing, the Prime Minister would be on the brink of grace and would rescue a life saving health program from perpetual political interference.

## 

Amazing grace, how sweet the sound

That saved a wretch like me!

I once was lost, but now am found

*Was blind, but now I see*.

*It was grace that taught my heart to fear*,

And grace my fears relieved;

*How precious did that grace appear*,

The hour I first believed!

(John Newton, 1 January 1773)[1]

## Introduction

25 March of 2007 marked the 200^th ^anniversary of the abolition of the slave trade in Britain [2]. The words for Amazing Grace were written by John Newton inspired by an epiphany on 21 March 1748 during a terrible storm aboard the ship Greyhound as he returned from the Plantain islands off the coast of West Africa after working as a land-based slave trader [3]. Recalling the near death experience during the storm some twenty-five years earlier when he had pleaded for the mercy of God, Newton, now a Christian Minster, penned the hymn to accompany a sermon. The now famous hymn was sung for the first time on New Years day in 1773 the very morning that it was written by the angst ridden minister [4]. Sadly, slavery has still not entirely left the world as millions of individuals are still forced into compulsory or oppressive labour even today [5].

When addiction is treated as a crime rather than as a healthcare matter, psychosocially wounded people are forced into conflict with the law. The criminalization of addiction comes at terrible price with the years of life lost due to incarceration for non-violent drug offences estimated to be equivalent to 100,000's of deaths in North America [6]. The far reaching consequences of addiction throughout much of the world has resulted in many thousands deaths due to fatal overdoses and HIV infections that still haunt us 25 years after the discovery of AIDS. North America's only Supervised Injection Facility (SIF) is an important innovation for curbing fatalities due to overdose, HIV and HCV and yet this healthcare program still faces the threat of political closure in June of 2008 by Canada's Prime Minister Stephen Harper. The motivation for such a closure would ignore the accumulated science and medical evidence associated with the program and would be purely political. It would result in unnecessary suffering and death of the most vulnerable citizenry. Like the anguished slave trader John Newton turned believer, who was lost before he was found, so, too, has the Canadian Prime Minister Stephen Harper lost his way. But we are still hopeful that he, too, will be found in time.

After renouncing the slave trade, Newton was ordained as an Anglican Minister in 1764 following which he became a widely renowned preacher and writer whose sermons were not only delivered but also published [7,8]. One of those influenced by the reflective preacher was slave trade abolitionist William Wilberforce. John Newton was a spiritual mentor and friend to Wilberforce during his campaign, as a Member of Parliament, to abolish slavery [5]. Newton convinced Wilberforce, who considered entering the priesthood to pursue instead a religious centred approach to politics. Moral struggles pertaining to the treatment of people as less significant humans like those in the day of John Newton and his understudy William Wilberforce are never far from us. People of Aboriginal ancestry, for example, were not granted the vote in Alberta, Canada until 1965 [9]. Arguably, in the case of people with addictions who are sometimes blamed for their condition, this moral battleground where some people are considered to have reduced human value still exists in the present day [10,11]. This moral borderland is illustrated by the ongoing struggle to transform the political treatment of addiction as a primarily criminal justice issue for the Chief of Police to a healthcare matter for the Chief of Medicine. Before the British slave trade was finally outlawed in 1807, Wilberforce had to introduce laws to end human trafficking for nearly twenty years [12,13]. The Bills, starting in 1789, were defeated repeatedly with the backing of the wealthy slave trading industry who maintained that it was essential that the matter be the subject of further study. At the time, the British government heavily subsidized the slave trade. It was afforded royal status and provided with annual grant for the security and maintenance of the largest slave factory in Western Africa (operated by the Royal African company in Sierra Leone on Bence Island). In the 1740's, a factory was not a facility for the mass production of industry goods but was, instead, an appalling detention area where newly captured Africans were taken into custody as part of their descent into slavery [3]. Even after Wilberforce's Bill banning the moving of slaves in ships passed into law after twelve attempts, it took nearly 50 years of effort before slavery was completely prohibited in 1833 three days after which Wilberforce died [13].

## Background on North America's only Supervised Injection Facility

In Vancouver, there has been a struggle over a period of many years to develop "low barrier" (with minimal barriers for client or patient enrolment) evidenced based strategies, such as needle distribution and supported housing, that would provide an extremely hard to reach target population, those living with multiple barriers including active addictions that have been unsuccessful in conventional treatment, with a doorway into healthcare. North America's first SIF, referred to as *Insite *in the community, was opened by the PHS Community Services Society, Vancouver Coastal Health (Province of BC) and Health Canada (Federal Government of Canada) in the spirit of evidenced based low threshold healthcare on 21 September 2003. The program was purpose designed both architecturally and programmatically so that marginalized drug addicts who were going to inject drugs regardless could be supervised by healthcare workers, ensuring that they do not share syringes (thereby reducing HIV, HCV) and that emergency procedures could be enacted during overdose events (so that fatal overdoses could be reduced in the population of people that use the facility). The Federal Government of Canada set 1.5 million dollars aside, to engage an independent team of scientists and physicians to evaluate the initiative. Following a call for proposals, the Centre for Excellence in HIV and AIDS was selected to perform the independent review. The highest standard for independent review of the program was chosen: the research would be subject to peer review of the world's leading scientists by publishing the results in scientific and medical journals.

### Purpose of Insite

The main purpose of Insite is to engage a target group of people who have not been reached by conventional services, to help curb the loss of life from overdoses and to curtail the spread of infectious disease including HIV/AIDS and Hepatitis (by prohibiting syringe sharing in the facility). There is an average of 600 visits from people living with serious and persistent addictions to the site each day. Each HIV infection and death from overdose prevented by the program gives one more person the chance, one day, to be on the threshold of a successful life. To date, **1,000,000 **injections that have taken place in the SIF, injections that would have otherwise occurred in the public realm in unsupervised and dangerous circumstances (where overdoses could occur without emergency interventions and syringes could be shared). There have been hundreds of overdose events at the facility, many of which, had they occurred in unsupervised settings would have resulted in death. Exactly how many people would have died, exactly, if the program did not exist is unknown. The answer would require a forbidden medical experiment; the non-existence of the supervised injection facility. But, surely, even one death would be too many.

The project was initially granted an exemption (under section 56) of the Controlled Drugs and Substances Act (CDSA). This exemption covered the facility until 12 September 2006. Near the end of Insite's three-year exemption for scientific study, a conservative government came into power on 6 February 2006. At this point, Stephen Harper, the first conservative Prime Minister in 13 years in Canada, appeared to take a political interest in the fate of the SIF.

In fact, like all human rights issues, the fate of people with addictions and their families should not the subject of partisan politics. Addiction is a deeply human issue that touches every spectrum of society. According to the UNAIDS Joint Programme on HIV/AIDS, one-third of the HIV infections outside the sub-Saharan world are due to injection drug use [14]. In addressing the pandemic of drug addiction, we do not have luxury of playing political parlour games. Notwithstanding, PM Harper gave no indication about the fate of the project until Friday 1 September 2006 after receiving the results of an opinion poll commissioned through his office that indicated Canadians supported the supervised injection facility and needle exchange programs [15]. Following the opinion poll, he commanded his Health Minister to grant Insite a temporary extension to operate until 31 December of 2007. Neither the PM nor the Health Minister provided any long-term plan, beyond temporary reprieve, for the operational sustainability of Insite. They left the healthcare providers, the Province of British Columbia and people with addictions wondering, waiting and worrying.

On 2 October 2007, Health Minister Tony Clement was ordered a second time by the Prime Minister to provide a temporary exemption for Insite's operation under the *Controlled Drugs and Substances Act*. This federal permit was extended to 30 June 30 2008. This extension was explicitly granted under the condition that further research could be completed to examine the impact of Insite on crime, treatment and prevention. Notably, the conservative Prime Minister and Health Minister have erased harm minization and the Four Pillars approach to addiction under which the SIF was established. While no one tree grows to heaven when it comes to addiction, the SIF has emerged through the Four Pillars model as an important part of a comprehensive approach to drug addiction. The Four Pillar approach to drug addiction was adopted in Vancouver, British Columbia and includes enforcement, harm reduction, treatment and prevention [16] [see Additional file 1]. In missing harm reduction, the federal Health Minister's approach, in contrast, became a kind of three-legged dog.

## Peer-reviewed research and evidence base on Vancouver's SIF

In actuality, Canada's SIF has already been the focus of extensive medical research and scientific evaluation. The research results to date have generated thirty peer-reviewed publications in some of the world's leading medical journals. Independent evaluation of the project has determined that it demonstrated effectiveness in terms of its main goal: harm minimization as well as beneficence in other realms including treatment, prevention and enforcement. The findings of the research team have been further independently appraised by impartial scientists through the peer review process before being accepted for publication in leading medical and scientific journals.

The research on Insite has already examined the impact of program on crime and public disorder. The SIF has not increased public disorder [17], crime or drug dealing [18] and there has not been an increase in drug use (Kerr et al., 2006a) or syringe littering [17]. The research indicates that Insite has decreased public injection and disorder while lowering the amount of publicly discarded syringes [17]. However, it is important to remember that the SIF is not a "crime reduction" program. The primary objective of the SIF is not crime reduction any more than the primary objective of police initiatives is the reduction of hypertension. Crime reduction is the responsibility of the police who are an important part of the Four Pillars approach. A more suitable strategy for crime reduction in the addiction treatment/harm minimization realm would be pharmaceutically assisted therapy [19]. The SIF has also had a positive impact in the treatment realm. It has increased the seeking of treatment and detox by people with addictions [20,17].

Most importantly, the SIF has reached its health related goals. It has met its first goal by reaching with a hard to reach and marginalized target population: high risk injection drug users [21,22]. It has reached its second goal: the reduction of HIV and HCV risk behaviour (syringe sharing) thereby positively impacting HIV risk behaviour [23,24]. The SIF has also been shown to improve the safety of injection practices that are known to reduce HIV [25] outside the SIF. Insite has reached its third main goal: saving lives by intervening in hundreds of overdose events some of which would have been fatal [20,26]. The SIF has shown beneficence in a crucial area of prevention: prevention of HIV, HCV and fatal overdoses.

## Business case for North America's first SIF

The business case for the SIF is becoming increasingly clear. If the SIF is considered to be, in part, a multifaceted needle distribution program with additional healthcare interventions including overdose prevention, then the health economic assumptions related to cost savings associated with syringe distribution follow. In a study examining the economic returns of needle distribution programs in Australia from the year 1991 to 2000, investigators compared the prevalence of HIV and HCV infections amongst people with active addictions in countries with and without needle and syringe distribution programs (NSP) [27] [see Additional file 2]. With regard to HIV, they began with a baseline study carried out in 1991 and examined 778 calendar years of data in 103 cities (67 without NSPs, 23 cities that launched NSP between the baseline and present study and 13 cities where NSPs were present when both studies were completed). With respect to HCV, the researchers analysed 190 calendar years of HCV seroprevelance data in 101 cities (41 cities without NSP, 9 cities that launched NSP between the baseline and present study and 51 cities where NSPs were present when both studies were completed). The data was then applied to the Australian population of people living with active injection drug addiction. Their results revealed that by the year 2000, there were 25,000 cases of HIV were prevented in persons that would live for a approximately 24 years after infection with an estimated $14,000 in treatment costs per year after diagnosis resulting in an overall cost savings of $7,025,000,000.

Cost Benefit of NSPs with respect to the prevention of 21,000 HCV by the year 2000 were estimated to be 783,000,000 bringing the combined savings for HIV and HCV to $7,808,000,000 over the life of the individuals otherwise infected. In order to calculate the final financial return on the investment in NSP, the researchers subtracted the initial investment of the Australian government (the cost to operate the programs) from the financial return. The investigators also took into consideration the years of life gained by individuals otherwise infected by HIV and HCV had NSPs not existed (quality adjusted life years: QALYs). The net effect of the NSPs was estimated to be 588,000 life years (25,000 individuals at 23 years each) for HIV and 1,200 life years for HCV [27]. Based on the case for needle distribution alone, the SIF is resulting in a massive amount of financial savings for Canada in terms of prevented cases of HIV and HCV without even beginning to consider the savings associated with preventing fatal overdoses.

After several years of operation, it is clear that an end to the SIF would have deleterious effects for the vulnerable individuals who cling to it for healthcare and community that relies on it to recover otherwise discarded needles while reducing public injections. Without Insite, one million injections would otherwise have occurred in unsafe settings in the community. The closure of Insite would result in 20,000 injections once again taking place each month in the ugly and dirty places such as alleyways and in front of local businesses and residents. On a human level, the level of human suffering brought by an end to Insite would be enormous: otherwise preventable fatal overdoses and deadly infections would ensue. Quite simply, if the SIF closes, vulnerable Canadians will die unnecessary deaths.

## A possible end to the politicization of addiction?

What is next, then, in the political card game? While politicians play the polls at political solitaire all the time, with each party chasing the polls in a desperate struggle to occupy the popular middle ground, there is no cheating at addiction. Everyone that dies from a preventable HIV infection or drug overdose is someone's son or daughter. Recall that before the British slave trade was finally outlawed in 1807, Wilberforce's Bills were defeated time and time again by the powerful slave trading industry who maintained further study was required [12,13]. The logic of further study parallels that of Prime Minister Harper's hand puppet, Health Minister Tony Clement, who steadfastly refuses to provide Canada's SIF with a long-term permit on the grounds that it requires, staggeringly, further study. The need for further study is particularly ludicrous in light of the fact that there are now **thirty **peer-reviewed published studies, paid for by Canadian tax dollars, indicating the beneficence and non-malevolence of the project. The Health Minister notes that he wants to see the impact of this healthcare project on crime. Imagine denying the necessary funding or permission from a Police Board for an important police initiative fighting gang violence until it's impact on epidemiology could be further studied.

## Chief Medical Health Officer or Chief of Police?

In the name of liberty, the PHS Community Services Society, operators of Insite and two people living with addiction that rely on the healthcare project entered a writ in the Supreme Court of British Columbia and statement of claim to the Attorney General of Canada in August of 2007. The case, represented by lawyers working pro bono, presents two lines of reasoning. Firstly, in the Constitution of Canada, there is a clear division of powers between the Federal and Provincial Government. The plaintiffs make the case that regulating the Supervised Injection Facility is not, in actuality, under the authority of the Federal Government. Secondly, under the Canadian Charter of Rights and Freedoms, each citizen of Canada is guaranteed the right to security of the person and the right not to be deprived as part of their life and security of the person (Section 7). Supporters of Insite including lawyers church ministers, politicians (provincial, municipal and federal), physicians, chief medical health officers, nurses, business leaders, leading medical and scientific researchers, AIDS service and advocacy organizations, family members, the Vancouver Police Department, the Province of British Columbia and people with active addictions living on the threshold of life, believe that closure of the SIF would seriously jeopardize the life chances of people with addictions.

## Will the Prime Minister be found?

Shortly after the 200^th ^anniversary of the abolition of slavery, will the Prime Minister Harper similarly teach his heart to feel for an important issue for one of the most marginalized people of our age: people with active addictions and their families? No human being deserves to die of a preventable overdose or HIV infection on the mantle of deleterious political policy just because they are marginalized. Will the Prime Minister be found and will he overcome his blindness to the medical and scientific evidence? If he is able to see, will he then believe with all his heart that everyone is a full citizen including those with addictions? If so, then grace could very well appear and the course of history in Canada changed so that the fundamental right to life saving healthcare for people with addictions is recognized by the Parliament of Canada. How sweet the sound.

## Supplementary Material

Additional file 1 **A Framework for Action: A Four-Pillar Approach to Drug Problems in Vancouv er. **Donald MacPherson: (Department CoVP ed.: City of Vancouver; 2001. Click here for file

Additional file 2 **Return on Investment in Needle and Syringe Programs in Australia: Report. **The National Centre for HIV Epidemiology and Clinical Research, Publications Production Unit: Commonwealth of Australia; 2002. Click here for file
