# Supplementary material for: Amazing grace: Vancouver's supervised injection facility granted six-month lease on life
Source: Harm Reduct J. 2008 Jan 24;5:3. doi: 10.1186/1477-7517-5-3 (PMC2233622; doi:10.1186/1477-7517-5-3)
Supplement: Additional file 1 — A Framework for Action: A Four-Pillar Approach to Drug Problems in Vancouv er. Donald MacPherson: (Department CoVP ed.: City of Vancouver; 2001. [file 1477-7517-5-3-S1.pdf]

A FRAMEWORK *FOR ACTION*

A Four-Pillar Approach to Drug Problems in Vancouver

PREVENTION

TREATMENT

ENFORCEMENT

HARM REDUCTION

DRAFT
DISCUSSION PAPER

Donald MacPherson,
Drug Policy Coordinator, City of Vancouver
November 21, 2000

Contents

Mayor's Message 1
Executive Summary 2

1. Regional, National and International Context 5
2. Background 11
3. Drug Use In Vancouver- Trends 12
4. Issues, Dilemmas and Attitudes 16
5. The Costs of Drug Addiction 18
6. Working Towards Solutions - A Community Effort 20
7. Outcomes in the U.S., U.K., and Europe 22
8. A Framework for Action – A Four-Pillar Approach 29
9. Provincial and Federal Responsibility 31
10. Pillar One - **Prevention** 32
11. Pillar Two - **Treatment** 35
12. Pillar Three - **Enforcement** 45
13. Pillar Four – **Harm Reduction** 53
14. Coordination, Monitoring and Evaluation Process 58
15. Conclusion - A Call to Action 59

Appendix A – Summary of Goals and Actions 60
Goals and Actions Flow Chart . . . . . *Insert*
Appendix B - Vancouver Agreement Initiatives 68
Appendix C – A List of Some Reports and Recommendations (1994-2000): Summary 69
Appendix D- *Placeholder forActions that May Require Legislative and/or Regulatory Changes in Order to be Implimented.* 72
Acknowledgements 73
References 74
Feedback Form - What Do You Think?

Writing and research:
Donald MacPherson, Drug Policy Coordinator, City of Vancouver
Mari-Louise Rowley, Pro-Textual Communications

Message from the Mayor

Vancouver is a city admired the world over for its beauty, climate, multicultural vibrancy, and progressive attitudes. However, more and more communities, individuals, and visitors are affected by the harsh realities of our major illegal drug problem. Substance misuse is affecting the health and well being of communities and individuals throughout the Lower Mainland.

Over the past several years, there have been numerous forums, meetings and reports to address the issues surrounding drugs. While some important initiatives have been undertaken to provide treatment and increase enforcement, it is clear that little progress has been made to reduce the negative impact of substance misuse on our neighbourhoods and our citizens.

Since 1993, Vancouver has averaged 147 illicit drug overdose deaths per year (CCENDU, 2000). Many of these occur in the Downtown Eastside, as well as in other neighbourhoods throughout the city. Hundreds of individuals have contracted HIV and hepatitis C from injection drug use and the fear of drug-related crimes in the city has increased. These trends must stop. We cannot ignore this issue. We cannot incarcerate our way out of it and we cannot liberalize our way out of it. Rather, all levels of government must play their part in managing it. What we need is a balance of public health and public order.

In September 2000, the federal and provincial governments and the City under the *Vancouver Agreement,* announced the first phase of a program to address the urgent and complex social, economic, and health and safety issues of the Downtown Eastside. This was an important first step, but there is a clear acknowledged need to address these issues in all Vancouver neighbourhoods.

The federal and provincial governments must do much more to fulfil their responsibilities with respect to drug misuse and the illegal drug trade. The four-pillar, approach outlined in this *Framework for Action* has proved successful in cities in the U.S., UK, and Europe. It is based on the four pillars of Prevention, Treatment, Enforcement, and Harm Reduction. All pillars are equally important and they must be integrated and jointly implemented to be effective. What the framework proposes is a no-nonsense, practical, city-wide approach. The key to making it work is cooperation, coordinated efforts, local participation, and a commitment to creating a safer and healthier community for everyone.

Mayor Philip Owen
City of Vancouver

2. Executive Summary

A Framework for Action is an urgent appeal to all levels of government, the many committed non-government agencies, our law enforcement agencies, our criminal justice system, and health care professionals to rally together to develop and implement a coordinated, comprehensive framework for action that will address the problem of substance misuse in the city of Vancouver - one that balances public order and public health and is based on four pillars; prevention, treatment, enforcement and harm reduction. To do this we must secure commitment for action and financial support from all levels of government, we must secure the support and cooperation of stakeholders, and we must foster widespread support from within the community.

The **purpose** of this *Framework for Action* is to:

1. Provide the City of Vancouver and its citizens with a framework for action that compels the provincial and federal governments to take responsibility for issues within their jurisdiction.

2. Show which levels of government are responsible for actions to achieve the goals in the framework.

3. Clarify Vancouver's drug problems and establish appropriate, achievable goals and actions.

Goals are presented along two themes: those that are achievable under current legislation and policy, and those that could be achievable with legislative and policy changes.

The four **goals** of *A Framework for Action* are:

1. **Provincial and Federal Responsibility:** To persuade other levels of government to take action and responsibility for elements of the framework within their jurisdiction by encouraging a regional approach to the development of services, and by demonstrating the city-wide, regional, national and international implications of the drug problems in Vancouver. This is the overarching goal and the key element to achieving the following three goals:

2. **Public Order:** To work towards the restoration of public order across Vancouver by reducing the open drug scene (particularly at Main and Hastings), by reducing the negative impact of illicit drugs on our community, by reducing the impact of organized crime on Vancouver communities and individuals, by providing neighbourhoods, organizations and individuals with a place to go with their concerns related to safety, criminal activity, drug misuse, and related problems, and by implementing crime prevention techniques to increase public safety.

3. **Public Health:** To work towards addressing the drug-related health crisis in Vancouver by reducing harm to communities and individuals, by increasing public awareness of addiction as a health issue, by reducing the HIV/ AIDS/hepatitis C crisis, by reducing overdose deaths, by reducing the number of those who misuse drugs, and by providing a range of services to groups at risk such as youth, women, Aboriginal persons, and the mentally ill.

4. **Coordinate, Monitor and Evaluate:** To advocate for the establishment of a single, accountable agent to coordinate implementation of the actions in this framework, and to monitor and evaluate implementation through senior representatives of the Vancouver/Richmond Health Board, the Vancouver Police Department, the City of Vancouver, the BC Centre for Disease Control, the Ministry of Children and Families, the Office of the Attorney General, and community representatives.

This draft discussion paper includes four major goals and 31 actions to achieve those goals, and the estimated cost of these actions is $20 to $30 million per year. This cost is considerably less than costs associated with addiction. For example: in 1997, the estimated direct costs arising from law enforcement and health care related to injection drug use and HIV/AIDS in British Columbia was $96 million annually (Pay Now or Pay Later, Millar 1998), and a recent Ontario study estimated annual direct costs to government as $33,761 per untreated injection drug user (Millar, 1998). A summary of the goals and associated actions including responsible agencies is in Appendix A.

This paper creates a framework for action to appropriately and effectively deal with city-wide substance misuse and associated crime. This *Framework for Action* discussion paper attempts to clarify that the four-pillar approach deals with people who have an addiction and need treatment, while clearly stating public disorder, including the open drug scene, must be stopped. In short, addiction needs treatment and criminal behaviour needs enforcement.

A Framework for Action includes the four pillars of *prevention, treatment, enforcement* and *harm reduction.* Each requires the interaction and support of the other three to help this city-wide framework improve public order and public health.

The following briefly describes the four pillars:

Prevention involves education about the dangers of drug use and builds awareness about why people misuse alcohol and drugs and what can be done to avoid addiction. *A Framework for Action* supports coordinated, evidence-based programs targeted to specific populations and age groups—programs that focus on the causes and nature of addiction as well as on prevention.

Treatment consists of a continuum of interventions and support programs that enable individuals with addiction problems to make healthier decisions

about their lives. These include detoxification, outpatient counselling and residential treatment, as well as housing, ongoing medical care, employment services, social programs, and life skills.

Enforcement strategies are key to any drug strategy. In order to increase public order and to close the open drug scene in the Downtown Eastside, more effective enforcement strategies will include a redeployment of officers in the Downtown Eastside, increased efforts to target organized crime, drug houses and drug dealers, and improved coordination with health services and other agencies to link drug and alcohol users to available programs throughout Vancouver and the region.

In order for *A Framework for Action* to increase public order, it requires the collaboration of various enforcement agencies such as the Vancouver Police Department, RCMP, the newly created Organized Crime Agency, probation services, and the courts with the other programs and agencies involved in each pillar.

Harm Reduction is a pragmatic approach that focuses on decreasing the negative consequences of drug use for communities and individuals. It recognizes that abstinence-based approaches are limited in dealing with a street-entrenched open drug scene and that the protection of communities and individuals is the primary goal of programs to tackle substance misuse. *A Framework for Action* attempts to demonstrate the need for harm reduction by outlining, and drawing upon, other successful programs around the world that have significantly reduced both the negative health and societal impacts and the costs of drug addiction.

1. Regional, National and International Context

While Vancouver has a significant drug problem, in reality the problem is regional, national and even international. The drug trade knows no boundaries. It is also big business. The movement toward a global economy, coupled with advanced communication technologies and increasingly sophisticated forms of transport and marketing have contributed to an escalating substance misuse problem worldwide.

Highly organized criminal factions have controlled the drug trade since the prohibition of heroin and cocaine in the early 1900s. The international "war on drugs" has, unfortunately, only succeeded in increasing drug production, trafficking, corruption, and fatalities. (Dan Gardner, How America Dictates the Global War on Drugs, Vancouver Sun, September 5-18, 2000).

Globalization of world economies has been very lucrative for international drug traffickers and this has exacerbated an already volatile situation. The increased movement of goods around the world, more "open" borders, and the ease with which funds can be transferred electronically through a myriad of bank accounts makes it easier than ever for drug criminals to do business. The City of Vancouver supports international efforts by the United Nations and its member countries in the battle against drug cartels and organized crime. However, there is still much to do at the local level.

Since the late 1980s, drugs have become more easily available, more potent and more deadly. The introduction of cocaine, crack-cocaine (a pure form of free-base cocaine which is sold in crystals or rocks and can be injected or smoked) and cheap heroin, combined with sophisticated drug trafficking strategies, has fuelled a marked increase in injection drug use worldwide. Countries such as Russia, Ukraine, Thailand, Vietnam, and India, where injection drug use was not common ten years ago, are now grappling with serious drug problems.

This rise in injection drug use has exacerbated outbreaks of HIV and hepatitis C around the world. Closer to home in the Pacific Northwest, Seattle, Washington and Portland, Oregon show an alarming rise in mortality rates from drug overdose. In 1999 there were 111 heroin overdose deaths in King County, Washington which includes Seattle (Drug Abuse Trends in Seattle-King County Area, 2000) and in 1998, there were 176 drug related deaths in Portland, Oregon, up 14 percent from 1997 (Drug Abuse Warning Network Annual Medical Examiner Data, 1998).

Vancouver is not alone in dealing with drug misuse and its related harm to society and individuals. However, many factors make our situation unique and contribute to escalating substance misuse.

A bustling seaport city, Vancouver is one of North America's main points of entry for drugs. Even though enforcement has increased to stem the tide of trafficking, and the Port of Vancouver has been the point-of-seizure for large

The planet has been reduced
to the size of a computer screen,
and the artificial borders which
we once called nations have,
for all intents and purposes,
begun to evaporate.
Borders have already become non-entities
for transnational organized crime.

Jeffrey Robinson, The Merger, 1999

shipments, most drugs find their way to market—whether destined for other North American cities, or to supply the estimated 12,000 injection drug users in the Lower Mainland (BC Centre for Excellence in HIV/AIDS, 1999).

Since the mid 1980s, a well-entrenched illicit drug market has developed in Vancouver's Downtown Eastside, fuelled by several social, economic and environmental factors:
· poverty;
· substandard housing;
· high unemployment;
· increased availability and low cost of heroin and cocaine;
· flight of legitimate business from the area;
· de-institutionalization of the mentally ill without adequate support structures in the Lower Mainland;
· displacement as a result of enforcement initiatives in the 1970s and 1980s that had the effect of pushing street level drug dealers into the Downtown Eastside from other areas of the city, thereby increasing the concentration of these factors in this community.

In addition, the response to the escalating problem by the alcohol and drug treatment system has been woefully inadequate. Today, illicit drugs are sold in virtually all Vancouver neighbourhoods. While couriers, cell phones, drug houses, and corrupt businesses are a part of the trade in other parts of Vancouver, in the Downtown Eastside the drug scene is open and public. Drug users buy and consume in full view of passers by. Addiction knows no borders, so surrounding municipalities struggle with many of the same issues. Although estimates vary, approximately forty percent of individuals who misuse drugs in the Downtown Eastside live in areas *outside* of the Downtown Eastside (Vancouver Injection Drug User Survey, 2000). Clearly, the problems are city-wide and beyond.

1.1 Lower Mainland Municipalities

In recognition of the regional and national nature of crime and drugs, the Lower Mainland Municipal Association (LMMA), recently received federal funding to develop a crime and drug prevention strategy. The first report[1] on behalf of Lower Mainland municipalities supports a four-pillar approach that balances public health and public order. The LMMA report recognizes that the crime and drug issues facing Vancouver extend into all municipalities to one degree or another. Further, the report recommends that prevention, treatment, enforcement and harm reduction actions be implemented across the region.

Consistent with the approach noted in the LMMA report, Lower Mainland municipalities must ensure that, within their areas of jurisdiction, they facilitate the implementation of prevention, treatment, enforcement and harm reduction actions.

This will ensure that a comprehensive range of services is available to drug addicts where they need it, not just in Vancouver. In addition, Lower

1. Dandurand and Chin. Towards a Lower Mainland Crime and Drug Misuse Prevention Strategy: Needs Assessment and Identification of Issues. Lower Mainland Municipal Association (LMMA), August 2000.

Mainland municipalities must improve their enforcement efforts to anticipate and respond to stronger enforcement efforts in Vancouver.

Various provincial ministries responsible should encourage municipalities throughout British Columbia, through policy or enabling legislation, to support the development of a full range of drug and alcohol services in order to provide adequate treatment and enforcement in their own communities.

1.2 National Strategy

Indeed, the four-pillar approach must be implemented nation wide. The national and international scale of the drug trade demands that Vancouver and the Lower Mainland be supported by national health funding for prevention and treatment, more effective legislation, more effective efforts against organized crime and more appropriate sentencing. In short, a strong, comprehensive national drug strategy.

1.3 Who is responsible for what?

This question is central to the successful implementation of *A Framework for Action*. Simply put, the City of Vancouver is responsible for by-law enforcement, zoning, licensing of businesses including liquor licensing, street cleaning and a range of community services related to where people work and live in the city. The provincial government is responsible for funding health and education services, which encompass many of the recommendations in this draft discussion paper. The provincial government has responsibility for provincial courts and provincial offences within the criminal justice system while the federal government is responsible for federal offences under the Criminal Code and the Controlled Drugs and Substances Act within the criminal justice system. The federal government is also responsible for immigration and health promotion and research, which encompass many of the recommendations in this draft framework for action.

However, multiple jurisdictions must be involved in dealing with Vancouver's crime prevention and substance misuse problems. The City of Vancouver, the provincial and federal governments, police and health authorities have specific responsibilities for health and enforcement issues, but many challenges exist in clarifying roles, responsibilities and funding.

The *Vancouver Agreement* brings the City of Vancouver, the Vancouver/ Richmond Health Board and the federal and provincial governments together in an attempt to coordinate a comprehensive approach to substance misuse in Vancouver. It should be noted that the *Vancouver Agreement* is a five-year agreement intended to deal with social and economic development, housing, health, justice and many other issues. The agreement has the DTES as its first focus, but it is intended to focus on city-wide initiatives throughout its five year term. (Refer to Appendix B for more detail about the *Vancouver Agreement*).

1.4 The City of Vancouver's Responsibility

The City has responsibility for a range of services that contribute to and facilitate community and individual well-being.

The City of Vancouver operates community centres, supports neighbourhood houses and facilities and programs in the downtown core, such as the Carnegie Centre, Gathering Place, and the Evelyne Saller Centre, which offer services to at-risk populations and contribute to the overall social development of the city. Supporting and creating positive conditions and opportunities for meaningful activities in the community creates a sound basis for substance misuse **prevention**.

The City's affordable housing initiatives, which serve individuals with special needs, such as the mentally ill, individuals with a dual diagnosis of mental health and substance misuse, individuals in recovery from substance misuse, youth-at-risk and other special groups in the community can support efforts to link housing with **treatment** programs.

Community safety and public order are among the primary concerns of municipal government. In addition to funding the Vancouver Police Department and supporting neighbourhood community policing efforts, the City of Vancouver provides a range of bylaw **enforcement** activities through the Permits and Licenses department and the Fire Department.

Aggressive enforcement of building and zoning bylaws in the past two years has reduced the impact of drug trafficking on many communities in Vancouver. The creation of the Neighbourhood Integrated Service Teams (NIST) throughout the city is a model of collaboration and cooperation in responding to community problems, including those associated with the illegal drug trade.

Police and other community services provided by the City can also play an important role in supporting **harm reduction** programs, by assisting drug users in accessing services and by working with community residents and organizations to address neighbourhood problems created by the sale and use of drugs by assisting citizens with drug and alcohol problems in accessing services as well as by working with community residents and organizations to address neighbourhood problems created by the sale and use of drugs.

Since the issue of substance misuse lives at the city level, municipalities have a significant role to play in recommending an appropriate, balanced response.

The City must continue its leadership role in addressing issues of substance misuse through advocacy for services and resources for residents that are provided by other levels of government.

1.5 The Provincial Government's Responsibility

Consistent with the provincial government's mandate and responsibility for health, education, and enforcement services to British Columbians, much of the responsibility for actions and funding under prevention, treatment, enforcement and harm reduction in this framework rests with provincial government ministries and the Vancouver/Richmond Health Board.

The following outlines general areas of responsibility. More detail follows in Sections 9 through 14.

**Prevention**	
Education	Ministry of Education
Community led processes to enable response to substance abuse	Ministry for Children and Families<br>Vancouver/Richmond Health Board
**Treatment**	
Drug Treatment	Ministry of Health,<br>Ministry for Children Families,<br>Vancouver/Richmond Health Board
Drug Treatment Research, clinical trials and evaluation	Ministry of Health
Employment and Training	Ministry of Social Development and Economic Security
**Enforcement**	
Drug Treatment Courts, Community Courts and other diversion programs	Ministry of Attorney General
Organized Crime Agency	Ministry of Attorney General
**Harm Reduction**	
Health and substance misuse referral services	Ministry of Health<br>Ministry for Children and Families<br>Vancouver/Richmond Health Board
Needle Exchanges	Ministry of Health<br>Vancouver/Richmond Health Board
Pilot accessible (low threshold) support programs	Vancouver/Richmond Health Board<br>Ministry for Children and Families
Shelter and Housing Options for drug users	BC Housing

1.6 The Federal Government's Responsibility

Consistent with the federal government's mandate and responsibility for the criminal justice system, health promotion and research, and immigration, significant responsibility for actions and funding of treatment and enforcement rests with federal government ministries such as the Department of Justice Canada and Health Canada.

The following outlines general areas of responsibility. More detail follows in Sections 9 through 14.

**Prevention**	
Health Promotion	Health Canada
**Treatment**	
Drug Treatment Research, clinical trials and evaluation	Health Canada<br>Canadian Institute for Health Research<br>Canada's Drug Strategy
Employment and Training	Human Resources Development Canada
**Enforcement**	
Domestic and International Drug Enforcement	RCMP<br>Canada's Drug Strategy
Drug Treatment Courts, Community Courts and other diversion programs	Department of Justice Canada
**Harm Reduction**	
Development of innovative pilot projects	Health Canada
Pilot accessible (low Threshold) support programs	Health Canada

2. Background

For the past several years community organizations and individuals have been calling for a concerted effort to address the issue of substance misuse in Vancouver and its attendant negative impact on communities and neighbourhoods. The purpose of this discussion paper is to provide an overview of the work that has already been done and a city-wide framework for action. While many key reports focus on both alcohol and drug misuse, the focus of *A Framework for Action* is on reducing harm to communities and individuals caused by the sale and misuse of illicit drugs.

Under the *Vancouver Agreement,* signed in March 2000, the Governments of Canada, British Columbia, and the City of Vancouver committed to work together to develop and implement a coordinated strategy to promote health and safety throughout Vancouver. This five-year collaboration acknowledges that a comprehensive drug strategy must be linked to housing, employment, and social and economic development. The recent *Vancouver Agreement* announcements are a good beginning to creating a healthier and safer community in the Downtown Eastside. But they are only a start, and communities throughout the Lower Mainland need a comprehensive plan of action to deal with substance misuse. (For more detail on the *Vancouver Agreement,* please refer to Appendix B).

In addition to the *Vancouver Agreement* initiatives, *Vancouver's Coalition for Crime Prevention and Drug Treatment* held a series of five public forums on a "continuum of care" approach to drug treatment. The forums brought together over 350 people from across the community for panel discussions and public dialogue on the four-pillar approach of prevention, treatment, enforcement and harm reduction. These forums followed two years of discussion through more than 33 meetings in which community members and more than 63 Coalition partners talked about issues and weighed possible approaches to drug addiction and property crime in Vancouver.

In addition to incorporating input from the public and the Coalition, *A Framework for Action* includes findings and recommendations from the Vancouver Agreement Substance Misuse Strategy, the Kaiser Youth Foundation, the Lower Mainland Municipal Association, the Federal and Provincial Governments, the Vancouver/Richmond Health Board (V/RHB), the Ministry for Children and Families, Vancouver City Police, the Chief Coroner's Office, and many other local and international organizations.

The research has been done and the groundwork laid for a comprehensive, workable framework for action.

Drug dependence is a serious health issue and must be treated with the same urgency and level of care as any other health problem, AND in collaboration with other health services.

Vancouver Agreement:
Comprehensive Substance Misuse Strategy, 2000

3. Drug Use In Vancouver – Trends

3.1 Drug Overdose - a Growing Crisis

Drugs such as heroin have long been available in Vancouver and other Canadian cities. However, the increase in purity of heroin around 1992 and the introduction of cheap cocaine and crack-cocaine to the city in the early 1990s had a devastating impact on individuals and communities.

For the first nine months of 2000, Vancouver has had 87 overdose deaths, three more than the first nine months of 1999.

British Columbia Coroner's Office

The total number of overdose deaths in British Columbia increased from 39 in 1988 to 331 in 1993 (Cain report, 1994.) In 1993, Vancouver had 201 deaths due to drug overdose, primarily associated with illicit drugs. In 1998, the number was 191. The number of illicit drug overdose deaths in Vancouver has averaged 147 per year for the last seven years (CCENDU report, 2000).

It is critical to understand that to die of a drug overdose, a person does not have to be a heavy user of drugs. Since little is known about the properties of street heroin, users often do not know the strength of the drug they are injecting or smoking. Casual users often do not realize that mixing heroin and alcohol dramatically increases the risk of overdose.

In 1998 overdose from injection drug use was the leading cause of death for adult males age 30–49 in British Columbia (Millar Report, 1998). While heroin-related deaths averaged 70 per year since 1991, cocaine has become a growing problem. The number of cocaine deaths has doubled from 10 to 20 per year as a result of the drug being injected or smoked. The death rate is highest for young males age 25–44. (CCENDU Report, 2000). Many of these deaths could have been prevented had the proper resources and policies been in place (Cain Report, 1994 and CCENDU Report, 2000).

$11,000 — The median household income in the Downtown Eastside in 1996.

CCENDU Report, 2000

Illicit Drug Overdose Deaths in Vancouver
Sources: Cain Report, 1994 and CCENDU Report 2000

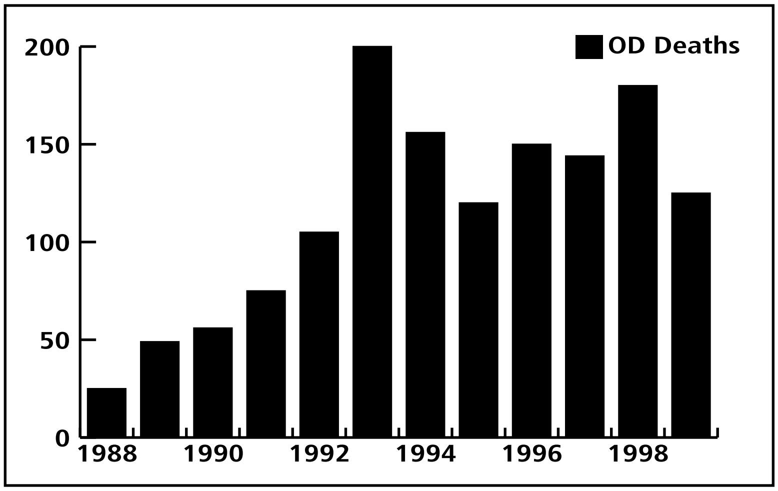

3.2 HIV and Hepatitis C Infection

Overdose deaths are only part of the picture. Injection drug use is responsible for half of new HIV infections recorded and 80% of newly identified hepatitis

C cases. While reported cases of HIV infection in Vancouver are down from 587 in 1992 to 314 in 1998, this number is misleading. HIV is not a reportable infection in BC. Only AIDS cases (HIV infections in persons who meet the criteria for AIDS) are reportable. And while there has been a dramatic decrease in AIDS, this is likely due to improvements in HIV therapy, which means that persons with HIV experience a much slower progression to AIDS. So the decrease in reported AIDS cases does not indicate a decrease in the rate of HIV infection (CCENDU Report, 2000).

The Vancouver Injection Drug User Survey (VIDUS) reports that new incidences of HIV infection among injection drug users has fallen from a high of 19 percent in 1997 to between three and five percent for the past two years. This is still considered a high figure compared with many European cities with similar numbers of drug users that report 1 percent or less for new incidences of HIV infection among their populations of users (VIDUS, 2000).

People are also becoming infected at a much younger age. In Canada, the average age has dropped from 32 to 23 years (Bognar et al, 1998; Health Canada, 1997).

Hepatitis C infection has risen dramatically. Since 1994, close to 2,000 cases have been reported annually in the Vancouver/Richmond Regional Health Board (VRHB) district. It is believed that roughly 70 percent of those cases are acquired through injection drug use (CCENDU, 2000). These numbers represent individuals from all areas of the health region and from all walks of life.

3.3 Drug Misuse Among Youth

Rebellion, risk-taking, and experimentation are common behaviours among youth. But cheap drugs, easy access and peer pressure have led to a marked increase in young users. The growing popularity of "club" drugs such as ecstasy and methamphetamine have increased the risks that individuals take with drugs. And with media advertising promoting conformity and the need to fit in, peer pressure is worse than ever. Smoking heroin is also a growing phenomenon among teenagers and youth who see it as a cool thing to do. Educational programs that use scare tactics or promote zero tolerance may increase the allure of drug use and the desire to rebel.

Often, young users come from a background of poverty, physical and sexual abuse, and substance misuse, particularly alcohol. Even before any influence by their peers, they are very much at risk of developing problems with alcohol or drugs. Experimentation with drugs and altered states of consciousness can start out innocently and often does not lead to harm. However, young people can develop severe drug dependence and can be suddenly forced to navigate a complex criminal environment in order to obtain the substance they are physically dependent upon to make it through each day.

In 1997, the number of homeless street youth in Vancouver was estimated at 300 to 500 in the peak summer months (Chand et al, 1997) and it is increasing. Without the appropriate social and health supports in place, which provide

Of our 17 year-old students,

80% admit to having tried alcohol recently

and of those 44% have been binge drinking

at least once in the last month

... Experimentation with marijuana

has risen by 50% in the last five years

to 58% of the 17 year-olds interviewed.

Kaiser Report,2000;
Adolescent Health Surveys,
McCreary Centre Society.

opportunities for street youth to get assistance and become involved in meaningful activities during this difficult time in their life, their social situation can deteriorate rapidly. These young people are at high risk of becoming involved with gangs, prostitution and the drug trade.

How do I get a young offender off drugs through treatment programs when the service isn't there?

Cain Report, 1994

Among those working with street youth, there is a perception that conditions on the street are generally deteriorating. This has been confirmed in a recent study by the City of Vancouver Social Planning Department (Homeless Street Youth in Downtown South: A Snapshot Study, 2000), which indicates that:

- Street youth are moving from involvement with the street to entrenchment on the street more rapidly.
- The number of street youth dying of drug overdose, HIV, suicide, or murder has increased over the past 18 months.
- There is a gradual and consistent increase in the number of female youth on the streets.
- There is an increase in the use of hard drugs by street youth.

More detailed studies should be undertaken to explore these preliminary indications. The linkages between drug and alcohol misuse and the sexual exploitation of children and youth are alarming and require immediate and ongoing investigation to develop a comprehensive approach to this issue.

3.4 Inadequate Treatment Services

Treatment for seriously addicted users is still inadequate. In the past ten years there has been little significant expansion of drug and alcohol addiction services despite the growing problem with substance misuse. In some areas there has even been a reduction. For example, the Pender Detox Centre in Vancouver was closed in 1995.

The costs of inaction are soaring. An estimated $100,000 is required per HIV infection in direct costs alone.

Bognar, Legare, Ross, 1998

A report by the Ministry of Children and Families states the dire need for resources "…not only more of the same, but new and innovative approaches need to be developed to attend to emerging trends and issues." (Review of Alcohol and Drug Services in Vancouver, May 1997).

In the summer of 2000, responsibility for alcohol and drug services for adults was transferred to the Vancouver/Richmond Health Board. The Vancouver/Richmond Health Board immediately began to expand services for people with drug and alcohol problems and mental illness. However, few new funds have been found to date for a significant expansion of services in the region. The Vancouver/Richmond Health Board has been working through the *Vancouver Agreement* to outline a comprehensive substance misuse strategy for the region to be implemented as funding is secured. The *Vancouver Agreement* announcement of new and expanded health services for Vancouver in September 2000 was the first step in this process.

In Vancouver, there are few services that deal with the most problematic addicts. In addition to long waiting lists, many programs require abstinence before an addict is granted entry. Often there is a cost involved, which users

must pay out of their own pockets. And if someone has a relapse, they are kicked out of the program and have to wait again before they are allowed to re-enter some programs.

Drug users who experience relapse are often treated in a punitive way by treatment providers. Too often in the current treatment system individuals are set up to fail. This inevitably leads to increased harm to the community and individuals. In fact, relapse is a part of the healing process and needs to be clearly understood and supported by treatment providers. Assistance for those who relapse must be built into treatment and support programs.

3.5 Crime and Substance Misuse

There is clearly a relationship between substance misuse and crime. However the extent of this relationship is difficult to pinpoint. Crimes that drug dependent individuals commit vary a great deal. Many addicts sell drugs to finance their own drug use. Some individuals resort to the sex trade to raise money for drugs. Others commit property crimes such as breaking into cars and homes, or shoplifting. Of course not all people who have substance misuse problems are criminals and, conversely, much crime is committed by those who do not have a drug or alcohol dependency.

Research indicates that many addicts commit crimes out of desperation— a clear indication that something must be done to prevent individuals from reaching this point.

Incidents of assault and property crime have been steadily decreasing in Vancouver. Recent statistics published by the Ministry of the Attorney General of BC report that crime rates for criminal code, property crime, and assault peaked in 1996 and have decreased significantly since then. However, since drug crimes only refer to possession or trafficking of illegal substances, there is currently no way to determine with any certainty how much crime is committed as a direct result of substance misuse.

This fact gives little solace to residents and business operators in areas of Vancouver where crime and public disorder seem to be a daily occurrence. Community members in these neighbourhoods have lived with the very serious negative impacts of substance misuse and associated crimes for many years and are anxious for something to be done.

Offenders, when arrested, are not tested
for alcohol or substance use…
except in cases of impaired driving.
As a result, official crime statistics
do not contain information about whether
alcohol or drug misuse was a factor
in the commission of the crime.

LMMA report, 2000:34

4. Issues, Dilemmas and Attitudes

4.1 Why People Use Drugs

Whether we like it or not, drugs are a part of modern life. Their use is more common and more insidious than many would like to admit. Aspirin, tranquilizers, caffeine, anti-depressants, alcohol, and tobacco help many people get through the day. To deal with the increasing complexity of daily life, we have become a society of substance users. Children grow up in an environment where mood-altering, pain-reducing, sleep-inducing substances are widely marketed and accepted.

Substance abuse disorders are like many other chronic illnesses (e.g. diabetes, hypertension, and asthma). They are caused by a combination of genetics, human biology, and environmental factors.

Millar report, 1998

Those who use "hard" drugs do so for many of the same reasons. Some use drugs for pleasure. Many use drugs to relieve physical or psychological pain. The mentally ill often take drugs to achieve a higher level of functioning. For those who use drugs as a refuge, they see the harm that they inflict upon themselves as the lesser of two—or perhaps several—evils. Users then become marginalized, alienated from friends and family, forced into risky circumstances, and isolated from health services and positive support.

However, most individuals use drugs for only a short period of time. And there is a growing consensus that for those who do develop a dependency on illicit drugs or legal substances the problem is primarily a health issue, rather than a criminal one. This discussion paper attempts to clarify that the four-pillar approach deals with the people who have an addiction and need treatment, while clearly stating that public disorder, such as the open drug scene, must be stopped. In short, addiction needs treatment and criminal behaviour needs enforcement.

4.2 Viewing Drug Users as Criminals

Drug users are often seen as criminals and the cause of an inordinate number of problems in society. As a society, we tend to respond to those addicted to illicit drugs with fear while tolerating a much greater number of individuals who are addicted to alcohol and tobacco, which have a much greater cost to society in general.

Even though the crime rate has been decreasing, many Lower Mainland communities perceive a growing risk of criminal victimization. A recent study found that no Lower Mainland law enforcement agencies are currently able to produce statistics on the frequency of crime related to addiction or substance misuse (LMMA report, 2000).

Whether victims of crime or not, people who live in or near the Downtown Eastside experience a different reality than what the statistics might indicate. The issue of displacement is real. Illicit drug dealing and prostitution have increased in the Downtown Eastside and surrounding neighbourhoods. The constant presence of drug dealing, drug use, and the associated risks of discarded drug paraphernalia are extremely stressful for those living in these communities. Over time, this takes its toll on perceptions of safety and well-being.

4.3 Mental Illness and Dual-Diagnosis

Many individuals who have alcohol or drug problems also have mental health problems. The frequency of dual diagnosis is well documented and poses additional challenges to health and treatment. People with mental health problems are particularly vulnerable to injection drug use and they are not well suited or well served by group interventions. (LMMA 2000; Bognar et al, 1998).

These individuals have difficulty even qualifying for treatment, and are frequently excluded from drug or alcohol treatment programs or mental health services on the basis that the presence of one disorder is an obstacle to the successful treatment of the other. (LMMA , 2000).

The move towards deinstitutionalization of the mentally ill without adequate housing, medical and support structures has left many homeless and hopeless. Mental health services are desperately inadequate. The BC Mental Health Monitoring Coalition has severely criticized the lack of funding and support, as well as the lack of progress by the provincial government in implementing the Mental Health Plan adopted in January 1998 (LMMA, 2000).

Clearly, there needs to be a greater understanding of the health issues of addiction in order to replace fear, apathy and anger with empathy and action. We need to understand just who drug users are and why they use drugs. We need to accept that they come from all ages, cultures, classes, sexes, professions and social-economic backgrounds. We must shed our stereotypical beliefs about addiction if we are going to improve how we deal with the drug problem.

Fear and lack of knowledge are two main factors in explaining the current attitude of the public towards both alcohol and drug addiction and mental disorder.

LMMA report, 2000

5. The Costs of Drug Addiction

Facing up to the problem of substance misuse means understanding the costs to society, not only in lives, health and safety, but also to the economy. If we do not invest in a comprehensive continuum of care for addictions, the human and financial costs of inaction will exceed any potential expenditures on expanding treatment and support programs for drug users in the long term. Maintaining the status quo is a much more expensive option. Compared with the rest of Canada, British Columbia fares poorly when it comes to the costs of addiction. British Columbia has the highest costs per capita for illicit drug use. A comprehensive analysis by the Canadian Centre for Substance Abuse, 1992, states: "The per-capita costs of illicit drugs range from $31 in Newfoundland to $60 in British Columbia. It is estimated that illicit drugs cost the British Columbia economy $207 million in 1992" (CCSA, 1992).

There is now evidence that, under epidemic conditions, 5 to 7 HIV infections are averted for every 100 HIV negative patients receiving methadone maintenance for a year. Furthermore, for every HIV infection averted in injection drug users in BC, a total lifetime medical cost of $145,344 is avoided.

Anderson, 2000

Vancouver has the highest cost per capita for illicit drugs. Although the costs related to tobacco and alcohol are higher, illicit drugs represent a large burden of illness-related costs, tend to involve younger victims, and may be more costly per case (Millar, 1998). If alcohol and tobacco are included, the Canadian Centre for Substance Abuse estimated the cost in 1996 to be over $2.25 billion for British Columbia (Kaiser report, 2000)

5.1 Direct Costs of Illicit Drug Use in BC

In 1997, the estimated direct costs arising from law enforcement and health care related to injection drug use and HIV/AIDS in British Columbia was $96 million annually. The cost of enforcement was 4.5 times higher than the cost of treatment. A U.S. study sponsored by the Physician Leadership on National Drug Policy indicated that for every dollar invested in treatment seven dollars are saved in health and social costs. (Background Paper on Drug Treatment Needs in Vancouver, 1998)

**Direct Health Care Costs**	**($ thousands)**
Hospitalization	$5,172
Co-Morbidity	$2,400
Residential Care	$4,854
Non-residential Treatment	$1,316
Ambulatory care	$1,458
Prescription Drugs	$1,500
Other health care costs	$ 321
Total	*$17,021*
**Law Enforcement**	
Police	$37,161
Courts	$20,020
Corrections	$20,020
Customs and Excise	$ 1,508
Total	*$78,710*
Total Direct Costs	***$95,731***

(HIV, Hepatitis, and Injection Drug Use in British Columbia - Pay Now or Pay Later. Millar, June 1998).

A recent Ontario study estimated the annual direct costs to government as $33,761 per untreated injection drug user (Millar, 1998). These costs are similar or higher in British Columbia, and they escalate every year.

In contrast, first-year treatment costs have been estimated at $21,000 per year per injection drug user. And about 50 percent of those undergoing treatment show substantial reduction in use and in criminal activity. Methadone treatment and counselling costs $4,000 per patient per year. The cost of an untreated heroin addict to society is $30,000 per year (Millar, 1998).

Each new case of HIV costs the health care system approximately $140,000. The Health Association of B.C. estimates that illicit drug use results in 2,600 hospitalizations per year and 16,000 hospital days for a total cost of $7.5 million annually (LMMA, 2000).

The cost of calling an ambulance is approximately $460 per call if an individual is taken to hospital (BC Ambulance Service). In 1998, there were 1,053 ambulance calls with a primary diagnosis of drug/alcohol overdose in the Downtown Eastside of Vancouver. This results in a cost of $484,340 for overdose calls alone in this small area of the city.

The estimates above do not include:

• health care costs for diagnostic services and preventative programs;

• costs of treating hepatitis B and C related to injection drug use;

• costs resulting from theft, property damage, etc;

• costs related to unemployment, lost productivity and social assistance (income assistance alone could cost as much as $67 million annually.)

The continuation of this epidemic represents a failure of societal values and attitudes. It is also a major cause of death and disease, leading to the waste of almost $100 million in direct government cost annually in British Columbia.

Millar, 1998

6. Working Towards Solutions— A Community Effort

What will it take for Lower Mainland Communities away from the Downtown Eastside to understand that they, too, have a serious problem, and that they cannot afford to ignore it any longer?

LMMA Report, 2000

The drug problem in Vancouver is city-wide. Substance misuse knows no boundaries. Out of the average 147 illicit drug overdose deaths that occur each year in Vancouver, only 62 are from the Downtown Eastside. 30 are in the West End and the rest, 55 overdose deaths, occur in areas other than the Downtown Eastside. Kitsilano, Kerrisdale, Point Grey, Grandview Woodlands, Shaughnessy, Mount Pleasant—no neighbourhood is immune. But each is unique and each one is affected in different ways. That is why every community must play their part in tackling substance misuse.

While many policies and strategies are clearly federal and provincial responsibilities, local communities can help to shape and implement these strategies. The involvement of local community groups, volunteer agencies and local businesses can help to catalyze effective action and coordinate efforts among municipal, provincial and federal governments.

Community involvement will ensure that the four pillar response to drug issues are:
- specific to the community,
- based on locally relevant information,
- immediate, and
- targeted to particular problems.

Vancouver already has active, committed community groups and organizations that are dedicated to realizing a workable, effective drug strategy in this city.

In the Downtown Eastside, Community Directions, a coalition of residents and organizations is currently developing an alcohol and drug strategy for the neighbourhood through a process that builds on previous work undertaken in the community.

The group From Grief to Action is an association of families and friends of drug users from across Vancouver whose purpose is to advocate for a comprehensive continuum of care for drug users, encourage support for families and friends of those struggling with addiction, advance recognition of drug use as a health issue, and promote effective prevention programs.

Drug users have organized to support each other and advocate for a comprehensive approach to dealing with drug addiction in Vancouver. The Vancouver Area Network of Drug Users (VANDU) has organized demonstrations, educational forums and lobbied all levels of government to act quickly to intervene in the epidemics of overdose, HIV and hepatitis C among injection drug users.

Kiwassa Neighbourhood House, Collingwood Neighbourhood House and Cedar Cottage Neighbourhood House all have active groups meeting to discuss strategies for responding to the harmful effects of the use and sale of illicit drugs on individuals and on the community.

Local Business organizations in Chinatown, Gastown and Strathcona have been vigorously advocating a comprehensive regional plan for drug treatment services throughout the Lower Mainland and increased enforcement efforts in the Downtown Eastside and surrounding areas.

The Vancouver Board of Trade, the Downtown Vancouver Association and the Downtown Business Improvement Association have advocated improved prevention, health and enforcement strategies and have demonstrated leadership in informing their members of the complex issues surrounding substance misuse.

The Vancouver Safety Coalition, an alliance of crime prevention and community organizations across the city has come together to focus on issues of crime and substance misuse as well as other community concerns.

The Community Policing Advisory Committee made up of board members from community policing offices is exploring the issue of substance misuse and the negative impact on Vancouver neighbourhoods in order to better advise police on community policing efforts.

These are just some of the community efforts to come to grips with the harmful effects of substance misuse on neighbourhoods in Vancouver. There are many others that have not been mentioned. Critical action must be undertaken at a local level if it is to be credible and effective within our communities. By involving those with local knowledge in the planning and implementation of a four-pillar approach to drug misuse, community efforts and collective resources can be integrated effectively into a plan of action. Involved communities that see the results of their efforts are healthier, safer communities.

7. Outcomes in the U.S., the U.K., and Europe

Cities in the U.S., the U.K and Europe have responded to the growing problem of substance misuse by adopting a wide range of treatment and harm reduction programs and enforcement strategies. In all instances, a continuum-of-care approach for drug users was undertaken and enforcement efforts were coordinated with drug strategies. The result was a marked decrease in deaths and drug-related harm. While there are many differences between the contexts of the U.S., the U.K., Europe and Canada with regard to health care funding, municipal responsibilities, law enforcement and criminal justice approaches, there are also many similarities. There is much to learn from experiences in other jurisdictions concerning how to better manage urban drug misuse through coordinated public health and public order strategies.

7.1 Portland, Oregon

In the mid 1970's the City of Portland was instrumental in creating Central City Concern, a non-profit organization that focused on inner-city individuals who were homeless, intoxicated, unstable, and most at risk of becoming seriously ill or dying. At the same time these marginalized individuals had a significant negative impact on local businesses and the public perception of safety in the streets.

Over the past 25 years, Central City Concern has developed an alcohol and drug treatment continuum that ties together a range of services from sobering and detox, transitional and permanent housing, inpatient and outpatient treatment, alcohol and drug free housing, job training, acupuncture services, SRO rehabilitation and repair projects. The success of the Central City Concern model is a result of the many partnerships that the organization has formed over the years, which have facilitated the coordination of services and support for individuals with substance misuse problems.

Central to the system that was put in place in Portland was linking stable housing with treatment for drug and alcohol addiction. The goal was to move people who were homeless and had addiction issues from the street into drug and alcohol treatment and at the same time to improve their housing conditions. The three types of housing that Central City Concern has developed includes: transitional housing as part of a drug and alcohol treatment continuum, permanent drug and alcohol free housing (dry housing) and permanent affordable housing for low income people.

Transitional supportive housing targets those that are homeless, have an addictions problem and wish to enter some form of drug treatment. If they agree to enter drug treatment then individuals can access Central City Concern transitional housing for up to 90 days. In 1998 there were 82 units of this type of housing.

The second component in the continuum of housing that Central City Concern has created is permanent alcohol and drug free housing. In 1998

there were 353 units. This type of housing is for those who wish to live in a supportive environment that reinforces their desire to remain abstinent from drugs and alcohol.

The bulk of the housing that Central City Concern has developed over the years is housing for low income individuals and families. In 1998 there were 617 of these units and work was underway on the first family housing project for the organization.

Central City Concern has played a significant role in turning around a bad situation in the mid 1970's that saw many individuals homeless, out of contact with health services and at risk of great harm to themselves and the community. In 1998 staff indicated the 30 per cent of the people who move through the continuum of treatment and housing return to the community without relapse. By linking detox and treatment with stable housing, employment projects and skills development Central City Concern has created a model continuum of care within the inner city of Portland which has had a profound effect on many individuals and has improved the quality of life in the community immensely.

Note: *Placeholder for Central City Concern Continuum of Care Map*[1]

7.2 Switzerland

In the late 1980s, Switzerland experienced a huge increase in public drug use, which resulted in large open drug scenes in the downtown neighbourhoods of its major cities. Treatment programs available at the time were high threshold and medium threshold services. Threshold refers to the eligibility criteria for entrance into programs and the state of "readiness" of individuals to participate and meet the demands of the various programs.

High threshold services are traditional abstinence-oriented therapies, residential treatment regimes, recovery houses, etc., where one has to attain a certain level of functioning before entering the program. Medium threshold services include medical and social care that have well defined therapeutic goals such as methadone programs, counselling and other types of support that also require adherence to a structured regime in order to stay in the program.

The Swiss found that with only high and medium threshold services available, they were reaching merely 20 percent of active drug users. In addition, individuals had been involved with the drug scene for an average of six years before they received any therapeutic intervention. By this time these users had become marginalized and had little contact with the health care system. Vancouver has experienced similar trends.

Over the past 15 years, the Swiss have developed and implemented a program based on the four pillars of prevention, treatment, enforcement, and harm reduction.

1. Placeholder means furtner information to come.

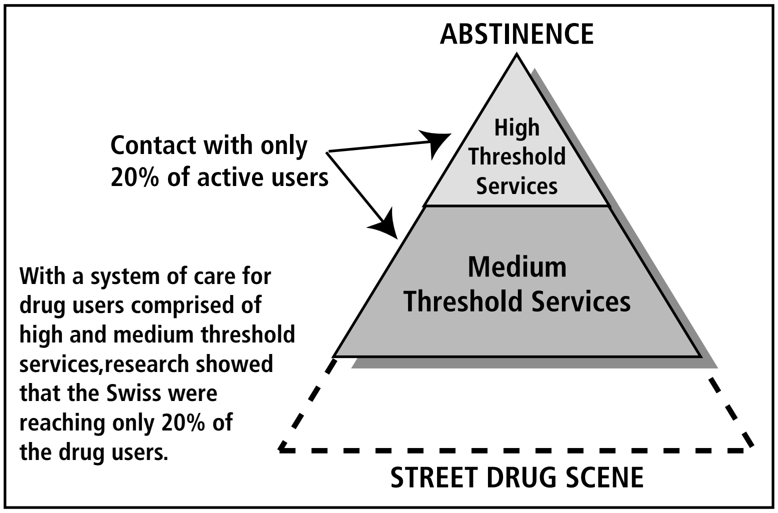

The origin of the strategy that we have developed in Switzerland is the counterproductive effects of uncoordinated actions that we experienced prior to the late 1980's.

Ruth Dreifuss, President of Switzerland. MacPherson, 1999.

7.2 a) Program Strategy

The Swiss program is a balancing act between public health and public order, with enforcement strategies complementing and assisting the efforts of health agencies. It emphasizes the importance of providing both harm reduction programs for those who continue to use drugs and treatment options for those who want to quit. Prevention and health promotion are considered to be the most important underpinnings of their strategy.

A key element of their program was the development of a range of low threshold services that are easily and immediately accessible to street drug users. The aim is to put them in contact with a continuum of care as early as possible.

Components of these low threshold services include:

- easier access to methadone;
- day centres for drug users;
- shelter beds for drug users;
- needle exchanges;
- outreach workers and programs;
- employment programs;
- safe injection sites; and,
- methadone in prisons.

Decentralization of services was part of the Swiss strategy and police support was crucial—and enthusiastic. Police finally had somewhere to direct addicts who were injecting drugs in public places. A park in Zurich that was essentially a "tolerance" area for intravenous drug users was effectively closed down. Police also agreed that drug addiction was a health issue, and that enforcement efforts should focus on apprehending the non-addicted mid-level and higher-level dealers and importers of illicit drugs.

Major municipalities in Switzerland actively lobbied the federal government to support the development of addiction services in outlying areas so that Swiss citizens who used drugs had access to services closer to home.

In addition, the Swiss undertook scientific, clinical trials whereby heroin was prescribed to a small group of chronic heroin addicts who had a history of failed attempts with other treatments, and who were unhealthy and socially

isolated. The trials took place in 17 different locations throughout the country and reached approximately 1,200 heroin users.

7.2 b) General Outcomes

The Swiss strategy got users off the street, and in many cases off drugs altogether. The development of easily accessible low threshold services also increased the need for more intensive treatment services for those who wanted out of the drug scene. As more individuals came into contact with basic health services, the need for longer-term, integrated drug treatment such as housing support, job training and ongoing post-treatment counselling also increased.

Between 1985 and 1991, the number of addicts entering long-term care facilities increased by 67 percent. A study of patients in three centres also showed that a broader range of drug users was seeking treatment earlier. However, the new diversity of patients put a greater burden on traditional treatment facilities, which were ill equipped to deal with youth, women, ethnic groups, people with HIV, etc. Programs had to be designed around these special populations.

Police resources were increased and federal legislation was introduced to prevent money laundering by criminal organizations involved in the sale and trafficking of drugs. At the end of 1998, accounts totalling Sfr 325 million had been frozen.

7.2 c) Health-Related Outcomes, 1988 - 1998

- 65% of drug users are in some form of treatment.
- 50% of the estimated 30,000 drug users in Switzerland are in methadone treatment and 15% are in abstinence-based programs.
- Many of the remaining 35% are in regular contact with harm reduction programs.
- Public consumption of drugs is no longer a major problem.

With implementation of comprehensive harm reduction and treatment programs coordinated with enforcement efforts the Swiss claim that 65% of active users are in some form of treatment. The remainder are in contact with harm reduction programs

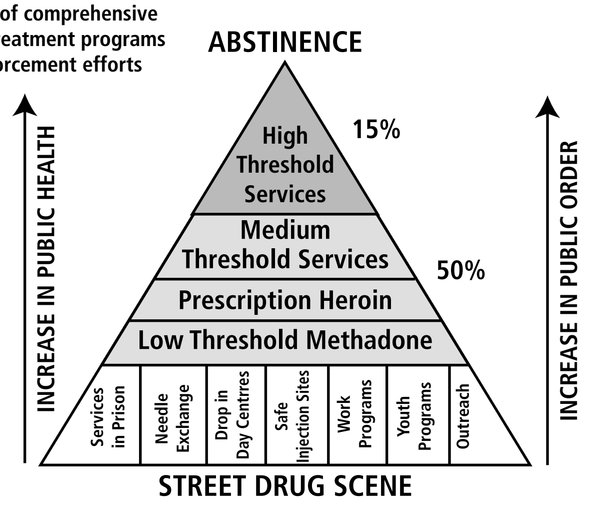

7.2 d) Outcomes of Heroin Prescription Program

Of the 1,200 individuals who participated for two years in the 17 heroin prescription programs throughout the country, results were also extremely positive:

(Ed. note: About heroin trials;)

A cost-benefit analysis revealed that the Swiss government saved 46 Swiss francs per patient per day due the increased health of the patients and the reduced cost of criminal investigations and prison time.

MacPherson report, 1999

- Income from illegal and semi-legal activities decreased from 59% to 10%.
- Criminal offences were reduced by 60%.
- Permanent employment increased from 14% to 32%.
- Unemployment fell from 44% to 20%.
- Participants' physical and mental health improved dramatically.
- Illicit heroin and cocaine use decreased significantly, as did contact with the drug scene.
- Housing situations improved and stabilized.

Participants attended treatment three times per day. As a result, one of the most dramatic successes of the heroin prescription program was social integration. Their regular visits with doctors, nurses, psychiatrists, and social workers initiated a network for human contacts that could guide users beyond the closed loop of the drug scene.

7.3 Frankfurt, Germany

With a population of 676,000, Frankfurt is similar in size to Vancouver. In the '70s and '80s, Frankfurt had very similar drug problems to Vancouver: an open drug scene in the downtown core, a 25 percent HIV rate among intravenous drug users, and an overdose death rate that hit 147 in 1991. In 1989, the City of Frankfurt created the position of Drug Policy Coordinator with a staff of seven people who had a mandate to work with city staff, public health authorities, the Justice, Social Services and Housing Departments, and the State Police to develop a coordinated drug strategy.

7.3 a) Program Strategy

The strategy Frankfurt adopted was similar to the Swiss strategy in that it focused on the development of a range of low threshold, harm reduction and treatment programs for addicts. The approach is referred to as "help and suppression" and combined health initiatives with a policing strategy that did not tolerate open consumption of drugs and moved addicts to crisis centres to either inject indoors or to get a range of treatment and shelter (Clarke, 2000).

Implementation began in 1991, with the introduction of low threshold methadone programs designed to reach an additional 1,000 users. The second phase involved a comprehensive harm reduction program with 300 additional shelter beds for drug users, five multi-service crisis centres, expanded needle exchange, education and outreach. These were instituted at the same time the police closed down the open drug scene.

Drug courts were introduced in the mid 1980s. Individuals who were sentenced to three years or less could choose treatment, including methadone, instead of prison.

In 1994, the Attorney General of the State of Hessen issued a legal opinion that safe injection rooms were not against the German drug laws. This development in legal opinion allowed the establishment of five safe injection rooms over the following two years.

With the increase in crack cocaine use in recent years a pilot program of

outreach to crack addicts was set up in one of the crisis centres. The main goal of the program is to make contact with this group of drug users and help to integrate them into other drug or youth services. "The program has been more effective than anyone thought. In the two years since it began they've introduced 200 of the estimated 400 crack users to methadone and about 60 crack users to detox and treatment, of whom only two are back on the street." (Clarke, 2000)

The final phase of their program, slated for 2001, is to establish a clinical research trial of heroin-assisted therapy in several cities in Germany.

7.3 b) Health-related Outcomes - 1997

Again, a coordinated approach using enforcement measures and harm-reduction programs had very positive outcomes.

- Public consumption of drugs was greatly reduced.
- The number of street users dropped from 1,500 to 100-200 throughout the city.
- The HIV rate among injection drug users decreased from 25% to 14%.

Current statistics show a major decrease in drug-related activity over the past seven years.

	1992	1999
number of open drug injectors per day	1,500	50
number of addicts	10,000	3,000
number of dealers	5,000	1,400
number of non-resident addicts	65%	20%

Annual Drug Overdose Deaths

(Frankfurt Police Department Annual Report, 1997)

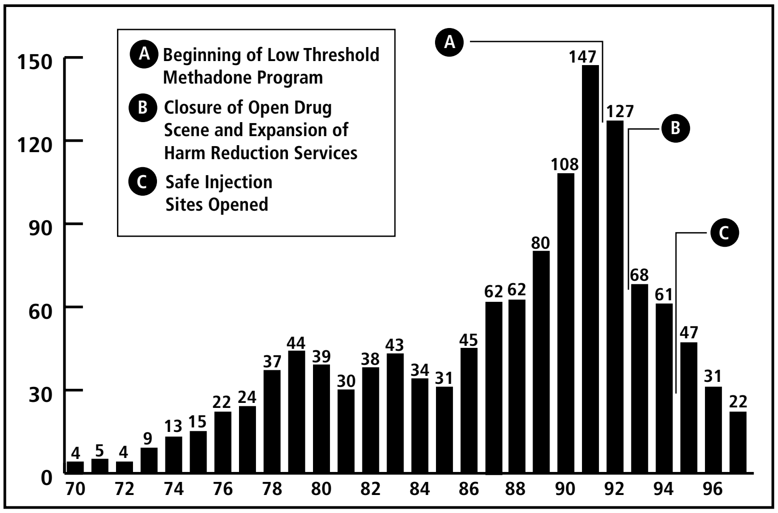

7.3 c) Crime-related Outcomes

- Theft from auto was reduced by 36%
- Break-ins were reduced by 13%
- Grievous bodily harm was reduced by 19%
- Registered first-time users of hard drugs decreased by 39%
- By 1997, drug related court cases had dropped by 15%

Frankfurt's comprehensive program is widely condoned in Germany and receives financial support from the local business community.

7.4 Liverpool

The Merseyside region of England, which includes Liverpool and surrounding area, is a successful example of a comprehensive approach to substance misuse. Merseyside has the second highest rate of drug use in England, yet it has managed to minimize the harms that the illicit drug scene has on Liverpool. Since 1986, the Merseyside region has pursued a pragmatic response to HIV infection by treating it as a public health problem. Merseyside never had an "open" drug scene such as the one in Vancouver or those in major European cities in the late 1980s and early '90s. The heroin epidemic in the Merseyside region began in the suburbs and drifted into other neighbourhoods over time.

The imperatives of the strategy are that services should make contact with drug users, maintain contact and make changes in behaviour.

Merseyside Advisory Council, 1988.

"We have no hesitation in concluding that the spread of HIV is a greater danger to public health than drug misuse. Accordingly, services which aim to minimize HIV risk behaviour by all available means should take precedence in development plans" (Merseyside Advisory Council on the Misuse of Drugs, 1988).

This focus on HIV prevention as opposed to substance misuse itself was significant in that it clearly directed public heath professionals to target the most serious harm. Dependency on heroin, cocaine or alcohol is a serious problem, but contracting HIV is a deadly one. The Merseyside program has evolved into one of the most successful in the world in reducing drug-related harm to individuals and communities.

Services implemented include:
- decentralized needle exchange programs in several neighbourhoods
- outreach workers
- prescribing clinics where addicts can obtain oral and injectable methadone, and where a small percentage can obtain injectable heroin, counselling and support in drop-in facilities.
- criminal justice interventions including: arrest referral schemes; bail support that allows a client who gains bail to move towards a treatment program; and drug treatment and testing orders, a probation initiative aimed at helping drug-using offenders by offering treatment programs rather than incarceration.

The success of the Merseyside has been resounding. In a population of 7,000 intravenous drug users there are only 20 known users that have contracted HIV.

Andrew Bennet, H.I.T.
1999

The success of the Merseyside program has been unquestionable. In a population of over 7,000 injection drug users, only 20 have contracted HIV. One of the main reasons for the program's success is that the police were brought into the picture early on in the planning of health services. Merseyside police not only support the harm reduction and treatment programs, they have the second highest number of arrests and charges for drug offences in England. However, there is a clear distinction between saving lives and enforcing the law in police practice.

Note: Placeholder for information on budgets for European programs.

8. A Framework for Action: A Four-Pillar Approach

The **purpose** of this *A Framework for Action* is to:

1) Provide the City of Vancouver and its citizens with a framework for action that compels the provincial and federal governments to take responsibility for issues within their jurisdiction.
2) Show which levels of government are responsible for actions to achieve the goals in the framework.
3) Clarify Vancouver's drug problems and establish appropriate, achievable goals.

Goals are presented along two themes: those that are achievable under current legislation and policy, and those that could be achievable with legislative and policy changes. This draft discussion paper includes four major goals and 31 actions to achieve those goals. The estimated cost of these actions is $20 to 30 million per year. A summary of the goals and associated actions including responsible agencies is in Appendix A.

The four **goals** of *A Framework for Action* are:

(1) Provincial and Federal Responsibility: To persuade other levels of government to take action and responsibility for elements of the framework within their jurisdiction by encouraging a regional approach to the development of services, and by demonstrating the city-wide, regional, national and international implications of the drug problems in Vancouver. This is the overarching goal and the key element to achieving the following three goals:

(2) Public Order: To work towards the restoration of public order across Vancouver by reducing the open drug scene (particularly at Main and Hastings), by reducing the negative impact of illicit drugs on our community, by reducing the impact of organized crime on Vancouver communities and individuals, by providing neighbourhoods, organizations and individuals with a place to go (such as a *healthy-city* office) with their concerns related to safety, criminal activity, drug misuse, and related problems, and by implementing crime prevention techniques to increase public safety.

(3) Public Health: To work towards addressing the drug-related health crisis in Vancouver by reducing harm to communities and individuals, by increasing public awareness of addiction as a health issue, by reducing the HIV/AIDS/hepatitis C crisis, by reducing overdose deaths, by reducing the number of those who misuse drugs, and by providing a range of services to groups at risk such as youth, women, Aboriginal persons, and the mentally ill.

Once addicts are engaged in care at some point on the continuum between drug use and abstinence, we can address their substance dependence and offer help for other medical and psychological problems.

Lower Mainland Municipal Association, 2000

(4) Coordinate, Monitor and Evaluate: To advocate for the establishment of a single, accountable agent to coordinate implementation of the actions in this framework, and to monitor and evaluate implementation through senior representatives of the Vancouver/Richmond Health Board, the Vancouver Police Department, the City of Vancouver, the BC Centre for Disease Control, the Ministry for Children and Families, the Office of the Attorney General, and community representatives.

A Framework for Action includes actions that have been recommended in various forms and degrees by most groups and agencies referred to in this document. What is unique is the focus and emphasis. Vancouver's four-pillar approach is based on prevention, treatment, enforcement and harm reduction. All of these elements are integral to creating a comprehensive, workable, coordinated response to substance misuse. It is a community response that combines street level intervention and longer-term support and treatment with education, outreach and enforcement. In other words, everyone working together under the same mandate: a safer, healthier community. Issues for discussion include:

- Drug use is often for a short period in a person's life and therefore we must do everything we can to prevent harm such as HIV, hepatitis C or death by overdose from occurring.
- Chronic addiction can require long periods of sustained support and a variety of services.
- Drug addiction is a health issue, not just a criminal justice one.
- The environment has changed and the same old approaches do not, and will not, work.
- Innovative programs and methods have demonstrated success in other jurisdictions, and should be considered in the development of pilot programs that will assist in implementing long-term actions.

The four-pillar approach is a framework that ensures a continuum of care for those suffering from substance addiction and communities impacted by those same people. It promotes realistic prevention and education programs; it insists that treatment services for those who develop substance dependencies be readily available; it helps to reduce harm to communities and individuals as a result of substance misuse. And, it recognizes that enforcement is crucial—to reducing drug-related criminal activity and to coordinating a response to the negative effects of the drug trade on local communities by supporting public health programs and referring offenders to drug services. It advocates a balance between public order and public health.

A Framework for Action recommends the involvement of all stakeholders in the design, monitoring and evaluation of these programs and services, and each pillar is an interlinking part of the continuum of care. It seeks to bring together the diversity of views and issues surrounding substance misuse so that we can build a consensus for action.

9. Provincial and Federal Responsibility

Goal 1. Provincial and Federal Responsibility: To persuade other levels of government to take action and responsibility for elements of the framework within their jurisdiction by encouraging a regional approach to the development of services, and by demonstrating the city-wide, regional, national and international implications of the drug problems in Vancouver.

9.1 Actions

Regional and National Drug Strategy

1. The **Provincial ministries responsible** implement policy that ensures municipalities throughout British Columbia support the development of a full range of drug and alcohol services.

2. The **Provincial Government** implement a policy framework for reducing the harm to the community and individuals associated with alcohol, tobacco and illicit drugs to guide and inform municipal decision makers in determining priorities for action.

3. The **Federal Government** take strong leadership in the following areas:

Note: Some actions in the following section may require legislative and/or regulatory changes in order to be implemented. These and others are in italics and are noted in Appendix E.

- *Review existing laws with regard to illicit drugs, organized crime, gathering of evidence in drug cases and protection of youth.*
- Implement new money laundering legislation.
- *Review existing laws and procedures to deal with refugee claimants who are engaged in the illegal drug trade.*
- *Initiate research and development of alternative pharmacotherapies for drug addiction including: Levo-alpha-acetyl-methadol (or, LAAM, a derivative of methadone that is long-acting), Buprenorphine (an alternate therapy for heroin users), Amphetamines and other drugs to treat cocaine addiction.*
- *Provide leadership in the development of national research into the feasibility of such initiatives as: heroin-assisted treatment, safe injection or consumption rooms, low threshold methadone prescribing practices and other innovative approaches to addiction treatment and the reduction of drug-related harm to individuals and communities.*

10. Pillar One - Prevention

Any prevention program to be successful must be credible, well communicated, and reinforced over time. That's not happening in British Columbia.
The resources and the priority devoted to prevention are, to be kind, miniscule.

Kaiser report, 2000

10.1 Defining Prevention

Prevention strategies consist of three main approaches: primary prevention, which attempts to prevent substance use altogether or delay the onset of substance use; secondary prevention interventions aimed at the early stages of substance misuse before serious problems have developed; and tertiary prevention interventions, which focus on preventing serious harm to individuals who have become addicted to drugs. Many interventions within the areas of secondary and tertiary prevention can also be referred to as harm reduction. This paper acknowledges that in certain contexts different language will be used to describe what are essentially efforts to prevent further harm or minimize the harm associated with substance misuse.

10.2 Problems with Current Prevention Programs

Prevention programs can do more than inform individuals about the problems associated with drug use. They are an opportunity to build awareness about why people use drugs and alcohol and what can be done to curb or avoid addiction. The problem with many programs now in place is that they are not evidence-based, and ineffective methods are still widely used (No Further Harm, British Columbia Medical Association, 1998.)

As well intentioned as they are, the programs that do exist are often poorly coordinated, under-resourced and scattered among education, social services, law enforcement, health promotion, and drug prevention agencies. They often lack clear objectives and specific targets. Rivalry among service providers is seen as an obstacle (LMMA, 2000: 44). In addition, there has been a dire lack of funding for primary prevention programs across the province. Primary prevention of substance misuse is a major part of every official regional health plan, and is a key element of the *Vancouver Agreement*. Yet there are currently no provincial or regional prevention plans or goals in place (LMMA, 2000: 44).

When I was in high school they had these so-called drug education classes. They told us if we used marijuana we would become addicted. They told us if we used heroin we would become addicted. Well, we all tried marijuana and found we did not become addicted. We figured the entire message was b.s.
So I tried heroin, and used it again and again, got strung out, and here I am.

Heroin addict, in Rosenbaum, 1999

Even the most successful programs often fall short of their goals, either because the goals are unrealistic or because the programs are targeted to the wrong group of individuals.

In schools, the topic of "Substance Abuse Prevention" is allocated to 1/18th of the Career and Personal Planning Programs and is often delivered after children have begun making potentially harmful choices. "With the age of first use dropping younger and younger, it's too often the case that by the time many students are beginning their classroom education about substance use and abuse, they're already using." (Kaiser report, 2000)

10.3 Attitudes around Prevention

The social aspect of substance misuse is often ignored. Social drinking is part of our daily life. It is not only condoned, it is highly advertised. Similarly, drug taking frequently takes place as part of a social group. Among youth it has become a part of adolescent exploration and experimentation. Many individuals—youth and adults alike—take drugs because they enjoy the experience.

However, most prevention literature assumes that drugs are used to assuage the troubles in one's life, and many prevention programs are abstinence-based, with unrealistic goals and moralistic messages that tend to alienate rather than educate, particularly with respect to youth. Prevention approaches must acknowledge that while recreational drug use does not always result in harmful behaviours, it can lead to them.

It is important to recognize that educational approaches have very little impact on convincing current users to stop. The school programs and the community interventions … are more effective among young non-users and experimenters than frequent users or those who abuse.

Paglia and Room, 1998:24

10.4 Prevention Programs – What Works

The Lower Mainland Municipal Association's recent report *Towards a Lower Mainland Crime and Drug Misuse Prevention Strategy* identified elements essential for effective primary prevention:

- realistic, achievable objectives;
- identification of who the program is targeting;
- strong leadership;
- effective coordination among various agencies;
- expertise in evidence-based strategies; and,
- long-term commitment of funding and resources.

In fact, these elements should be used as a guide for all four pillars of *A Framework for Action*. Programs that work best are multi-level community approaches that include participation from a range of sectors including schools, families, workplaces, places of worship, secular organizations, governments, and the media. The goals of prevention must be clear and tailored to specific populations, and they should focus as much on the underlying causes of addiction as on prevention itself.

10.5 Programs for Youth

In particular, prevention programs for youth must recognize that drug use does not automatically lead to a downward spiral of addiction. From their point of view, it is a often a social and exploratory activity.

Youth prevention programs should focus on their particular context, where young people are in their personal development, the potential dangers and the immediate risks involved with substance misuse, and what should be done if problems occur. Young people are generally concerned about the here and now, so prevention programs should also address what they care most about, such as daily and weekend activities, graduations, and decisions they face about their immediate future.

Drug education in most schools … almost never teaches [young people] the basic information about how to minimize the risks of using drugs if they insist on trying them.

Gardner, The Vancouver Sun, Sept. 12, 2000

It is also clear that information-only approaches are not sufficient and that young people should have input into the development of prevention programs. Prevention programs for youth that focus on the immediate risks of substance misuse are regarded as much more successful than those that have a goal of preventing long-term drug and alcohol use.

Some excellent evidence-based teaching resources have been developed in BC, including resources for cultural groups in their respective languages. For example, Alcohol-Drug Education Service, a Vancouver-based organization, has produced a drug education program approved by the Ministry of Education for grades six and seven. The program, called *Making Decisions*, provides interactive learning that promotes life skills and critical thinking,

Goal 2.Public Order:
To work towards the restoration of public order across Vancouver by reducing the open drug scene (particularly at Main and Hastings), by reducing the negative impact of illicit drugs on our community, by reducing the impact of organized crime on Vancouver communities and individuals, by providing neighbourhoods, organizations and individuals with a place to go (such as a healthy-city office) with their concerns related to safety, criminal activity, drug misuse, and related problems, and by implementing crime prevention techniques to increase public safety.

Goal 3.Public Health:
To work towards addressing the drug-related health crisis in Vancouver by reducing harm to communities and individuals, by increasing public awareness of addiction as a health issue, by reducing the HIV/AIDS/hepatitis C crisis, by reducing overdose deaths, by reducing the number of those who misuse drugs, and by providing a range of services to groups at risk such as youth, women, Aboriginal persons, and the mentally ill.

and shows students how to recognize and deal with peer pressures and influences that may lead to substance abuse (LMMA, 2000).

However, many of these resources are not reaching all of the schools that need them, and substance abuse prevention comprises a very small part of the education curriculum—as little as two to three hours per year (LMMA, 2000; Kaiser Youth Foundation, 2000).

10.6 Prevention –Actions

Note: Some actions in the following section may require legislative and/or regulatory changes in order to be implemented. These and others are in italics and are noted in Appendix D.

4. Support and fund a community led process that increases the ability of neighbourhoods within Vancouver to respond to the negative impacts of substance misuse in their communities. This process should be led by community and resident organizations in Vancouver with the goals of: increasing the awareness of substance misuse; providing information in a variety of languages on the four-pillar approach and crime prevention techniques; and supporting community-based responses to the misuse of drugs and alcohol.
   Lead Agency: City of Vancouver
   Partner Agencies: Vancouver/ Richmond Health Board, Ministry for Children and Families, National Crime Prevention Centre, Private Foundations
5. Establish a prevention/education task force to develop a pilot, city-wide school curriculum for elementary and high schools, and a public education campaign to be delivered in community centres, neighbourhood houses and through the mass media. Members would include the Vancouver School Board, Ministry of Education, Ministry for Children and Families, Vancouver/Richmond Health Board, City of Vancouver, Vancouver Park Board, Vancouver Police Department, substance misuse prevention organizations, Grief to Action Society (families and friends of addicted children) and community representatives.
   Lead Agency: Ministry for Children and Families
   Partner Agencies: Ministry of Education, Vancouver School Board, Vancouver/Richmond Health Board, Vancouver Police Department
6. *Explore options for distributing BC Benefit cheques throughout the month in order to reduce harm to individuals and the community by decreasing the sale and use of drugs and alcohol at one time during the month by those on BC Benefits who suffer from addiction and mental health problems.*
   Lead Agency: *Ministry of Social Development and Economic Security*
7. Consider the creation of a Healthy City Office within the City of Vancouver in order to support a coordinated response to community health and safety and crime prevention in the city and to promote and support projects that work towards creating healthier and safer neighbourhoods within Vancouver.
   Lead Agency: City of Vancouver
   Partner Agencies: Vancouver/Richmond Health Board, Ministry for Children and Families, Vancouver Police Department.

11. Pillar Two - Treatment

11.1 Defining Treatment

Treatment refers to a series of interventions and supports that enable individuals to deal with their addiction problems, make healthier decisions about their lives, and eventually resume their places in the community. To successfully help an individual through this process, a continuum of treatment with multiple points of contact is required for treatment to be effective. Basic primary health care services are often the entry point for individuals into more specific drug treatment approaches. These include detox, methadone, outpatient and residential treatment programs, dual diagnosis programs, as well as programs for women, Aboriginal people and other ethnic populations. A range of treatment options that target different populations is essential.

The proportion of Aboriginal people represented in the Vancouver Injection Drug User Survey (VIDUS) is particularly high, especially the number of women. Thirty-nine percent of the women, and 18 percent of the men in the VIDUS study are of Aboriginal origin (CCENDU 2000). In a recent study conducted by the Vancouver Police Department that focused on interventions with street involved youth by two special youth units 41 per cent of 283 interventions involved Aboriginal youth, 31 per cent female and 10 per cent male. The majority of these interventions were not due to criminal charges but were as a result of the officers determining that the child's health and safety was in immediate danger. (Vancouver Police Department, 2000). The preceding statistics underline the need for special strategies to deal with Aboriginal people with addictions and Aboriginal youth at risk.

In addition, there must be services to support people before and during treatment, such as harm reduction programs, shelter and housing, as well as programs to support them after treatment, including ongoing medical care, employment, and life skills training. Early intervention is a crucial aspect of any treatment system. The earlier that action is taken in an individual's substance misuse the better the chance that the harm to the individual and the community will be minimized or eliminated altogether. The harsh reality for many who become addicted is that they are increasingly marginalized as treatment systems repeatedly fail to provide the support they need.

Investing in a broad range of treatment services not only makes sense on humanitarian grounds, it also makes sense economically. The Alberta Alcohol and Drug Abuse Commission estimates that each dollar invested in treatment saves or returns $7.14 after one year in increased productivity as well as health and justice system costs," (Kaiser report, 2000:3).

A much-quoted RAND Corporation study in the United States compared the effectiveness of four types of drug control in reducing cocaine consumption. The four areas were: domestic law enforcement (arresting and imprisoning buyers and sellers), interdiction (stopping drugs at the border), source control (attacking the drug trade abroad), and drug treatment. The RAND study showed that treatment was seven times more effective than law

The simple fact is: there is not enough of anything. There are waiting lists for everything and we are chronically under-serving many.

Ministry of Children and Families, 1997 report.

Many participants in both the single parents group and the First Nations group expressed fear that an attempt to access services would result in the loss of their children.

Community Voices report

enforcement, 10 times more effective than interdiction, and 21 times more effective than attacking drugs at their source (Kaiser report, 2000).

11.2 Problems with Treatment

In Vancouver there is an urgent need for expanded treatment resources. Existing services are both inadequate and poorly coordinated. Currently, treatment services are spread between private and government agencies, and fragmented among health regions, mental health services, several provincial ministries, probation and prison services, and services delivered by school districts. These need to be streamlined and coordinated.

The idea that to offer alcohol and drug services within one's community is to invite all the undesirable problem people from other communities to move into the neighbourhood is a pretty lame excuse for inaction and irresponsibility.

LMMA report, 2000

Other obstacles to a continuum of care approach to treatment include:
- lack of availability of treatment and long waiting lists;
- poor evaluation of existing programs and the tendency to throw more money into programs that don't work;
- lack of early intervention, even though studies have shown that this is crucial to preventing a lengthy and harmful problem with drugs or alcohol;
- fragmented service structures;
- current treatment programs do not provide service for the most difficult street-involved addicts who continually remain isolated and marginalized from the treatment system;
- lack of user involvement in evaluating programs.

Clearly, treatment resources in Vancouver are insufficient. One only has to visit the streets of the Downtown Eastside, or talk to the parents of addicted youth who cannot find appropriate treatment in Vancouver, to see that the health of many of the drug users is extremely poor and that they are only in contact with health services on an emergency basis.

11.3 Creating a Continuum of Care

Evaluation and coordination of current programs is a critical first step to developing treatment services for people with addiction problems. But we must act quickly to determine what works, what doesn't, and what resources are needed to create a progressive and responsive treatment continuum.

Different drug use and consumption patterns must guide treatment strategies. Relapse is part of the process for a great many individuals who move through treatment programs and this should be seen as part of the process rather than failure. Individual circumstances and response to treatment must be taken into consideration. For example, women must not fear losing their children if they present themselves to detox centres or treatment programs. Culturally appropriate programs must meet the needs of Aboriginal people and other cultural groups and be accessible in various languages. Finally, we must acknowledge that abstinence is not always a desired or realistic goal for all individuals, particularly long-term addicts, and that a person's treatment needs may change over time. Treatment interventions must:
- recognize the chronic nature of drug dependence;
- offer a range of options that address the multiple needs of the individual;
- respond to the needs of specialized populations;

• identify different pathways to treatment; and
• reduce obstacles to treatment for special groups, such as women, youth, Aboriginals, people with HIV and/or hepatitis C, and individuals with mental health problems (*Vancouver Agreement* Draft Comprehensive Substance Misuse Strategy, 2000).

Creating a continuum of care requires an array of services with many points of contact and entry into treatment in order to deal with different populations of drug users.

Treatment approaches for chronic, street-involved drug users will differ from strategies for those individuals who are in an early phase of substance misuse and may be more motivated to move towards abstinence. Multiple points of contact help to bring a maximum number of drug users into the treatment continuum.

11.4 Treatment, Housing and Social Programs

In Portland, Oregon the connection between treatment resources and housing was a highly successful part of that city's effort to deal with an inner city homeless and primarily alcohol-addicted population in the mid 1970s.

In Vancouver housing has not kept pace with the need to provide stable and supportive housing for people with addiction problems. While much social housing has been built in the past the majority of it has been focused on seniors or those over 45 years of age. Younger persons and those with serious addiction problems often have great difficulty in securing stable, affordable housing. The social housing that has been built usually does not include funding for support services and consequently housing providers are often unable to deal adequately with individuals who may have problems that require an increased staffing level in order to provide a supportive housing environment for them.

In the Downtown Eastside there are approximately 6,000 Single Room Occupancy (SRO) hotel rooms that provide low-income accomodation and 4,000 social housing units (City of Vancouver, 1998). Many of the hotel rooms are unstable and are clearly unsuitable for long term accommodation. The rooms are very small (often 100 square feet) and individuals often live in poorly managed and ageing buildings that do not provide an environment that is conducive to people improving their health status. In fact the health of SRO residents often deteriorates the longer they live under these conditions.

In recent years BC Housing, through the Low Income Urban Singles (LIUS) program has shifted focus from seniors to providing housing for individuals within a broader age range who have lived in the SROs. While this improves housing for an important population funding for health support services to this housing is often lacking. The Vancouver Richmond Health Board provides some services within the limits of their budget but do not receive extra funding to match the increase in units of this kind of housing.

Vancouver has a positive history of providing supportive housing for those

Studies from around the world that have evaluated community-based drug and alcohol treatment programs for homeless people conclude that people need secure and stable housing before treatment can be effective.

Taking Responsibility for Homelessness, an action plan for Toronto, 1998:116.

with mental health problems. Housing developments in the Downtown Eastside such as Triage, the Portland Hotel, the Jim Green Residence and Lookout are examples of supportive and stable housing for those with mental health problems. Clearly future housing projects should focus on those with addiction problems and funding for health support services for these projects should be allocated from the Ministry of Health to the Vancouver Richmond Health Board at the same time as BC Housing allocates more LIUS units.

In 1998, there were only 11 youth detox beds in the Lower Mainland, most of them in Vancouver.

Bognar, Legare and Ross, 1998 and LMMA report, 2000.

The Toronto Homelessness Task Force also concluded that a range of housing options is required for people suffering from substance misuse:

- Wet shelters, in which substance use is tolerated and is not considered a reason to bar or discharge a person;
- Damp houses, in which substance use is tolerated off-site and where on-site support is provided to help the person make the transition to abstinence in a non-threatening way
- Dry houses, in which abstinence is a clear expectation.

The importance of stable housing in the overall framework to deal with substance misuse cannot be overstated. A comprehensive continuum of care that incorporates housing and social programs is being developed through the Vancouver Agreement (Towards a Comprehensive Substance Misuse Strategy, 2000).

11.5 Drug Treatment Courts

An intergovernmental committee is currently working to develop a drug treatment court model for Vancouver. The premise underlying the concept of drug treatment courts is that people who are addicted to heroin and cocaine are not deterred from using drugs by criminal justice sentences.

The addicted drug abuser no more makes a personal choice to be an addict than an obese inactive person chooses to have arteriosclerosis or diabetes. Addicts deserve to be treated the same as people with any other disease and to be given the best available care. However this does not mean that some form of coercion is never appropriate.

John Millar, 1999

Instead, the criminal justice system could be used to move people towards drug treatment. This would have a greater benefit to both the addict and to the community in reducing the harm caused by substance misuse. If an accused is accepted into the drug treatment court their case is moved to a specialized program. "The participant then begins an intensive judicially supervised treatment program that focuses on stability in housing and employment as well as substance misuse" (Summary document, Vancouver Drug Treatment Court, 1999).

Treatment consists of a personalized program that includes methadone maintenance treatment, group therapy, counselling and attendance at self-help groups such as Narcotics Anonymous. A pilot drug treatment court project has been underway in Toronto since December 1998. The Toronto project is specifically designed to help offenders who have a long history of addiction to cocaine and/or heroin and, in most cases, have been in the business of dealing drugs. Commercial traffickers are screened out and are not accepted into treatment. In the Toronto program, offenders plead guilty to the offence and sentencing is postponed until completion or discharge from the program. Upon successful completion, an individual receives a non-custodial sentence and in certain circumstances the charge may be withdrawn.

Drug treatment courts and variations on the concept of diverting drug addicts away from prison sentences and towards drug treatment programs exist in many countries, including the U.S. In Canada, they are a new phenomenon and deserve careful consideration in British Columbia. In the fall of 2000, an evaluation of the Toronto drug treatment court will be complete which may provide insight into the relevance, the cost benefits and efficacy of a drug treatment court model within the Canadian context.

It is important to understand that drug treatment courts do not take the place of other drug treatment programs. They complement other components in the continuum of care and provide yet another opportunity to intervene and assist an individual to move towards improved health. Some have expressed fears that implementing drug treatment courts will jeopardize expansion of the voluntary treatment sector (which is currently inadequately funded) to help those who voluntarily present themselves for drug treatment. This is an important point and must be carefully considered.

Making proven treatments available for injection drug users—with the compassion, respect and care they deserve—will help them recover from or cope with their addiction.

Millar Report, 1998

11.6 Methadone Maintenance Programs

The prescription of methadone to heroin users has become the main treatment for those wishing to reduce their heroin consumption or stop using it altogether. Methadone is a long acting synthetic drug that is prescribed as a legal substitute for heroin and has been used for many years to stabilize heroin users, improve their health and reduce dependence on an illegal substance. Yet it continues to be a subject of debate, despite the fact that research has shown that methadone maintenance significantly reduces harm to individuals and to society—as well as greatly reducing health care costs and crime (Parker, 1997).

Methadone treatment and counselling costs $4,000 per patient per year. The cost of an untreated heroin addict to society is $30,000 per year (Millar Report, 1998:19). Studies show that users want, and need, rapid access to methadone in a non-punitive, non-judgemental environment (LMMA, 2000). Since 1995, there has been a dramatic increase in the number of methadone patients in the province. The number has grown from 1,491 in 1995 to 5,563 in 2000 (CCENDU, 2000). This has been an area of significant growth in the treatment of addiction, which has led to a considerable reduction in harm to communities and individuals. While BC's methadone treatment program is considered to be one of the best in Canada, there is much room for improvement.

Greater access to methadone maintenance treatment can ultimately reduce the incidence of HIV, Hepatitis C and some types of crime.

BC Medical Association, 1998
from LMMA Report 2000.

Even as the drug problem has escalated there continues to be barriers to gaining access to treatment. These include:
• delays in access;
• user and dispensing fees;
• additional fees for counselling;
• too few physicians who are licensed to prescribe;
• restrictive rules and procedures; and,
• lack of integration with other addiction services.

Given the well-documented success of methadone maintenance programs, we must do everything possible to remove these barriers in order to ensure that individuals who can benefit from this type of treatment have the opportunity to do so.

11.7 Other Opiate Replacement Therapies

While methadone maintenance is the primary treatment for heroin addiction, other medications are being used in other jurisdictions. Since methadone does not work for all individuals, it makes good sense to explore other options that will give practitioners and drug users a broader choice of medications.

The BCMA recommends that research projects using other medications be initiated to broaden the range of drugs available for use in treating opiate addiction. Levo-alpha-acetyl-methadol (LAAM) is similar to methadone but is longer acting and can be administered on alternate days or even three times per week (methadone must be administered daily), Buprenorphine is another possible substitute for methadone and is being used in some jurisdictions but is not approved for use in Canada.

Research into these and other experimental drugs should be initiated to explore whether these medications can be added to the treatments available to addicted individuals. Further research on pharmacotherapy for cocaine users is also recommended. Stimulant maintenance involving amphetamines and cocaine has been the subject of some research to date and should also be explored within scientific trials to address the issue of severe cocaine misuse (Alexander, 2000).

11.8 Heroin Assisted Treatment

In Europe, scientific trials exploring the prescription of heroin are currently taking place in Switzerland and the Netherlands. A multi-city, heroin-assisted treatment trial is also being proposed for some cities in Germany. For several decades, the British have used the prescription of heroin for a small number of addicts. The rationale for this therapy is to provide a method of treatment for a highly marginalized street population of heroin users who do not respond to conventional methadone treatment. Results in Switzerland have been promising but concerns regarding the research design have caused other countries such as the Netherlands and Germany to conduct their own scientific trials.

There is currently a proposal for a multi-centre, randomized controlled trial of heroin-assisted therapy for injecting opiate users to take place in several Canadian cities, including Vancouver. Researchers from the BC Centre for Excellence in HIV/AIDS research are involved in the development of this study. If undertaken, the study would target a group of chronic, opiate-dependent injection drug users who have not responded to other forms of treatment. The primary goals of this research are:

- to examine the option of heroin-assisted therapy as a treatment modality for chronic opiate dependence and to develop a scientifically rigorous and ethically defensible study design to address this issue; and

• to determine the political feasibility and acceptability by community and user groups of heroin prescription in select urban areas of North America.

The current proposal for clinical research is in its final stages of development. Canadian research contributions to the growing body of evidence concerning heroin-assisted treatment will provide valuable information and direction for future addictions treatment.

11.9 Needle Exchange Programs

The perception by some that drug use is condoned and even facilitated by needle exchange programs (NEPs) is problematic. The research contradicts this belief. The fact remains that sharing needles among a marginalized population is one of the deadliest activities an individual can engage in—and one of the quickest ways to spread HIV and other deadly blood-borne diseases. It is a public health imperative to do everything possible to prevent the transmission of deadly diseases.

Extensive research has led to a widespread consensus that needle exchange programs not only help prevent diseases such as HIV and hepatitis C, but they also provide a point of contact with health care services for a marginalized population of users, many of whom also have physical and mental health problems. Research has also shown that needle exchange programs work best when they are part of a broad range of services for individuals.

NEPs do not increase drug use; they prevent disease transmission, improve health, and can lead users to further treatment. As a critical component of a continuum of care, needle exchange programs must become part of a broad public health strategy to deal with addiction. Clean injecting equipment should be made available to injection drug users through community health clinics, pharmacies, and through other agencies working with drug users throughout Vancouver. The distribution and exchange of needles must be decentralized so that users have easy access to clean needles closer to where they live.

NEPs are also an excellent way to provide information regarding safer injection techniques, health services, treatment programs, housing, and employment to a large population of drug users.

Policies regarding how needles are given out, methods of return and procedures to recover used needles in the community must be spelled out. There has been considerable debate over how the distribution of needles should occur. Some adhere to the notion that needles should be given out on a one for one basis (for every needle given out the user must present a needle). This way the recovery of used needles is increased as drug users bring back used ones in order to get clean ones. This also ensures maximum contact between drug users and needle exchange staff and other health care workers.

Others argue that the greater public interest in preventing HIV transmission is achieved more effectively by maximizing the distribution of needles to cover the greatest number of injections and developing a range of effective strategies for needle recovery from users in the community. This debate raises important issues for individuals and communities as discarded needles

I wanted to get clean and was trying to get into detox. I called twice a day for days. They kept telling me there was a six month wait. I got tired of it and went back to using. Three days later, guess who calls. By then I was right back to what I had wanted to leave.

(Injection drug user)
Bognar, Legare, Ross, 1998.

Health Canada has concluded that NEPs prevent HIV infection among injection drug users, are not responsible for an increase in the number of drug users, and not responsible for lowering the age at which persons inject drugs for the first time.

Injection Drug Use and HIV/AIDS, 1999:82.

Goal 2.Public Order:
To work towards the restoration of public order across Vancouver by reducing the open drug scene (particularly at Main andHastings), by reducing the negative impact of illicit drugs on our community, by reducing the impact of organized crime on Vancouver communities and individuals, by providing neighbourhoods, organizations and individuals with a place to go (such as a healthy-city office) with their concerns related to safety, criminal activity, drug misuse, and related problems, and by implementing crime prevention techniques to increase public safety.

Goal 3.Public Health:
To work towards addressing the drug-related health crisis in Vancouver by reducing harm to communities and individuals, by increasing public awareness of addiction as a health issue, by reducing the HIV/AIDS/hepatitis C crisis, by reducing overdose deaths, by reducing the number of those who misuse drugs, and by providing a range of services to groups at risk such as youth, women, Aboriginal persons, and the mentally ill.

pose a significant risk to individuals. Clearly achieving the goals of reaching the maximum population of intravenous drug users and of 100 per cent needle recovery is desirable.

11.10 Breaking Down the "Not In My Backyard" (NIMBY) Barriers

The attitude "Not in My Backyard" or "NIMBY" is often an issue when attempting to locate alcohol and drug services of any kind within communities. While there is a great deal of consensus that these services be decentralized throughout Vancouver and the Lower Mainland, there is little commitment from municipalities and neighbourhoods to advocate for them.

There is also little evidence that recovering addicts move to other neighbourhoods to receive treatment. In fact, the reasons they move are generally common to many individuals who do not use drugs: cheaper accommodation, employment opportunities, proximity to family, to get a fresh start. Those who do drift to neighbourhoods such as the Downtown Eastside usually do not do so in order to get treatment (LMMA, 2000). Community opposition to treatment facilities also ignores the fact that in order to recover and reintegrate into society, addicts need community resources and support—in addition to basic social interaction. Municipalities clearly have a significant role in supporting their citizens and advocating services for both those who do not have substance misuse problems and those who do.

11.11 Treatment –Actions

Note: Some actions in the following section may require legislative and/or regulatory changes in order to be implemented. These and others are in italics and are noted in Appendix D.

8. *Increase methadone availability by removing current barriers (such as user fees, counselling fees, and restrictive regulations) for the methadone maintenance program in order to treat an additional 1,000 clients in the Lower Mainland over the next two years, with the Downtown Eastside as a priority area for expansion. Continue the expansion of the Provincial Methadone Maintenance Treatment programs within other areas across Vancouver and the province where there is a highly marginalized group of opiate users and those who use opiates and stimulants in combination.*
   Lead Agency: *Ministry of Health*
   Partner Agencies: *College of Physicians and Surgeons, Ministry for Children and Families, Vancouver/Richmond Health Board*

9. Ensure that a continuum of long-term supportive housing is developed including housing and/or shelter to stabilize those who misuse drugs and alcohol, and drug- and alcohol-free housing for individuals in recovery and that funding from the Ministry of Health for support services to new LIUS projects is tied to new allocations of these units.
   Lead Agency: BC Housing
   Partner Agencies: Ministry of Health, Vancouver/Richmond Health Board, City of Vancouver

10. Establish the 15-bed unit at BC Women's Hospital as planned by the Vancouver Richmond Health Board to include women with children and pregnant women who need detoxification and primary health care services related to substance misuse.
    Lead Agency: Ministry of Health
    Partner Agencies: Vancouver/Richmond Health Board

11. Establish 20 treatment beds for youth outside of the Downtown Eastside in several small, low-community-impact, residential treatment programs that: recognize the role of drug misuse and risk taking in adolescent development; have safety and the long-term well being of youth, rather than abstinence, as the overriding goal; and recognize that abstinence is also an important goal for many.
    Lead Agency: Ministry for Children and Families
    Partner Agency: Vancouver/Richmond Health Board

12. Expand support services to families of children who are involved with substance misuse in order to breakdown stereotypes, help parents deal with feelings of guilt and anger, and help them understand addiction issues such as relapse and the often desperate measures taken by addicted youth.
    Lead Agency: Ministry for Children and Families
    Partner Agency: Vancouver/Richmond Health Board

13. Establish six medical detox beds at St. Paul's Hospital as planned by the Vancouver Richmond Health Boardfor those seeking to withdraw from drugs and/or alcohol and who have serious medical problems.
    Lead Agency: Ministry of Health
    Partner Agency: Vancouver/Richmond Health Board

14. *Take steps to initiate clinical trials of a range of medications (including LAAM and Buprenorphine) for heroin and (amphetamines and cocaine) for cocaine addiction in order to increase the options that doctors have available for treatment for those who are methadone-resistant or who have not responded to treatment options over the long term.*
    Lead Agency: *Health Canada*
    Partner Agencies: *Ministry of Health*

15. *Proceed with the proposed multi-city clinical research trials into the feasibility of heroin-assisted treatment through St. Paul's Hospital and the BC Centre for Excellence in HIV/AIDS Research in Vancouver and other Canadian cities for those who are methadone-resistant or who have not responded to treatment options over the long term.*
    Lead Agency: *Health Canada*
    Partner Agencies: *Ministry of Health*

16. Expand and decentralize needle exchange services across the Vancouver/ Richmond region by providing needle exchange in all primary health care clinics, hospitals, pharmacies and through non-profit groups and user groups.
    Lead Agency: Vancouver/Richmond Health Board
    Partner Agencies: College of Pharmacists

17. Pilot accessible (low threshold) support programs or day centres for addicts in neighbourhoods outside of the Downtown Eastside to help prevent those who use drugs, particularly youth, from becoming more deeply involved in the inner city drug scene.
    Lead Agency: Health Canada
    Partner Agencies: Vancouver/Richmond Health Board, Ministry for Children and Families

18. Commit to creating a range of culturally appropriate strategies and services for Aboriginal persons within the four pillars of prevention, treatment, enforcement and harm reduction with a priority on the development of services for Aboriginal women with addictions and Aboriginal youth at risk.
    Lead Agencies: Vancouver Richmond Health Board, Ministry for Children and Families
    Partner Agencies: Vancouver Police Department

12. Pillar Three – Enforcement

12.1 Defining Enforcement

Enforcement consists of a broad range of activities carried out by regulatory agencies, licensing authorities, police, the courts, and other sectors within the criminal justice system. Coordinated enforcement is a key pillar in any drug strategy. Police have a difficult and critical role to undertake in minimizing the harm caused by substance misuse.

In order to uphold public safety and create a climate of social responsibility, the laws against the sale of illicit substances, associated crimes, and the misuse of alcohol must be enforced. This *Framework for Action* discussion paper attempts to clarify that the four-pillar approach deals with people who have an addiction and need treatment, while clearly stating public disorder, including the open drug scene, must be stopped. In short, addiction needs treatment and criminal behaviour needs enforcement.

And if enforcement is to be effective, individuals and businesses involved in the illegal drug trade or facilitating the misuse of alcohol must be dealt with expeditiously.

Police also have a major role to play in assisting communities minimize the negative impacts of drug dealing and misuse by working with community organizations and existing crime prevention groups to address community health and safety. Police and other enforcement agencies cannot stop drug dealing— but they can limit it significantly. In many cases enforcement efforts result in improved conditions in neighbourhoods and an increased ability to coordinate efforts with other disciplines, jurisdictions, and with members of the community.

12.2 Problems Surrounding Enforcement

Too often enforcement efforts fail to achieve a lasting change as problems quickly reappear or are displaced to nearby streets. The visible drug scene in the Downtown Eastside is one example. Another is the hundreds of marijuana grow operations that seem to spring up again as quickly as they are discovered and closed down. Yet another example is the number of drug houses that disrupt neighbourhoods for months or years before being forced to close down.

Reasons why enforcement efforts often fail to achieve significant results include:

- Police have few available tools to maximize the impact of enforcement efforts, such as options to divert those arrested to a range of support or treatment programs.
- The sheer scale of the drug market, and the sophistication and mobility of criminal organizations that import and market illegal drugs is overwhelming.
- Current laws require cumbersome processes for the gathering of evidence for trials.
- There is a severe backlog of criminal cases in the courts.

Traffickers are being charged and held for court. They are subsequently released the next day with court orders. They are then quickly encountered again trafficking on the streets.
In talking to these people, the courts are merely a hindrance and referred to as a 'cost of doing business.' These people know that it will not cost them anything, as a lawyer is provided through legal aid and it is very unlikely that they will spend any time in jail.

(Police officer), Cain report, 1994:68

- Judges are reluctant to treat addiction entirely as a criminal matter because the problem will not be solved by simply sending addicts to jail and prison.

Vancouver Police support the expanded health services announced under the *Vancouver Agreement* in September 2000 because they provide police with a range of tools that will help them steer those with addiction problems towards a healthier life.

But these tools are still not enough to help police deal effectively with the growing drug crisis. They also need more tools within the criminal justice system.

Some of these tools might include:

- The development of community courts to allow immediate prosecution of minor offences and divert many of those arrested into community service where they would have access to counselling, drug treatment programs and other social supports.
- The establishment of night courts to ensure 24-hour access to judges and crown prosecutors and result in more timely trials. The current backlog of cases means that the time between an arrest and a trial can be as long as ten months.
- The creation of drug treatment courts to give those who have committed minor drug-related offences the option of going to treatment instead of to prison.
- The increased use of community impact statements in court to allow the judges to hear from those in the community who experience the negative impact of substance misuse on a daily basis.
- Increased cooperation between immigration officials and police.
- Changes in laws and City by-laws to give police more power to enforce against drug dealing and public consumption of drugs.

From the point of view of arrests, incarceration, release [we] just haven't made a dent. And for a number of reasons. For starters, there is a huge glut of drugs on the market, so there is a constant steady supply.

Larry Campbell, former Chief Coroner, Province of British Columbia

The criminal justice system, like the health care system and addiction services, has not kept up to the changes in scale and structure of the illicit drug trade, or to its negative impact on our communities. The increasing sophistication of organized crime may require new and more innovative methods of policing and enforcement.

12.3 The Scale of the Illicit Drug Market

The drug market in the Vancouver region is huge and complex. The problem that its sheer scale presents for those involved in enforcement is equally onerous. There are simply too many individuals and organizations in the business of selling illicit drugs for enforcement personnel to respond to. In addition, individuals on the street who actually sell illicit drugs are often no more than carriers for drug traffickers who do not touch the drugs but reap most of the profits.

More troubling is the fact that while most anger and outrage is directed at highly visible street level dealers, the actual importers of large quantities of

heroin and cocaine are often very sophisticated in avoiding the law, have good international connections, and can hire the expertise to ensure that legal actions against them are difficult to carry out. Drug traffickers take great care to erase any trace or connection between themselves and shipments of drugs from Asia or South America. These people are masters of manipulation and impersonation, and can blend into mainstream society quite easily. They employ a cadre of personnel to handle their shipments, and to distribute the drugs to the various markets locally and throughout the country.

There are an estimated 12,000 injection drug users in the Lower Mainland. An estimated 8,000 of those live in Vancouver (BC Centre for Excellence in HIV/AIDS, 1999). The market for illicit drugs in the Lower Mainland is significant and it supports a thriving industry. In the Downtown Eastside alone the size of the market for heroin and cocaine is huge. For example, if 100 drug dealers each bring in an average of $3,000 in a 24-hour period, this adds up to approximately $110 million per year in this small neighbourhood alone.

Combined with the approximately 7,000 licensed bar seats in the area the scale of the market in drugs and alcohol is overwhelming. The impact on the Downtown Eastside and surrounding communities is devastating.

The market in drugs and alcohol in the Downtown Eastside community draws people from the entire region and overwhelms a neighbourhood with a rich historic fabric and a diverse mixture of people. And this is just the tip of the iceberg. The Downtown Eastside market is a small part of the regional market that is much less visible and takes a variety of forms, such as drug houses, business operations, and delivery services commonly referred to as "dial-a-dope." The capacity of community policing initiatives to significantly disrupt the illegal drug market is limited and must be strategically targeted at local public order issues and specific neighbourhood safety concerns in an effort to minimize the negative impact of the drug trade.

Highlights from the Royal Canadian Mounted Police Criminal Intelligence Directorate include the following information:

- At least 100 tonnes of hashish, 15 tonnes of cocaine, and six tonnes of liquid hashish are smuggled into Canada each year. Production of marijuana is estimated at 800 tonnes. One to two tonnes of heroin are required annually to meet the demand by the Canadian heroin user population.
- Drug trafficking remains the principal source of revenue for most organized crime groups. In Canada, the drug trade has the potential to generate criminal proceeds in excess of $4 billion at the wholesale level, and of $18 billion at the street level.
- Italian-based organized crime is involved in upper echelon importation and distribution of many types of drugs. Asian-based groups are active in heroin, and increasingly in cocaine trafficking at all levels. Colombian-based traffickers still control much of the cocaine trade in the cities of Eastern and Central Canada. Outlaw motorcycle gangs play a major role in the importation and large-scale distribution of cannabis, cocaine and chemical drugs.

The courts should distinguish between users and pushers …

They have made enforcement virtually impossible.

Cain report, 1994.

Independent Canadian and foreign entrepreneurs are also important suppliers of drugs to the Canadian market.

Clearly, street level enforcement efforts have severe limitations given the scale of the problem. Expectations that extra officers at the street level can significantly alter a problem of this scale and complexity are unrealistic. Many other interventions are required at higher levels of the illicit drug market to reduce the supply of drugs. And comprehensive health initiatives are needed to begin to reduce the demand for illicit drugs in the city.

As police officers, part of our oath of office is to protect life. In the drugs field that policy must include saving life as well as enforcing the law. Clearly, we must reach injectors and get them the help they require, but in the meantime we must try and keep them healthy, for we are their police as well.

D. O'Connell, The role of the police in Merseyside. Presentation at the first International Conference on the Reduction of Drug Related Harm, Liverpool, April 1990

Police and other enforcement personnel are often the first line of contact for addicted individuals. They can provide a critical link between street-level drug users and a range of health and social support services, some within the criminal justice system in the form of diversion programs or drug courts. Police officers often have daily contact with individuals who misuse drugs and can make interventions that not only benefit the community but also move an individual drug user towards health services.

Enforcement strategies involve a broad range of agencies and often require the coordination of efforts across disciplines. The success of a four-pillar strategy requires the complex collaboration of various agencies including the police, probation services, the courts, youth services, emergency health services, corrections, liquor licensing branches, and business license authorities.

12.4 The Issue of Displacement

There has been a history of displacement of street level illicit drug dealing and prostitution in Vancouver. Over the years these activities have simply been shifted to different areas of the city. The movement of prostitution out of the West End and the increased enforcement actions on Granville Street during Expo '86 are examples of displacement that had negative impacts on the Downtown Eastside. Many argue that this displacement has seriously enlarged the drug market in the Downtown Eastside, leaving that neighbourhood to deal with the negative consequences. In fact, increased suppression of drug-related activity can lead to geographic displacement of drug markets and, perhaps more seriously, to other forms of drug dealing.

12.5 Dial-a-Dope

Recently, there has been a critical change in the way that drugs are sold in Vancouver. Both people on the street and police officers have said that by increasing enforcement in the Downtown Eastside we have unwittingly not only displaced drug dealing but have forced it to take other forms. One of the most intractable forms of drug dealing that has recently escalated in scale is the phenomenon of "dial-a-dope." This form of drug dealing is invisible, has no impact on the street drug markets, and is particularly convenient for youth and others who fear the risk of being seen buying drugs. It is also extremely difficult and expensive to enforce against.

12.6 Money Laundering

The increased sophistication of the drug marketing organizations at the global level means that international cooperation in responding to drug marketing and organized crime is more critical than ever. One of the main

Commenting on "Dial-a-dope,"

targets for enforcement authorities attempting to break up criminal organizations are the enormous sums of money that flow across borders from the profits made from the sale of illicit drugs.

The ability to launder large sums of illegally earned money is the life-blood of organized crime. Funds that show up in bank accounts around the world are difficult if not impossible to trace back to their illicit origins. It is estimated that between $5 billion and $17 billion is moved within and through Canada each year by criminal organizations. Preventing money laundering is difficult, but there are international efforts underway to try to curtail it.

New legislation on the Proceeds of Crime (Money Laundering) is being developed by the federal government. The new legislation has three main components:

- The mandatory reporting of suspicious transactions, which requires a range of regulated businesses including financial institutions, casinos, currency exchange businesses etc., "to report any financial transactions that they have reasonable grounds to suspect are related to a money laundering offence."
- The required reporting to Canada Customs of large cross-border movements of currency or monetary instruments across the Canadian border.
- The creation of a new Financial Transactions and Reports Analysis Centre of Canada which will serve as a central repository for information about money laundering activities across Canada.

This legislation will assist the police in targeting higher-level criminal activities related to the drug trade and reducing its profits to organized crime.

12.7 Decriminalization, Legalization, Prohibition

Enforcement efforts can and do make a difference in the quality of life in Vancouver neighbourhoods. They do this by addressing persistent neighbourhood problems and by increasing the ability of communities to respond to substance misuse issues. However, the capacity of police and other enforcement agencies to significantly alter the structure and volume of the regional drug trade is a matter of considerable debate. Some argue that enforcement of the laws against possession of small quantities of illicit drugs is actually counterproductive to restoring public order and to deterring the sale and use of illicit drugs.

The prosecution of addicted drug users who deal drugs to support their habit usually involves relatively small quantities of illicit drugs and is increasingly ineffective and costly. Individual users who have their drugs confiscated by police must find more money to replace their drugs. This, in turn, can lead to increased instability for the drug user and the community, increased crime, prostitution, and other forms of harm and public disorder.

For the drug dealers, police enforcement does disrupt the market place and has a variety of consequences. Some dealers are arrested and detained for varying periods of time. Others who have not been arrested and are still "in business" find that they have a whole new group of customers aside from their "regulars," as people start looking for new sources of drugs. In this situation the price of drugs usually increases for two reasons.

The first is that the risk to the dealer is greater, because the new customers are unknown and might include undercover police. The second reason is that after a group of dealers are arrested users have fewer choices and prices can be raised for the duration of the market disruption. Again, with higher prices more funds must be found to purchase drugs.

Currently there is much debate about legalization of illicit drugs, particularly marijuana. The recent Supreme Court of Canada ruling of July 31, 2000, confirmed an earlier trial court decision granting a stay of proceedings brought against Terrance Parker for cultivating cannabis for medicinal uses contrary to the Narcotic Control Act, and for possession of cannabis contrary to the Controlled Drugs and Substances Act. This sparked much dispute on drug policy in this country. Many argue for separating marijuana from hard and addictive drugs.

However, many individuals also support increasing enforcement in an attempt to make prohibition work. They argue that prohibition of illicit drugs is critical in limiting access to dangerous substances. Among other things, the price of illicit drugs is higher than it would be if they were legal. This, they argue, deters young people from becoming involved in drug use. Prohibition of drugs such as heroin, cocaine and cannabis sends a strong message to society that these drugs are dangerous and that criminal prosecution will result if one is caught selling or using them.

However, the reality for youth is that street drugs are easy to obtain and the strength and purity are unknown, whereas substances such as alcohol are regulated and relatively difficult to procure for teenagers.

Increasing efforts to prohibit the sale and use of drugs would require stronger penalties for engaging in the drug trade. At the very minimum, this would mean incarceration of more people for longer periods of time. Drug dealers would have to receive longer sentences and drug users would be subject to a wider range of punitive options and coercive efforts to move them into drug treatment programs.

12.8 Enforcement Summary

Despite the ongoing debates over the merits or pitfalls of decriminalization or legalization versus the continued prohibition of illicit drugs, there are very serious problems in local communities related to substance misuse that must be addressed now. Many addicted individuals are in despair and some communities are bearing more than their share of the negative impacts of the illegal drug trade. Police have been very clear in recent years that there are limits to what they can do without the support of complementary health services oriented toward getting people off the street and into a continuum of programs that deal with substance misuse.

The recent shift toward community policing strategies by the Vancouver Police Department holds a promise of more collaboration between police, community groups and the various levels of government in responding to substance misuse issues in local communities. Enforcement efforts at the higher levels of the drug trade must compete with the tactics of sophisticated and well-financed criminal organizations. Innovation and changes in legisla-

tion to give enforcement agencies more authority in certain spheres may be necessary in order to stifle organized crime. New legislation on money laundering is an example of this kind of legislative change that supports enforcement efforts to reduce the harm associated with the illegal drug market.

12.9 Enforcement –Actions

Note: Some actions in the following section may require legislative and/or regulatory changes in order to be implemented. These and others are in italics and are noted in Appendix D.

19. Increase the Organized Crime Unit, the Vancouver Police Drug Squad and the RCMP Drug Squad unit in order to better target organized crime, drug houses that cause neighbourhood disruption and mid and upper level drug dealers that supply street level drug dealers.
    Lead Agencies: Solicitor General (Federal), Office of the Attorney General (Provincial), City of Vancouver
    Partner Agencies: Vancouver Police Department

20. Institute a senior-level Drug Action Team comprised of senior staff from: Vancouver Police, City of Vancouver, Vancouver/Richmond Health Board, the Attorney General's office, Ministry for Children and Families, the RCMP and community representatives. In coordination with local Neighbourhood Integrated Service Teams, local Community Health Committees, service agencies and Community Policing organizations, this group will coordinate responses to serious drug-related issues raised by neighbourhoods.
    Lead Agency: City of Vancouver
    Partner Agencies: Vancouver Police Department, Vancouver/Richmond Health Board, Office of the Attorney General (Provincial), Ministry for Children and Families, RCMP

21. Initiate a pilot Drug Treatment Court in Vancouver and advocate for creating a range of diversion programs within the criminal justice system that give individuals the option of entering treatment and support programs instead of going to trial and prison. Also explore community courts and options related to community service.
    Lead Agency: Office of the Attorney General (Provincial)
    Partner Agencies: Department of Justice Canada, Ministry of Health (Provincial)

22. *Review existing Federal and Provincial laws and City by-laws to determine what changes are needed to give police and the courts better tools to respond to changes in the illegal drug trade such as "dial a dope" operations, public consumption of drugs, and the sexual exploitation of youth.*
    Lead Agencies: *Solicitor General (Federal), Office of the Attorney General (Provincial), City of Vancouver*
    Partner Agencies: *Department of Justice Canada, Ministry for Children and Families*

Goal 2.Public Order:
To work towards the restoration of public order across Vancouver by reducing the open drug scene (particularly at Main and Hastings), by reducing the negative impact of illicit drugs on our community, by reducing the impact of organized crime on Vancouver communities and individuals, by providing neighbourhoods, organizations and individuals with a place to go (such as a healthy-city office) with their concerns related to safety, criminal activity, drug misuse, and related problems, and by implementing crime prevention techniques to increase public safety.

Goal 3.Public Health:
To work towards addressing the drug-related health crisis in Vancouver by reducing harm to communities and individuals, by increasing public awareness of addiction as a health issue, by reducing the HIV/AIDS/hepatitis C crisis, by reducing overdose deaths, by reducing the number of those who misuse drugs, and by providing a range of services to groups at risk such as youth, women, Aboriginal persons, and the mentally ill.

23. Continue the redeployment of police officers in the Downtown Eastside to increase contact and visibility in the community and improve police coordination with health services and other agencies to link drug and alcohol users to available programs.
    Lead Agency: Vancouver Police Department
    This initiative is also part of the Vancouver Agreement initiatives announced September 29, 2000 and cross-referenced in Appendix B.

24. *Explore legal and policy options related to the provision of mandatory treatment for a small group of repeat criminal offenders who are addicted to heroin, cocaine, or alcohol and responsible for a high percentage of crimes committed in the city.*
    Lead Agency: *Office of the Attorney General*
    Partner Agency: *Ministry of Health*

25. *Explore legal and policy options to allow for mandatory drug treatment for youth involved in the illegal drug trade and severely addicted youth who are at risk of harming themselves and others as a result of their addiction.*
    Lead Agency: *Ministry for Children and Families*
    Partner Agency: *Office of the Attorney General*

13. Pillar Four – Harm Reduction

13.1 Defining Harm Reduction

If we are going to implement a successful drug strategy in Vancouver, we must acknowledge the need for harm reduction programs and realize that accepting harm reduction as part of the strategy does not mean condoning the use of illicit drugs. It means accepting the fact that drug use does and will occur—and accepting the need to minimize the harm this has on communities and individuals. And it means recognizing that abstinence-based strategies are often impractical and ineffective in dealing with the street-entrenched drug scene.

One of the major challenges we face in designing and implementing a coordinated, comprehensive drug policy is establishing a common definition of harm reduction— one that is accepted and endorsed by government agencies, health care providers, law enforcement, working groups, drug users, and the community at large. Harm reduction is a key component of both national and provincial drug strategies. Canada's Drug Strategy and BC's Ministry for Children and Families, Addiction Services Program Guidelines define "harms" as follows:

- Physical harms include death, illness, addiction, the spread of disease such as HIV/AIDS and hepatitis, and injury caused by drug-related accidents and violence.
- Psychological harm can include fear of crime and violence and the effects of family breakdown.
- Societal harm refers to breakdown of social systems.
- Economic harm includes the large-scale impact of the illegal drug trade and enforcement efforts as well as economic harm to individual users and society, including costs of decreased and lost productivity, workplace accidents, health care harms, and business and neighbourhood economic development.
- Harms to the individual may be physical, psychological, spiritual, social, and economic.

The goals of harm reduction in *A Framework for Action* are twofold: to reduce harm to the community, and to reduce harm to the individual. This includes harms resulting from public nuisance, disorder, and the debris of the drug scene such as litter, discarded needles and other paraphernalia. The Federal/Provincial Harm Reduction Working Group set out five guiding principles of harm reduction:

- First, do no harm.
- Respect the basic human dignity of persons who use drugs.
- Maximize intervention options.
- Focus on the harms caused by drug use, rather than drug use per se.
- Choose appropriate outcome goals.

The overriding goal must be to minimize risk to the individual, the community, and society as a whole through providing care and support to our most vulnerable citizens.

National Action Plan, 1997:17

A Framework for Action recognizes that:

- Harm reduction is a pragmatic approach with the overall goal of reducing harms to communities and individuals.
- Harm reduction involves establishing a hierarchy of achievable goals, which taken one at a time, step by step, can lead to a fuller, healthier life for drug users, and a safer, healthier community for everyone.
- Harm reduction recognizes that abstinence may not be a realistic or desirable goal for certain users, particularly in the short term.
- Harm reduction must include a law enforcement strategy to move addicts off the street, out of back alleys and into health services.

Discussions on the merits of harm reduction have been ongoing for several years. Many major stakeholders, including the Vancouver/Richmond Health Board, Health Canada, Chief Coroner's Office of BC, the Lower Mainland Working Group on Communicable Diseases, and other government and community organizations
consider harm reduction programs to be essential elements in the continuum of treatment services.

13.2 Coming to Terms with Harm Reduction

The overriding goal must be to minimize risk to the individual, the community, and society as a whole through providing care and support to our most vulnerable citizens.

National Action Plan, 1997:17

Most stakeholders agree that reducing drug-related harm is a good thing, but there is disagreement as to how this should be accomplished. Some people think that abstinence-based approaches are the only way to reduce harms. Others believe enforcement is the key. However, abstinence is an unrealistic goal for many chronic drug users and can lead to greater harm because individuals may consistently fail to achieve the goal of abstinence and fall out of programs. This only increases a person's experience of failure and can lead to even more destructive behaviours. Many drug users are not ready or willing to enter treatment programs that demand abstinence from drugs. Others are more vulnerable to overdose, depression and suicide immediately after coming out of a period of abstinence.

Since much drug use is for a short period of time, harm reduction programs help individuals stay healthier while reducing the spread of HIV, hepatitis C and other infectious diseases. It has also been shown that those who use small amounts of heroin or cocaine, or who participate in methadone programs, cause relatively little harm to themselves or to society.

The smaller percentage of serious, street entrenched drug users remain outside the system. They are usually homeless or living in substandard housing, are socially isolated, and are affected with a range of physical and mental health problems. This group is the main target for front-line harm reduction services. The reason is simple. The more desperate they become with their addiction, the more likely they are to harm themselves or the community.

The primary purpose of low threshold harm reduction programs is to continually build relationships with those on the margins of the health care system. In Vancouver, some say that the traditional treatments for heroin

addiction will not work for many street drug users whose drug of choice is cocaine. As there are no standard treatments for cocaine use, harm reduction focuses instead on building relationships with this highly marginalized population. The continuity and deepening of these relationships over time is crucial to being able to help these individuals the moment they are ready and able to receive it.

For long-time or chronic drug users, harm reduction programs must include measures to help addicts stay alive, prevent the transmission of disease, encourage safe consumption practices that minimize the impact on the surrounding community, and to reduce public disorder and consumption of drugs on the street. Programs that reduce the risks of illness, death and transmission of communicable diseases protect not only the drug users, but also the entire community.

The ultimate criterion of success is not the achievement of an idealized state (such as abstinence) but the net impact on harm indicators.

A Drug Strategy For British Columbia, 2000

13.3 Low Threshold Support Programs or Day Centres

The term "low threshold" refers to programs where abstinence is not a requirement for admittance. Low threshold support programs have been a key component in successful European drug strategies. In combination with a broad range of services, these programs operate out of a variety of facilities and provide a place for people to get off the street, get away from the drug scene, and engage with others in positive activities. These programs provide a supportive environment for individuals who need some respite from the street or are contemplating moving towards detox and/or treatment.

Support programs can be found in a variety of forms, including employment and skills training, contact cafes, day centres, and art and poetry workshops. These programs provide the necessary linkages with other treatment programs, social services and employment programs and housing. Often basic services such as food, showers, telephones, and laundry are provided.

Addicts spend a great deal of time procuring drugs, consuming drugs and raising funds for more drugs. When drug consumption is reduced or ceased individuals are left with a great deal of time on their hands. This is often one of the most difficult aspects of moving away from the drug scene. Low threshold support programs provide a place to go where a person can participate in a range of activities in a supportive environment. The structure and stability that these programs provide is critical for individuals attempting to rebuild their lives and re-integrate into society. The proposed Resource Centre in the Downtown Eastside is modeled after these programs.

13.4 Safe Injection Rooms or Consumption Rooms

Some stakeholders have proposed safe injection rooms, or consumption rooms, as a partial solution to the negative public health effects and public disorder caused by the open drug scene in the Downtown Eastside. These legally sanctioned facilities could provide a safe, secure environment where drug users could inject away from the dirt and dangers of the street—and in contact with health professionals trained in safe injection techniques and overdose response. Opportunities for referrals to further treatment and support programs would be maximized. The call for a trial of safe injection rooms is a result of several factors:

- the price of heroin and cocaine has decreased while the purity has increased, escalating the risk of overdose and death;
- the continuing epidemic of fatal and non-fatal drug overdoses in Vancouver;
- the persistent high rate of HIV and hepatitis C infection;
- public injection and consumption of drugs in the Downtown Eastside;
- the high cost of medical intervention and emergency response to drug overdoses and other serious medical conditions brought on by unsafe injection practices.
- compelling evidence from the European experience that safe consumption sites reduce both health risks and risks to the community of substance misuse.

Safe injection rooms or consumption rooms reduce drug-related harm in several ways:

- Supervised injection rooms reduce deaths by overdose.
- They provide a safe, clean and secure place for users to inject while reducing the visibility of drug consumption on the street.
- They provide an opportunity for multiple contacts with health care staff, social workers, and other individuals who can help users move toward healthier choices, such as drug treatment programs, primary health care and other social services.
- The provision of clean equipment and the disposal of used equipment reduces HIV and hepatitis C transmission, risks to the community from discarded needles and thus reduce health care costs in the long term.
- Police have a place to direct street users to and therefore be able to more easily separate drug users from non-addicted dealers.
- Risks to the community are reduced as the open consumption of drugs can be more easily discouraged.

Two key guiding principles have emerged from the European experience with safe injection rooms. The first is community involvement in the form of education, information sharing, consultation and debate during the early stages of policy making. The second is the importance of ensuring that the design, location, rules and regulations of safe injection rooms are appropriate for the needs of the target group (Not Just for Us, 2000). Developing safe injection sites according to these principles will ensure that the needs and concerns of the community are considered and that drug users in the area will utilize the facilities.

Concerns have been expressed about the potential for safe injection rooms or consumption rooms to attract drug users from other municipalities and even other regions of Canada. Evidence from Europe and surveys of street drug addicts in Australia indicate that addicts will travel only a short distance between the point of purchase and the use of drugs (Clarke, 2000; Not Just for Us, 2000). Consequently in order to be effective safe injection facilities or consumption rooms must be in close proximity to existing local street drug markets.

As the Vancouver/Richmond Health Board begins to implement a comprehensive drug and alcohol program, the current debate over whether safe injection rooms can play a role within the Vancouver context deserves careful consideration. A stringently-controlled clinical trial site or sites would allow authorities and members of the community to evaluate the efficiency of such an approach in reaching the goals of improved public health and a reduction of street disorder.

Note: *Placeholder for information about regulatory or legislative issues concerning safe injection sites.*

13.5 Harm Reduction –Actions

Note: Some actions in the following section may require legislative and/or regulatory changes in order to be implemented. These and others are in italics and are noted in Appendix D.

26. Provide short-term shelter and housing options for active drug users currently living on the street.
    Lead Agency: BC Housing
    Partner Agencies: City of Vancouver, Vancouver/Richmond Health Board

27. *Establish a multi-sectoral task force with representation from all levels of government to consider the feasibility of a scientific medical project to develop safe consumption facilities in Vancouver and in other appropriate areas in the region and across the country in order to reduce health risks and minimize open drug scenes.*
    Lead Agency: *Health Canada*
    Partner Agencies: *Vancouver/Richmond Health Board, City of Vancouver, Vancouver Police Department, RCMP, Attorney General*

28. Implement an overdose death prevention campaign that involves the Vancouver/Richmond Health Board, Vancouver Police, BC Ambulance Service, City of Vancouver, drug user organizations and community agencies to develop overdose prevention strategies.
    Lead Agency: Vancouver/Richmond Health Board
    Partner Agencies: Vancouver Police Department, BC Ambulance Service, City of Vancouver

29. Establish testing procedures for street drugs that will give the community quick access to information about the changes in purity and quality of street drugs in order to prevent drug overdose and deaths. Develop official policy to support efforts to test drugs for content and purity at Rave parties.
    Lead Agency: Vancouver Police Department
    Partner Agencies: RCMP, B.C. Coroners Office, City of Vancouver, Vancouver/Richmond Health Board

30. Advocate for appropriate housing for those with mental illness and dual-diagnosis problems throughout the region.
    Lead Agency: Vancouver/Richmond Health Board
    Partner Agencies: BC Housing, City of Vancouver, Ministry of Health

Goal 2.Public Order:
To work towards the restoration of public order across Vancouver by reducing the open drug scene (particularly at Main and Hastings), by reducing the negative impact of illicit drugs on our community, by reducing the impact of organized crime on Vancouver communities and individuals, by providing neighbourhoods, organizations and individuals with a place to go (such as a healthy-city office) with their concerns related to safety, criminal activity, drug misuse, and related problems, and by implementing crime prevention techniques to increase public safety.

Goal 3.Public Health:
To work towards addressing the drug-related health crisis in Vancouver by reducing harm to communities and individuals, by increasing public awareness of addiction as a health issue, by reducing the HIV/AIDS/hepatitis C crisis, by reducing overdose deaths, by reducing the number of those who misuse drugs, and by providing a range of services to groups at risk such as youth, women, Aboriginal persons, and the mentally ill.

14. Coordination, Monitoring and Evaluation Process

Goal 4. Coordinate, Monitor and Evaluate: To advocate for the establishment of a single, accountable agent to coordinate implementation of the actions in this framework, and to monitor and evaluate implementation through senior representatives of the Vancouver/Richmond Health Board, the Vancouver Police Department, the City of Vancouver,the BC Centre for Disease Control, Ministry of Children and Families, the Office of the Attorney General, and community representatives.

14.1 Action

31. Oversee balanced implementation of the four-pillar approach; prevention, treatment, enforcement and harm reduction.

15. Conclusion—A Call-to-Action

We are all aware of the drug crisis in Vancouver. There have been hundreds of discussions, meetings, and forums since the former Chief Coroner of British Columbia, Vince Cain, called for urgent action in 1994 as a result of the epidemic of overdose among intravenous drug users. Even during this period of research, public debate and education, the crises of drug use, overdose, HIV, and hepatitis C continue across Vancouver and in other parts of British Columbia.

A Framework for Action is an urgent appeal to all levels of government, the many committed non-government agencies, our law enforcement agencies, our criminal justice system, and health care professionals to rally together to develop and implement a coordinated, comprehensive framework for action for the City of Vancouver - one that balances public order and public health and is based on the four pillars of prevention, treatment, enforcement and harm reduction. The actions outlined in this document must be taken as part of a coordinated health and enforcement strategy to create a safer, healthier community.

We must implement a city-wide strategy to curb the negative impacts of substance misuse on communities and individuals. We must work together to eliminate the open drug scene in the Downtown Eastside. Community members must be assured that enforcement efforts and the implementation of crisis health services will not lead to an increase in neighbourhood problems, but will in fact restore public order and lead to an increase in public health.

Vancouver's image as a safe, civilized city is marred by the harm that communities, individuals and businesses are experiencing as a result of the drug crisis—and our inaction in dealing with it. We need to coordinate our efforts and secure the resources needed to initiate a comprehensive program that will make Vancouver a safer, healthier community for everyone. And since our problem mirrors what many cities across Canada are faced with, *A Framework for Action* could become a template for consideration by provincial and federal governments as a framework for national policy concerning urban illegal drug use.

The recent commitment of $13.9 million in municipal, provincial and federal funds as the initial phase of the Vancouver Agreement's five-year health, social and economic initiative for the Downtown Eastside is an important first step. The City of Vancouver is committed to facilitating cooperation and support from all levels of government in order to ensure our community receives the resources needed to implement many of the actions within *A Framework for Action*.

A Framework for Action is a draft discussion paper intended to summarize the issues, invite community participation, review and comment, and catalyze action. Only by moving forward with each of the four pillars in a balanced way will we be successful in managing the drug crisis in Vancouver.

The arguments to act now to halt the epidemics of HIV and injection drug use are compelling. The longer the wait for action, the larger the toll, be it human misery and death, deterioration of neighbourhoods, waste of avoidable tax dollar expenditure, or the further deterioration of a society which does not care.

Bognar, Legare, Ross, 1998.

Appendix A
Goals and Actions - Summary

Goal 1

Provincial and Federal Responsibility: To persuade other levels of government to take action and responsibility for elements of the framework within their jurisdiction by encouraging a regional approach to the development of services, and by demonstrating the city-wide, regional, national and international implications of the drug problems in Vancouver. This goal is the overarching goal and the key element to achieving the following four goals.

Actions:

Regional and National Drug Strategy

1. The **Provincial ministries responsible** implement policy that ensures municipalities throughout British Columbia support the development of a full range of drug and alcohol services.

2. The **Provincial Government** implement a policy framework for reducing the harms to the community and individuals associated with alcohol, tobacco and illicit drugs to guide and inform municipal decision makers in determining priorities for action.

3. The **Federal Government** take strong leadership in the following areas:

Note: Some actions in the following section may require legislative and/or regulatory changes in order to be implemented. These and others are in italics and are noted in Appendix D.

· *Review existing laws with regard to illicit drugs, organized crime, gathering of evidence in drug cases and protection of youth.*

· Implement new money laundering legislation.

· *Review existing laws and procedures to deal with refugees claimants who are engaged in the illegal drug trade.*

· *Initiate research and development of alternative pharmacotherapies for drug addiction including: Levo-alpha-acetyl-methadol (or, LAAM, a derivative of methadone that is long-acting), Buprenorphine (an alternate therapy for heroin users), Amphetamines and other drugs to treat cocaine addiction.*

· *Provide leadership in the development of national research into the feasibility of such initiatives as: heroin-assisted treatment, safe injection or consumption rooms, low threshold methadone prescribing practices and other innovative approaches to addiction treatment and the reduction of drug-related harms to individuals and communities.*

Goal 2

Public Order: To work towards the restoration of public order across Vancouver by reducing the open drug scene (particularly at Main and Hastings), by reducing the negative impact of illicit drugs on our community, by reducing the impact of organized crime on Vancouver communities and individuals, by providing neighbourhoods, organizations and individuals with a place to go with their concerns related to safety (such as a *healthy-city* office), criminal activity, drug misuse, and related problems, and by implementing crime prevention techniques to increase public safety.

Actions:

Note: Some actions in the following section may require legislative and/or regulatory changes in order to be implemented. These and others are in italics and are noted in Appendix D.

Prevention

6. *Explore options for distributing BC Benefit cheques throughout the month in order to reduce harm to individuals and the community by decreasing the sale and use of drugs and alcohol at one time by those on BC Benefits who suffer from addiction and mental health problems.*
   Lead Agency: *Ministry of Social Development and Economic Security*

7. Consider the creation of a Healthy City Office within the City of Vancouver in order to support a coordinated response to community health and safety and crime prevention in the city and to promote and support projects that work towards creating healthier and safer neighbourhoods within Vancouver.
   Lead Agency: City of Vancouver
   Partner Agencies: Vancouver/Richmond Health Board, Ministry for Children and Families, Vancouver Police Department.

Treatment

8. *Increase methadone availability by removing current barriers (such as user fees, counselling fees, and restrictive regulations) for the methadone maintenance program in order to treat an additional 1,000 clients in the Lower Mainland over the next two years, with the Downtown Eastside as a priority area for expansion. Continue the expansion of the Provincial Methadone Maintenance Treatment programs within other areas across Vancouver and the province where there is a highly marginalized group of opiate users and those who use opiates and stimulants in combination.*
   Lead Agency: *Ministry of Health*
   Partner Agencies: *College of Physicians and Surgeons, Ministry for Children and Families, Vancouver/Richmond Health Board*

9. Ensure that a continuum of long-term supportive housing is developed including housing and/or shelter to stabilize those who misuse drugs and alcohol, and drug- and alcohol-free housing for individuals in recovery.
   Lead Agency: BC Housing
   Partner Agencies: Vancouver/Richmond Health Board, City of Vancouver

Enforcement

19. Increase the Organized Crime Unit, the Vancouver Police Drug Squad and the RCMP Drug Squad unit in order to better target organized crime, drug houses that cause neighbourhood disruption and mid and upper level drug dealers that supply street level drug dealers.
Lead Agencies: Solicitor General (Federal), Office of the Attorney General (Provincial), City of Vancouver}
Partner Agencies: Vancouver Police Department

20. Institute a senior-level Drug Action Team comprised of senior staff from: Vancouver Police, City of Vancouver, Vancouver/Richmond Health Board, the Attorney General's office, Ministry for Children and Families, the RCMP and
community representatives. In coordination with local Neighbourhood Integrated Service Teams, local Community Health Committees, service agencies and Community Policing organizations, this group will coordinate responses to serious drug-related issues raised by neighbourhoods.
Lead Agency: City of Vancouver
Partner Agencies: Vancouver Police Department, Vancouver/Richmond Health Board, Office of the Attorney General (Provincial), Ministry for Children and Families, RCMP

21. Initiate a pilot Drug Treatment Court in Vancouver and advocate for creating a range of diversion programs within the criminal justice system that give individuals the option of entering treatment and support programs instead of going to trial and prison. Also explore community courts and options related to community service.
Lead Agency: Office of the Attorney General (Provincial)
Partner Agencies: Department of Justice Canada, Ministry of Health (Provincial)

22. Review existing Federal and Provincial laws and City by-laws to determine what changes are needed to give police and the courts better tools to respond to changes in the illegal drug trade such as "dial a dope" operations, public consumption of drugs, and the sexual exploitation of youth.
Lead Agencies: *Solicitor General (Federal), Office of the Attorney General (Provincial), City of Vancouver*
Partner Agencies: *Department of Justice Canada, Ministry for Children and Families*

23. Continue the redeployment of police officers in the Downtown Eastside to increase contact and visibility in the community and improve police coordination with health services and other agencies to link drug and alcohol users to
available programs.
Lead Agency: Vancouver Police Department
This initiative is also part of the Vancouver Agreement initiatives announced September 29, 2000 and cross-referenced in Appendix B.

24. *Explore legal and policy options related to the provision of mandatory treatment for a small group of repeat criminal offenders who are addicted to heroin, cocaine, or alcohol and responsible for a high percentage of crimes committed in the city.*
    Lead Agency: *Office of the Attorney General*
    Partner Agency: *Ministry of Health*

Harm Reduction

26. Provide short-term shelter and housing options for active drug users currently living on the street.
    Lead Agency: BC Housing
    Partner Agencies: City of Vancouver, Vancouver/Richmond Health Board

27. *Establish a multi-sectoral task force with representation from all levels of government to consider the feasibility of a scientific medical project to develop safe consumption facilities in Vancouver and in other appropriate areas in the region and across the country in order to reduce health risks and minimize open drug scenes.*
    Lead Agency: *Health Canada*
    Partner Agencies: *Vancouver/Richmond Health Board, City of Vancouver, Vancouver Police Department, RCMP, Attorney General*

Goal 3

Public Health: To work towards addressing the drug-related health crisis in Vancouver by reducing harm to communities and individuals, by increasing public awareness of addiction as a health issue, by reducing the HIV/AIDS/hepatitis C crisis, by reducing overdose deaths, by reducing the number of those who misuse drugs, and by providing a range of services to groups at risk such as youth, women, Aboriginal persons, and the mentally ill.

Actions:

Note: Some actions in the following section may require legislative and/or regulatory changes in order to be implemented. These and others are in italics and are noted in Appendix D.

Prevention

4. Support and fund a community led process that increases the ability of neighbourhoods within Vancouver to respond to the negative impacts of substance misuse in their communities. This process should be led by community and resident organizations in Vancouver with the goals of: increasing the awareness of substance misuse; providing information in a variety of languages on the four-pillar approach and crime prevention techniques; and supporting community- based responses to the misuse of drugs and alcohol.
   Lead Agency: City of Vancouver
   Partner Agencies: Vancouver/ Richmond Health Board, Ministry for Children and Families, National Crime Prevention Centre, Private Foundations

5. Establish a prevention/education task force to develop a pilot, city-wide school curriculum for elementary and high schools, and a public education campaign to be delivered in community centres, neighbourhood houses and through the mass media. Members would include the Vancouver School Board, Ministry of Education, Ministry for Children and Families, Vancouver/Richmond Health Board, City of Vancouver, Vancouver Park Board, Vancouver Police Department, substance misuse prevention organizations, Grief to Action Society (families and friends of addicted children) and community representatives.
   Lead Agency: Ministry for Children and Families
   Partner Agencies: Ministry of Education, Vancouver School Board, Vancouver/Richmond Health Board, Vancouver Police Department

Treatment

10. Establish the 15-bed unit at BC Women's Hospital as planned by the Vancouver/Richmond Health Board to include women with children and pregnant women who need detoxification and primary health care services related to substance misuse.
    Lead Agency: Ministry of Health
    Partner Agencies: Vancouver/Richmond Health Board

11. Establish 20 treatment beds for youth outside of the Downtown Eastside in several small, low-community-impact, residential treatment programs that: recognize the role of drug misuse and risk taking in adolescent development; have safety and the long-term well being of youth, rather than abstinence, as the overriding goal; and recognize that abstinence is also an important goal for many.
    Lead Agency: Ministry for Children and Families
    Partner Agencies: Vancouver/Richmond Health Board

12. Expand support services to families of children who are involved with substance misuse in order to breakdown stereotypes, help parents deal with feelings of guilt and anger, and help them understand addiction issues such as relapse and the often desperate measures taken by addicted youth.
    Lead Agency: Ministry for Children and Families
    Partner Agencies: Vancouver/Richmond Health Board

13. Establish six medical detox beds at St. Paul's Hospital as planned by the Vancouver/Richmond Health Boardfor those seeking to withdraw from drugs and/or alcohol and who have serious medical problems.
    Lead Agency: Ministry of Health
    Partner Agencies: Vancouver/Richmond Health Board

14. *Take steps to initiate clinical trials of a range of medications (including LAAM and Buprenorphine) for heroin and (amphetamines and cocaine) for cocaine addiction in order to increase the options that doctors have available for treatment for those who are methadone-resistant or who have not responded to treatment options over the long term.*
    Lead Agency: *Health Canada*
    Partner Agencies: *Ministry of Health*

15. *Proceed with the proposed multi-city clinical research trials into the feasibility of heroin-assisted treatment through St. Paul's Hospital and the BC Centre for Excellence in HIV/AIDS Research in Vancouver and other Canadian cities for those who are methadone-resistant or who have not responded to treatment options over the long term.*
    Lead Agency: *Health Canada*
    Partner Agencies: *Ministry of Health*

16. Expand and decentralize needle exchange services across the Vancouver/Richmond region by providing needle exchange in all primary health care clinics, hospitals, pharmacies and through non-profit groups and user groups.
    Lead Agency: Vancouver/Richmond Health Board
    Partner Agency: College of Pharmacists

17. Pilot accessible (low threshold) support programs or day centres for addicts in neighbourhoods outside of the Downtown Eastside to help prevent those who use drugs, particularly youth, from becoming more deeply involved in the inner city drug scene.
    Lead Agency: Health Canada
    Partner Agencies: Vancouver/Richmond Health Board, Ministry for Children and Families

18. Commit to creating a range of culturally appropriate strategies and services for Aboriginal persons within the four pillars of prevention, treatment, enforcement and harm reduction with a priority on the development of services for Aboriginal women with addictions and Aboriginal youth at risk.
    Lead Agencies: Vancouver Richmond Health Board, Ministry of Children and Families
    Partner Agencies: Vancouver Police Department

Enforcement

25. Explore legal and policy options to allow for mandatory drug treatment for youth involved in the illegal drug trade and severely addicted youth who are at risk of harming themselves and others as a result of their addiction.
    Lead Agency: *Ministry for Children and Families*
    Partner Agency: *Office of the Attorney General*

Harm Reduction

28. Implement an overdose death prevention campaign that involves the Vancouver/ Richmond Health Board, Vancouver Police, BC Ambulance Service, City of Vancouver, drug user organizations and community agencies to develop overdose prevention strategies.
    Lead Agency: Vancouver/Richmond Health Board
    Partner Agencies: Vancouver Police Department, BC Ambulance Service, City of Vancouver

29. Establish testing procedures for street drugs that will give the community quick access to information about the changes in purity and quality of street drugs in order to prevent drug overdose and deaths. Develop official policy to support efforts to test drugs for content and purity at Rave parties.
    Lead Agency: Vancouver Police Department
    Partner Agencies: RCMP, B.C. Coroners Office, City of Vancouver, Vancouver/Richmond Health Board

30. Advocate for appropriate housing for those with mental illness and dual-diagnosis problems throughout the region.
    Lead Agency: Vancouver/Richmond Health Board
    Partner Agencies: BC Housing, City of Vancouver, Ministry of Health

Goal 4

Coordinate, Monitor and Evaluate: To advocate for the establishment of single, accountable agent to coordinate implementation of the actions in this framework, and to monitor and evaluate implementation through senior representatives of the Vancouver/Richmond Health Board, the Vancouver Police Department, the City of Vancouver, the BC Centre for Disease Control, the Ministry for Children and Families, the Office of the Attorney General, and community representatives.

Action:

31. Oversee balanced implementation of the four-pillar approach; prevention, treatment, enforcement and harm reduction.

Appendix B
Vancouver Agreement Announcements

Summary of September 29, 2000 First Focus Announcements

In September 2000, the federal and provincial governments along with the City under the *Vancouver Agreement*, announced the first phase of a program to address the urgent and complex social, economic and health and safety issues of the Downtown Eastside. This was an important first step.

Specific Announcements – September 29, 2000

- Establishment of a Downtown Eastside Treatment Centre with new and expanded treatment services to close gaps in care systems and improve their effectiveness. The centre will provide a range of detox, sobering services, stabilization services, outreach, and methadone therapy.
- Expansion of treatment services (sobering and detox services) throughout the city.
- Creation of an indoor Health Connection program to provide frontline health and substance misuse referral services, life skills training and social support programs for street involved drug and alcohol users.

The total cost of the preceding three initiatives is $2.1 million.

- Redeploy police officers in the Downtown Eastside to increase contact and visibility in the community.
  Cost: $1.6 million (of re-allocated funds)
- Improve police coordination with health services and other agencies to link drug and alcohol users to available programs.
- Stepped up enforcement efforts targeting drug dealers.
- A physical re-design of the Carnegie Centre entrance to reduce illegal drug activity at the corner of Main and Hastings Streets.
  Cost: $200,000
- Expansion of street improvement programs including expanded graffiti removal and needle and drug paraphernalia pick-up.
  Cost: $180,000
- New housing developments located in various neighbourhoods
  Cost: $7.5 million
- The establishment of the Partners in Economic and Community Help fund to provide loans, loan guarantees, grants and lease subsidies to organizations and businesses in the Downtown Eastside area.
  Cost: $2.3 million

These nine initiatives will involve a total investment of $13.9 million in the community.

Appendix C
A List of Some Reports and Recommendations (1994-2000):Summary

1. Cain, J.V. (1994). Report of the Task Force into Illicit Narcotic Overdose Deaths in British Columbia, detailed 65 recommendations. Key among these are:
   - Establish an independent Substance Abuse Commission.
   - Improve education for the public and emergency service providers on how to deal with overdose situations.
   - Provide more facilities for detox, treatment, recovery and outreach, including needle exchange, Narcan, and methadone treatment.
   - Within the overall framework of harm reduction, review the feasibility of providing a heroin maintenance program.
   - Consider transferring responsibility for BC's methadone dispensing program from the federal Bureau of Dangerous Drugs to the provincial Ministry of Health.
   - Provide more substantial funding for supportive recovery programs.
   - Provide better education to social assistance and ministry employees on substance abuse to give them a greater understanding of the addict and addiction.
   - Establish educational programs in parenting and life skills, and job placement strategies for welfare recipients.
   - Review low rental premises to ensure accommodation standards are met.
   - Increase availability of appropriate housing options, such as community homes, independent living apartments, safe houses, and transition houses for recovering addicts in order to stop exposure to an alcohol or drug environments.
   - Establish treatment centres for family substance abuse.
   - Provide adequate daycare, travel, and financial support to mothers attending substance abuse treatment programs.
   - Improve access to detox facilities for young substance abusers and develop follow-up programs for parents and youth.
   - Invite First Nations people to participate more fully in the planning of regional and local services and programs.
   - Ensure the mentally ill have the necessities of life and community support.
   - Provide alcohol and detox treatment programs and mandatory educational programs for young offenders.
   - Develop locally relevant teaching modules within the secondary school curriculum that deal with life skills, substance abuse, coping, and parenting.
   - Consider the feasibility of decriminalizing the possession and use of specified substances by addicted users.

- Consider the merits of legalizing the possession of "soft" drugs such as marijuana.

2. Millar, J.S. (1998). HIV, Hepatitis, and Injection Drug Use in British Columbia – Pay Now or Pay Later, a report by the provincial health officer emphasizes the human and monetary costs of substance misuse and recommends a Harm Reduction approach:
   - Establish a provincial Substance Abuse Commission.
   - Provide adequate mental health services, health care, housing, and social support to IDU's at all stages of addiction and recovery.
   - Adopt the principal that all children in BC have access to good quality childcare.
   - Improve coordination between mental health and addiction services.
   - Discontinue user fees for methadone therapy.
   - As part of a comprehensive harm reduction approach, pilot a program to test controlled, legal availability of heroin for addicts.
   - Increase methadone availability to serve an additional 1,000 addicts in Vancouver.
   - Increase detox, residential care, counselling, and other therapies for non-heroin injection drug u sers by 50%.

3. The Downtown Eastside / Strathcona Alcohol and Drug Advisory Committee conducted a series of focus groups to produce Community Voices (1994), a report outlining 16 recommendations based on access, prevention, coordination, and participation.
   - Improve access to detox services and establish a community-based clinic that offers 24-hour alcohol and drug services.
   - Special consideration should be given to the needs of women, children, seniors, First Nations people, people with dual diagnosis, and those whose first language is not English in the design and delivery of drug service.
   - Improve prevention and information programs for all groups.
   - Improve coordination and communication between services that deal with alcohol and drug issues.
   - Ensure participation and representation by all members of the community in all aspects of policy development and decision making.

4. Dandurand and Chin (2000). Injection Drug Use and the Epidemic of HIV in the Lower Mainland, a report by the Lower Mainland Working Group on Communicable Diseases (LMMA) outlines several organizational and service recommendations:
   - Establish an inter-regional, inter-sectoral task group to develop coordinated strategies for the prevention and treatment of drug addiction across the Lower Mainland.
   - Health regions should include injection drug users wherever possible in planning services.

- Undertake a public education program to aid in the reconceptualization of addiction as a health issue, and promote harm reduction as a sensible public health approach.
- Ensure multiple needle exchange services are available 24 hours a day, 7 days a week.
- Increase mobile health services, detox services and adequate transitional housing within an expanded and integrated system of care.
- Ensure that comprehensive methadone treatment and counselling are readily available without charge.
- Expand HIV treatment to injection drug users as their addictions stabilize with treatment.
- Address training and safety needs for hospital staff.

5. A Case for an Independent Substance Abuse Prevention and Addictions Commission, (2000), a report by the Kaiser Youth Foundation, identifies inadequate education and prevention strategies, disparities in funding for treatment and services, gaps in these services, and the need to coordinate government and non-government activities. It recommends the following:

- Establish a Substance Abuse Prevention and Addictions Commission as an autonomous agency linked to government though an Order-in-Council.
- Develop a comprehensive strategic plan for the reduction of substance abuse.
- Develop information systems to support evidence-based decision-making.

Appendix D
Placeholder for Actions that May Require Legislative and/or Regulatory Changes in Order to be Implemented

Acknowledgements

The City of Vancouver would like to acknowledge all government and non-government and community organizations whose reports and work have been referenced in this paper and have helped to shape the content of this paper, including: the Lower Mainland Municipal Association, BC Ministry for Children and Families, BC Ministry of Health, BC Chief Coroner's Office, Vancouver/Richmond Health Board, Kaiser Youth Foundation, Vancouver Police, Lower Mainland Working Group on Communicable Diseases, the BC Medical Association, the Downtown Eastside/ Strathcona Drug and Alcohol Advisory Committee and Vancouver's Coalition for Crime Prevention and Drug Treatment.

The partners of *Vancouver's Coalition for Crime Prevention and Drug Treatment* are:

Vancouver School Board
Vancouver Board of Trade
Vancouver International Airport Authority
University of British Columbia
S.U.C.C.E.S.S
United Way
Downtown Vancouver BIA
Rotary Club of Vancouver
Health Canada
Insurance Corporation of British Columbia
Kaiser Youth Foundation
Collingwood CPC
Vancouver Recovery Club
Boys & Girls Club of Greater Vancouver
Hope in Vision
Downtown Vancouver Association
Salvation Army
Mount Pleasant BIA
BARWATCH
Kerrisdale BIA
Alcohol - Drug Education Service
Robson Street BIA
Chinese CPC
Odd Squad Productions
Grandview-Woodlands CPC
Cedar Cottage Community Policing Centre
Circle of Hope Coalition Society
The International Dyslexia Association, BC Branch
Vancouver Police Native Liaison Society
Together We Can
Vancouver Park Board
Vancouver Port Corporation
Tourism Vancouver
Volunteer Vancouver
Simon Fraser University
Vancouver Foundation.
VanCity Credit Union
Vancouver Hotel Association
Royal Canadian Mounted Police
Insurance Bureau of Canada
Browning - Ferris Industries (BFI)
The Gathering Place
The United Youth Movement
The British Columbia Regiment
Vancouver Family Court & Youth Justice Committee
Taiwanese - Canadian Cultural Society
Downtown Eastside Youth Activities Society
Mount Pleasant CPC
Granville CPC
Kensington Community Centre
First United Church
Vancouver Economic Development Commission
YWCA of Vancouver
Davie Street CPC
Concert Properties Ltd.
Canadian Bankers Association
Renfrew Collingwood Drug & Alcohol Committee
Anglican Diocese of New Westminster
BC Coalition for Safer Communities

References

Anderson, D. In New York City, a Community Court and a New Legal Culture, 1996.

Anderson, J.F. Drug Courts: Moving Towards the Establishment of a Provincial Position. Draft, May 19, 2000.

Anderson, J.F. Interpreting the Relation Between Injection Drug Use and Harm: a Cautionary Note. Canadian Medical Association Journal, June 13, 2000

Alexander, B.K., Tsou, J.Y. Prospects for Stimulant Maintenance, in press, 1998.

Bognar, Legare and Ross. Injection Drug Use and the Epidemic of HIV in the Lower Mainland. Lower Mainland Working Group on Communicable Diseases, September 1998.

Cain, J.V. Report of the Task Force into Illicit Narcotic Overdose Deaths in British Columbia. Office of the Chief Coroner, British Columbia, September 1999.

Campbell, Drucker, Weber. Episode 9, Transcripts of Interviews with Chief Coroner Larry Campbell, Dr. Ernest Drucker, and Dr. Urban Weber. City of Vancouver, 2000.

The Canadian HIV/AIDS Legal Network. Injection Drug Use and HIV/AIDS: Legal and Ethical Issues. Health Canada, 1999.

Canadian Centre for Substance Abuse, 2000. http://www.ccsa.ca/costhigh.htm

Chand, Manjit and Thompson. You Have Heard this Before: Street Involved Youth and Service Gaps, 1997.

City of Vancouver, Social Planning Department. Homeless Street Youth in Downtown South: A Snapshot Study, 2000.

City of Vancouver, Background Paper on Drug Treatment Needs in Vancouver, Reports for Public Discussion, July 1998

City of Vancouver, Housing Plan for the Downtown Eastside, Chinatown, Gastown, Strathcona (draft) 1998

Clarke, J. Report on Visit to Drug Programs in Amsterdam, Netherlands and Frankfurt, Germany, 2000.

Community Voices: A report by the Downtown Eastside / Strathcona Alcohol and Drug Advisory Committee, March 1994.

The Drug Policy Letter, January/February 2000.

A Drug Strategy for British Columbia: A Discussion Paper by the Federal/Provincial Harm Reduction Working Group, June 12, 2000.

Dandurand and Chin. Towards a Lower Mainland Crime and Drug Misuse Prevention Strategy: Needs Assessment and Identification of Issues. Lower Mainland Municipal Association (LMMA), August 2000.

From Grief to Action, Statement of Goals, 2000.

Fry and Testro. Not Just for Us. Safe Injecting Rooms: A survey of the attitudes of 215 street-based heroin users in Melbourne, Victoria. The

Youth Substance Abuse Service in Association with Turning Point Alcohol and Drug Centre. Australia, April 2000.

Gardner, Dan. How America Dictates the Global War on Drugs. The Vancouver Sun, September 5-18, 2000.

Harm Reduction: Concepts and Practise: A Policy Discussion Paper. Canadian Centre on Substance Abuse (CCSA) National Working Group on Policy, 1999.

Harm Reduction Network Publication, 1994.

HIV/AIDS and Injection Drug Use: A National Action Plan. www.cfdp.hivaids.html

Kaiser Youth Foundation. A Case for an Independent Substance Abuse Prevention and Addictions Commission, May 2000.

Kendall, P. A Drug Strategy for British Columbia: Discussion Paper. BC Ministry of Health and Ministry Responsible for Seniors, June 12, 2000.

King County Public Health. Drug Abuse Trends in Seattle-King County Area, May 2000

MacPherson, D. Comprehensive Systems of Care for Drug Users in Switzerland and Frankfurt, Germany. Social Planning Department, City of Vancouver, June 1999.

McLean, M. Vancouver Drug Epidemiology and Drug Crime Statistics 2000. Canadian Community Epidemiology Network on Drug Use (CCENDU), 2000.

Ministry for Children and Families for British Columbia. Review of Alcohol and Drug Services in Vancouver, May 1997.

Millar, J. S. HIV, Hepatitis and Injection Drug Use in British Columbia – Pay Now or Pay Later? BC Ministry of Health, June 1998.

Millar, J.S. Canada's Drug Problem: Time to Get Serious. Letter in the Canadian Medical Association Journal, February 23, 1999.

Municipal Health Service, Amsterdam. Care for the Future: Opiate Addicts in Amsterdam, 1999.

North American Opiate Medications Initiative Working Group. A Multi-Centre, Randomized Controlled Trial of Heroin-Assisted Therapy for Injecting Opiate Users, 1998.

Osborn, Bud. Hundred Block Rock. Arsenal Pulp Press, Vancouver, 1999.

Paglia and Room. Preventing Substance-Use Problems Among Youth: A Literature Review and Best-Advice Paper, 1998.

Parker, H. Methadone Maintenance and Crime Reduction on Merseyside, Crime Detection and Prevention Series, 1997.

Poirier, A. Let Me Go: Comprehensive Solutions to Drugs, Crime and Addiction in the Inner City (film transcript). National Film Board of Canada, 1998.

Riley and O'Hare. Harm Reduction: History, Definition and Practice: First, Do

No Harm. The Canadian Centre of Substance Abuse National Working Group on Drug Policy, 1999.

Robinson, Jeffrey. The Merger, How Organized Crime is Taking Over Canada and the World. McClelland and Stewart, 1999.

Rosenbaum, Marsha. Safety First: A Reality-Based Approach to Teens, Drugs, and Drug Education, The Lindesmith Centre – West, 1999

Royal Canadian Mounted Police Criminal Intelligence Directorate. Drug Situation in Canada, 2000.

Single, Eric. A Harm Reduction Framework for Drug Policy in British Columbia. The BC Federal/Provincial Harm Reduction Working Group, December 1999.

SAPAC-BC. A Case for an Independent Substance Abuse and Prevention and Addictions Commission, May 2000.

Substance Abuse and Mental Health Services Administration, U.S. Department of Health and Human Services. Drug Abuse Warning Network Annual Medical Examiner Data, 1998.

Vancouver City Council. Policy Report. March 28, 2000.

Vancouver Agreement, Comprehensive Substance Misuse Strategy, August 2000.

Vancouver Drug Treatment Court. Summary Document, 1999.

Vancouver Injection Drug User Survey (VIDUS), 2000

Vancouver Police Department. Youth at Risk Research. September, 2000

Victoria (Australia) Drug Policy Expert Committee. Drugs: Responding to the Issues, Engaging the Community, April 2000.

Feedback Form—What do you think?

A) Do you support or oppose the major goals in *A Framework for Action?*

Goal 1.
Provincial and Federal Responsibility: To persuade other levels of government to take action and responsibility for elements of the framework within their jurisdiction by encouraging a regional approach to the development of services, and by demonstrating the city-wide, regional, national and international implications of the drug problems in Vancouver.

Please circle one: ***strongly support*** ***somewhat support*** ***somewhat oppose*** ***strongly oppose***

Goal 2.
Public Order: To work towards the restoration of public order across Vancouver by reducing the open drug scene (particularly at Main and Hastings), by reducing the negative impact of illicit drugs on our community, by reducing the impact of organized crime on Vancouver communities and individuals, by providing neighbourhoods, organizations and individuals with a place to go (such as a healthy-city office) with their concerns related to safety, criminal activity, drug misuse, and related problems, and by implementing crime prevention techniques to increase public safety.

Please circle one: ***strongly support*** ***somewhat support*** ***somewhat oppose*** ***strongly oppose***

Goal 3.
Public Health: To work towards addressing the drug-related health crisis in Vancouver by reducing harm to communities and individuals, by increasing public awareness of addiction as a health issue, by reducing the HIV/AIDS/hepatitis C crisis, by reducing overdose deaths, by reducing the number of those who misuse drugs, and by providing a range of services to groups at risk such as youth, women, Aboriginal persons, and the mentally ill.

Please circle one: ***strongly support*** ***somewhat support*** ***somewhat oppose*** ***strongly oppose***

Goal 4.
Coordinate, Monitor and Evaluate: To advocate for the establishment of a single, accountable agent to coordinate implementation of the actions in this framework, to monitor and evaluate implementation through senior representatives of the Vancouver/Richmond Health Board, the Vancouver Police Department, the City of Vancouver, the BC Centre for Disease Control, Ministry of Children and Families, the Office of the Attorney General, and community representatives.

Please circle one: ***strongly support*** ***somewhat support*** ***somewhat oppose*** ***strongly oppose***

B) What are your overall impressions of the four-pillar approach to drug misuse, including *prevention, treatment, enforcement* and *harm reduction?*

Please circle one: ***excellent*** ***very good*** ***good*** ***fair*** ***poor***

Regional and National Drug Strategy (Actions 1-3)

1. The **Provincial ministries responsible** implement policy that ensures municipalities throughout British Columbia support the development of a full range of drug and alcohol services.

Please circle one: ***strongly support*** ***somewhat support*** ***somewhat oppose*** ***strongly oppose***

2. The **Provincial Government** implement a policy framework for reducing the harm to the community and individuals associated with alcohol, tobacco and illicit drugs to guide and inform municipal decision makers in determining priorities for action.

Please circle one: ***strongly support*** ***somewhat support*** ***somewhat oppose*** ***strongly oppose***

3. The **Federal Government** take strong leadership in the following areas:

- *Review existing laws with regard to illicit drugs, organized crime, gathering of evidence in drug cases and protection of youth.*
- Implement new money laundering legislation.
- *Review existing laws and procedures to deal with refugee claimants who are engaged in the illegal drug trade.*
- *Initiate research and development of alternative pharmacotherapies for drug addiction including: Levo-alpha-acetyl-methadol (or, LAAM, a derivative of methadone that is long-acting), Buprenorphine (an alternate therapy for heroin users), Amphetamines and other drugs to treat cocaine addiction.*
- *Provide leadership in the development of national research into the feasibility of such initiatives as: heroin-assisted treatment, safe injection or consumption rooms, low threshold methadone prescribing practices and other innovative approaches to addiction treatment and the reduction of drug-related harm to individuals and communities.*

Please circle one: ***strongly support*** ***somewhat support*** ***somewhat oppose*** ***strongly oppose***

Prevention (Actions 4 -7)

Action 4

Support and fund a community-led process that increases the ability of neighbourhoods within Vancouver respond to the negative impacts of substance misuse in their communities. This process should be led by community and resident organizations in Vancouver neighbourhoods with the goals of: increasing the awareness of substance misuse; providing information in a variety of languages on the four-pillar approach and crime prevention techniques; and supporting community-based responses to the misuse of drugs and alcohol.
Lead Agency: City of Vancouver
Partner Agencies: Vancouver/ Richmond Health Board, Ministry of Children and Families, National Crime Prevention Centre, Private Foundations

Please circle one: ***strongly support*** ***somewhat support*** ***somewhat oppose*** ***strongly oppose***

Action 5
Establish a prevention/education task force to develop a pilot, city-wide school curriculum for elementary and high schools, and a public education campaign to be delivered in community centres, neighbourhood houses and through the mass media. Members would include the Vancouver School Board, Ministry of Education, Ministry of Children and Families, Vancouver/Richmond Health Board, City of Vancouver, Vancouver Park Board, Vancouver Police Department, substance misuse prevention organizations, Grief to Action Society (families and friends of addicted children) and community representatives.
Lead Agency: Ministry for Children and Families
Partner Agencies: Ministry of Education, Vancouver School Board, Vancouver/Richmond Health Board, Vancouver Police Department

Please circle one: ***strongly support*** ***somewhat support*** ***somewhat oppose*** ***strongly oppose***

Action 6
Explore options for distributing BC Benefit cheques throughout the month in order to reduce harm to individuals and the community by decreasing the sale and use of drugs and alcohol at one time during the month by those on BC Benefits who suffer from addiction and mental health problems.
Lead Agency: Ministry of Social Development and Economic Security

Please circle one: ***strongly support*** ***somewhat support*** ***somewhat oppose*** ***strongly oppose***

Action 7
Consider the creation of a Healthy City Office within the City of Vancouver in order to support a coordinated response to community health and safety and crime prevention in the city and to promote and support projects that work towards creating healthier and safer neighbourhoods within Vancouver.
Lead Agency: City of Vancouver
Partner Agencies: Vancouver/Richmond Health Board, Ministry of Children and Families, Vancouver Police Department.

Please circle one: ***strongly support*** ***somewhat support*** ***somewhat oppose*** ***strongly oppose***

Treatment (Actions 8-18)

Action 8
Increase methadone availability by removing current barriers (such as user fees, counselling fees, and restrictive regulations) for the methadone maintenance program in order to treat an additional 1,000 clients in the Lower Mainland over the next two years, with the Downtown Eastside as a priority area for expansion. Continue the expansion of the Provincial Methadone Maintenance Treatment programs within other areas across Vancouver and the province where there is a highly marginalized group of opiate users and those who use opiates and stimulants in combination.
Lead Agency: Ministry of Health
Partner Agencies: College of Physicians and Surgeons, Ministry of Children and Families, Vancouver/Richmond Health Board

Please circle one: ***strongly support*** ***somewhat support*** ***somewhat oppose*** ***strongly oppose***

Action 9

Ensure that a continuum of long-term supportive housing is developed including housing and/or shelter to stabilize those who misuse drugs and alcohol, and drug- and alcohol-free housing for individuals in recovery.

Lead Agency: BC Housing

Partner Agencies: Vancouver/Richmond Health Board, City of Vancouver

Please circle one: ***strongly support*** ***somewhat support*** ***somewhat oppose*** ***strongly oppose***

Action 10

Establish the 15-bed unit at BC Women's Hospital as planned by the Vancouver Richmond Health Board to include women with children and pregnant women who need detoxification and primary health care services related to substance misuse.

Lead Agency: Ministry of Health

Partner Agencies: Vancouver/Richmond Health Board

Please circle one: ***strongly support*** ***somewhat support*** ***somewhat oppose*** ***strongly oppose***

Action 11

Establish 20 treatment beds for youth outside of the Downtown Eastside in several small, low-community-impact, residential treatment programs that: recognize the role of drug misuse and risk taking in adolescent development; have safety and the long-term well being of youth, rather than abstinence, as the overriding goal; and recognize that abstinence is also an important goal for many.

Lead Agency: Ministry of Children and Families

Partner Agency: Vancouver/Richmond Health Board

Please circle one: ***strongly support*** ***somewhat support*** ***somewhat oppose*** ***strongly oppose***

Action 12

Expand support services to families of children who are involved with substance misuse in order to breakdown stereotypes, help parents deal with feelings of guilt and anger, and help them understand addiction issues such as relapse and the often desperate measures taken by addicted youth.

Lead Agency: Ministry of Children and Families

Partner Agency: Vancouver/Richmond Health Board

Please circle one: ***strongly support*** ***somewhat support*** ***somewhat oppose*** ***strongly oppose***

Action 13

Establish six medical detox beds at St. Paul's Hospital as planned by the Vancouver/Richmond Health Board for those seeking to withdraw from drugs and/or alcohol and who have serious medical problems.

Lead Agency: Ministry of Health

Partner Agency: Vancouver/Richmond Health Board

Please circle one: ***strongly support*** ***somewhat support*** ***somewhat oppose*** ***strongly oppose***

Action 14

Take steps to initiate clinical trials of a range of medications (including LAAM and Buprenorphine) for heroin and (amphetamines and cocaine) for cocaine addiction in order to increase the options that doctors have available for treatment for those who are methadone-resistant or who have not responded to treatment options over the long term.

Lead Agency: Health Canada
Partner Agency: Ministry of Health

Please circle one: ***strongly support*** ***somewhat support*** ***somewhat oppose*** ***strongly oppose***

Action 15

Proceed with the proposed multi-city clinical research trials into the feasibility of heroin-assisted treatment through St. Paul's Hospital and the BC Centre for Excellence in HIV/AIDS Research in Vancouver and other Canadian cities for those who are methadone-resistant or who have not responded to treatment options over the long term.

Lead Agency: Health Canada
Partner Agency: Ministry of Health

Please circle one: ***strongly support*** ***somewhat support*** ***somewhat oppose*** ***strongly oppose***

Action 16

Expand and decentralize needle exchange services across the Vancouver/Richmond region by providing needle exchange in all primary health care clinics, hospitals, pharmacies and through non-profit groups and user groups.

Lead Agency: Vancouver/Richmond Health Board
Partner Agency: College of Pharmacists

Please circle one: ***strongly support*** ***somewhat support*** ***somewhat oppose*** ***strongly oppose***

Action 17

Pilot accessible (low threshold) support programs or day centres for addicts in neighbourhoods outside of the Downtown Eastside to help prevent those who use drugs, particularly youth, from becoming more deeply involved in the inner city drug scene.

Lead Agency: Health Canada
Partner Agencies: Vancouver/Richmond Health Board, Ministry for Children and Families

Please circle one: ***strongly support*** ***somewhat support*** ***somewhat oppose*** ***strongly oppose***

Action 18

Commit to creating a range of culturally appropriate strategies and services for Aboriginal persons within the four pillars of prevention, treatment, enforcement and harm reduction with a priority on the development of services for Aboriginal women with addictions and Aboriginal youth at risk.

Lead Agencies: Vancouver Richmond Health Board, Ministry of Children and Families
Partner Agencies: Vancouver Police Department

Please circle one: ***strongly support*** ***somewhat support*** ***somewhat oppose*** ***strongly oppose***

Enforcement (Actions 19-25)

Action 19

Increase the Organized Crime Unit, the Vancouver Police Drug Squad and the RCMP Drug Squad unit in order to better target organized crime, drug houses that cause neighbourhood disruption and mid and upper level drug dealers that supply street level drug dealers.

Lead Agencies: Solicitor General (Federal), Office of the Attorney General (Provincial), City of Vancouver

Partner Agencies: Vancouver Police Department

Please circle one: ***strongly support*** ***somewhat support*** ***somewhat oppose*** ***strongly oppose***

Action 20

Institute a senior-level Drug Action Team comprised of senior staff from: Vancouver Police, City of Vancouver, Vancouver/Richmond Health Board, the Attorney General's office, Ministry of Children and Families, the RCMP and community representatives. In coordination with local Neighbourhood Integrated Service Teams, local Community Health Committees, service agencies and Community Policing organizations, this group will coordinate responses to serious drug-related issues raised by neighbourhoods.

Lead Agency: City of Vancouver

Partner Agencies: Vancouver Police Department, Vancouver/Richmond Health Board, Office of the Attorney General (Provincial), Ministry of Children and Families, RCMP

Please circle one: ***strongly support*** ***somewhat support*** ***somewhat oppose*** ***strongly oppose***

Action 21

Initiate a pilot Drug Treatment Court in Vancouver and advocate for creating a range of diversion programs within the criminal justice system that give individuals the option of entering treatment and support programs instead of going to trial and prison. Also explore community courts and options related to community service.

Lead Agency: Office of the Attorney General (Provincial)

Partner Agencies: Department of Justice Canada, Ministry of Health (Provincial)

Please circle one: ***strongly support*** ***somewhat support*** ***somewhat oppose*** ***strongly oppose***

Action 22

Review existing Federal and Provincial laws and City by-laws to determine what changes are needed to give police and the courts better tools to respond to changes in the illegal drug trade such as "dial a dope" operations, public consumption of drugs, and the sexual exploitation of youth.

Lead Agencies: Solicitor General (Federal), Office of the Attorney General (Provincial), City of Vancouver

Partner Agencies: Department of Justice Canada, Ministry of Children and Families

Please circle one:

strongly support ***somewhat support*** ***somewhat oppose*** ***strongly oppose***

Action 23
Continue the redeployment of police officers in the Downtown Eastside to increase contact and visibility in the community and improve police coordination with health services and other agencies to link drug and alcohol users to available programs.
Lead Agency: Vancouver Police Department
This initiative is also part of the Vancouver Agreement initiatives announced September 29, 2000 and cross-referenced in Appendix B.

Please circle one: ***strongly support*** ***somewhat support*** ***somewhat oppose*** ***strongly oppose***

Action 24
Explore legal and policy options related to the provision of mandatory treatment for a small group of repeat criminal offenders who are addicted to heroin, cocaine, or alcohol and responsible for a high percentage of crimes committed in the city.
Lead Agency: Office of the Attorney General
Partner Agency: Ministry of Health

Please circle one: ***strongly support*** ***somewhat support*** ***somewhat oppose*** ***strongly oppose***

Action 25
Explore legal and policy options to allow for mandatory drug treatment for youth involved in the illegal drug trade and severely addicted youth who are at risk of harming themselves and others as a result of their addiction.
Lead Agency: Ministry of Children and Families
Partner Agency: Office of the Attorney General

Please circle one: ***strongly support*** ***somewhat support*** ***somewhat oppose*** ***strongly oppose***

Harm Reduction (Actions 26-31)

Action 26
Provide short-term shelter and housing options for active drug users currently living on the street.
Lead Agency: BC Housing
Partner Agencies: City of Vancouver, Vancouver/Richmond Health Board

Please circle one: ***strongly support*** ***somewhat support*** ***somewhat oppose*** ***strongly oppose***

Action 27
Establish a multi-sectoral task force with representation from all levels of government to consider the feasibility of a scientific medical project to develop safe consumption facilities in Vancouver and in other appropriate areas in the region and across the country in order to reduce health risks and minimize open drug scenes.
Lead Agency: Health Canada
Partner Agencies: Vancouver/Richmond Health Board, City of Vancouver, Vancouver Police Department, RCMP, Attorney General

Please circle one: ***strongly support*** ***somewhat support*** ***somewhat oppose*** ***strongly oppose***

Action 28
Implement an overdose death prevention campaign that involves the Vancouver/Richmond Health Board, Vancouver Police, BC Ambulance Service, City of Vancouver drug user organizations and community agencies to develop overdose prevention strategies.
Lead Agency: Vancouver/Richmond Health Board
Partner Agencies: Vancouver Police Department

Please circle one: strongly support somewhat support somewhat oppose strongly oppose

Action 29
Establish testing procedures for street drugs that will give the community quick access to information about the changes in purity and quality of street drugs in order to prevent drug overdose and deaths. Develop official policy to support efforts to test drugs for content and purity at Rave parties.
Lead Agency: Vancouver Police Department
Partner Agencies: RCMP, B.C. Coroners Office, City of Vancouver, Vancouver/Richmond Health Board

Please circle one: strongly support somewhat support somewhat oppose strongly oppose

Action 30
Advocate for appropriate housing for those with mental illness and dual-diagnosis problems throughout the region
Lead Agency: Vancouver/Richmond Health Board
Partner Agencies: BC Housing, City of Vancouver, Ministry of Health

Please circle one: strongly support somewhat support somewhat oppose strongly oppose

Action 31
To advocate for the establishment of a single, accountable agent to coordinate implementation of the actions in this framework, to monitor and evaluate implementation through senior representatives of the Vancouver/Richmond Health Board, the Vancouver Police Department, the City of Vancouver, the BC Centre for Disease Control, Ministry of Children and Families, the Office of the Attorney General, and community representatives.

Please circle one: strongly support somewhat support somewhat oppose strongly oppose

Public Consultations and City-Wide Community Dialogues

Please indicate with a ✔ how you would like to be consulted:

_____written feedback form _____email

_____fax _____small group meetings

_____public forums _____other, please specify ______________________________

Comments or Suggestions?

Prevention

See Prevention Actions 4-7 for reference.

Treatment

See Treatment Actions 8-18 for reference.

Enforcement

See Enforcement Actions 19-25 for reference.

Harm Reduction

See Harm Reduction Actions 26-30 for reference.

General Comments

(Please attach further comments if desired)

Optional

Name or Organization : ________________________________

Phone: ________________________________

Thank you for taking the time to provide your comments.

Please return your feedback form to:

Donald MacPherson,
c/o Vancouver's Coalition for Crime Prevention
and Drug Treatment
#201-111 Water Street
Vancouver, BC V6B 1A7

Fax: (604) 688-7302

You can also obtain a copy of the discussion paper and/or provide your feedback through the **websites** at:

www.city.vancouver.bc.ca
www.crimepreventiondrugtreatment.com
