# Supplementary material for: Amazing grace: Vancouver's supervised injection facility granted six-month lease on life
Source: Harm Reduct J. 2008 Jan 24;5:3. doi: 10.1186/1477-7517-5-3 (PMC2233622; doi:10.1186/1477-7517-5-3)
Supplement: Additional file 2 — Return on Investment in Needle and Syringe Programs in Australia: Report. The National Centre for HIV Epidemiology and Clinical Research, Publications Production Unit: Commonwealth of Australia; 2002. [file 1477-7517-5-3-S2.pdf]

AUSTRALIA

Return on Investment in Needle and Syringe Programs in Australia

Report

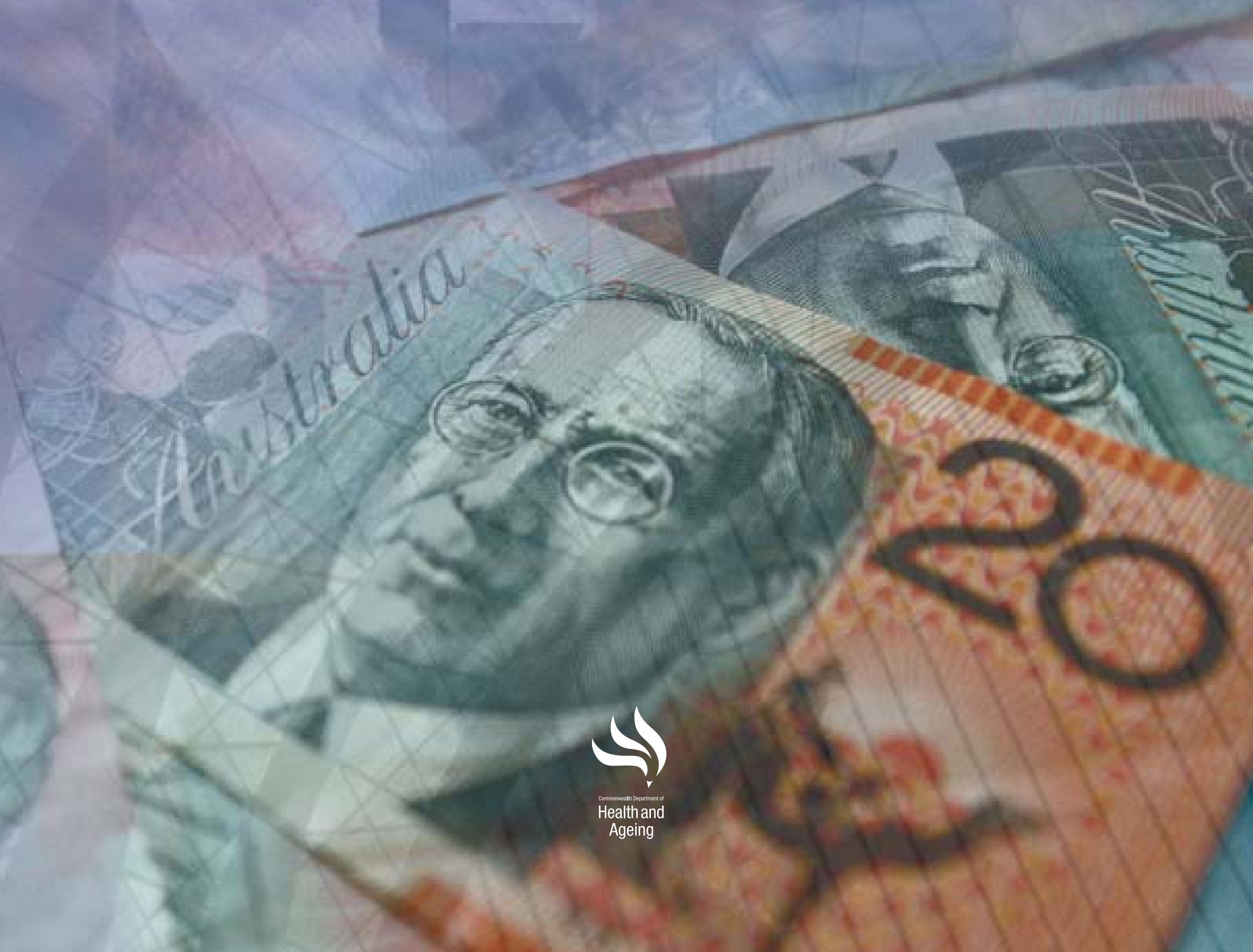

Commonwealth Department of
Health and
Ageing

Return on Investment in Needle and Syringe Programs in Australia • Report

COMMONWEALTH DEPARTMENT OF HEALTH AND AGEING

RETURN ON INVESTMENT IN NEEDLE & SYRINGE PROGRAMS IN AUSTRALIA

REPORT

HEALTH OUTCOMES INTERNATIONAL PTY LTD

IN ASSOCIATION WITH

THE NATIONAL CENTRE FOR HIV EPIDEMIOLOGY AND CLINICAL RESEARCH

AND

PROFESSOR MICHAEL DRUMMOND, CENTRE OF HEALTH ECONOMICS, YORK UNIVERSITY

© Commonwealth of Australia 2002

ISBN 0 642 82116 X

This work is copyright. Apart from any use as permitted under the *Copyright Act 1968*, no part may be reproduced by any process without prior written permission from the Commonwealth available from the Department of Communications, Information Technology and the Arts. Requests and inquiries concerning reproduction and rights should be addressed to the Manager, Copyright Services, Info Access, GPO Box 1920, Canberra ACT 2601.

Publication approval number: 3122

Publications Production Unit (Public Affairs, Parliamentary and Access Branch)
Commonwealth Department of Health and Ageing

EXECUTIVE SUMMARY 1
EFFECTIVENESS OF NSPS FOR PREVENTING TRANSMISSION OF HIV AND HCV INFECTION 1
FINANCIAL EFFECTS OF NSPS 2
QUALITY OF LIFE (QOL) EFFECTS OF NSPS 4
CONCLUSION 6
1 INTRODUCTION 7
1.1 BACKGROUND 7
1.2 OBJECTIVES 7
1.3 METHODOLOGY 8
1.4 ACKNOWLEDGEMENT 8
2 AN OVERVIEW OF NEEDLE AND SYRINGE PROGRAMS (NSPS) 9
2.1 AUSTRALIA'S STRATEGIES ON HIV/AIDS AND HEPATITIS C 9
2.2 OVERVIEW OF NEEDLE AND SYRINGE PROGRAMS 9
2.3 OPERATIONS OF NEEDLE AND SYRINGE PROGRAMS 10
3 THE IMPACT OF NSPS ON HIV AND HCV 12
3.1 EFFECTIVENESS OF NSPS FOR PREVENTING TRANSMISSION OF HIV AND HCV INFECTION 12
3.2 METHODOLOGY FOR ESTIMATES OF NUMBERS OF PEOPLE LIVING WITH HIV ACQUIRED THROUGH INJECTING DRUGS 25
3.3 METHODOLOGY FOR ESTIMATES OF NUMBERS OF PEOPLE LIVING WITH HCV ACQUIRED THROUGH INJECTING DRUGS 27
3.4 NUMBER OF HIV INFECTIONS PREVENTED THROUGH THE INTRODUCTION OF NSPS 29
3.5 NUMBER OF HCV INFECTIONS PREVENTED THROUGH THE INTRODUCTION OF NSPS 30
4 FINANCIAL EFFECTS OF NSPS 31
4.1 DESCRIPTION OF THE ECONOMIC MODEL 31
4.2 EXPENDITURE ON NSPS 32
4.3 IMPACTS OF NSPS ON HIV AND HCV 33
4.4 METHODOLOGY FOR HEALTH CARE COSTINGS FOR HIV 36
4.5 METHODOLOGY FOR HEALTH CARE COSTINGS FOR HCV INFECTION 38
4.6 HIV TREATMENT COSTS 39
4.7 RETURN ON INVESTMENT 42
4.8 SENSITIVITY ANALYSIS 44
4.9 DISCUSSION 45
5 QUALITY OF LIFE (QOL) EFFECTS OF NSPS 47
5.1 INTRODUCTION 47
5.2 QUALITY-ADJUSTED LIFE-YEARS (QALYS) 48

5.3 METHODOLOGY FOR ESTIMATING THE QALYS GAINED FROM THE PREVENTION OF HIV AND HCV INFECTIONS 48
5.4 NUMBER OF CASES OF HIV AND HCV AVOIDED 50
5.5 NUMBER OF LIFE YEARS GAINED 50
5.6 QUALITY ADJUSTED LIFE YEARS GAINED 52
5.7 SENSITIVITY ANALYSIS 55
5.8 DISCUSSION 56

APPENDICES

APPENDIX A OVERVIEW OF STATE AND TERRITORY NEEDLE AND SYRINGE PROGRAMS 57
APPENDIX B DISCUSSION OF THE METHODOLOGY USED IN THE ECOLOGICAL STUDY 73
APPENDIX C DETAILED TABLES OF EFFECTS OF NSPS ON HIV AND HCV 75
APPENDIX D DETAILED TABLES ON FINANCIAL EFFECTS AND NPV OF NSPS 105
APPENDIX E DETAILED TABLES ON QUALITY OF LIFE EFFECTS OF NSPS 125
APPENDIX F REFERENCES 145

EXECUTIVE SUMMARY

In 2000, the Department of Health and Ageing engaged Health Outcomes International Pty Ltd (HOI) in association with the National Centre for HIV Epidemiology and Clinical Research (NCHECR) to undertake a study into the economic effectiveness (or return on investment) of needle and syringe programs (NSPs) in Australia.

The study updates and expands a study previously undertaken by Hurley, Jolley and Kaldor which investigated the effectiveness and cost effectiveness of needle and syringe programs in relation to HIV/AIDS (see 'The effectiveness and cost-effectiveness of needle and syringe exchange programs' in *An Economic Evaluation of Aspects of the Australian HIV/AIDS Strategie*s, Technical Appendix 2 to *Valuing the past…investing in the future - Evaluation of the National HIV/AIDS Strategy 1993-94 to 1995-96)*.

The study seeks to analyse the effectiveness of needle and syringe programs in preventing transmission of HIV, and hepatitis C (HCV) in Australia from 1991 (that is from when NSPs were well established in all jurisdictions except Tasmania) to the end of 2000. The study then uses these findings to calculate the return on investment from NSPs from 1991 to 2000.

EFFECTIVENESS OF NSPS FOR PREVENTING TRANSMISSION OF HIV AND HCV INFECTION

In this study, NCHECR repeated the ecological study of change in HIV prevalence in cities with and without NSPs because several countries have introduced NSPs since the previous study (Hurley et al. 1997). The study also used a similar methodology to assess the effectiveness of NSPs for prevention of HCV infection.

The ecological study design was used to compare HIV and HCV infection among injecting drug users in countries with and without NSPs. Data recorded on HIV and HCV infection included both seroprevalence and seroincidence studies. NSPs were defined as programs distributing needles and syringes, either free or with minimal charge, irrespective of whether they operated from a fixed or mobile site, whether return of a used syringe was mandatory, or the range of other HIV and HCV prevention and treatment services provided.

For HIV, there were 778 calendar years of data from 103 cities with HIV seroprevalence measurements from more than one year and information on NSP implementation. Studies were from 67 cities without NSP, 23 cities that implemented NSP between the first and last study, and 13 cities that already had NSP when the studies were carried out.

The analysis found that cities that introduced NSPs had a mean annual 18.6% decrease in HIV seroprevalence, compared with a mean annual 8.1% increase in HIV seroprevalence in cities that had never introduced NSPs (mean difference –24.7% [95% CI: –43.8%, 0.5%], p=0.06). An analysis which weighted each city by one over the variance of the fitted regression line estimated the mean difference in annual rates of change in HIV-seroprevalence between cities with and without NSPs to be –32.7% [95% CI: -37.5% to -27.6%] p<0.001. In cities with an initial HIV prevalence less than 10% and with sero-surveys over a period of at least three years, the mean annual decrease in HIV prevalence was 4.0% in cities that introduced NSPs, compared with a mean annual 28.6% increase in cities without NSPs (mean difference –25.3% [95% CI: -50.8%, 13.3%], p=0.2). In these cities, the weighted analysis estimated the mean difference to be –18.4% [95% CI: -32.0% to –2.0%] p=0.030. Because the unweighted results are qualitatively very similar and, for all cities, the point estimate is smaller than the weighted analysis, estimates of NSP effectiveness were based on the unweighted analysis, representing a more conservative approach.

For HCV, there were 190 calendar years of HCV seroprevalence data from 101 cities. Data were from 41 cities without NSP, 9 cities that implemented NSP between the first and last study, and 51 cities that already had NSP when the studies were carried out.

Median HCV prevalence was 75% (range 24% to 96%) in studies from cities without NSPs and 60% (range 17% to 98%) in cities with NSPs (NPtrend p=0.01). Overall the results indicated little change in HCV prevalence before

NSPs were introduced, followed by a decline after the introduction of NSPs. If HCV prevalence was 75% or 50% respectively before NSPs were introduced, the results correspond to around a 1.5% or 2% decline in HCV prevalence per annum.

The results of the analysis of the effect of NSPs on HIV and HCV prevalence internationally were then applied to estimates of the Australian injecting drug user population to estimate the number of cases of HIV and HCV avoided as a result of the activities of NSPs over ten years during the 1990s. The estimates are presented below.

Estimates of Injecting Drug Users Living with HIV/AIDS

- With NSP Introduction

The number of injecting drug users living with HIV/AIDS is estimated to have peaked in the early 1990s at approximately 470 cases, with a peak in people living with AIDS of less than 100 in the late 1990s. The cumulative number of deaths from HIV/AIDS by 2010 is projected to be approximately 350.

- Without NSP Introduction

The number of injecting drug users living with HIV/AIDS is estimated to peak in 2000 at approximately 26,000, with a peak in people living with AIDS of almost 3,000 in 2010. The estimated cumulative number of deaths from HIV/AIDS by 2010 is projected to be approximately 5,000.

- Prevented through NSP Introduction

By the year 2000, approximately 25,000 HIV infections are estimated to have been prevented among injecting drug users since the introduction of NSPs in 1988, and by 2010 approximately 4,500 deaths are projected to have been prevented.

Estimates of Injecting Drug users with HCV and HCV-related Deaths

- With NSP Introduction

In 2000, the number of injecting drug users living with HCV was estimated to be approximately 200,000 (approximately 150,000 with chronic HCV infection). By 2010 an estimated 11,800 injecting drug users are projected to be living with cirrhosis, and estimated cumulative HCV-related deaths are projected to be 1,800.

- Without NSP Introduction

In 2000, the number of injecting drug users living with HCV is estimated to be approximately 220,000 (approximately 165,000 with chronic HCV infection). By 2010 an estimated 12,500 injecting drug users are projected to be living with cirrhosis, and estimated cumulative HCV-related deaths are projected to be 1,900.

- Prevented through NSP Introduction

By the year 2000, approximately 21,000 HCV infections are estimated to have been prevented among injecting drug users since the introduction of NSPs in 1988, (of which approximately 16,000 would have developed chronic HCV); while by 2010 approximately 650 fewer injecting drug users are projected to be living with cirrhosis and 90 HCV-related deaths would have been prevented.

Financial Effects of NSPs

Expenditure on NSPs

Between 1991 and 2000, an estimated $141 million ($150 million in 2000 prices) was expended on NSPs across Australia, comprised of $122 million (87%) by government, and $19 million (13%) in consumer expenditure.

These data cover expenditure on NSPs operating within the programs managed by State and Territory health authorities. It excludes costs associated with the many retail pharmacies that also sell needles and syringes on a commercial basis, for which reliable data is not available on the number of needles sold or the level of expenditure by consumers.

TREATMENT COSTS AVOIDED

Estimates of the lifetime costs of treatment for HIV and HCV cases avoided are based on past and current treatment regimes by disease stage and applied over the projected lifetime of cases. Standardised costs have been used for each component of health care using year 2000 prices.

- HIV

For HIV, annual treatment costs are estimated to rise progressively to the year 2008 as patients progress to later stages of the disease, at which time they peak at approximately $269 million. Thereafter, annual costs decline, brought about mainly by the declining number of patients in the second and third stages of HIV. Total HIV treatment costs avoided over the lifetime of cases are estimated at $7,025 million (undiscounted). These represent the savings that accrue from a combination of the following:

- Approximately 25,000 cases of HIV avoided, who
- live for an average of about 24 years after infection, and who
- incur average treatment costs of nearly $14,000 each year of their life after diagnosis.

- HCV

For HCV, annual treatment costs rise progressively to the year 2040, at which time they peak at approximately $18.8 million and decline thereafter. The major factor influencing this cost profile is the number of patients who progress to liver failure who, while relatively small in number, have extremely high costs of treatment. Total HCV treatment costs avoided over the lifetime of cases are estimated at $783 million (undiscounted).

Overall, total treatment costs avoided over the life of the cases of HIV and HCV avoided by NSPs are approximately $7,808 million (before discounting). The costs of HIV treatment avoided are approximately ten times those for HCV, which reflects a combination of the number of cases avoided in the first instance (25,000 for HIV compared to 21,000 for HCV), a higher diagnosis rate for HIV than HCV, and higher average annual treatment costs for HIV than for HCV.

FINANCIAL RETURN ON INVESTMENT

The calculation of financial return on investment discounts future cashflows associated with the investment in the NSP program and treatment costs avoided by an agreed discount rate. The discount rate most commonly used in government programs of this nature is 5% per annum. For the purposes of illustration, we have also applied discount rates of 3% and 0%.

- HIV IMPACTS

The results of the analysis of financial return on investment in NSPs to government and in total, having regard to the impacts on HIV alone, are presented in Table 1.

Table 1 Net Present Value of investment in NSPs for HIV.

Discount Rate	Net Present Value, 1991 ($million, Year 2000 Prices)	
	Govt Expenditure	All Expenditure
	Lifetime Costs of Treatment	
5%	$2,277	$2,262
3%	$3,415	$3,398
0%	$6,896	$6,876

The analysis indicates that there have been significant financial savings accruing to government from the investment in NSPs to date, and that these savings will continue to accrue into the future.

- HIV AND HCV IMPACTS COMBINED

The financial return on investment in NSPs to government and in total, having regard to the impacts on HIV and HCV combined, are presented in Table 2.

Table 2 Net Present Value of investment in NSPs for HIV and HCV combined.

Discount Rate	Net Present Value, 1991 ($million, Year 2000 Prices)	
	Govt Expenditure	All Expenditure
	Lifetime Costs of Treatment	
5%	$2,402	$2,386
3%	$3,653	$3,637
0%	$7,678	$7,658

The analysis indicates that the incorporation of HCV into the NPV calculations has further increased the savings accruing to government and in total.

In summary, the study indicates that the financial return on investment will exceed manyfold the original investment in NSPs, and that the original investment had been fully recouped and surpassed by the end of the investment period, before any future savings are taken into account. The investment in NSPs is justified by the effect on HIV alone, with the effect on HCV providing an additional financial benefit, albeit a smaller one than HIV. Sensitivity analysis on the main variables used in the analysis indicates that the results are robust under a range of alternative assumptions and scenarios.

QUALITY OF LIFE (QOL) EFFECTS OF NSPS

Since both HIV and HCV are potentially life-threatening conditions, one of the main benefits from averting infections is the prevention of premature mortality. In addition, significant quality of life benefits may also accrue from the avoidance of HIV and HCV. The most widely used approach for estimating quality of life benefits in economic evaluations is the quality-adjusted life-year (QALY). In this approach, states of health are assigned a health state preference or 'utility' value, on a scale including 1.0 (full health) and 0 (death). The amount of time an individual spends in a given health state is then multiplied by the health state preference value to calculate the quality-adjusted life-years (QALYs) gained. The main advantage of the QALY approach is that it provides one combined measure of the benefits of a program that both extends life and maintains quality of life.

LIFE YEARS GAINED

The number of life years gained provides a measure of the additional number of years by those persons who would otherwise have been infected with HIV and HCV, but for the effect of NSPs.

The effect of NSPs in terms of life years gained is much greater for HIV than for HCV. The 25,000 persons avoiding HIV are expected to gain an additional 588,000 life years (about 23 years each) than if they had contracted HIV. In comparison, the 21,000 persons avoiding HCV are expected to gain only about 1,200 life years over their lifetime. The difference in these outcomes is essentially due to the different mortality rates associated with each disease and their rate of progression through the various stages.

QUALITY ADJUSTED LIFE YEARS GAINED

The application of an adjustment factor to the number of life years gained to take account of the quality of life effects of these diseases leads to a measure referred to as Quality Adjusted Life Years (QALYs). QALYs gained incorporates both the quantity of life gained, and the quality of life gained by avoiding HIV and HCV.

The 25,000 persons avoiding HIV are expected to gain an additional 715,000 quality adjusted life years than if they had contracted the disease. In comparison, the 21,000 persons avoiding HCV are expected to gain about 120,000 quality adjusted life years over their lifetime. The difference between the two diseases is largely attributable to the greater effect of HIV on the “quantity” of life compared to HCV, rather than the “quality” effect.

Applying the same discount rates used in the financial analysis (viz 5%, 3% and 0%) to QALYs gained results in the figures shown in Table 3

Table 3 Net Present Value of QALYs gained for HIV and HCV

Discount Rate	Net Present Value, 1991 (QALYs)		
	HIV	HCV	HIV & HCV
5%	138,072	32,207	170,279
3%	248,364	50,041	298,406
0%	715,245	119,992	835,237

The analysis of the effects of HIV and HCV on both the quantity of life and the quality of life of persons with these diseases adds a further dimension to the assessment of the effect of NSPs among injecting drug users. The benefits demonstrated for consumers in terms of the number of lives saved, the number of life years gained, and the improved quality of life are additional to the direct financial benefits to governments previously identified.

Our analysis demonstrates that NSPs have contributed significantly to:

- ☐ The number of cases of HIV and HCV avoided;
- ☐ A reduction in the number of deaths from HIV, and to a lesser extent from HCV;
- ☐ An increase in the number of life years among injecting drug users, particularly from the avoidance of HIV; and
- ☐ An improvement in the quality of life among injecting drug users who would otherwise have contracted HIV or HCV.

Each of these outcomes should be considered over and above the direct financial benefits achieved from the investment in NSPs. It is clear that if we were to place a monetary value against any of these outcomes, the financial gains already demonstrated would be significantly increased.

CONCLUSION

The study into the effect of NSPs on HIV and HCV, and the consequent return on investment from these programs has reinforced the original findings by Hurley, Jolley and Kaldor. The results demonstrate that NSPs are effective in reducing the incidence of both diseases and that they represent an effective financial investment by government.

From a financial perspective, we have considered only the direct costs of treatment saved by the avoidance of HIV and HCV. Such an approach is inherently conservative, and it is likely that there are further financial benefits derived from the investment in NSPs not included in our findings. As such, the savings we have demonstrated, if anything, understate the total financial benefits to government and members of the community.

When considering the effect of NSPs on the lives of those immediately affected by their operation, namely injecting drug users, the study again demonstrates that NSPs have a positive impact. This has been measured in terms of avoidance of deaths, gains in the duration of life and improvements in the quality of life of injecting drug users. Such benefits are additional to the financial benefits demonstrated.

The study has considered the investment in NSPs during the 1990s, at which time we have assumed that the investment ceased. The consideration of effect has been limited to the future benefits accruing from the cases of HIV and HCV avoided during the investment period. The results demonstrate that, across all measures of effect used in the study, NSPs have yielded a significant public health benefit, and that continued investment is warranted from both a financial and human perspective.

1 INTRODUCTION

1.1 BACKGROUND

In 2000, the Department of Health and Ageing engaged Health Outcomes International Pty Ltd (HOI) in association with the National Centre for HIV Epidemiology and Clinical Research (NCHECR) to undertake a study into the economic effectiveness (or return on investment) of needle and syringe programs (NSPs) in Australia.

The study updates and expands a study previously undertaken by Hurley, Jolley and Kaldor which investigated the effectiveness and cost effectiveness of needle and syringe programs in relation to HIV/AIDS (see 'The effectiveness and cost-effectiveness of needle and syringe exchange programs' in *An Economic Evaluation of Aspects of the Australian HIV/AIDS Strategie*s, Technical Appendix 2 to *Valuing the past…investing in the future - Evaluation of the National HIV/AIDS Strategy 1993-94 to 1995-96)*.

This report is a joint production of Health Outcomes International Pty Ltd and the National Centre for HIV Epidemiology and Clinical Research (NCHECR) with support from Professor Michael Drummond, Centre of Health Economics, York University, UK.

1.2 OBJECTIVES

The study seeks to analyse the effectiveness of needle and syringe programs in preventing transmission of HIV, hepatitis C (HCV) and hepatitis B (HBV) in Australia from 1991 (that is from when NSPs were well established in all jurisdictions except Tasmania) to the most recent possible period. The study then uses these findings to calculate the return on investment from NSPs from 1991 to the present.

Specifically the aims of the study were to:

- Estimate the effectiveness of NSPs in relation to preventing transmission of HIV as well as hepatitis B and C;
- Calculate the return on investment in NSPs from 1991 to the present; and
- Provide contemporary research on the effectiveness and efficiency of the NSPs in order to assist stakeholders and governments to demonstrate the role of NSPs as a core population health activity, and to support further investment in NSPs if necessary.

For several reasons the project examined effectiveness in relation to prevention of HIV and hepatitis C, but not hepatitis B. First, epidemiological data were more readily accessible for HIV and hepatitis C, in particular in the Australian setting. For example, the NSP survey that is conducted each year tests injecting drug users for HIV and hepatitis C, but as yet does not include hepatitis B testing. Second, the vast majority (possibly greater than 95%) of injecting drug users exposed to hepatitis B do not develop chronic infection, and are therefore not at risk of major hepatitis B-related morbidity and mortality. Third, there is greater uncertainty in relation to the natural history of chronic hepatitis B.

The introduction of NSPs may have reduced incidence of hepatitis B among injecting drug users in Australia, particularly as uptake of hepatitis B vaccination is not optimal. However, it is felt that the cost savings through hepatitis B prevention would have been considerably lower than for either HIV or hepatitis C. The exclusion of hepatitis B from the analysis therefore represents a conservative approach, and may underestimate, to some extent, the total costs of treatment avoided.

1.3 METHODOLOGY

The study comprised two discrete stages. The first related to the development of an agreed methodology that examined the evidence base available to support the study, and from that evidence, to develop an approach that maximised the use of available data. This stage comprised three components:

- ☐ An international literature review that examined national and international research of relevance to the study. The review identified a body of evidence that could inform the project, promote development in specific areas and encourage debate among stakeholders on issues of interest. The literature review did not seek to examine the findings of the literature, but rather to simply identify whether or not there is a sufficient body of evidence available to support a study of this type. Particular topics of interest explored included: evaluations (economic and other) of NSPs internationally; studies into the incidence and prevalence of HIV, HBV and HCV; and quality of life studies for patients with chronic illnesses (particularly HIV and HCV).
- ☐ Consultations with Commonwealth, State and Territory representatives were undertaken to develop a profile of NSPs across Australia, and to determine the range, nature and duration of operational data (activity and costs) within each jurisdiction to be used in the study.
- ☐ Following the above, a methodology for the study was developed and provided to the study Advisory Committee for consideration and comment.

The second stage of the study was the implementation of the approved methodology, the outcomes of which are presented in this report. The key components of the methodology were:

- ☐ An ecological study of the effect of NSPs on HIV and HCV, based on the international literature together with a range of related information and data from within Australia.
- ☐ Collection of data on the costs of operating NSPs in all Australian jurisdictions.
- ☐ Collection of data on the lifetime costs of treatment of HIV and HCV in the current clinical environment.
- ☐ Determination of Quality of Life (QoL) values for persons with HIV and HCV.
- ☐ Development and application of an economic model to evaluate the return on investment in NSPs.
- ☐ Determination of the quality of life impacts of NSPs on HIV and HCV.
- ☐ Preparation of draft and final reports presenting our findings.

In applying the findings of the impact of NSPs on HIV and HCV in Australia, we have assumed that NSPs have had no effect on the size of the injecting drug user population (i.e. that NSPs do not increase drug use). Whilst acknowledging the debate that exists on this subject, the available evidence from Australia and overseas has not demonstrated that NSPs have resulted in an increase in drug use, and hence our assumption is reasonable (See Guydish et al (1993), Watters et al (1994), Wolk et al (1990) and Schoenbaum et al (1996)).

1.4 ACKNOWLEDGEMENT

Throughout the course of the study, a number of individuals and organisations across Australia have contributed information, data, advice and other forms of assistance to the researchers. Their contribution is gratefully acknowledged.

2 AN OVERVIEW OF NEEDLE AND SYRINGE PROGRAMS (NSPS)

The information presented in this section has been largely derived from the paper "Needle and Syringe Programs: a review of the evidence" published by the Australian National Council on AIDS, Hepatitis C and Related Diseases (ANCAHRD).

2.1 AUSTRALIA'S STRATEGIES ON HIV/AIDS AND HEPATITIS C.

The first National HIV/AIDS Strategy was launched in 1989. According to Professor Richard Feachem, from the World Bank, who oversaw the evaluation of the second National HIV/AIDS Strategy:

"The first National HIV/AIDS Strategy released by the Commonwealth Government in 1989 provided a framework for an integrated response to the HIV epidemic and a plan for action across a range of policy and program activities. Needle and Syringe Programs were a key component on the education and prevention strategy."[1]

Professor Feacham concluded: *'Needle and Syringe Exchange Programs must be a foundation of Australia's prevention efforts in a third Strategy and beyond'*. The third National HIV/AIDS Strategy (Partnerships in Practice: National HIV/AIDS Strategy 1996-97 to 1998-99) continued to support Needle and Syringe Programs as an important part of its prevention program for people who inject drugs.

The fourth National HIV/AIDS Strategy and the first National Hepatitis C Strategy, continue to support Needle and Syringe Programs as effective harm reduction interventions.

2.2 OVERVIEW OF NEEDLE AND SYRINGE PROGRAMS

Needle and Syringe Programs are a public health measure to reduce the spread of blood borne viral infections such as HIV and hepatitis C among injecting drug users. These Programs are supported by the National Drug Strategy's harm reduction framework. They provide a range of services that include provision of injecting equipment, education and information on reduction of drug-related harms, referral to drug treatment, medical care and legal and social services. Equipment provided includes needles and syringes, swabs, vials of sterile water and 'sharps bins' for the safe disposal of used injection equipment. The aim of providing sterile injecting equipment is to prevent the shared use of injecting equipment, which can lead to the transmission of blood borne viral infections. Staff also address the potential for transmission of infection via sexual contact by providing condoms and safer sex education. By engaging injecting drug users in health services, those who continue to use drugs are likely to incur less harm to themselves and society. They are also an important point for collection of used injecting equipment.

The first Australian Needle and Syringe Program began in Sydney in 1986 as a trial project. The testing of syringes returned to this Darlinghurst Program detected an increase in HIV prevalence, suggesting that HIV was spreading among clients. In the following year Needle and Syringe Programs became NSW Government policy. Other States and Territories followed soon after.

There are a number of different models of Needle and Syringe Programs operating in Australia that vary between different jurisdictions and sometimes by locality. Depending on the jurisdiction, the proportions of these that are government run and non-government run also vary. Furthermore, of the NSPs operating in the non-government sector, a number of these are 'peer-based' NSPs. Peer-based NSPs can be distinguished by the employment of past or current drug users in the development and provision of NSP services to networks of injecting drug users.

[1] Feachem, RGA. 1995. Valuing the past… Investing in the future. Evaluation of the National HIV/AIDS Strategy 1993-94 to 1995-96. AGPS, Canberra.

It is widely understood that peer-based services have had a significant and positive impact on the delivery and acceptability of NSPs to injecting drug users.

Broadly the following NSP service models exist throughout Australia:

Primary outlets are stand-alone agencies that are specifically established to provide injecting equipment, sometimes along with primary medical care. Staff provide these specific services in a non-judgmental manner and develop a rapport with individuals who are otherwise hard to reach.

Secondary outlets offer needle distribution or exchange as one of a range of other health or community services. Typical secondary outlets include hospital Accident and Emergency Departments and Community Health Centres.

Mobile services are distribution and exchange services provided by vehicle or on foot.

Outreach services have workers who move around from place to place to extend the reach of the service, often out of hours.

Vending machines dispense needle and syringe packs containing several 1ml syringes for a small fee. These machines are monitored and restocked by Needle and Syringe Program staff.

Needle and Syringe Programs tend to be located in relatively public places because they need to be accessible. Various government-sponsored pharmacy schemes operate throughout Australia. Generally the schemes provide 1ml syringes, which can either be purchased, or, in NSW, exchanged free on return of a pack with used syringes. In addition to those participating in the government-sponsored schemes, other pharmacies sell needles and syringes and other equipment used for injecting on a commercial basis.

Over 40 countries operate Needle and Syringe Programs including: Australia, Belgium, Brazil, Bulgaria, Canada, China, Croatia, Czech Republic, Denmark, Estonia, Finland, Germany, Greece, Hungary, India, Kazakhstan, Latvia, Luxembourg, Nepal, Netherlands, Norway, Philippines, Poland, Portugal, Slovak Republic, Salvador, Slovenia, Thailand, Ukraine, United Kingdom and the United States of America.

2.3 OPERATIONS OF NEEDLE AND SYRINGE PROGRAMS

While Needle and Syringe Programs operate in all Australian States and Territories, their type, level of activity and funding arrangements differ considerably between jurisdictions. As part of the current study, a profile on NSPs in each State and Territory was developed, in association with representatives from the respective State and Territory health authorities. These profiles are presented in Appendix A.

In addition, State and Territory health authorities were asked to provide details of the level of government expenditure and consumer fees paid for NSP services in recent years, together with estimates of the number of needles and syringes distributed. A summary of the data reported is presented in Table 2.1. It should be noted that in several instances, estimates have been imputed based on data provided by health authorities and the analysis of trends within each State/Territory.

The information presented in the table excludes expenditure on, and needles and syringes distributed through pharmacies that sell these products on a commercial basis and are separate from government-auspiced NSPs. Reliable data on these services are not available across all jurisdictions, and consequently they have been excluded from the analysis presented in this report. However, in order to test the possible effect of their inclusion in the financial analysis, sensitivity analysis presented in Section 4.8 considers the impact of higher levels of costs of operating NSPs without any increase in benefit.

Table 2.1 Expenditure and needles distributed by NSPs by State/Territory, 1999/2000 (1)

	Government Expenditure ($'000)	Consumer Expenditure ($'000)	Total Expenditure ($'000)	Needles Distributed (000)
ACT	$531	$8	$539	593
NSW	$9,827	$463	$10,290	11,566
NT	n.a.	-	n.a.	604[2]
Qld	$1,678	-	$1,678	5,300
SA	$787	$43	$830	3,018
Tas	$484	$138[2]	$622	1,381[2]
Vic	$4,767	-	$4,767	6,177
WA	$1,227	$2,349[2]	$3,576	3,209
**Total[1]**	**$19,673**	**$3,001**	**$22,674**	**31,848**

1 Data relates to government-auspiced NSPs only. Excludes expenditure on needle and syringes sold through pharmacies on a commercial basis.

2 Includes figures imputed from data provided by State/Territory health authorities.

3 THE IMPACT OF NSPS ON HIV AND HCV

3.1 EFFECTIVENESS OF NSPS FOR PREVENTING TRANSMISSION OF HIV AND HCV INFECTION

3.1.1 INTRODUCTION

Measures to prevent HIV infection among people who inject drugs generally focus on preventing blood contact during injection by reducing injection, promoting use of sterile equipment when injecting, or adopting safer injecting practices. Consequently, Needle and Syringe Programs (NSPs) are a key strategy for preventing transmission of HIV infection in several countries, including Australia. In other countries, implementation has been limited by uncertainty about their effectiveness.

Randomised trials of the effectiveness of NSP in preventing HIV transmission have not been conducted. Several observational studies have assessed the impact of NSPs on self-reported risk behaviours, in particular use of sterile syringes or re-use of one's own syringe (Drucker et al. 1998). A few studies have compared HIV incidence or HIV, HBV or HCV prevalence in participants and non-participants of NSPs (Bruneau et al. 1997; Des Jarlais et al. 1995; Hagan et al. 1999; van Ameijden et al. 1994). One study compared NSP implementation in countries with sustained low HIV prevalence to those with high HIV prevalence (Hurley et al. 1997). While another used an ecological study design to compare changes in HIV prevalence in cities with and without NSPs (Hurley et al. 1997). The data generally, but not always, show NSPs to be effective in preventing HIV transmission.

In contrast to HIV infection, prevalence of HCV infection among injecting drug users is universally high, regardless of whether the studies were done in cities with or without NSPs (MacDonald et al. 1996). It is likely that HCV prevalence was already very high among injecting drug users before NSPs were introduced. However, there are no studies that quantify the impact of NSPs on HCV infection. In this study, we have repeated the ecological study of change in HIV prevalence in cities with and without NSP because several countries have introduced NSP since the previous study (Hurley et al. 1997). We have also used a similar methodology to assess the effectiveness of NSP for prevention of HCV infection. A discussion on the rationale behind the approach adopted in this study is presented in Appendix B.

3.1.2 METHODS

The ecological study design was used to compare HIV and HCV infection among injecting drug users in countries with and without NSPs. Data recorded on HIV and HCV infection included both seroprevalence and seroincidence studies. NSPs were defined as programs distributing needles and syringes, either free or with minimal charge, irrespective of whether they operated from a fixed or mobile site, whether return of a used syringe was mandatory, or the range of other HIV and HCV prevention and treatment services provided.

Several sources were used to identify published reports of HIV and HCV prevalence and incidence among injecting drug users and implementation of NSPs. Three electronic databases that indexed relevant journals were searched from January 1984 to June 2001. Both Medline and Embase databases were used because each placed an emphasis on research from different continents, that is, North America and Europe respectively. The Current Contents database was also used because it included literature from Social Science and Psychology journals. Additional studies were obtained from country specific surveillance reports, the HIV/AIDS Surveillance Database (US Census Bureau & UNAIDS, 2000), relevant websites, and through review of the index of journals frequently cited in the electronic searches.

All studies with sample size of at least 50 were included. Cities with HIV prevalence studies were only included if HIV was measured among injecting drug users in two or more calendar years. Studies of HIV or HCV among incarcerated injecting drug users were excluded because very few countries provide NSP during imprisonment.

Studies reported in journals published in languages other than English were only included if sufficient information was provided in the abstract to determine whether the study was suitable for inclusion and all required data points were reported in the abstract. References used in the analysis are provided in Appendix F.

Number of injectors tested per calendar year, percentage with HIV and /or HCV, presence or absence of NSP, and recruitment site were recorded for all studies. If studies reported data aggregated for more than one calendar year, the mid-point of the study period was used as the survey date. Data were also recorded on HIV and HCV prevalence among new and young injectors where available. Studies of HCV incidence were included if they reported numbers of incident HCV infections and person-years of follow-up.

Analysis compared change in HIV and HCV prevalence between cities with and without NSPs at the time of the surveys. For HIV prevalence, city-specific change in prevalence was used in the analysis. For HCV prevalence, however, it was not possible to use city-specific change because relatively few cities had more than one estimate of prevalence.

For each city, the annual rate of change of HIV seroprevalence was estimated by fitting a regression line on a logit scale, with calendar years centred to 1990. The annual rate of change of HIV seroprevalence was also estimated using regressions weighting the comparison of cities with and without NSPs according to one over the variance of the regression estimator (Hurley et al. 1997). The effect of NSPs was assessed by comparing the annual rate of change in HIV seroprevalence in cities that had ever introduced NSPs with cities that had never introduced NSPs. Analyses of HIV seroprevalence were performed comparing all cities, and also in the subset of cities which had an initial HIV seroprevalence of less than 10%, and had results from at least three surveys available over at least three years. Analyses were repeated using regressions weighted according to survey sample size, and also excluding cities in developing countries.

A random effects regression model was used for analyses of HCV seroprevalence because few cities had data points before and after NSPs were introduced, and to allow appropriately for within and between city effects. The analysis model fits regression equations of the form:

$$\text{Logit(HCV prevalence)} = \zeta + \eta^{*}\text{(calendar year)} + \nu^{*}\text{(year since NSPs started)}$$

The parameter estimate for ν can then be directly interpreted as the modifying effect of NSPs on logit(HCV prevalence) levels per year. The effect of NSPs on HCV prevalence was estimated using all data from all cities, excluding studies that used blood stored since 1981, and for cities that introduced NSP between the first and last available study. A random effects regression model was also used to estimate the effect of NSPs on HCV prevalence using data available for people reporting less than three years of drug injection. Other regression models, such as ML random effects, and GEE, were also used on the sampled HCV prevalences and gave identical results (data not reported).

Two sets of analyses were performed to assess the effect of NSPs on HCV incidence. In the first set of analyses, random effects and GEE negative-binomial models were used to compare cohorts in cities with and without NSPs, allowing for within and between city effects in the analysis and for over-dispersion effects. In the second analysis, an overall incidence rate was calculated for each city by summing the numbers of incident infections and person-years of follow-up. Straightforward negative-binomial regression models were then used to compare cities with and without NSPs.

3.1.3 HIV Seroprevalence

There were 778 calendar years of data from 103 cities with HIV seroprevalence measurements from more than one year and information on NSP implementation. Studies were from 67 cities without NSP, 23 cities that implemented NSP between the first and last study, and 13 cities that already had NSP when the studies were carried out (Table 3.1.1).

HIV prevalence ranged from zero to 79% at the first data point for each city (median 18%), with 53 cities reporting first HIV prevalence 10% or less. Data were reported from 1978 to 1999. Studies with first HIV prevalence 10% or less were available from 23 cities without NSP, 19 cities that implemented NSP between the first and last study, and 13 cities that already had NSP when the studies were carried out

The fitted HIV prevalence regression lines are presented for those cities that had never introduced NSPs in Figure 3.1a, and for those cities that had ever introduced NSPs in Figure 3.1b. To illustrate the fitting procedure, the fitted regression lines and the reported HIV seroprevalence survey results are shown for two sites (Songkla Province, Thailand and Sydney, Australia) in Figures 3.1c and 3.1d respectively.

The overall comparison of annual rates of change of HIV seroprevalence in cities that never introduced NSPs with cities that did introduce NSPs are summarised in Table 3.1.2. Cities that introduced NSPs had a mean annual 18.6% decrease in HIV seroprevalence, compared with a mean annual 8.1% increase in HIV seroprevalence in cities that had never introduced NSPs (mean difference –24.7% [95% CI: –43.8%, 0.5%], p=0.06).

In cities with an initial HIV prevalence less than 10% and with sero-surveys over a period of at least three years, the mean annual decrease in HIV prevalence was 4.0% in cities that introduced NSPs, compared with a mean annual 28.6% increase in cities without NSPs (mean difference –25.3% [95% CI:-50.8%, 13.3%], p=0.2).

Variability of the point estimate was markedly reduced and statistical significance markedly increased when the analyses for all cities and cities with HIV prevalence less than 10% were weighted according to one over the regression estimate (The better fit implies a smaller variance, and therefore its reciprocal is larger, representing a larger weight). However, a disadvantage of the weighted analyses is that it tends to put much greater weight on the few cities in which the linear regression gives a very good fit to the available HIV seroprevalence estimates. For this reason, and because the unweighted results are qualitatively very similar and, for all cities, the point estimate is smaller than the weighted analysis, estimates of NSP effectiveness were based on the unweighted analysis.

3.1.4 HCV SEROPREVALENCE

There were 190 calendar years of HCV seroprevalence data from 101 cities. Data were from 41 cities without NSP, 9 cities that implemented NSP between the first and last study, and 51 cities that already had NSP when the studies were carried out (Table 3.1.3). There were 71 cities with data available for one calendar year, 13 cities with data for two calendar years and 17 cities with data for three or more calendar years. In the 30 cities with HCV seroprevalence data available for more than one year, 60% had already implemented NSPs before the first year of measurement and 30% introduced NSP between the first and last year of measurement.

Median HCV prevalence was 75% (range 24% to 96%) in studies from cities without NSP and 60% (range 17% to 98%) in cities with NSP (NPtrend p=0.01). Data were reported from 1973 to 2000 (Figure 3.2). HCV results from stored samples collected between 1973 and 1989 were reported by 21 cities. There were 44 cities with their first study carried out between 1990 and 1994 and 36 cities with their first study between 1995 and 1999.

Overall the results indicated little change in HCV prevalence before NSPs were introduced, followed by a decline after introduction of NSPs (Table 3.1.4). If HCV prevalence was 75% or 50% respectively before NSPs were introduced, the results correspond to around a 1.5% or 2% decline in HCV prevalence per annum.

Similar results were obtained when two studies based on samples from the 1970s and one from 1980 were excluded from analysis and when analysis was limited to nine cities that implemented NSP between the first and last study (Table 3.1.4). Other analyses, using different regression models, such as ML random effects, and GEE, gave similar results (data not presented).

3.1.5 HCV SEROPREVALENCE AMONG NEW INJECTORS

There were 48 studies, from 19 cities, with HCV seroprevalence estimated among people reporting less than three years of injecting drug use (Figure 3.3). Most studies were from nine Australian cities (n=35, 73%). There were also two studies from both Baltimore and New York, and one study each from Chicago, Dublin, Lille, Liverpool, Manipur, Padua, New Zealand (four cities combined), and Valencia.

Most studies were carried out in cities with NSPs (43 studies from 16 cities). Five studies were carried out in four cities without NSPs. Before and after NSP data were only available from one city. Studies were carried out between 1985 and 2000, with half since 1996 (Figure 3.3). Sample size ranged from 14 to 303, median 53.

Median HCV prevalence was substantially lower in cities with than without NSPs (19% vs 71%; Table 3.1.5). On average, HCV prevalence in cities with NSPs was 37% lower than in cities without NSPs using random effects regression modelling (mean (sd): 25% (+18%) vs. 66% (+15%), $p<0.001$; Table 3.1.6).

3.1.6 HCV INCIDENCE

HCV incidence was reported for 27 time periods from nine countries. All three studies in cities without NSP were from Italy (Naples, Padua and Rome) in early 1990. HCV incidence studies in cities with NSPs were from six Australian cities (nine data points), Amsterdam (four data points), Baltimore (three data points), Berlin (one data point), Czechoslovakia (one data point), Geneva (two data points), Malmo (one data point), New Zealand (one data point), and Seattle (one data point).

On average, HCV incidence was 25 per 100 person years in studies from cities without NSPs compared with 16 per 100 person years in studies from cities with NSPs (Table 3.1.7). Similar rates were obtained when HCV incidence was aggregated for each city (Table 3.1.9). Analyses consistently indicated a non-statistically significant protective effect for HCV incidence in cities with NSPs using random effects and GEE negative-binomial regression models for all data points and straightforward negative-binomial regression modelling for data aggregated by city (Table 3.1.8).

3.1.7 DISCUSSION

On average, HIV seroprevalence decreased in studies of injecting drug users in cities with NSPs whereas in studies from cities without NSPs, HIV seroprevalence increased. Seroprevalence of HCV also decreased annually in studies carried out after NSPs were introduced. HCV prevalence was substantially lower among people reporting less than three years of drug injection in cities with NSPs compared to cities without NSPs. There was also a non-statistically significant protective effect for HCV incidence in cities with NSPs when compared to those without NSPs.

There are several limitations associated with the ecological study design that should be considered when interpreting the findings from these studies. Seroprevalence data used in the analyses were collected according to different protocols and in diverse populations. It is unlikely that estimates of HIV and HCV seroprevalence in cities with NSPs would differ systematically from those in cities without NSPs, so any such sampling bias would underestimate the effectiveness of NSPs. Because cities were selected for analysis by the existence of published HIV and HCV serological surveys, bias may have been introduced by the decision to do a survey in a particular city at a particular time.

Data on NSPs used in the analyses were based on presence or absence of NSPs rather than on the extent and uptake of these services. Given the positive findings, however, it is likely that inclusion of these parameters would result in a dose response effect on HIV and HCV seroprevalence from NSPs. In addition, it is not possible to separate the effects of implementation of NSPs from the other HIV prevention strategies (Benedikt et al. 2000). In most settings, introduction of NSPs is one component of a broader harm reduction package to reduce the risk of transmission of blood-borne viruses and other harm associated with injecting drug use. Other components include education and counselling, drug dependency treatment strategies such as methadone maintenance

therapy, and provision of clean injecting equipment through other outlets in particular pharmacies. Adequate data was not available on individual components of harm reduction strategies to allow an evaluation of the impact of components other than provision of clean injecting equipment (NSPs). Sensitivity analysis has been conducted to determine the outcome of lower rates of NSP effect on HIV (See Section 4.8).

The excess risk of HIV in people who inject drugs is not due solely to sharing needles, other injecting practices and sexual behaviour patterns increase HIV risk. In contrast to HIV, HCV infection is rarely spread through sexual transmission (MacDonald et al. 1996).

It is also possible that HIV seroprevalence may have remained low in some of the cities with NSPs, irrespective of their introduction. HCV infection, however, is universally high among drug injectors. In most countries HCV infection became endemic among this population before there was widespread publicity about transmission of blood borne viruses through injecting practices. Because HCV infection remains asymptomatic for longer than HIV infection, it is also possible that people with HCV infection remain in the population of injectors for longer than those with HIV infection, therefore increasing the prevalence of HCV infection in seroprevalence surveys of injectors.

If NSPs decrease the incidence of HIV and HCV, the rate of increase in seroprevalence should decrease, although the seroprevalence itself may not decrease, at least initially. It is likely that the lower effect of NSP on HCV than HIV seroprevalence can be attributed to the generally higher prevalence of HCV compared to HIV before the introduction of NSPs.

NSPs influence HIV and HCV transmission by increasing use of sterile syringes for injection and lowering the rate of syringe sharing thereby reducing contact with each virus. Some NSPs also provide referrals to drug treatment centres, condoms and education about minimising risk. The difference in rate of change of HIV seroprevalence between cities with and without NSPs and the decrease in HCV prevalence in cities after the introduction of NSPs may not be due solely to NSPs. Nonetheless, the study provides evidence that NSPs reduce the spread of HIV and HCV infection.

Table 3.1.1 Location of cities and sites of recruitment for cities with at least two HIV prevalence studies according to NSP status from the time of first to last study

Location of studies		No. cities without NSP	No. cities with & without NSP	No. cities with NSP
Asia	China	3	0	0
	India	1	1	0
	Malaysia	4	0	0
	Myanmar	4	0	0
	Nepal	0	0	1
	Thailand	22	2	0
	Vietnam	0	1	0
Australia		0	2	8
Canada		0	3	0
Europe	Austria	0	1	0
	Czech Republic	1	0	0
	Denmark	0	1	0
	France	0	0	1
	Germany	0	1	0

Location of studies		No. cities without NSP	No. cities with & without NSP	No. cities with NSP
	Greece	0	1	0
	Israel	1	0	0
	Italy	10	0	0
	Netherlands	0	0	2
	Spain	3	0	0
	Switzerland	0	1	0
South America	Argentina	0	1	0
	Brazil	5	0	0
United Kingdom		0	4	1
United States		13	4	0
**Total cities**		**67**	**23**	**13**
**Recruitment sites**				
Deceased		0	4	0
Detoxification/rehabilitation		226	18	0
Drug treatment agency		95	72	8
Entry to treatment		33	17	0
Field & snowball		16	25	7
Health service		0	2	0
HIV testing centre		6	17	0
Infectious diseases hospital		14	1	0
Multiple sites		15	61	6
NSP/pharmacy		0	27	35
Sexual health clinics		4	12	2
Other/not reported		26	25	3
**Total studies**		**435**	**281**	**61**

Table 3.1.2 Estimated annual rate of change in HIV seroprevalence according to weighting of analysis and sample selection for cities without and with NSPs

Weighting of analysis/ Sample selection	Cities without NSPs	Cities with NSPs
**No weighting of analysis**		
**All cities**		
Number	63	36
Mean	8.1%	-18.6%
(95% CI)	(-2.8%, 20.1%)	(-42.6%, 15.3%)
Mean difference (95%CI)	-24.7% (-43.8%, 0.5%), p=0.057	

Weighting of analysis/ Sample selection	Cities without NSPs	Cities with NSPs
**Cities with initial HIV prevalence <10%, three calendar years of data**		
Number	19	25
Mean	28.6%	-4.0%
95% CI	(-4.9%, 73.8%)	(-28.5%, 29.0%)
Mean difference (95%CI)	-25.3% (-50.8%, 13.3%), p=0.165	
**Weighting of analysis**		
**All cities**		
Number	63	36
Mean	5.1%	-29.2%
(95% CI)	(1.4%, 9.1%)	(-30.8%, -27.6%)
Mean difference (95%CI)	-32.7% (-37.5%, -27.6%), p=<0.001	
**Cities with initial HIV prevalence <10% and three calendar years of data**		
Number	19	25
Mean	32.1%	7.8%
95% CI	(22.1%, 42.8%)	(-4.8%, 22.0%)
Mean difference (95%CI)	-18.4% (-32.0%, -2.0%), p=0.030	

Table 3.1.3 Location of cities and sites of recruitment for cities with HCV prevalence studies according to NSP status from the time of first to last study

Location of studies		Number of cities without NSP	Number of cities without & with NSP	Number of cities with NSP
Asia	China	2	0	0
	Bangladesh	1	0	0
	India	0	0	1
	Japan	3	0	0
	Malaysia	1	0	0
	Nepal	0	0	1
	Taiwan	1	0	0
	Thailand	3	0	1
Australia		0	2	10
Canada		0	0	1
Europe	Austria	1	1	0
	Belgium	1	0	2
	Croatia	1	0	0

	Denmark	0	0	1
	France	1	0	4
	Germany	1	0	2
	Greece	1	0	0
	Hungary	1	0	0
	Iceland	1	0	0
	Israel	1	0	0
	Italy	7	0	1
	Luxembourg	0	0	1
	Netherlands	0	0	1
	Norway	1	0	0
	Poland	2	0	0
	Portugal	0	1	0
	Saudi	1	0	0
	Slovenia	0	0	1
	Spain	3	1	2
	Sweden	0	1	1
	Switzerland	0	1	1
New Zealand		0	0	5
South America	Argentina	0	0	1
	Brazil	2	0	0
United Kingdom		0	0	11
United States		5	2	4
**Total cities**		**41**	**9**	**51**
**Recruitment sites**				
Detoxification/rehabilitation		12	2	5
Drug treatment agency		14	9	10
Field & snowball		6	2	9
HIV testing /Sexual health centre		4	5	14
Multiple sites		2	5	15
NSP/pharmacy		0	11	37
Other		6	11	11
**Total studies**		**44**	**45**	**101**

Table 3.1.4 Estimation of the effect of NSPs on HCV prevalence per year using random effects regression

Inclusion criteria	logit(HCV)	Coefficient	Std. Error	p value	95% CI
**All cities and all data points**					
	Calendar year	-0.008	0.02	0.7	-0.05, 0.04
	Years since NSP	-0.079	0.03	0.003	-0.13, -0.02
	Constant	1.040	0.24	<0.001	0.56, 1.52
	sigma_u	0.5637			
	sigma_e	0.8082			
	rho	0.3275	(fraction of variance due to u_i)		
**All cities and excluding data points before 1981**					
	Calendar year	-0.0460	0.03	0.1	-0.10, 0.12
	Years since NSP	-0.0576	0.03	0.05	-0.11, -0.001
	Constant	92.775	59.3	0.1	-23.5, 209.1
	sigma_u	0.5627			
	sigma_e	0.8084			
	rho	0.3264	(fraction of variance due to u_i)		
**All nine cities with data points before and after NSP**					
	Calendar year	0.0446	0.04	0.2	-0.03, 0.11
	Years since NSP	-0.1317	0.05	0.01	-0.24, -0.03
	Constant	-87.17	70.8	0.2	-226, 51.6
	sigma_u	0.2255			
	sigma_e	0.8245			
	rho	0.0696	(fraction of variance due to u_i)		

Table 3.1.5 Summary of HCV prevalence rates among people reporting less than three years of drug injection according to availability of NSPs

NSP	Number of studies	Mean HCV prevalence	Standard deviation	Median HCV prevalence	Inter-quartile range
No NSP	5	66%	15%	71%	5%
With NSP	43	25%	18%	19%	21%

Table 3.1.6 Estimation of the effect of NSPs on HCV prevalence among people reporting less than three years of drug injection using random effects regression

HCV prevalence	Coefficient	Std. Error	p value	95% CI
NSP	-37.06	7.75	<0.001	-52.25, -21.86
Constant	64.50	8.41	<0.001	48.01, 80.98
sigma_u	22.74			
sigma_e	8.70			
rho	0.87	(fraction of variance due to u_i)		

Table 3.1.7 HCV incidence rates per 100 person years for cohorts according to availability of NSPs

NSP	Number of studies	Mean HCV incidence	Standard deviation	Median HCV prevalence	Inter-quartile range
No NSP	3	24.7/100py	16.9	28.6/100py	33.1
With NSP	24	16.4/100py	9.9	15.0/100py	10.6

Table 3.1.8 Comparison of HCV incidence in cohorts with and without NSP using negative binomial regression modeling

Type of model/ scnumber	IRR	Std. Error	p value	95% CI
**Random effects negative binomial model**				
NSP	0.55	0.25	0.18	0.23, 1.32
Total pyrs	(exposure)			
/ln_r	2.38	0.95		0.52. 4.24
/ln_s	3.45	1.17		1.16, 5.75
r	10.81	10.27		1.68, 69.53
s	31.62	37.01		3.19, 313.5
**GEE negative binomial model**				
NSP	0.69	0.47	0.58	0.19, 2.54
Total pyrs	(exposure)			

Table 3.1.9 HCV incidence rates per 100 person years for each city overall according to availability of NSPs

NSP	Number of studies	Mean HCV incidence	Standard deviation	Median HCV prevalence	Inter-quartile range
No NSP	3	24.7/100py	16.9	28.6/100py	33.1
With NSP	14	18.5/100py	11.4	15.9/100py	16.2

Table 3.1.10 Comparison of HCV incidence for each city with and without NSP using negative binomial regression modeling

Type of model/ scnumber	IRR	Std. Error	p value	95% CI
**Random effects negative binomial model**				
NSP	0.73	0.30	0.44	0.32, 1.64
Total pyrs	(exposure)			
/lnalpha	-1.28	0.45		-2.16. -0.40
alpha	0.28	0.13		0.12, 0.67

Figure 3.1a Fitted HIV prevalence in cities without NSPs.

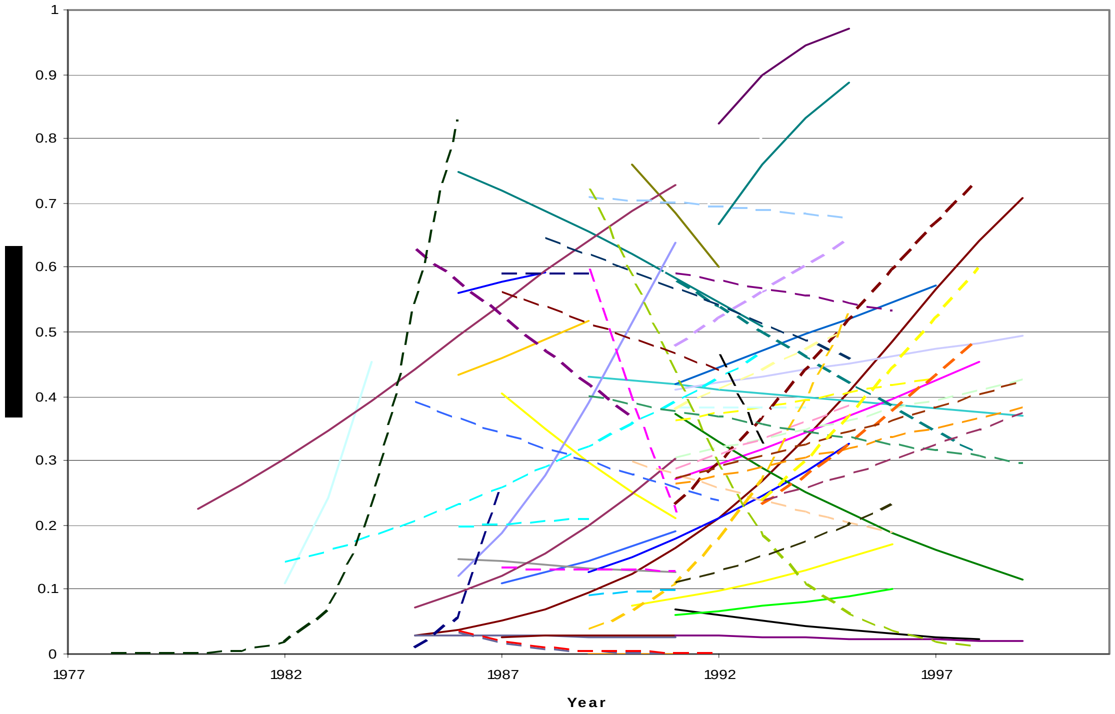

Figure 3.1b Fitted HIV prevalence in cities with NSPs.

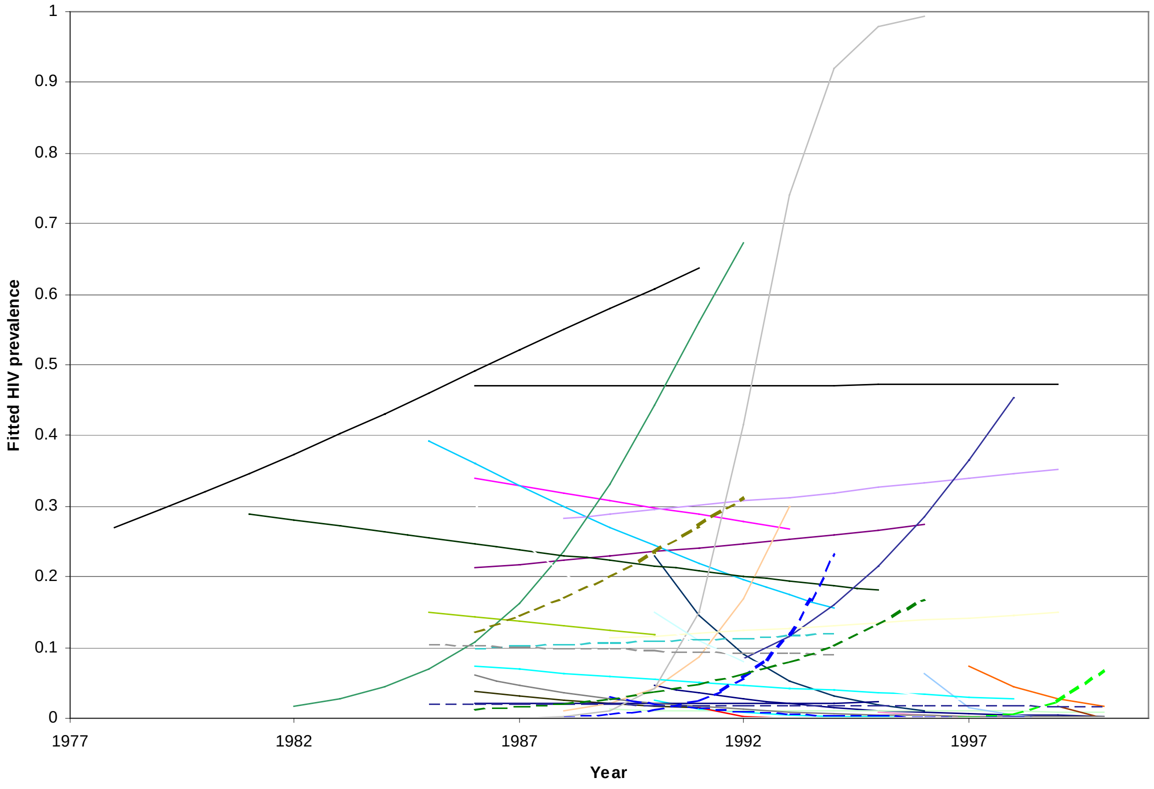

Figure 3.1c HIV seroprevalence in injecting drug users per year of survey for a city without NSP, Songkla Province, Thailand. (Lines represent fitted values from the logistic regression model)

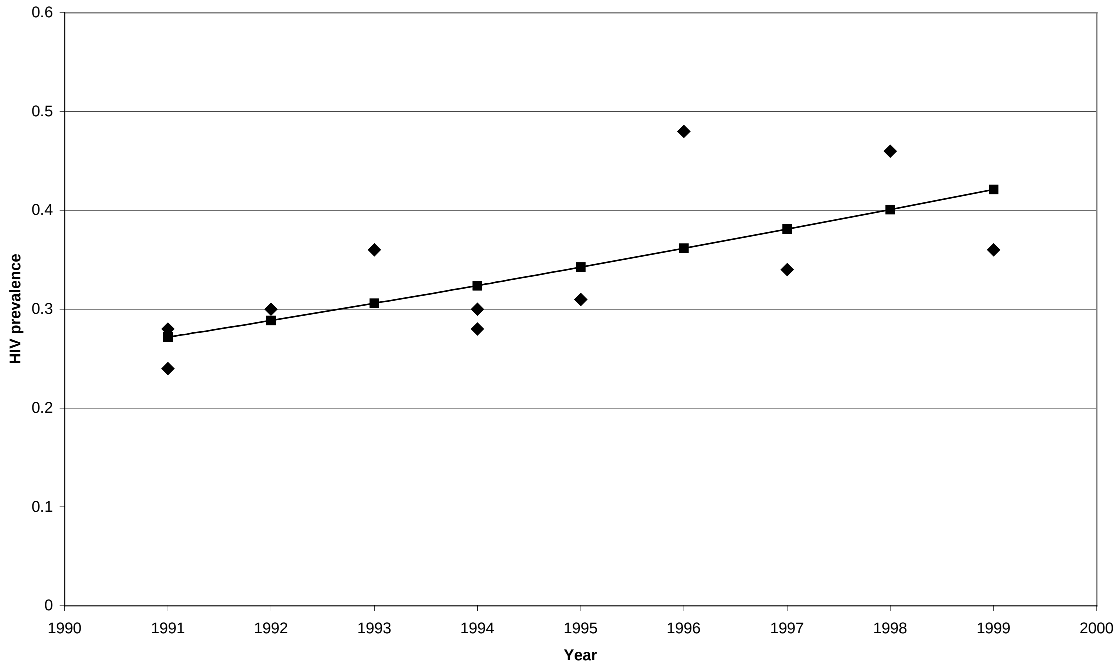

Figure 3.1d HIV seroprevalence in injecting drug users per year of survey for a city with NSP, Sydney, Australia. (Lines represent fitted values from the logistic regression model)

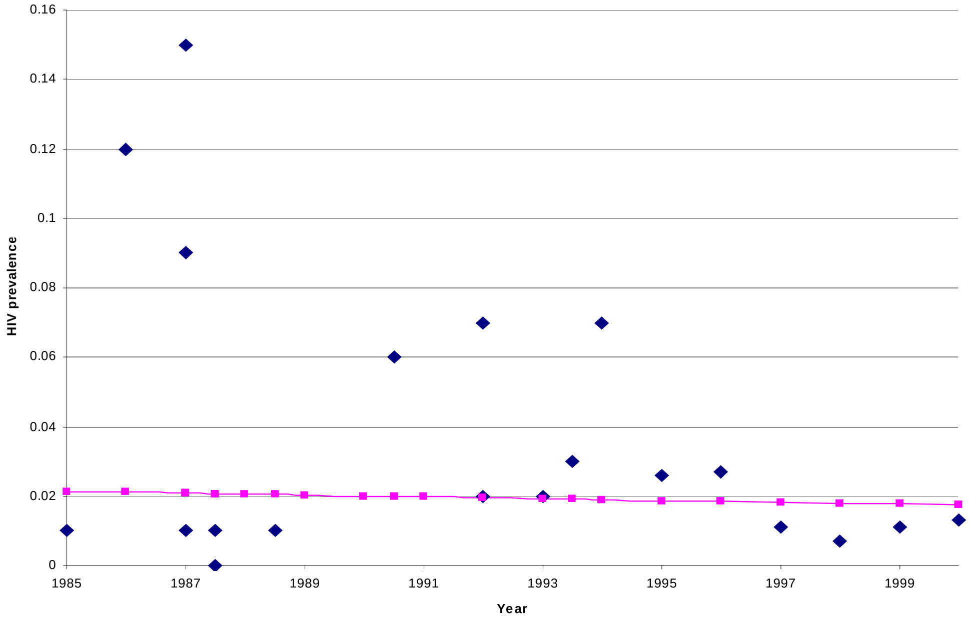

Figure 3.2 HCV seroprevalence among injecting drug users according to NSP status of city and year of study

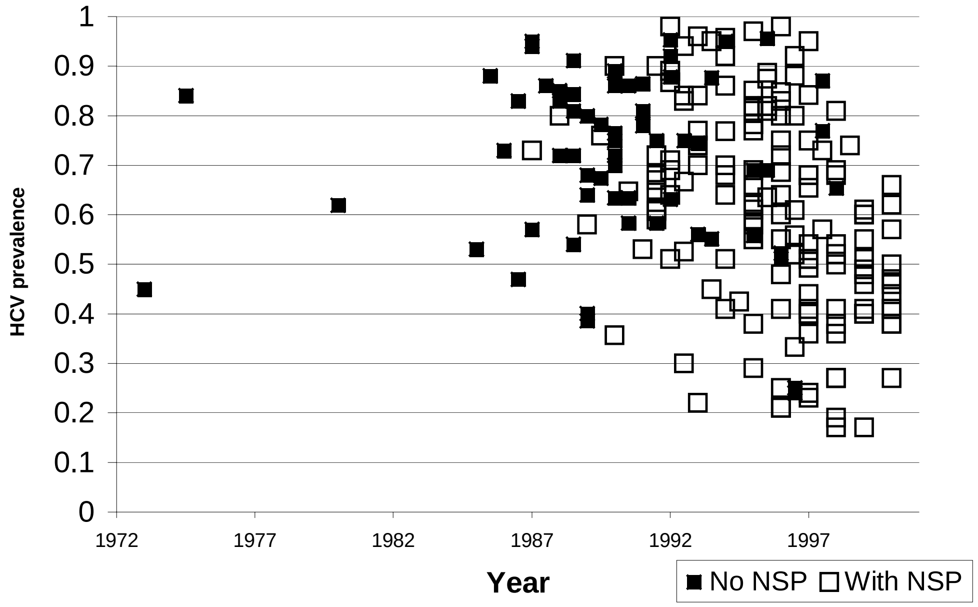

Figure 3.3 HCV seroprevalence among people reporting less than three years of drug injection according to NSP status of city and year of study

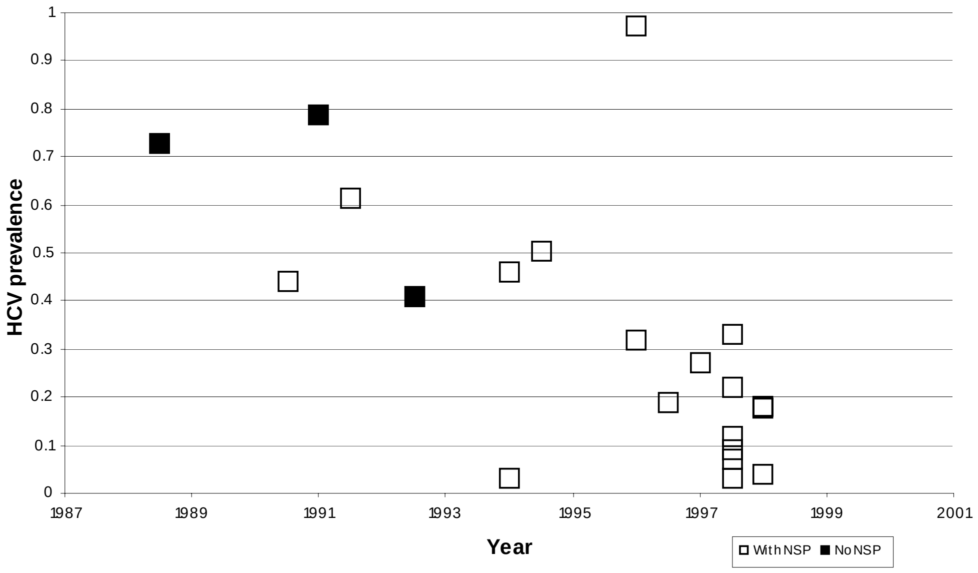

3.2 METHODOLOGY FOR ESTIMATES OF NUMBERS OF PEOPLE LIVING WITH HIV ACQUIRED THROUGH INJECTING DRUGS

3.2.1 ESTIMATES AND PROJECTIONS OF HIV/AIDS INCIDENCE

Estimates of past HIV incidence and future AIDS incidence as a result of injecting drug use were obtained using back-projection methods. The method uses observed AIDS incidence data (adjusted for reporting delay), and knowledge of the rate at which HIV infected people progress to AIDS, to reconstruct the likely pattern of past HIV incidence. It is then also possible to estimate future AIDS incidence. The form of back-projection used was that suggested by Becker et al (1991), as modified by Marschner and Watson (1992). Because of the relatively small numbers of AIDS cases reported due to injecting drug use, back-projection analyses were applied to annual AIDS counts.

The baseline rate of progression to AIDS was modelled using a Weibull-with-levelling distribution (Rosenberg et al. 1992), corresponding to a median time to AIDS of just under 10 years and a progression rate of 11.2% at four years (Alcabes et al. 1993). The extended definition of AIDS, adopted in Australia in January 1988, was assumed to result in a 10% increase in the rate of progression to AIDS (Rosenberg et al. 1992).

Because of the uncertainties surrounding both the effect of combination antiretroviral treatments in reducing the rate of progression to AIDS, and the numbers of people living with HIV infection taking up such treatments, back-projections were performed using the following methods. First, a back-projection based on AIDS cases diagnosed to the end of 1994 was performed to estimate the pattern of HIV incidence up to this time. Over this period only moderately effective antiretroviral treatments were available, assumed to correspond to an overall 10% reduction in the rate of progression to AIDS, so the pattern of past HIV incidence can be reliably reconstructed. Second, the effects of improved combination treatments since the beginning of 1995 were then estimated, based on the estimated pattern of HIV incidence, so as to closely approximate AIDS incidence observed between 1996 and 2000.

The effects of improved combination treatments on reducing the overall rate of progression to AIDS were estimated based on cases of AIDS reported due to injecting drug use, and are summarised in the Table 3.2.1 below.

Table 3.2.1 Estimated percentage effect of combination antiretroviral treatments in reducing the overall rate of progression to AIDS between 1995 and 1999

Year	1996	1997	1998	1999	2000
Estimated reduction in progression rate (%)	77	69	60	52	44

Projections of AIDS incidence from 2001 onwards were made by assuming that the effect of treatments on the rate of progression to AIDS continued at the year 2000 level.

In analyses HIV incidence was fixed at 20 cases per year from 1994 onwards. The level at which HIV incidence was fixed was decided on the basis of the number of HIV diagnoses and diagnoses of newly acquired HIV infection reported to the National HIV Database, and was also chosen to be consistent with the estimated HIV incidence obtained from the back-projection analyses.

Back-projection estimates of HIV incidence need to be adjusted for underreporting of AIDS diagnoses, and deaths prior to AIDS. Reporting of AIDS cases was thought to be relatively complete in Australia, with completeness estimated to be at least 95% (Grulich et al. 1999). Deaths among IDUs are estimated to be approximately 1% per annum (Thorley 1981; English et al. 1995). The median time to AIDS is thought to be just

under 10 years, so, taken together, HIV incidence was inflated by 15% to allow for underreporting of AIDS and deaths prior to AIDS.

3.2.2 Estimates of the Number of Injecting Drug Users Living with HIV Infection

Estimates of the number of IDUs living with HIV infection by disease stage (CD4+ cell count more than 500/σl, a CD4+ cell count of less than 500/σl and AIDS free, or living with AIDS) were based on the estimated pattern of past HIV incidence. The rate of progression to a CD4+ cell count fewer than 500/σl was modelled using a similar Weibull-with-levelling distribution to that used to model the time from HIV infection to AIDS. The median time from HIV infection to a CD4+ cell count of 500/σl was assumed to be 4 years, with 95% below 500/σl by 10 years. Survival following AIDS among IDUs in Australia was reasonably consistent between 1988 and 1995. The effect of combination antiretroviral treatment in improving survival following AIDS from 1996 was assumed to be similar to the effect of treatment in reducing the rate of progression to AIDS in Table 3.2.1, and to continue at the year 2000 rate from 2001 onwards. Background death rates were based on ABS life tables, assuming that the mean age at HIV seroconversion among IDUs was 30 years, and that there were 3 male HIV-infected IDUs for each female HIV-infected IDU (ABS 1995).

3.2.3 Estimating the Number of Injecting Drug Users Living with HIV without NSPs

The effect of needle and syringe programs (NSPs) in reducing HIV transmission among IDUs has been estimated to correspond to an annual reduction in (logit) HIV prevalence of 0.28 (see Section 3.1).

HIV prevalence among IDUs in Australia between 1980 and 2000 was based on the estimated numbers of IDUs living with HIV described above, and estimates of the numbers of IDUs in Australia.

Numbers of IDUs in Australia were estimated as follows. The number of dependent heroin users in Australia in 1997 was assumed to be 75,000 (Hall et al. 2000). A reasonable fit to available estimates over the previous two decades was obtained by assuming a constant net 8% increase in dependent heroin users per year. To allow for injecting of other drugs, the total number of regular IDUs was assumed to be 33% greater than the number of dependent heroin users (i.e. 100,000 regular IDUs in 1997 (Law 1999). The number of occasional IDUs was assumed to be 175,000 in 1997 (Law 1999) with the same annual percentage increases.

NSPs were first introduced in Australia in late 1987. Hence, NSPs were assumed to have reduced HIV prevalence among IDUs from 1988 onwards. The pattern of HIV prevalence if NSPs had not been introduced was estimated by increasing (logit) HIV prevalence by 0.28 per year from 1988 onwards. From this, a pattern of HIV incidence if NSPs had not been introduced was derived.

Estimates of the numbers of IDUs living with HIV by disease stage if NSPs had not been introduced were obtained by applying the same models described above regarding rates of progression from HIV infection to CD4+ cell count <500 cells/σl, to AIDS and survival before and following AIDS.

3.2.4 Estimated Effect of NSPs in Reducing Numbers of Injecting Drug Users with HIV by Disease Stage

To allow costing of the effect of NSPs in reducing the number of people living with HIV, estimates of the reduction in the number of people living with HIV by disease stage were obtained by subtracting the estimates obtained with NSPs from the corresponding estimates without NSPs. In these analyses, HIV incidence due to injecting drug use was assumed to cease from 2001 onwards, and estimates were projected forward until all people infected with HIV were estimated to have died.

3.3 METHODOLOGY FOR ESTIMATES OF NUMBERS OF PEOPLE LIVING WITH HCV ACQUIRED THROUGH INJECTING DRUGS

3.3.1 ASSUMPTIONS USED IN MODELS OF HCV INCIDENCE

The incidence of HCV in Australia was modelled based on the following assumptions regarding the past pattern of injecting drug use in Australia:

- There were 100,000 regular IDUs in 1997 (Hall et al. 2000), with a constant net increase of 8% per year since 1970, and with 5% stopping injecting each year (Thorley 1981; English et al. 1995).
- There were 175,000 occasional IDUs in 1997, with a constant net increase of 8% per year since 1970, and with 10% stopping injecting each year (Law 1999).
- There were no IDUs in 1960, with a linear increase in the number of both regular and occasional IDUs between 1960 and 1970.

Other assumptions made in modelling HCV incidence were the same as those adopted by the HCV Projections Working Group (Law 1999):

- 65% of IDUs who start injecting regularly have previously injected occasionally (from the Delphi study).
- The HCV incidence rate in uninfected regular IDUs was taken to be 18% per annum from 1960 until 1985, after which it was taken to decrease linearly to 13% in 1989 and thereafter.
- The HCV incidence rate in occasional IDUs was taken to be 20% of that in regular IDUs.
- All people starting or stopping injecting, or becoming regular rather than occasional IDUs, did so independent of their HCV status.
- HCV incidence due to receipt of infected blood or blood products was taken to be 15% of HCV incidence in IDUs until the early 1980s, after which it was assumed to have gradually decreased following the introduction of donor self-deferral related to injecting drugs (which began in 1983), and to be stopped entirely from 1990 onwards with the introduction of blood donor screening for HCV.
- HCV incidence through other transmission routes (such as needle stick injuries in health care workers, or tattoos) was taken to be 10% of HCV incidence in IDUs between 1987 and 1997, reflecting the data on risk factors for recent incident HCV infections. Prior to 1987 it was assumed to increase linearly to 20% of HCV incidence in IDUs in 1977, and then fixed at this absolute number of infections per year prior to this, again broadly consistent with data on risk factors for prevalent HCV infections, and for people with HCV infection attending liver clinics.
- The number of HCV infections between 1950 and 1960 was held constant at a low level proportional to the modelled HCV incidence among IDUs. Any HCV infections prior to 1950 were assumed to have negligible effect on estimates and projections, and were not modelled.

3.3.2 ESTIMATES OF RATES OF HCV-RELATED LIVER DISEASE PROGRESSION

It was assumed that 75% of people exposed to HCV developed HCV chronic infection (i.e. 25% of exposed people cleared HCV) (Law 1999). Of people with chronic HCV infection, it was assumed that one third had normal ALT values, one third abnormal ALT values, and one third abnormal ALT values with further covariates which indicate they would be at increased risk of progression (eg high alcohol intake). Rates of progression from stage 0/1 liver disease to stage 2/3 liver disease, and from stage 2/3 disease to cirrhosis are shown in Table 3.3.1.

Table 3.3.1 Annual rates of liver disease progression

Disease Stage	Stage 0/1 to Stage 2/3	Stage 2/3 to Cirrhosis
Not chronic HCV	0%	0%
Chronic HCV, normal ALT	1%	1%
Chronic HCV, abnormal ALT	2%	2%
Chronic HCV, normal ALT and further cofactors	3%	3%

Note: Stage 0=no hepatic fibrosis, stage 1=minimal hepatic fibrosis; stage 2=moderate hepatic fibrosis; stage 3=severe hepatic fibrosis; stage 4=cirrhosis.

Taken together, these assumptions combine so that of all people exposed to HCV, 5.3% are estimated to develop cirrhosis by 20 years, with 7.1% of people with chronic HCV developing cirrhosis by 20 years. This is consistent with current evidence regarding progression rates to cirrhosis (Freeman et al. 2001).

Rates of developing liver failure or hepatocellular carcinoma (HCC) from cirrhosis were assumed to be 4% and 1% respectively (Fattovich et al. 1997). It was further assumed that HCC could develop following liver failure, but not vice-versa. HCV-related mortality following cirrhosis was taken to be 1.5% per annum (Fattovich et al. 1997).

Mortality unrelated to HCV, both before and after cirrhosis, was assumed to be 1% per year (Thorley 1981; English et al. 1995) due to injecting drug use. Background mortality was based on ABS life tables, assuming that the mean age at HCV seroconversion among IDUs was 25 years, and that there were 2 male HCV-infected IDUs for each female HCV-infected IDU (ABS 1995).

Estimates of the numbers of people living with HCV by disease stage, and the incidence of liver cancer and HCC, were derived by combining these progression rates with the HCV incidence pattern estimated through the models described above.

3.3.3 Estimates of the Number of Injecting Drug Users Living with HCV without NSPs

The modelled estimate of HCV incidence in Australia that has occurred with NSPs described above corresponds to a gradual increase in HCV prevalence among regular IDUs until the mid- to late-1980s, followed by a gradual decline to around 52% HCV prevalence in 2000. NSPs were first introduced in Australia in late 1987. Hence, NSPs were assumed to have reduced HCV prevalence among IDUs from 1988 onwards. The pattern of HCV prevalence if NSPs had not been introduced was estimated by assuming that HCV prevalence would have remained constant at 1988 levels from 1988 onwards. From this, a pattern of HCV incidence if NSPs had not been introduced was derived. It was further assumed that the introduction of NSPs had no effect on HCV transmissions through routes other than injecting drug use.

Estimates of the numbers of people living with HCV by disease stage if NSPs had not been introduced were then derived using the same progression rate distributions described above.

3.3.4 Estimated Effect of NSPs in Reducing Numbers of Injecting Drug Users with HCV by Disease Stage

To allow costing of the effect of NSPs in reducing the number of IDUs living with HCV, estimates of the reduction in the number of people living with HCV by disease stage were obtained by subtracting the estimates obtained with NSPs from the corresponding estimates without NSPs. In these analyses, HCV incidence due to injecting drug use was assumed to cease from 2001 onwards, and estimates were projected forward until all people infected with HCV were estimated to have died, either from HCV-related or unrelated mortality.

3.4 NUMBER OF HIV INFECTIONS PREVENTED THROUGH THE INTRODUCTION OF NSPS

3.4.1 ESTIMATES OF INJECTING DRUG USERS LIVING WITH HIV/AIDS WITH NSP INTRODUCTION

Estimates and projections of the number of people living with HIV acquired through injecting drug use by disease stage and HIV/AIDS-related deaths from 1981 though 2070 are provided in Table 3.4.1 (See Appendix C). The number of people living with HIV/AIDS is estimated to have peaked in the early 1990s at approximately 470 cases, with a peak in people living with AIDS of less than 100 in the late 1990s. The cumulative number of deaths from HIV/AIDS by 2010 is projected to be approximately 350.

3.4.2 ESTIMATES OF INJECTING DRUG USERS LIVING WITH HIV/AIDS WITHOUT NSP INTRODUCTION

Corresponding estimates and projections of the number of people living with HIV/AIDS by disease stage and HIV/AIDS-related deaths without the introduction of NSPs are provided in Table 3.4.2 (See Appendix C). The number of people living with HIV/AIDS was estimated to peak in 2000 at approximately 26,000, with a peak in people living with AIDS of almost 3,000 in 2010. The estimated cumulative number of deaths from HIV/AIDS by 2010 was approximately 5,000.

3.4.3 ESTIMATES OF HIV INFECTIONS AND HIV/AIDS DEATHS PREVENTED THROUGH NSP INTRODUCTION

Estimates of the number of HIV infections and HIV/AIDS deaths prevented through the introduction of NSPs (over the period 1988 through 2000) are provided in Table 3.4.3 (See Appendix C). By the year 2000, approximately 25,000 HIV infections are estimated to have been prevented since the introduction of NSP in 1988, and by 2010 approximately 4,500 deaths are projected to have been prevented.

3.4.4 ESTIMATES OF HIV/AIDS CASES PREVENTED BY DISEASE STAGE AND DIAGNOSIS CATEGORY

An estimated 90% of the Australian HIV-infected population is diagnosed (NCHECR 2001). We have assumed that the proportion diagnosed is 100% for people with AIDS, and would be higher among people with progressive HIV disease (CD4<500/mm^3) than people with early HIV disease (CD4 >500/mm^3) (Table 3.4.4).

Based on data from the Australian HIV Observational Database (AHOD) over the period January 1997- March 2001, 71% of people with diagnosed HIV infection in Australia were receiving antiretroviral therapy. The proportion of injecting drug users receiving antiretroviral therapy was not significantly different to other risk categories (63% versus 71%, $p>0.05$). The proportion of people receiving antiretroviral therapy is 90% for AIDS, 50% for CD4 < 500/mm^3, and 69% for CD4 > 500/mm^3. Due to probable selection bias in AHOD for people with early HIV disease who are receiving antiretroviral therapy, the population level proportion is likely to be somewhat lower. Therefore, 40% of people with early stage HIV disease have been estimated to be receiving antiretroviral therapy.

Table 3.4.4 Proportions of people with diagnosed HIV and antiretroviral therapy use by disease stage

Disease stage	Diagnosed/Undiagnosed (%)	Antiretroviral therapy among diagnosed group[1]
Early HIV disease (CD4 >= 500/mm^3)	80/20	40%
Progressive HIV disease (CD4 < 500/mm^3)	90/10	70%
AIDS	100/0	90%

All people with undiagnosed HIV are assumed to not be receiving antiretroviral therapy.

Estimates of the number of HIV/AIDS cases prevented through the introduction of NSP by disease category and diagnosis category are provided in Table 3.4.5 (See Appendix C). These estimates form the basis for the calculation of the health care cost savings provided by the introduction of NSPs.

3.5 NUMBER OF HCV INFECTIONS PREVENTED THROUGH THE INTRODUCTION OF NSPS

3.5.1 ESTIMATES OF INJECTING DRUG USERS WITH HCV AND HCV-RELATED DEATHS WITH NSP INTRODUCTION

Estimates and projections of the number of people living with HCV acquired through injecting drug use by disease stage and HCV-related deaths from 1961 through to 2075 with the introduction of NSPs are provided in Table 3.5.1 (See Appendix C). In 2000, the number of people living with HCV is estimated to be approximately 200,000 (approximately 150,000 with chronic HCV infection). By 2010 an estimated 11,800 people are projected to be living with cirrhosis, and estimated cumulative HCV-related deaths are projected to be 1,800.

3.5.2 ESTIMATES OF INJECTING DRUG USERS WITH HCV AND HCV-RELATED DEATHS WITHOUT NSP INTRODUCTION

Corresponding estimates and projections of the number of people living with HCV by disease stage and HCV-related deaths without the introduction of NSPs are provided in Table 3.5.2 (See Appendix C). In 2000, the number of people living with HCV is estimated to be approximately 220,000 (approximately 165,000 with chronic HCV infection). By 2010 an estimated 12,500 people are projected to be living with cirrhosis, and estimated cumulative HCV-related deaths are projected to be 1,900.

3.5.3 ESTIMATES OF HCV INFECTIONS AND DEATHS PREVENTED THROUGH NSP INTRODUCTION

Estimates of the number of HCV infections and HCV-related deaths prevented through the introduction of NSPs (over the period 1988 through 2000) are provided in Table 3.5.3 (See Appendix C). By the year 2000, approximately 21,000 HCV infections are estimated to have been prevented since the introduction of NSP in 1988, (of which approximately 16,000 would have developed chronic HCV); while by 2010 approximately 650 fewer people are projected to be living with cirrhosis and 90 HCV-related deaths would have been prevented.

3.5.4 ESTIMATES OF HCV CASES PREVENTED BY DISEASE STAGE AND DIAGNOSIS CATEGORY

In Australia, an estimated 70% of people living with hepatitis C are diagnosed (NCHECR 2001). Table 3.5.4 outlines the estimates of diagnosed chronic hepatitis C by stage of liver disease. It was assumed that proportions of diagnosed chronic hepatitis C would increase with disease stage to reach 100% for advanced liver disease complications (HCC, liver failure).

Table 3.5.4 Proportions of people with diagnosed chronic hepatitis C at different disease stages

Disease stage	Diagnosed/Undiagnosed (%)
Mild chronic hepatitis C	60/40
Moderate chronic hepatitis C	75/25
Compensated cirrhosis	80/20
Liver failure	100/0
Hepatocellular carcinoma	100/0

Note: An estimated 70% (140,000/200,000) of people living with hepatitis C in Australia are aware of their HCV status (NCHECR 2001).

Estimates of the number of HCV cases prevented through the introduction of NSP by disease stage and diagnosis category are provided in Table 3.5.5 (See Appendix C). These estimates form the basis for the calculation of the health care cost savings provided by the introduction of NSPs. Although there may be quality of life impairment and health care costs for people who are HCV antibody positive but do not have chronic hepatitis C, we have taken the conservative approach of basing our analyses on cases of chronic hepatitis C only.

4 FINANCIAL EFFECTS OF NSPS

An economic model was developed that compares the costs of operating NSPs during the 1990s to the anticipated savings that will accrue from the number of cases of HIV and HCV avoided as a result of NSPs. This section describes the data sources and methodology used in the financial analysis.

4.1 DESCRIPTION OF THE ECONOMIC MODEL

4.1.1 OVERVIEW

The model used to analyse the financial effects of NSPs examines the direct costs of operating NSPs during the 1990s, and compares those costs to the future financial savings that are anticipated to flow from that investment. In this instance, these savings relate to the direct costs of treatment of cases of HIV and HCV that would otherwise have occurred until death, had it not been for the existence of NSPs. Because the investment in NSPs occurred over a ten year period, while the savings will continue to accrue into the future until all cases avoided have died, the cashflows associated with both are discounted back to a common reference point, namely the commencement of the investment period. The net value of these two cashflow streams after discounting, known as the Net Present Value, takes into account the fact that a dollar today is valued more highly than a dollar in, say, ten years, and thus converts them to a common dollar equivalent. The concept of discounting cashflows thus enables us to assess the current value of future costs and savings for any investment decision.

4.1.2 DIRECT AND INDIRECT COSTS AND BENEFITS

The issue of whether to include both direct and indirect costs and benefits was considered during the course of the design development. It was decided that in undertaking the analysis, only direct costs and benefits will be included.

- Direct costs include the costs of operating NSPs themselves, the infrastructure associated with their development and operation, and the costs of safe disposal of used syringes and needles. Conceptually, direct costs may also include the costs of volunteers and other unpaid workers in NSPs, and in-kind support provided by host agencies. However, the data reported by State and Territory health authorities were not able to identify or quantify this component, and it is therefore excluded from the analysis.
- Direct cost offsets or savings are those related to reduced costs due to the prevention or avoidance of HIV and HCV attributable to NSPs. These have been based on the lifetime costs of treatment of the diseases, and are discussed further below.

Indirect costs include productivity losses brought about by increased illness. Examples of indirect benefits include the value of increased productivity due to lives saved and extended employment. Typically, economic evaluations that have included indirect costs and benefits have demonstrated them to be many times the value of direct costs and benefits. In many cases, their inclusion has so overwhelmed the value of the direct benefits, that they have dominated the outcome. However, their measurement has often been the subject of considerable debate and criticism. This is particularly so when dealing with specific sub-populations, in this case injecting drug users.

At the same time, while the major focus of the study is on the public health perspective, it should be recognised that programs of this type may have implications for many other aspects of society that are not reflected in the economic analysis.

Given this history, together with the requirement that this study be based on a strong evidence base and be able to withstand close scrutiny, we have excluded indirect costs and benefits from the main economic analysis. However, it should be recognised that these costs and benefits exist, even if they are not quantified in the analysis.

4.2 EXPENDITURE ON NSPS

Data on the expenditure on operating NSPs in Australia during the 1990s was sought from all State and Territory health authorities by way of a standard survey instrument. A spreadsheet with explanatory comments and notes was provided to all authorities, with the request that they complete the fields as far as available data enabled.

Expenditure was identified under the following components:

- Overhead and infrastructure expenditure;
- Direct operating expenditure on public NSPs;
- Subsidies paid to community pharmacies; and
- Consumer expenditure.

All States and Territories provided the data sought, though to varying degrees of completion. In nearly all instances, data was provided for at least the last three to five years of the study period. Where data was not available, estimates were imputed for each component, based on trends within that component for the respective State/Territory. Table 4.2.1 illustrates the aggregate expenditure on NSPs across Australia for the period 1990/91 to 1999/2000, expressed in Year 2000 prices.

Table 4.2.1 Expenditure on NSPs, Australia, 1990-91 to 1999-2000 ($'000)(1)

1990-1991	1991-1992	1992-1993	1993-1994	1994-1995	1995-1996	1996-1997	1997-1998	1998-1999	1999-2000	Total
**Overhead and Infrastructure Costs**										
$441	$455	$530	$560	$541	$539	$714	$757	$841	$1,153	$6,531
**Direct Operating Expenditure on Public NSPs**										
$7,215	$7,730	$8,172	$8,710	$9,089	$10,251	$12,213	$13,250	$13,690	$15,243	$105,562
**Subsidies to Community Pharmacies**										
$826	$1,045	$1,129	$1,318	$1,497	$1,551	$2,079	$2,347	$2,975	$3,278	$18,045
**Consumer Costs**										
$1,091	$1,183	$1,608	$1,905	$1,865	$1,555	$2,043	$2,625	$2,930	$3,001	$19,807
**Total Government Direct Expenditure**										
$8,042	$8,774	$9,301	$10,028	$10,586	$11,802	$14,292	$15,597	$16,664	$18,521	$123,607
**Total Government Expenditure**										
$8,483	$9,230	$9,831	$10,589	$11,127	$12,341	$15,006	$16,354	$17,505	$19,673	$130,138
**Total Expenditure**										
$9,574	$10,413	$11,438	$12,494	$12,992	$13,897	$17,048	$18,979	$20,435	$22,674	$149,944

(1) Year 2000 Prices

Over the decade, a total of $150 million (Year 2000 prices) was expended on NSPs across Australia, comprised of $130 million (87%) by government, and $20 million (13%) in consumer expenditure. Overhead and infrastructure costs ($7 million) accounted for 5% of government expenditure, with direct operating costs of public NSPs ($106 million) accounting for a further 81%, and subsidies paid to community pharmacies for NSP services ($18 million) the remaining 14%.

After adjusting for inflation over the period, total expenditure on NSPs increased by 2.3 times over the ten years. Figure 4.1 illustrates the growth in real expenditure of the various components over the ten-year period.

Figure 4.1 Expenditure on NSPs, 1990-91 to 1999-2000 (Year 2000 Prices)

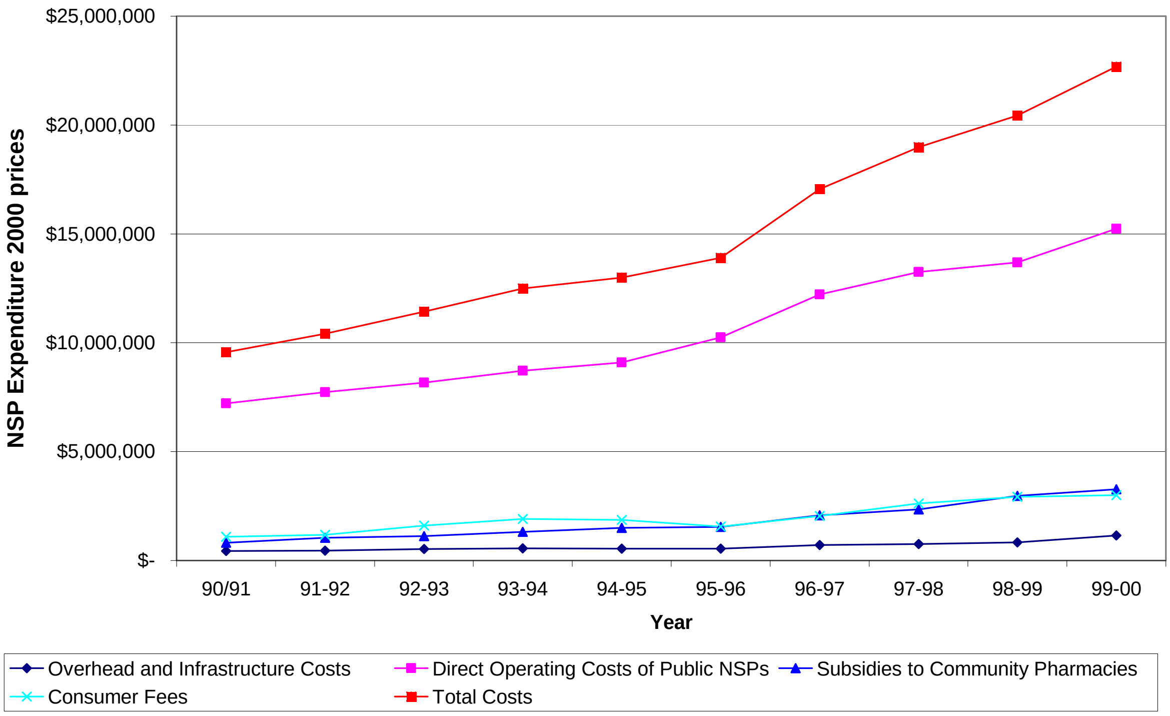

It should be noted that the data presented above covers expenditure on NSPs operating within the programs managed by State and Territory health authorities. Many retail pharmacies also sell needles and syringes on a commercial basis, for which reliable data is not available on the number of needles sold or the level of expenditure by consumers. The relative activity of the retail market in this area varies considerably between States (eg in Queensland, an estimated 5 million needles are distributed through the retail market). Costs of needle and syringes bought through the retail market are borne by consumers rather than through government subsidy. Consequently the total investment by consumers in needle and syringes may be understated in the analysis. For the purposes of illustration, sensitivity analysis has been conducted to assess the impact of doubling the above expenditure figures, and is presented in Section 4.8.

4.3 IMPACTS OF NSPS ON HIV AND HCV

The impact of NSPs on HIV and HCV is presented in Section 3, and was prepared by the National Centre in HIV Epidemiology and Clinical Research, The University of New South Wales. Estimates of the number of HIV and HCV infections avoided through the introduction of NSPs by stage of disease are discussed in Sections 3.4.4 and 3.5.4, with detailed figures for HIV and HCV contained in Tables 3.4.5 and 3.5.5 respectively (See Appendix C).

Figures 4.2 and 4.3 illustrate the estimated number of HIV and HCV cases with and without NSPs and the number of cases avoided, until all cases avoided have died.

In both instances, the figures illustrate that the total number of cases avoided accumulates up to the end of 2000, the end of the NSP investment period, then progressively decline as they progress through the various disease stages and mortality takes effect. The difference in scales of the two figures should be noted, reflecting the higher prevalence of HCV among IDUs.

Figure 4.2 HIV cases with, without and avoided by NSPs

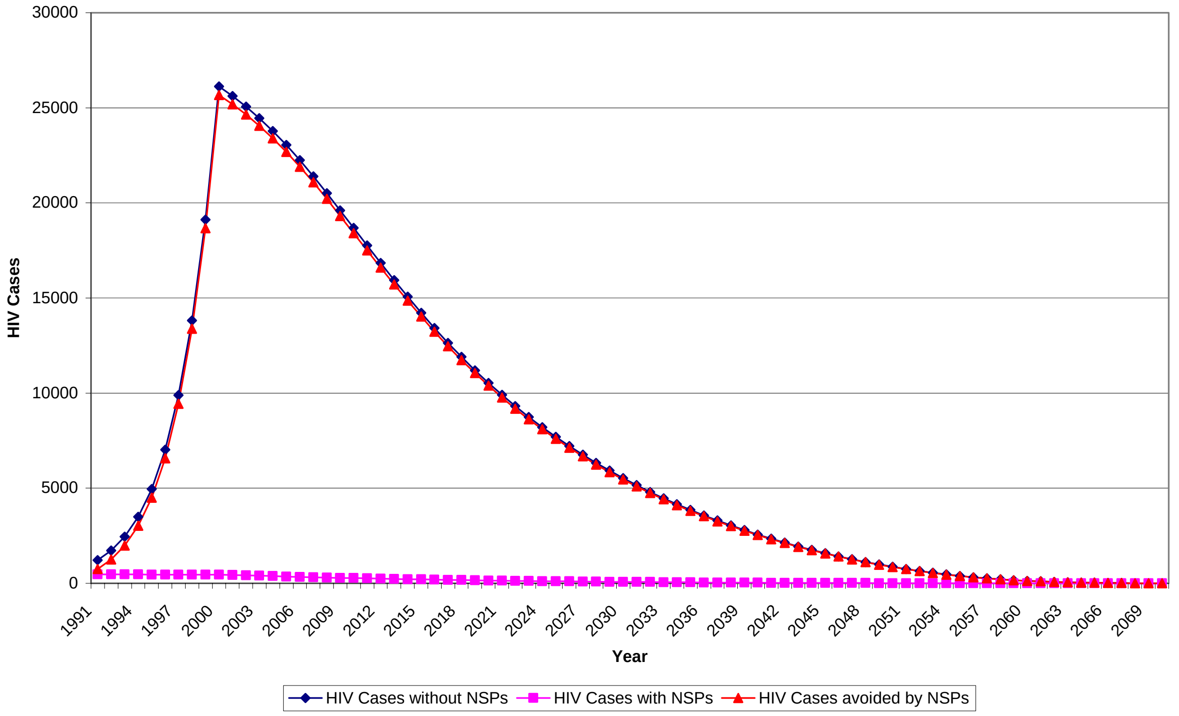

Figure 4.3 HCV cases with, without and avoided by NSPs

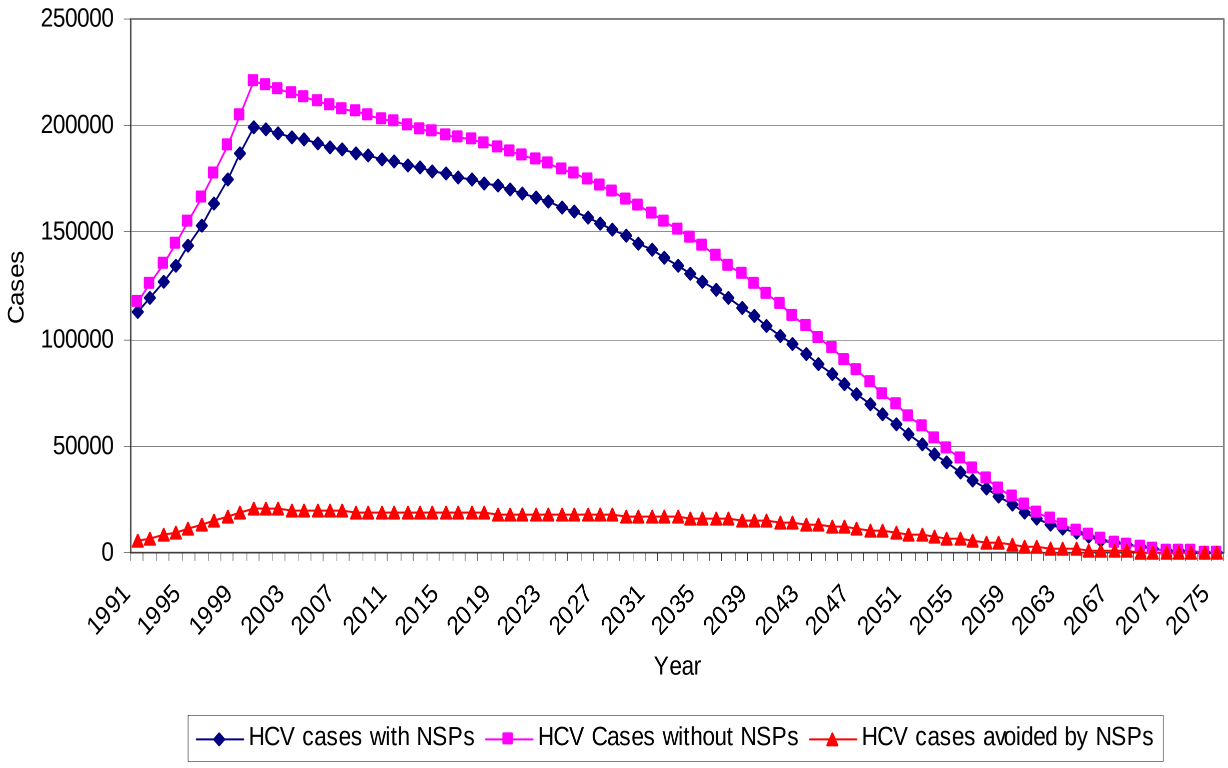

Figures 4.4 and 4.5 illustrate the stages of disease in HIV and HCV for the cases avoided by NSPs.

Figure 4.4 HIV cases avoided by stage of disease

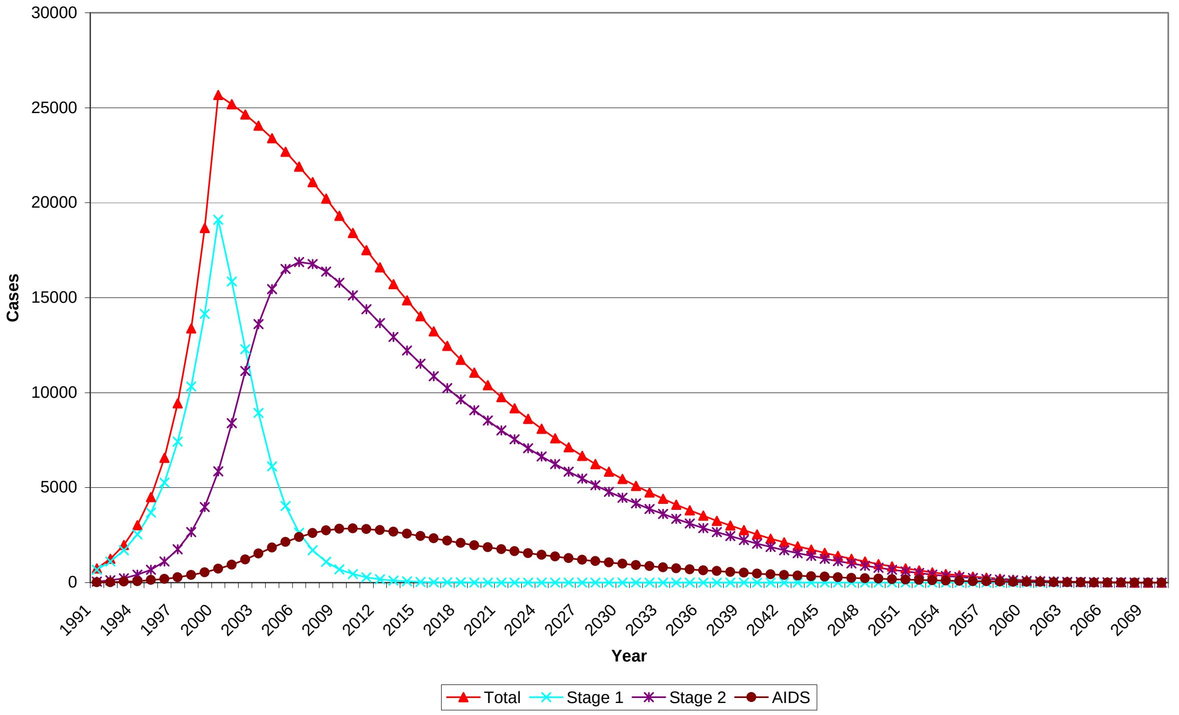

Figure 4.5 HCV cases avoided by stage of disease

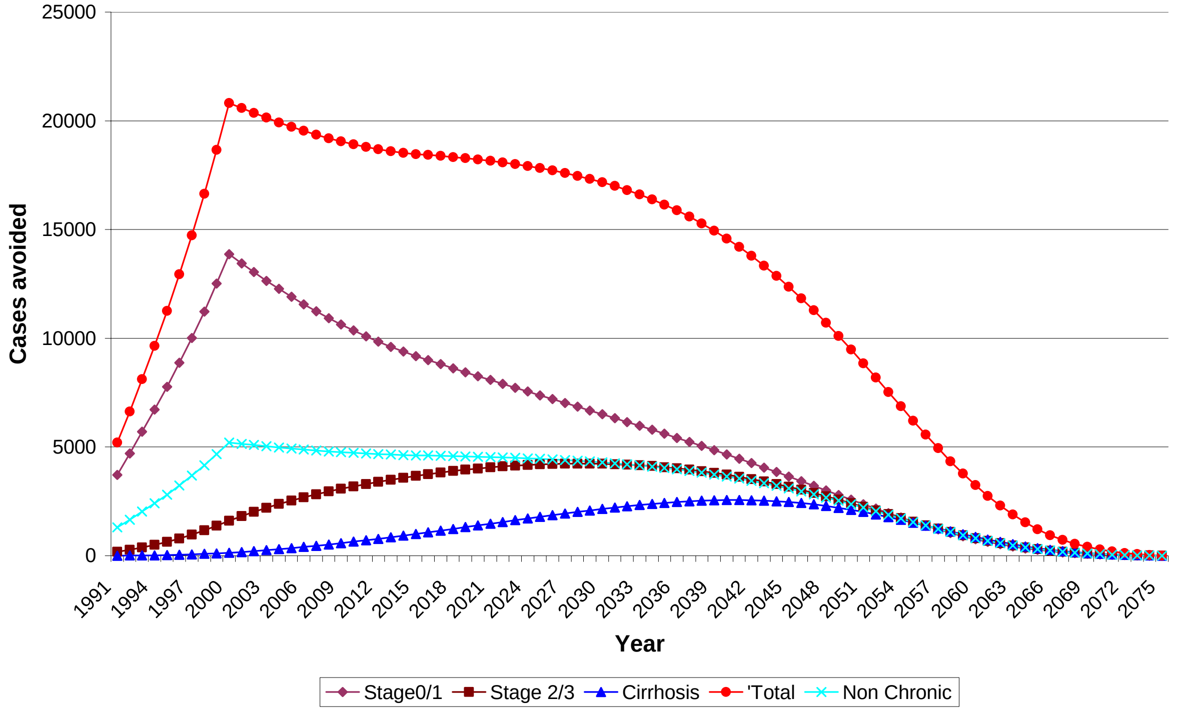

The figures illustrate the rate of progression of the two diseases, and the proportional distribution of the various stages of each disease over time, with later stages of the disease gaining greater prominence over time.

These data were applied in the economic model, with treatment costs included only for those patients diagnosed with the disease, as discussed in Sections 3.4 and 3.5. To the extent that some consumers not diagnosed with the disease also incur costs of treatment, the exclusion of these consumers from the model represents a conservative approach (i.e. the costs of treatment avoided may be understated).

4.4 METHODOLOGY FOR HEALTH CARE COSTINGS FOR HIV

Initially, a review of national and international literature relating to quality of life assessment and health care for HIV/AIDS was undertaken. Additional information on health care utilisation for HIV/AIDS was sought from various databases and sources, including:

- The Australian HIV Observational Database - data on antiretroviral therapy uptake by risk group and stage of disease, and utilisation of specific HIV investigations (CD4 count and HIV viral load)
- The Highly Specialised Drugs Program - data on use and costs of antiretroviral therapy in Australia

The following assumptions were employed in determination of health care costs for HIV/AIDS:

- All people who acquire HIV infection are at risk of progression to advanced HIV disease.
- The health care costs of acute HIV are small, due to the often asymptomatic nature of newly acquired HIV infection, and therefore were not considered in the total costs.

4.4.1 COSTS OF ANTIRETROVIRAL THERAPY

Data on antiretroviral therapy use and costs in Australia was obtained from the Highly Specialised Drug Scheme (NCHECR 2001). The per person cost of antiretroviral therapy increased significantly from 1996 (AUD$4,385) to 1997 (AUD$9,163) following the introduction of highly active antiretroviral therapy (HAART) (Table 4.4.1). Based on this data and a previous HIV/AIDS health care costing assessment in Australia (Hurley et al. 1995) we have estimated antiretroviral therapy per person costs to be AUD$4,000 for the period 1990-1996 and AUD$10,500 from 1997 (Table 4.4.2).

Table 4.4.1 Antiretroviral therapy expenditure in Australia, 1996-2000[1]

	1996	1997	1998	1999	2000
Total patients	5,617	6,425	6,085	6,296	6,520
Total costs (AUD$)	$24,632,000	$58,876,000	$66,312,000	$67,689,000	$69,413,000
Cost per patient (AUD$)	$4,385	$9,163	$10,897	$10,751	$10,646

1. Based on data available from the Highly Specialised Drug Scheme and the Australian HIV Observational Database (NCHECR 2001)

4.4.2 COSTS OF OTHER HIV/AIDS MANAGEMENT

HIV/AIDS Treatment protocols and costs were partly based on the previous HIV/AIDS health care costing assessment in Australia (Hurley et al. 1995). In addition, information on CD4 count and HIV viral load utilisation was obtained from AHOD. Regarding hospitalisation costs (including treatment of complications), the following assumptions were made:

- Only people who have progressed to AIDS are hospitalised.

- ☐ Annual hospitalisation, diagnosis and complication treatment costs for people with AIDS prior to 1997 are based on Hurley et al (1995).
- ☐ Total hospitalisation, diagnosis and complication treatment costs for people with AIDS are similar in the periods 1990-1996 and from 1997.
- ☐ Survival for people with AIDS is approximately 18 months pre-1997 and 3 years from 1997, therefore annual costs for hospitalisation, diagnosis and complication treatment costs for people with AIDS will be reduced by 50% from 1997 (Table 4.4.2).

Table 4.4.2 Treatment protocols and their costs for HIV disease

Disease stage and health care service costs	Costs
**1. Early HIV disease**[1]	
Doctor visits (specialist x 1, primary care x 3)	$213
HIV viral load testing x 3	$528
CD4 count x 3	$60
Other laboratory services (full blood count x 3, biochemistry x 3, liver function tests x 3)	$169
Antiretroviral therapy	$4,000 (1990-1996) $10,500 (from 1997) for estimated 40% on treatment
**2. Progressive HIV disease**[1]	
Doctor visits (specialist x 2, primary care x 4)	$295
HIV viral load testing x 4	$704
CD4 count x 4	$80
Other laboratory services (full blood count x 4, biochemistry x 4, liver function tests x 4)	$225
Antiretroviral therapy	$4,000 (1990-1996) $10,500 (from 1997) for estimated 70% on treatment
**3. AIDS**[2]	
Doctor visits (specialist x 2, primary care x 4)[1]	$295
HIV viral load testing x 4[1]	$704
CD4 count x 4[1]	$80
Other laboratory services (full blood count x 4, biochemistry x 4, liver function tests x 4)[1]	$225
Antiretroviral therapy	$4,000 (1990-1996) $10,500 (from 1997) for estimated 90% on treatment
Diagnosis of HIV complications	$3,228 (1990-1996) $1,614 (from 1997)
Prophylaxis and management of opportunistic infections[2]	$15,132 (1990-1996) $7,566 (from 1997)
Hospital bed-days[2]	$50,328 (1990-1996) $25,164 (from 1997)

1 Health care services are per year per case in disease stage, based on CMBS Schedule Fee (2000).
2 Cost estimates for AIDS (except antiretroviral therapy from 1997) are based on previous Australian figures from Hurley et al (1995), without adjustment for inflation.

The above annual costs of treatment of HIV by stage of disease were than converted to Year 2000 prices, where required, by application of the relevant CPI ratio. The annual costs of treatment by stage of disease were then applied to the number of survivors in each stage. Detailed figures of the results are provided in Table 4.4.3 (See Appendix D), with discussion presented in Section 4.6.

4.5 METHODOLOGY FOR HEALTH CARE COSTINGS FOR HCV INFECTION

The following assumptions were employed in determination of health care costs for hepatitis C:

- 75% of people who acquire HCV infection develop chronic hepatitis C.
- The health care costs of acute hepatitis C are small, due to the largely asymptomatic nature of newly acquired HCV infection, and therefore were not considered in the total costs.
- All people with chronic hepatitis C are at risk of progression to advanced liver disease complications.
- People can either remain in disease states or progress forward but not regress.

4.5.1 TREATMENT PROTOCOLS AND COSTS FOR HCV

Table 4.5.1 outlines treatment protocols for HCV and their cost estimates. Additional health care service items for stages 1-3, but for total period rather than per year are:

- Liver ultrasound x 2
- Liver biopsy x 2
- Pathology services (HIV serology x 1, HBV serology x 1, Iron studies x 1, full blood count x 4, alpha-1-antytrypsin level x 1, caeruloplasmin level x 1, ANA/auto-antibodies x 1, HCV genotype x 1, HCV viral load x 1)

These costs have been estimated at $2,358 per case (Year 2000 prices)

Hospitalisation is assumed to only be required for:

- Liver biopsy (day only stay)
- Liver failure (incorporated into total cost estimate)
- Hepatocellular carcinoma (incorporated into total cost estimate)

Of the estimated 150,000 people living with chronic hepatitis C in Australia (Law 1999), less than 10% will have received combination therapy, the preferred treatment for hepatitis C, consisting of interferon and ribavirin. For this reason, and the assumption that the costs of combination therapy will have been balanced by some reduction in development of advanced liver disease complications, we have not costed combination therapy in the analysis.

Table 4.5.1 Treatment protocols and their costs for HCV disease

Disease stage and health care services	Costs
**1. Mild chronic hepatitis C[1]**	
Doctor visits (specialist x 1, primary care x 2)	$164
Pathology services (liver function tests x 2)	$39
**2. Moderate chronic hepatitis C[1]**	
Doctor visits (specialist x 1, primary care x 2)	$164
Pathology services (liver function tests x 2)	$39
**3. Compensated cirrhosis[1]**	
Doctor visits (specialist x 2, primary care x 2)	$198
Pathology services (liver function tests x 2, full blood count x 1, alpha-fetoprotein x 1, liver ultrasound x 1)	$182

Table 4.5.1 (Cont) Treatment protocols and their costs for HCV disease

Disease stage and health care services	Costs
**4. Liver failure**[2]	
Without transplant (80% of patients), cost per patient	$164,340
With transplant (20% of patients), cost of transplant	$75,000
Expected cost per episode	$146,472
**5. Hepatocellular carcinoma**[2]	
Without surgery (76% of patients), cost per patient	$117,895
With surgery (33% of patients), cost per patient	$28,290
Expected cost per episode	$88,325

1 Health care services are per year per case in disease stage, based on CMBS Schedule Fee (2000).
2 Cost estimates are previous Australian figures from Shiell et al (1994), without adjustment for inflation.

4.6 HIV TREATMENT COSTS

The annual costs of treatment of HIV by stage of disease (Table 4.4.2) were converted to Year 2000 prices, where required, by application of the relevant CPI ratio. These were then applied to the number of survivors in each stage. Detailed figures of the results are provided in Table 4.4.3 (See Appendix D).

Figure 4.6 illustrates the annual costs of treatment for the diagnosed cases of HIV avoided as a result of the ten year investment in NSPs. Annual treatment costs rise progressively to the year 2008 as patients progress to later stages of the disease, at which time they peak at approximately $269 million. Thereafter, annual costs decline, brought about mainly by the declining number of patients in the second and third stages of HIV.

Figure 4.6 Annual costs of treatment of diagnosed cases of HIV avoided by NSPs (Not discounted)

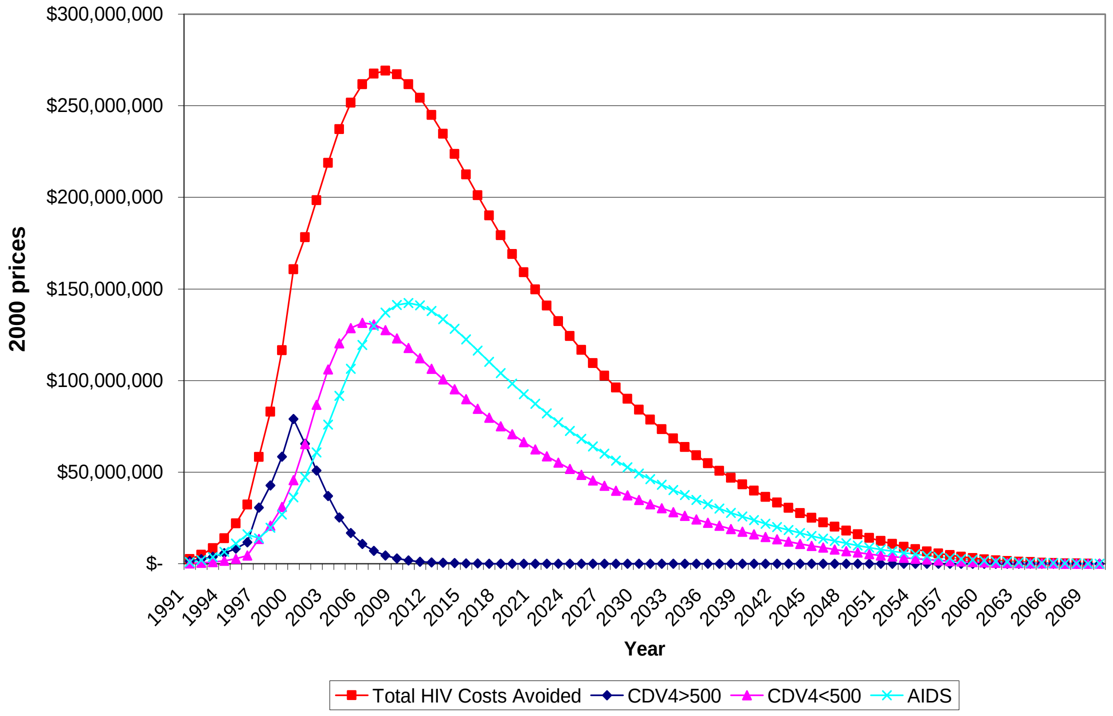

Cumulative HIV treatment costs avoided over the lifetime of consumers are illustrated in Figure 4.7. Costs accumulate throughout the lifetime of survivors, but at a slower rate after about 2008.

Figure 4.7 Cumulative costs of treatment of diagnosed cases of HIV avoided by NSPs (Not discounted)

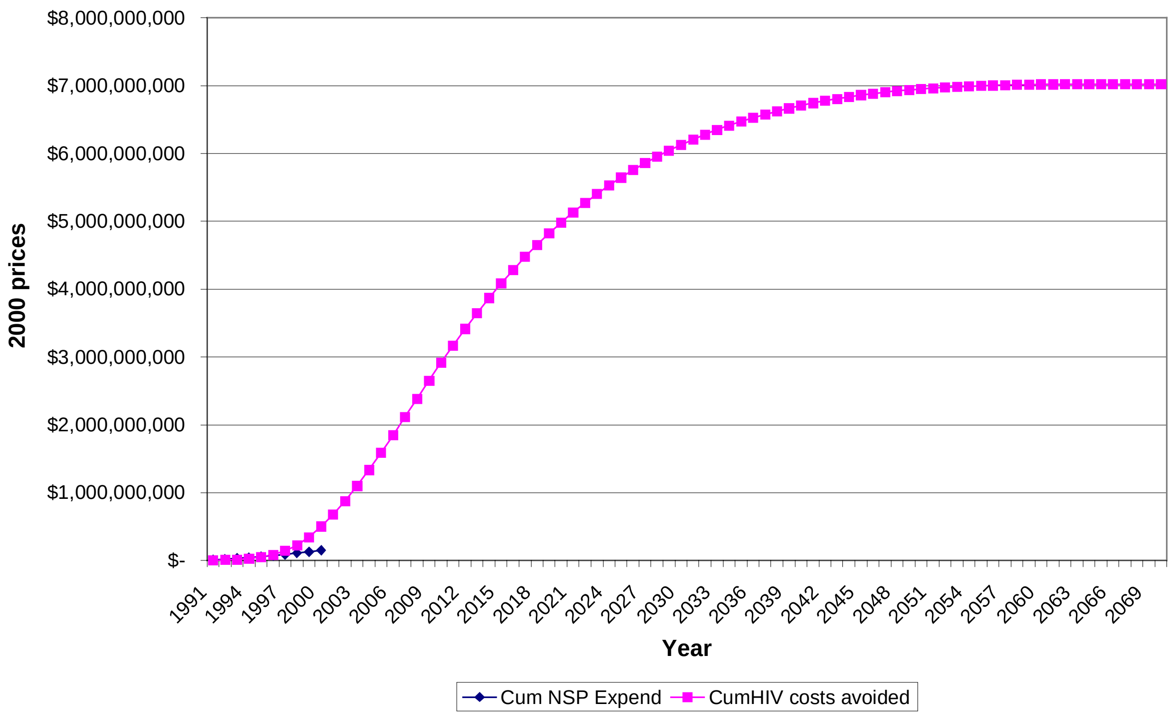

4.6.1 HCV Treatment Costs

The annual costs of treatment of HCV by stage of disease (Table 4.5.1) were converted to Year 2000 prices, where required, by application of the relevant CPI ratio. These were then applied to the number of diagnosed survivors in each stage of the disease. Detailed figures of the results are provided in Table 4.4.3 (See Appendix D).

Figure 4.8 illustrates the annual costs of treatment for the diagnosed cases of HCV avoided as a result of the ten year investment in NSPs. Annual treatment costs rise progressively to the year 2040, at which time they peak at approximately $18.8 million and decline thereafter. The major factor influencing this cost profile is the number of patients with liver failure who, while relatively small in number, have extremely high costs of treatment.

Cumulative costs of treatment of HCV are presented in Figure 4.9. Costs accumulate throughout the period as patients progress through stages of HCV, reaching a plateau in the late 2050s. The shapes of the curves for both annual and cumulative costs of HIV and HCV treatment are indicative of the different rates of progression through each disease, with progression in HCV occurring at a much slower rate than for HIV, and hence treatment for later stages of the disease peaking much later for HCV than for HIV. This deferral has implications for the determination of the Net Present Values of this expenditure, as discussed below.

Overall, total treatment costs avoided over the life of the cases of HIV and HCV avoided by NSPs are approximately $7,808 million (before discounting). The costs of HIV treatment avoided are approximately ten times those for HCV, which reflects a combination of the number of cases avoided in the first instance (25,000 for HIV compared to 21,000 for HCV), a higher diagnosis rate for HIV than HCV, and higher average annual treatment costs for HIV than for HCV.

Figure 4.8 Annual costs of treatment of diagnosed cases of HCV avoided by NSPs (Not discounted)

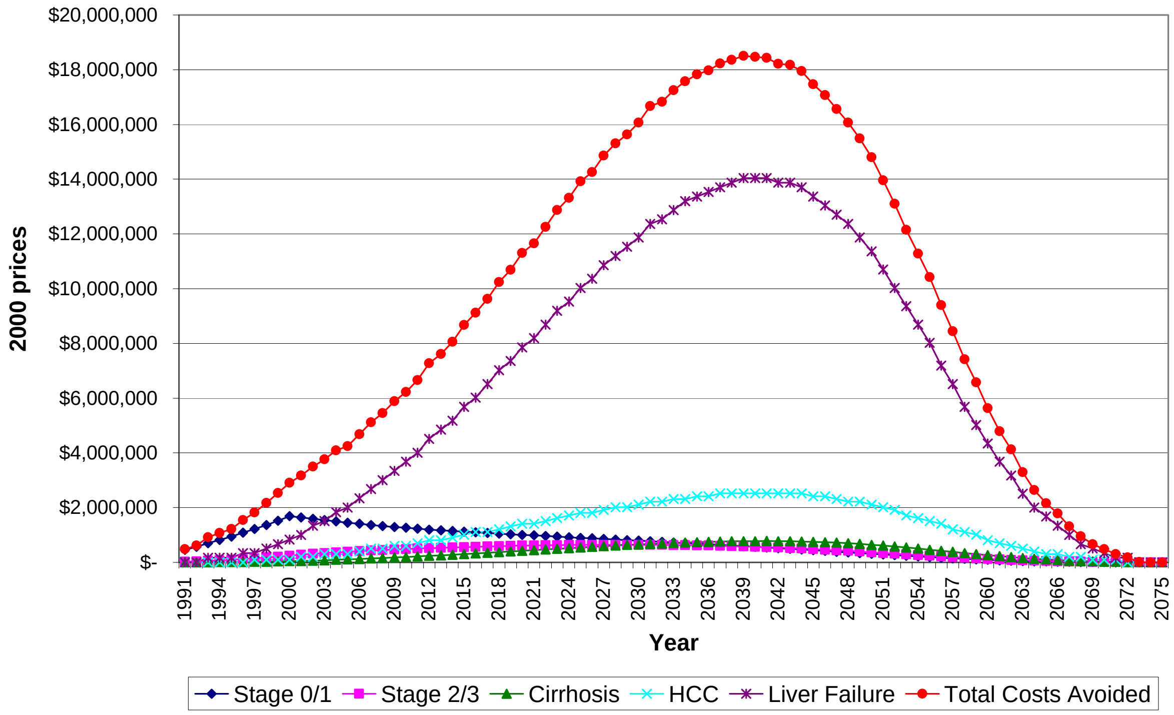

Figure 4.9 Cumulative costs of treatment of diagnosed cases of HCV avoided by NSPs (Not discounted)

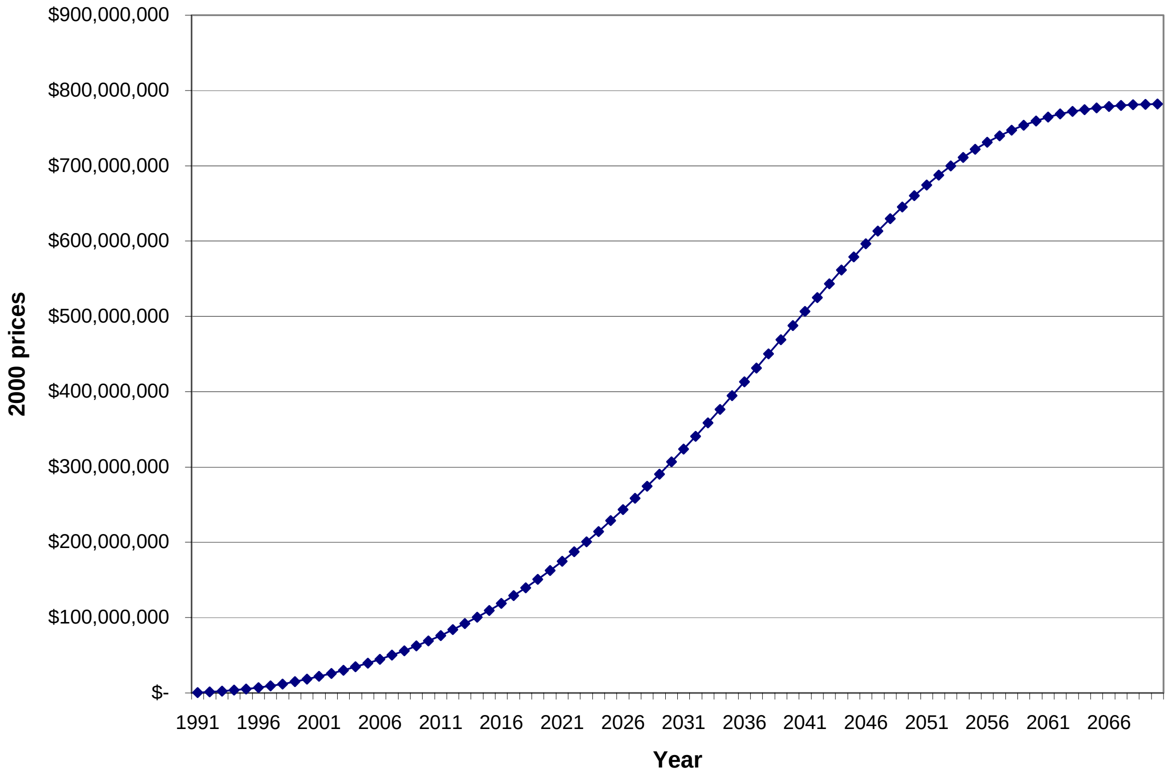

4.7 RETURN ON INVESTMENT

The calculation of the return on investment from NSPs takes into account the total investment by government and consumers in NSPs during the 1990s, together with anticipated savings resulting from treatment costs avoided for persons who would otherwise have contracted HIV and HCV over their lifetime, were it not for the availability of NSPs during the decade under study.

The calculation of return on investment discounts future cashflows associated with the investment in the NSP program and treatment costs avoided by an agreed discount rate. The discount rate most commonly used in government programs of this nature is 5% per annum. For the purposes of illustration, we have also applied discount rates of 3% and 0%.

When considering the return on investment, one of the questions to be considered is "Whose investment?" In this instance, expenditure on NSPs has been made by government and consumers. In our analysis, we have presented findings that illustrate both the return to government from its investment, as well as the return on total investment. We have also considered the return on investment over the lifetime costs of treatment of cases avoided, as well as the return achieved to the end of the investment period itself (Year 2000) ignoring any savings that accrue thereafter.

The impact of NSPs on both HIV and HCV has been considered in the analysis. Given the history of NSPs, their original purpose, and the stronger evidence base demonstrating their impact on the incidence of HIV among injecting drug users (see Section 3), our primary focus has been on HIV. Our initial analysis therefore considers the return on investment in NSPs from HIV avoidance alone. In the second part of the analysis, we consider what additional savings may have been derived from the avoidance of HCV among injecting drug users.

4.7.1 HIV IMPACTS

The results of the analysis on return on investment in NSPs to government and in total, having regard to the impacts on HIV alone, are presented in Table 4.7.1. Detailed tables are provided in Table 4.4.3 (See Appendix D).

Table 4.7.1 Net Present Value of investment in NSPs for HIV.

Discount Rate	Net Present Value, 1991 ($million, Year 2000 Prices)	
	Govt Expenditure	All Expenditure
	**Lifetime Costs of Treatment**	
5%	$2,277	$2,262
3%	$3,415	$3,398
0%	$6,896	$6,876
	**To Year 2000**	
5%	$242	$227
3%	$287	$270
0%	$373	$353

The table illustrates that the net savings to government from its investment in NSPs over the lifetime of cases of HIV avoided (after deducting the value of the initial government investment) before discounting are $6,896 million. Discounting these savings at 5% results in a Net Present Value (NPV) of their investment of $2,277 million ($3,415 million at 3% discount rate). When considering the total investment in NSPs (by including consumer

expenditure), the equivalent returns are $6,876 million (undiscounted), $2,262 million (discount rate of 5%) and $3,398 million (discount rate of 3%).

To put these outcomes in perspective, they represent the savings that accrue from a combination of the following:

- ☐ A total investment of approximately $150 million (Year 2000 prices) in NSPs during the 1990s, that resulted in
- ☐ approximately 25,000 cases of HIV avoided, who
- ☐ live for an average of about 24 years after infection, and who
- ☐ incur average treatment costs of nearly $14,000 each year of their life after diagnosis.

Under these circumstances, the analysis indicates that there have been significant savings accruing to government from the investment in NSPs to date, and that these savings will continue to accrue into the future.

This is further illustrated by considering the return achieved to the end of the investment period (i.e. to Year 2000) without taking into account any additional savings that accrue in the future. This is also demonstrated in Table 4.7.1 where the NPV of the savings to the Year 2000 are shown, both to government and as a whole. By the year 2000, government had achieved net savings of $373 million (after deducting the value of their investment), the NPV of which at a discount rate of 5% is $242 million ($287 million at a discount rate of 3%). The equivalent returns on the total investment in NSPs over the same period were $353 million (undiscounted), $227 million (discount rate of 5%) and $270 million (discount rate of 3%)

4.7.2 HIV AND HCV IMPACTS COMBINED

In the second stage of the analysis, we consider the effects of NSPs on HIV and HCV combined. The return on investment in NSPs to government and in total, having regard to the impacts on HIV and HCV combined, are presented in Table 4.7.2. Detailed tables are provided in Table 4.4.3 (See Appendix D).

Table 4.7.2 Net Present Value of investment in NSPs for HIV and HCV combined.

Discount Rate	Net Present Value, 1991 ($million, Year 2000 Prices)	
	Govt Expenditure	All Expenditure
**Lifetime Costs of Treatment**		
5%	$2,402	$2,386
3%	$3,653	$3,637
0%	$7,678	$7,658
**To Year 2000**		
5%	$255	$240
3%	$302	$285
0%	$391	$371

The table illustrates that the net savings to government from its investment in NSPs over the lifetime of cases of HIV and HCV avoided (after deducting the value of the initial government investment) before discounting are $7,678 million. Discounting these savings at 5% results in a Net Present Value (NPV) of their investment of $2,402 million ($3,653 million at 3% discount rate). When considering the total investment in NSPs (by including consumer expenditure), the equivalent returns are $7,658 million (undiscounted), $2,386 million (discount rate of 5%) and $3,637 million (discount rate of 3%).

The analysis indicates that the incorporation of HCV into the NPV calculations has further increased the savings accruing to government and in total. This is to be expected, as no additional investment has been required, and some 21,000 cases of HCV are estimated to have been avoided. The impact on savings, however, is significantly lower than for HIV, due to the lower annual costs of treatment for the earlier stages of HCV, and the fact that the higher costs associated with the relatively small proportion of patients who progress to liver failure are deferred until much later and are considerably reduced by discounting.

As noted in Section 4.5.1, we have not taken into account the costs of combination therapy for the treatment of HCV, due primarily to the small proportion of people with HCV receiving this treatment to date. Should this situation change and combination therapy become more widely prescribed, annual treatment costs are also expected to increase. Under these circumstances, the estimates of savings presented above are likely to underestimate the savings that would accrue under this treatment regime. This, of course, would also depend on the effect of combination therapy in slowing the rate of disease progression.

4.8 SENSITIVITY ANALYSIS

The analysis presented above has been based on the best estimates available for each of the key variables used in the economic model. In order to test the robustness of the results, sensitivity analysis has been conducted on a number of the variables affecting the outcomes. These are:

- ☐ Halving the rate of effect of NSPs on HIV. This analysis seeks to address the issue of the extent to which NSPs contribute to the reduction in HIV as opposed to other concomitant activities (see Section 3.1.7).
- ☐ Quartering the effect of NSPs on HIV. This analysis further extends the examination of reduced NSP effects on HIV.
- ☐ Doubling the level of investment in NSPs over the ten years. This analysis examines the result of increasing the expenditure on NSPs without any increase in effect on HIV. By so doing, it takes into account the potential contribution of the commercial pharmacy market.
- ☐ Halving the annual treatment costs for HIV. This analysis considers the results of possible future reductions in the costs of HIV treatment.

The outcomes for each of these variations in isolation are illustrated in Table 4.8.1, applied only to the impact on HIV, and based on a discount rate of 5% in all scenarios.

Table 4.8.1 Net Present Value of investment in NSPs for HIV – Sensitivity Analysis.

	**Net Present Value, 1991 ($million, Year 2000 Prices)**	
	**Govt Expenditure**	**All Expenditure**
	**Lifetime Costs of Treatment**	
Original Estimate	$2,277	$2,262
Half NSP Effect on HIV	$333	$318
Quarter NSP Effect on HIV	$52	$37
Double NSP Investment	$2,180	$2,151
Half HIV Annual Treatment Costs	$1,090	$1,075

The analysis indicates that the outcomes previously presented are most sensitive to the impact of NSPs on HIV incidence. This is to be expected because of the nature of the estimation technique employed, which uses the logit scale as its base. Consequently, halving the rate of effect of NSPs on HIV incidence has a proportionally

greater effect on the number of cases avoided over time. Nevertheless, even at the most conservative estimate of effect (one-quarter of the original effect estimate) the return on investment on both government expenditure and total expenditure on NSPs is positive. This also holds true for variations to the other input variables in the model. Of some interest is the fact that even when the annual costs of treatment for HIV are halved, NSPs continue to meet the required investment criteria. The sensitivity analysis indicates that the results presented are robust, and that the return on investment from NSPs is positive in all other tested scenarios.

4.9 DISCUSSION

The evaluation of the financial effect of NSPs on HIV and HCV has been based on:

- The reported operating and overhead costs of NSPs across Australia during the 1990s;
- Estimates of the number of cases of HIV and HCV avoided as a result of NSPs;
- Past and current treatment regimes and associated costs of treating HIV and HCV;
- Projections of future treatment costs based on the above; and
- The application of a Net Present Value (NPV) model to determine the return on investment to both government and in total for NSPs.

The analysis indicates that the return on investment will exceed manyfold the original investment in NSPs, and that the original investment had been fully recouped and surpassed by the end of the investment period, before any future savings are taken into account. The investment in NSPs is justified by the effect on HIV alone, with the effect on HCV providing an additional financial benefit, albeit a smaller one than HIV. Sensitivity analysis on the main variables used in the analysis indicates that the results are robust under a range of alternative assumptions and scenarios.

A number of observations are offered about the results presented.

- The factor that has the greatest impact in the financial analysis is the effect of NSPs in reducing the incidence of HIV (and to a lesser extent HCV). The evidence base for the estimation of effect has increased significantly since the earlier study by Hurley and Kaldor, with a greater number of sites now reporting data on HIV seroprevalence, which has been used in the analysis of effect presented in Section 3. As noted in Section 3.1.7, however, NSPs typically operate in an environment where a range of public health and other initiatives are in place. It is not possible to isolate the effects of NSPs from other elements in these initiatives. Indeed, it may be that NSPs are simply a “marker” for a range of activities whose combined effect is that demonstrated to date. Notwithstanding this point, the sensitivity analysis conducted indicates that even under scenarios where the effect of NSPs on HIV incidence is reduced by 75% of the original estimate, the return on investment from NSPs remains positive.
- The analysis of return on investment has considered only the direct costs and savings associated with NSPs. In particular, only direct health care savings relating to treatment costs of HIV and HCV have been incorporated into the analysis. There is clearly a greater range of other potential financial savings to be derived from a reduction in HIV and HCV, savings that would accrue to governments, patients and their carers as well as wider society. For example, savings to government and the community in terms of employment and unemployment, education and parenting costs are likely to be considerable. The exclusion of these savings from the analysis means that the case presented may understate, potentially significantly, the total financial benefits of NSP programs.
- Estimates of future treatment costs have been based on current treatment regimes and the costs associated with those regimes. As has been shown with the introduction of antiretroviral therapy for the treatment of HIV, methods and costs of treatment can change very quickly, which may have a significant effect on patients, as well the analysis of return on investment. The sensitivity analysis conducted on the results to date indicates that, even if future treatment costs halve, NSPs would continue to be a sound

investment strategy. However, any radical changes to treatment methods and their effect on disease progression and life expectancy may affect the outcomes presented.

- The analysis presented has considered the retrospective investment in NSPs, as well as the direct health care savings accrued to date and in the future associated with that investment, and assumes that the investment in NSPs ceases in the year 2000. Given the current population of injecting drug users and the level of use of NSP services, together with the demonstrated effect of NSPs on HIV and HCV, it is clear that an ongoing investment in NSPs will continue to avert the incidence of HIV and HCV, and that savings will continue to accrue. Consequently, the model demonstrates not only that the return on investment to date in NSPs has been positive, but also that ongoing investment in NSPs is warranted.

5 QUALITY OF LIFE (QOL) EFFECTS OF NSPS

5.1 INTRODUCTION

Since both HIV and HCV are potentially life-threatening conditions, one of the main benefits from averting infections is the prevention of premature mortality. However, there are considerable contrasts in the natural history of these two chronic viral infections, particularly in the rate of progression to advanced disease and related complications.

Prior to the introduction of improved antiretroviral therapy in the mid-1990s, a half of people with HIV would have developed advanced immunodeficiency and associated AIDS illness complications during the first 10 years of infection. Although a small proportion of people – possibly 5% – were considered long-term non-progressors the majority of the other half would have had evidence of immune function deterioration, and were at risk of progression to AIDS in subsequent years. Survival following development of AIDS was approximately 18 months. Thus, it was expected that very few people with HIV would have been alive or free of AIDS after 15-20 years infection. Although several pre-AIDS HIV conditions are associated with considerable morbidity, the major morbidity associated with HIV infection was as a result of the development of specific AIDS illnesses.

Since the introduction of highly active antiretroviral therapy (HAART), HIV disease progression has markedly slowed, both for people prior to and following development of AIDS illnesses. Morbidity and mortality associated with AIDS illnesses have declined by 50-80% in settings where access to HAART is widespread. On the other hand, there has been an increase in morbidity associated with side effects of therapy. This effect, however, is relatively modest when compared with prior AIDS illness-related morbidity. The sustainability of the effect of HAART on immune function, and longer-term therapeutic toxicity are two areas where there is some uncertainty regarding levels of morbidity and mortality that will be experienced by people with HIV in the next decade.

The natural history of HCV infection varies with HIV in many areas. First, a proportion of people – possibly 20-40% – do not develop chronic infection and are therefore not at risk of advanced liver disease complications. Second, progression to advanced disease is both slower than HIV and not inevitable. An estimated 20% of people with chronic hepatitis C will develop cirrhosis over 15-40 years and be at risk of liver failure and liver cancer. Although the remaining 80% will not develop morbidity associated with advanced liver disease complications, many people suffer considerable morbidity related to a range of relatively non-specific symptoms. These include chronic lethargy, abdominal discomfort, and headaches. There is also recent evidence that HCV may cause cognitive impairment including difficulties with concentration. Pre-advanced liver disease morbidity in chronic hepatitis C is unrelated to the extent of liver damage.

As with HIV, antiviral therapy for HCV has improved in recent years. Currently, 50% of people with chronic hepatitis C who commence standard of care antiviral therapy (interferon and ribavirin combination) develop a sustained response that equates to a probable cure of their infection. Antiviral therapy therefore has the potential to considerably reduce morbidity and mortality related to chronic hepatitis C, however, for several reasons uptake to date has been relatively limited.

HIV and HCV may also have psychosocial effects among infected persons, some of which may be associated with discrimination and social stigma. These will also impact on the person’s quality of life, regardless of whether the disease progresses or not.

Given the above, it is likely that gains in quality of life are one of the major health benefits of the prevention of HIV and HCV infections.

5.2 Quality-Adjusted Life-Years (QALYs)

The most widely used approach for estimating quality of life benefits in economic evaluations is the quality-adjusted life-year (QALY). In this approach, states of health are assigned a health state preference or 'utility' value, on a scale including 1.0 (full health) and 0 (death). The amount of time an individual spends in a given health state is then multiplied by the health state preference value to calculate the quality-adjusted life-years (QALYs) gained.

The QALYs gained from a given health care intervention are estimated by considering the difference in progression, through the various health states, with and without the intervention concerned. This is shown schematically in Figure 5.1. Here the intervention leads to QALY gains both by increasing or maintaining quality of life and by extending life. The main advantage of the QALY approach is that it provides one combined measure of the benefits of a program that both extends life and maintains quality of life.

In the context of HIV or HCV, we might expect that the health state values for early stages of disease, such as early HIV (CD4 count above 500/mm^3) or mild chronic hepatitis, are higher than those for the later stages of disease, such as AIDS or liver failure. Therefore, if the NSP reduces the probability of infection, or increases the average time to infection, we would expect fewer individuals in a cohort of IDUs to progress to the later stages of disease during their lifetime. Under the QALY approach this will lead to QALY gains.

Figure 5.1 Quality-adjusted life years

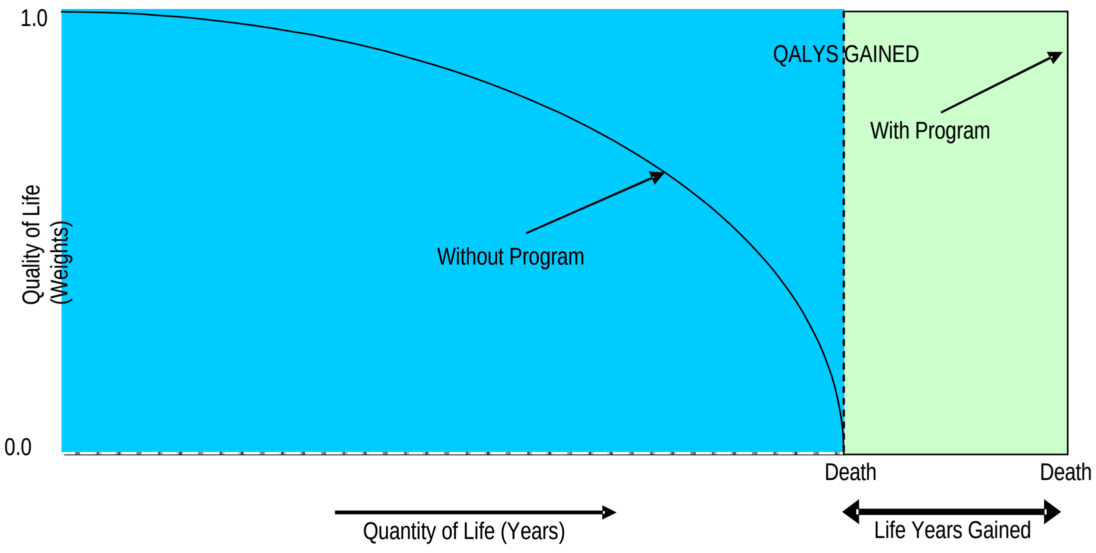

5.3 Methodology for estimating the QALYs gained from the prevention of HIV and HCV infections

In order to calculate the QALYs gained it is necessary to estimate the duration of time individuals will experience particular health states and the 'utility' values for those states. The durations of time spent in particular health states for someone with HIV or HCV has been estimated from the epidemiological literature and Australian data. These have been incorporated into the calculation of the number of cases prevented by stage of disease and the projections of survivors beyond 2000, as outlined in Section 3, and presented in Tables 3.4.5 and 3.5.5 in Appendix C.

Health state preference values for the disease stages are not readily available. One approach would be to undertake a free-standing study to estimate the values by standard gamble or time trade-off (Drummond, et al, 1997). However, this approach was considered beyond the scope of the current project.

Rather, it was decided to use values for health states existing in the literature. For example, Tengs et al (2000) report more than 1,000 health state values, many of which relate to HIV or HCV. In addition, there have been some papers published specifically relating to HIV (Holtgrave and Pinkerton, 1997) and HCV (Bennett, et al, 1997)

5.3.1 QALY VALUES FOR HIV

The quality of life estimates for HIV have been based on a review updating costs of HIV illness and quality of life estimates since the introduction of combination antiretroviral therapy (Holtgrave & Pinkerton 1997). Their estimates were based on an extensive review of studies, in which HIV-infected patients formed the study population of almost all identified studies. There are some differences in the disease phases they used and those we have employed. We have divided the undiagnosed HIV phase into two, one with “early HIV disease” and one with “progressive HIV disease”. In Holtgrave & Pinkerton (1997) all people with undiagnosed HIV were given a quality of life rating of 0.94. We have assumed that people with “progressive HIV disease” who remain undiagnosed would in general have a slightly lower quality of life rating than those with earlier HIV disease. We have assumed that all people with AIDS are diagnosed.

Definitions for disease states and associated quality of life adjustments for HIV are presented in Table 5.3.1:

Table 5.3.1 Quality of life values by disease stage of HIV

Disease Stage	Description	QALY Value
Early HIV Disease – Undiagnosed.	HIV infection with CD4 count above 500/mm^3, unaware of HIV serostatus.	0.94
Early HIV Disease – Diagnosed.	HIV infection with CD4 count above 500/mm^3, aware of HIV serostatus and no antiretroviral therapy.	0.87
Progressive HIV Disease – Undiagnosed.	HIV infection with CD4 count below 500/mm^3, unaware of HIV serostatus.	0.90
Progressive HIV disease – Diagnosed.	HIV infection with CD4 count nadir below 500/mm^3 and commenced on antiretroviral therapy.	0.76
AIDS	AIDS as defined by clinical condition.	0.62

5.3.2 QALY VALUES FOR HCV

Quality of life adjustments for HCV were partly based on previous published estimates from a panel of hepatologists (Bennett et al. 1997). However, recent evidence from quality of life assessments among patient assessments was used to adjust the ratings provided by Bennett et al (1997). For example, studies indicate no significant difference in quality of life based on either degree of hepatic inflammation (as measured by ALT/AST) or extent of hepatic fibrosis (Bonkovsky et al. 1999). Therefore, we have used the same quality of life adjustment for diagnosed mild and moderate chronic hepatitis. Undiagnosed categories have higher quality of life estimates for two reasons. Firstly, development of symptomatic disease may often be a reason for HCV testing. Secondly, recent evidence suggests that quality of life impairment increases following diagnosis of hepatitis C (Rodger et al. 1999). We have combined the quality of life adjustments from Bennett et al (1997) for ascities (0.35), variceal haemorrhage (0.28), and hepatic encephalopathy (0.30), to produce a category for liver failure (0.32). We have assumed that all people with liver failure and HCC are aware of their HCV status. For the 25% of HCV infections that do not progress to chronic hepatitis, we have allocated a quality of life value of 1.0. This is a conservative estimate, as many people with hepatitis C who have not progressed to chronic infection are unaware of their non-viraemic status and may suffer significant impairment in quality of life related to psychosocial mechanisms.

Definitions for disease states and associated quality of life adjustments for HCV are presented in Table 5.3.2:

Table 5.3.2 Quality of life values by disease stage of HCV

Disease Stage	Description	QoL Value
HCV antibody positive – non-chronic hepatitis C.	HCV infected but does not progress to chronic hepatitis.	1.00
Mild chronic hepatitis – Undiagnosed	Chronic hepatitis C, unaware of HCV status, with stage 0-1 (no-minimal) hepatic fibrosis.	0.94
Mild chronic hepatitis – Diagnosed.	Chronic hepatitis C, aware of HCV status, with stage 0-1 (no-minimal) hepatic fibrosis.	0.82
Moderate chronic hepatitis – Undiagnosed.	Chronic hepatitis C, unaware of HCV status, with stage 2-3 (moderate-severe) hepatic fibrosis.	0.94
Moderate chronic hepatitis – Diagnosed.	Chronic hepatitis C, aware of HCV status, with stage 2-3 (moderate-severe) hepatic fibrosis.	0.82
Compensated cirrhosis – Undiagnosed	Chronic hepatitis C, unaware of HCV status, with associated cirrhosis but no evidence of liver failure or hepatocellular carcinoma (HCC).	0.84
Compensated cirrhosis – Diagnosed	Chronic hepatitis C, aware of HCV status, with associated cirrhosis but no evidence of liver failure or hepatocellular carcinoma (HCC).	0.74
Liver failure	Chronic hepatitis C associated cirrhosis that has progressed to de-compensation.	0.32
Hepatocellular Carcinoma (HCC)	Chronic hepatitis C associated cirrhosis that has progressed to HCC.	0.10

5.3.3 ADDITIONAL ELEMENTS OF THE QALY CALCULATIONS

Although somewhat more controversial than discounting costs, it is conventional to discount QALYs by the same rate. This has the effect of slightly reducing the estimate of the total QALYs gained from NSPs, as many of the QALYs are gained in the future.

5.4 NUMBER OF CASES OF HIV AND HCV AVOIDED

The estimates of the number of cases of HIV and HCV avoided as a result of the availability of NSPs during the 1990s are detailed in Section 3, and in Tables 3.4.5 and 3.5.5 in Appendix C. The outcomes have also been illustrated previously in Figures 4.2 and 4.3.

In summary, approximately 25,000 cases of HIV infection and 21,000 cases of HCV infection are estimated to have been avoided as result of NSP activities during the 1990s. Of those with HCV infection, approximately 16,000 persons would develop chronic hepatitis C. By the year 2010, some 4,500 deaths from HIV are estimated as being avoided, approximately 650 fewer people would be living with cirrhosis and 90 HCV-related deaths would have been prevented.

5.5 NUMBER OF LIFE YEARS GAINED

The number of life years gained provides a measure of the additional number of years by those persons who would otherwise have been infected with HIV and HCV, but for the effect of NSPs. This is estimated by deducting the number of life years they would have lived with HIV or HCV from the number of years they are expected to live without the disease. The results are indicative of the effect of the mortality rate for HIV/HCV on the target population, compared to that for the same population without these diseases.

The effect of NSPs on HIV and HCV is illustrated in Figures 5.2 and 5.3, with detailed tables provided in Table 5.5.1 (See Appendix E).

Figure 5.2 Life Years Gained for HIV cases avoided

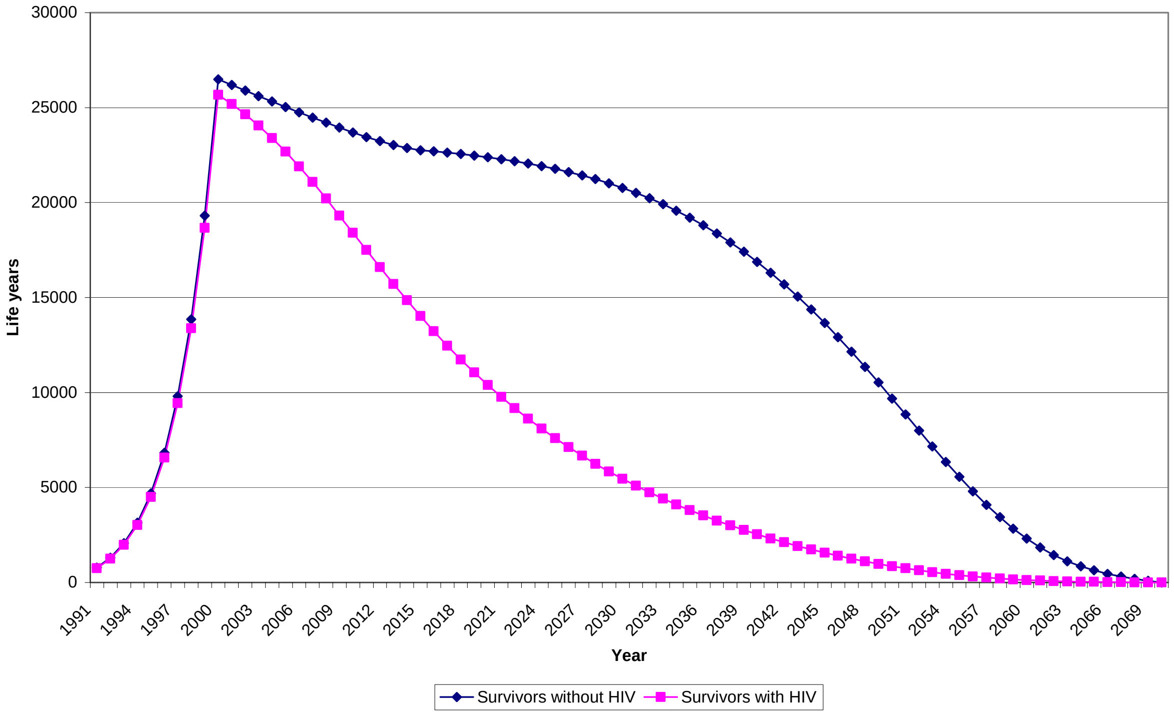

Figure 5.3 Life Years Gained for HCV cases avoided

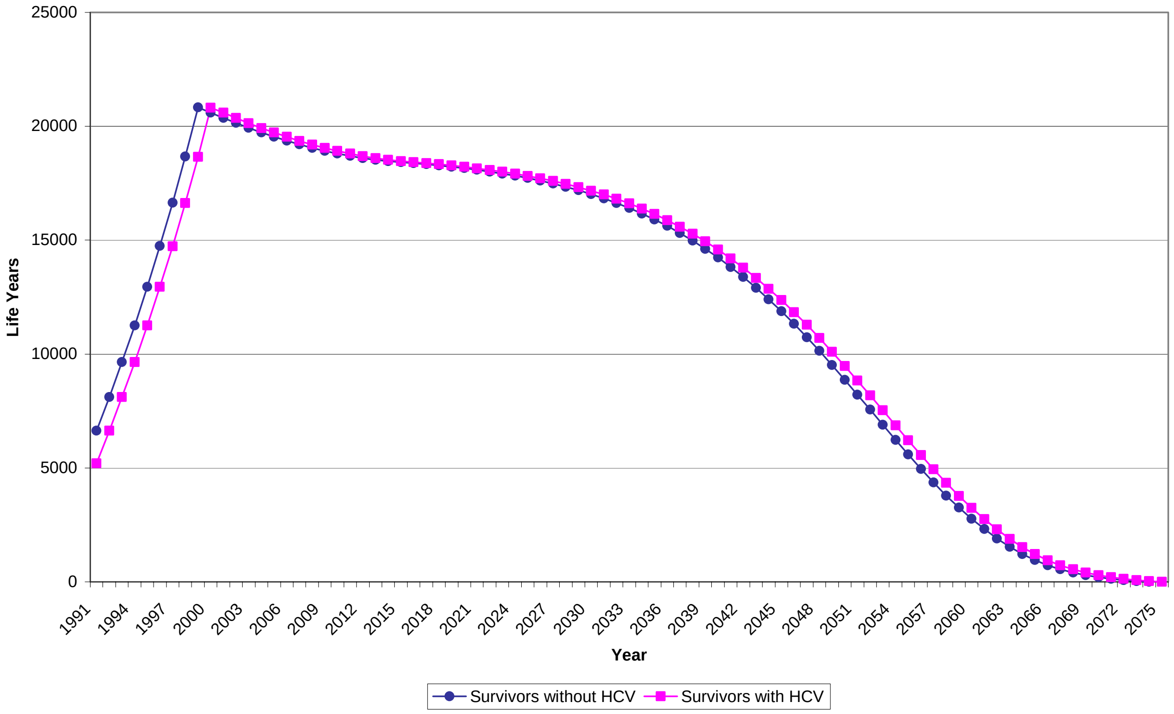

In the figures, the gap between the curves “Survivors without HIV/HCV” and “Survivors with HIV/HCV” represents the number of life years saved over the lifetime of those affected. As is evident from both the figures and the tables, the effect of NSPs in terms of life years saved is much greater for HIV than for HCV. The 25,000 persons avoiding HIV are expected to gain an additional 588,000 life years (about 23 years each) than if they had contracted HIV. In comparison, the 21,000 persons avoiding HCV are expected to gain only about 1,200 life years

over their lifetime. The difference in these outcomes is essentially due to the different mortality rates associated with each disease and their rate of progression through the various stages, both of which have been incorporated into the analysis.

5.6 Quality Adjusted Life Years Gained

The preceding analysis of life years gained takes into account the mortality effect of HIV and HCV on persons within the target population, namely injecting drug users. However, this analysis does not take into account any differences in the quality of life for those with HIV or HCV compared to those without the disease. As discussed in Section 5.2, the application of an adjustment factor to take account of the quality of life effects of these diseases leads to a measure referred to as Quality Adjusted Life Years (QALYs). Comparing this measure to the number of life years that the affected population lives in a disease-free state (i.e. by avoiding HIV and HCV) provides a measure of the QALYs gained as a result of NSPs. QALYs gained therefore incorporate both the quantity of life gained, and the quality of life gained by avoiding HIV and HCV.

The outcomes of this analysis are presented in Table 5.5.1 (See Appendix E) and in Figures 5.4 to 5.7.

Figure 5.4 Life Years and QALYs gained by HIV survivors

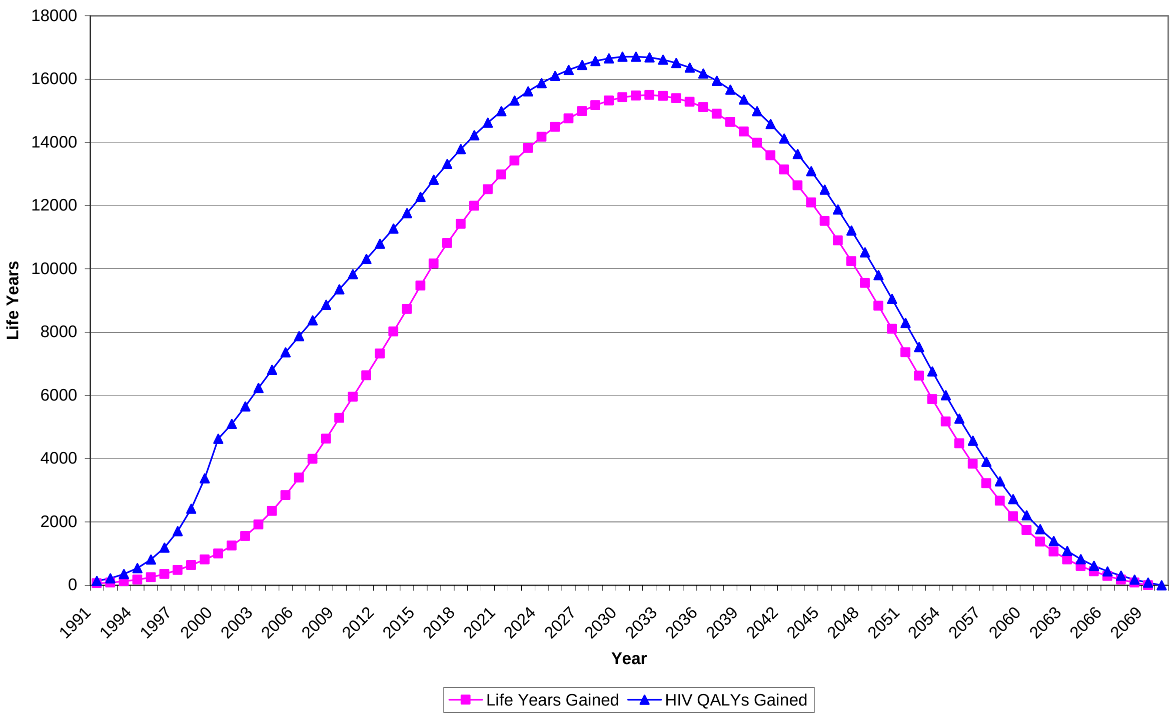

Figure 5.5 Life Years and QALYs gained by HCV survivors

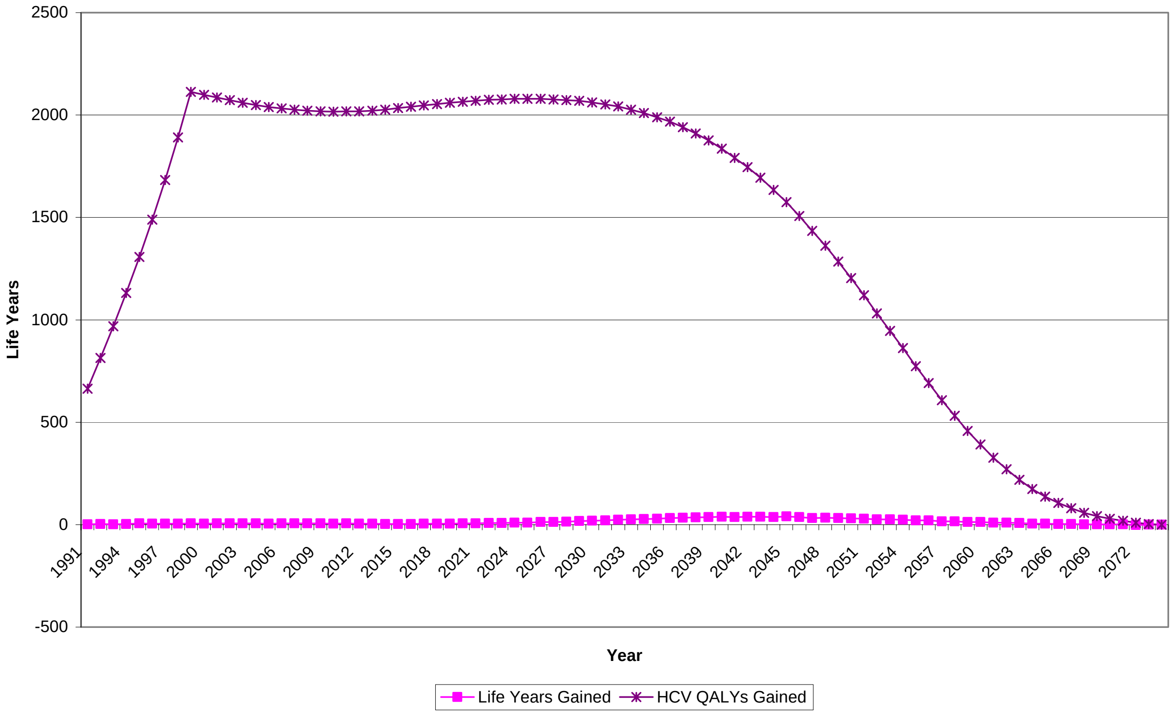

In figures 5.4 and 5.5 the curves "HIV/HCV QALYs Gained" represent the quality adjusted life years for persons who would have had HIV/HCV, but for the effect of NSPs. The gap between these curves and the curves "Life Years Gained" represents the quality effect of HIV and HCV on their lives. The 25,000 persons avoiding HIV are expected to gain an additional 715,000 quality adjusted life years than if they had contracted the disease. In comparison, the 21,000 persons avoiding HCV are expected to gain about 120,000 quality adjusted life years over their lifetime. The difference between the two groups is largely attributable to the greater effect of HIV on the "quantity" of life compared to HCV, rather than the "quality" effect.

In the case of HIV, the number of life years gained each year increases up to the year 2033, and thereafter continues at a progressively slower rate. The curve for QALYs gained generally follows a similar pattern, reflecting the dominant effect of the "quantity" component.

In contrast, the number of life years gained for persons avoiding HCV is relatively small. However, when considering the effect of HCV on the quality of life, considerable gains are evident. These gains are relatively constant up to the year 2035, then decline each year to death.

Figures 5.6 and 5.7 illustrate the cumulative number of life years and QALYs gained by avoiding HIV and HCV. The shape of the curves "Life Years Gained" illustrates the progressive effect of the different mortality rates and is considerably steeper for HIV than for HCV. The increasing gap between "Life Years Gained" and "QALYs Gained", and the timing of its emergence, illustrates the differences in quality of life effect between the two diseases.

Figure 5.6 Cumulative Life Years and QALYs gained by HIV survivors

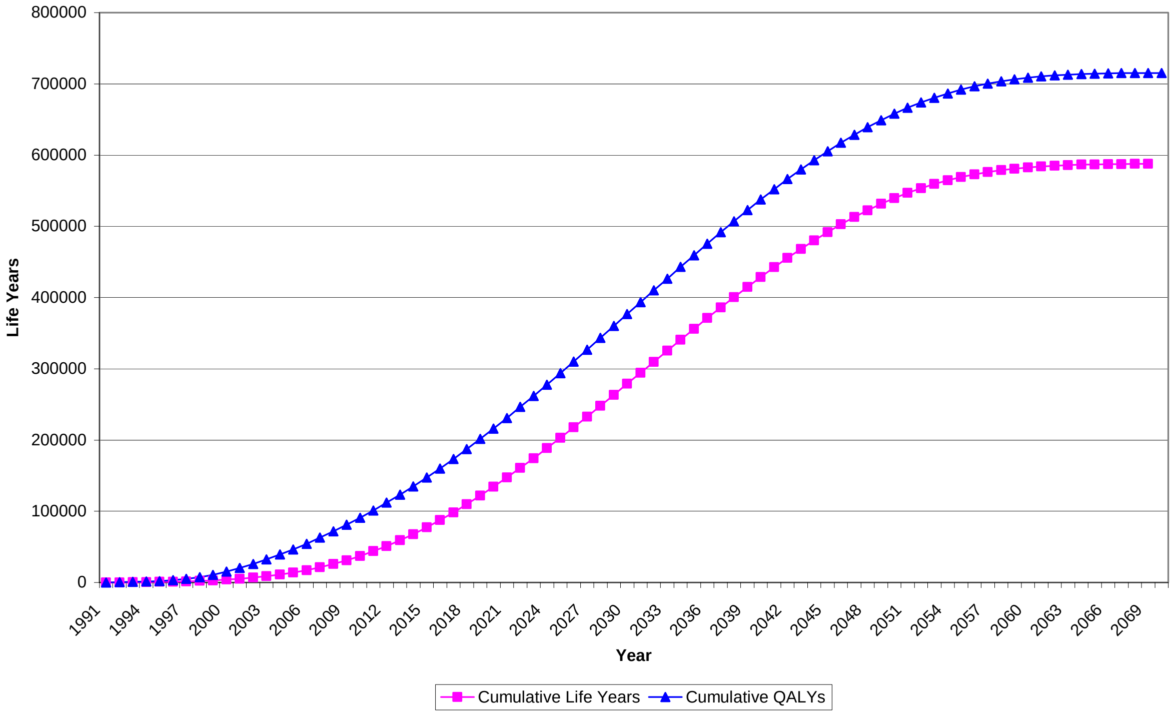

Figure 5.7 Cumulative Life Years and QALYs gained by HCV survivors

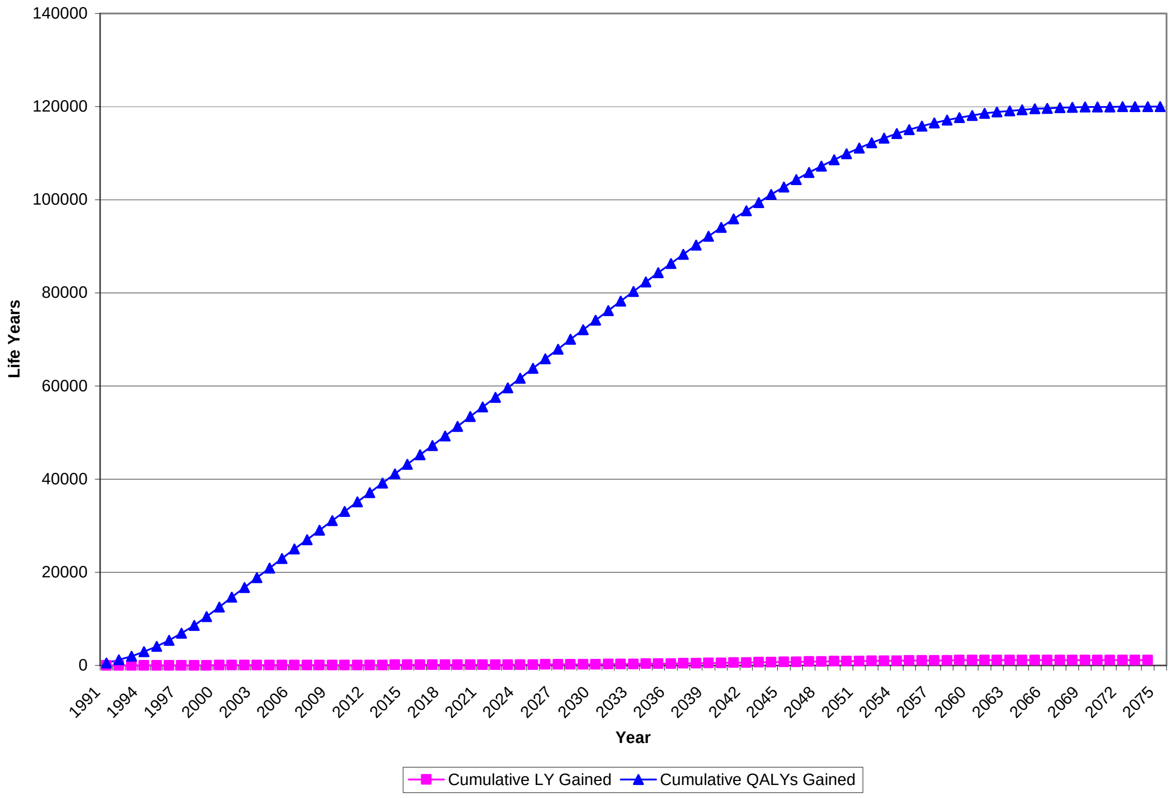

As previously noted, it is not uncommon to discount QALYs gained in the future in the same way as we have discounted future financial benefits. This approach is based on the principle that an improvement in the quality of

life is likely to be valued more if it occurs earlier than if it occurs later in life, just as a dollar gained earlier is likely to be valued more than a dollar gained later.

Applying the same discount rates used in the financial analysis (viz 5%, 3% and 0%) to QALYs gained results in the figures shown in Table 5.6.1

Table 5.6.1 Net Present Value of QALYs gained for HIV and HCV

Discount Rate	Net Present Value, 1991 (QALYs)		
	HIV	HCV	HIV & HCV
5%	138,072	32,207	170,279
3%	248,364	50,041	298,406
0%	715,245	119,992	835,237

A total of approximately 715,000 QALYs were gained by the avoidance of HIV, the present value of which, at a discount rate of 5%, is 138,000 QALYs (248,000 at a 3% discount rate). The equivalent gains for HCV are 120,000 QALYs over their lifetime, the present value of which (discounted at 5%) is 32,000 QALYs (50,000 at 3%). Discounting has the effect of reducing the present value of gains made in later years relative to those made in earlier years. Consequently, the ratio of the present value of QALYs gained to total QALYs gained for HIV (i.e. 138,072/715,245) is lower than that for HCV (i.e. 32,207/119,992), reflecting the fact that HCV makes a higher proportion of its QALY gains in the earlier years compared to HIV.

5.7 SENSITIVITY ANALYSIS

The QALY adjustment factors used in the analysis of effect of NSPs on the quality of life of injecting drug users have been based on estimates from the literature, as described in Section 5.3.1.

In order to test the effect of different QALY factors on the outcomes demonstrated to date, we have conducted sensitivity analysis by increasing the QALY adjustment factor by 5% across all stages of both HIV and HCV. Such an increase could come about with improved methods of treating each disease, which, while perhaps not altering the rate of disease progression or mortality, improve the quality of life in each stage. The effect of this approach is essentially to reduce the QALYs saved by NSPs, as those who might otherwise be infected by HIV or HCV would enjoy a higher quality of life than under our original assumptions.

The alternative QALY values are shown in Table 5.7.1, and the effects of the application of these values shown in Table 5.7.2.

Table 5.7.1 Alternative quality of life values by disease stage of HIV and HCV

HIV		HCV	
Disease Stage	QALY Value	Disease Stage	QALY Value
Early HIV Disease – Undiagnosed.	0.987	HCV antibody positive – non-chronic hepatitis C.	1.000
Early HIV Disease – Diagnosed.	0.9135	Mild chronic hepatitis – Undiagnosed.	0.987
Progressive HIV Disease – Undiagnosed.	0.945	Mild chronic hepatitis – Diagnosed.	0.861

HIV		HCV	
**Disease Stage**	**QALY Value**	**Disease Stage**	**QALY Value**
Progressive HIV disease – Diagnosed.	0.798	Moderate chronic hepatitis – Undiagnosed.	0.987
AIDS	0.651	Moderate chronic hepatitis – Diagnosed.	0.861
		Compensated cirrhosis – Undiagnosed.	0.882
		Compensated cirrhosis – Diagnosed.	0.777
		Liver failure.	0.336
		Hepatocellular Carcinoma (HCC).	0.105

Table 5.7.2 Net Present Value of QALYs gained for HIV and HCV, alternative QALY values

Discount Rate	Net Present Value, 1991 (QALYs)		
	HIV	HCV	HIV & HCV
5%	129,151	22,603	151,754
3%	235,943	35,528	271,471
0%	692,880	87,118	779,998

The effect of increasing the quality of life adjustment factor for HIV and HCV is to raise the total number of QALYs for people with these diseases, and hence to reduce the QALY gains made by avoiding them. It should be noted that the effect on HCV, however, is greater than that for HIV. This reflects the fact that the major component of the QALY gains made in HIV is derived from an improvement in the quantity of life saved, whereas HCV QALY gains are made up almost entirely of quality of life effects.

In both instances, the gains made in terms of quality of life effects of NSPs in HIV and HCV remain considerable, and reinforce the importance of this aspect of their effect.

5.8 DISCUSSION

The analysis of the effects of HIV and HCV on both the quantity of life and the quality of life of persons with these diseases adds a further dimension to the assessment of the effect of NSPs among injecting drug users. As demonstrated in Section 4, the investment in NSPs to date has been shown to be financially beneficial, and satisfies current government investment criteria on financial grounds alone. Any benefits to consumers in terms of the number of lives saved, the number of life years gained, and the improved quality of life are therefore additional to the direct financial benefits to governments previously identified.

Our analysis demonstrates that NSPs have contributed significantly to:

- The number of cases of HIV and HCV avoided;
- A reduction in the number of deaths from HIV, and to a lesser extent from HCV;
- An increase in the number of life years among injecting drug users, particularly from the avoidance of HIV; and
- An improvement in the quality of life among injecting drug users who would otherwise have contracted HIV or HCV.

Each of these outcomes should be considered over and above the direct financial benefits achieved from the investment in NSPs. It is clear that if we were to place a monetary value against any of these outcomes, the financial gains already demonstrated would be significantly increased.

APPENDIX A

OVERVIEW OF STATE AND TERRITORY NEEDLE AND SYRINGE PROGRAMS

AUSTRALIAN CAPITAL TERRITORY

SNAPSHOT

No of NSPs:	35 (including pharmacies)
Syringes distributed in 1999-2000:	593,000
Approximate Cost:	$539,000

HISTORY AND PROGRAM FRAMEWORK

Needle and Syringe Programs have been operational in the ACT since 1989. The Drugs of Dependence Act 1989 was amended to provide for the licensing of three occupational groupings (doctors, nurses and pharmacists) plus individuals who had successfully completed an authorised training program and who were accredited by the Chief Health Officer. The training program is currently conducted by Assisting Drug Dependence Inc (ADDInc) with funding from ACT Department of Health and Community Care. ADDInc is also contracted to coordinate all other services associated with the operation of NSPs including supply, distribution and returns.

TYPES OF PROGRAMS

As at June 2001, there were 35 NSPs operating in the ACT consisting of:

- 2 primary outlets (ADDInc and Canberra Injectors Network);
- 11 secondary outlets such as government funded health services like community health centres and non Government services; and
- 22 pharmacy based programs.

There is usually a small outreach service provided for sex workers, but this is not operational at this time. The pharmacy program is relatively new, having been established with the assistance of COAG funds some 12 months ago. A person has been employed at ADDInc to coordinate the development and growth of the pharmacy program. Except for this paid position, the program is largely self-funded.

Pharmacy packs (consisting of 4 syringes, 4 ampoules of water, swabs, spoons and a disposal unit) are provided to the pharmacy at a cost of $2 and sold to customers for $4. All pharmacies in the program (along with all other NSP sites in the ACT) provide disposal facilities. However, there is no discount for people who return packs to pharmacies (as is the case in some jurisdictions).

The primary and secondary outlets provide a different type of kit consisting of syringes, water ampoules, swabs, spoons, cotton wool and a disposal unit. All of this equipment is available to users without charge. They also have available other forms of injecting paraphernalia such as butterfly clips and wide boar syringes, some of which is sold at cost recovery rates. The primary outlets provide the full range of ancillary services including information, education, referral, disposal and condoms.

The ACT has a comprehensive needle and syringe disposal strategy. As noted above, all NSPs (including pharmacies) have disposal facilities. This is supported by the following strategies (largely funded by the Department of Urban Services):

- sharps disposal facilities in every public toilet in the ACT;
- a Sharps hotline – a city ranger collects disposed syringes from public land and from private citizens who find a syringe on their land. Businesses are expected to pay for disposal costs;
- training for individuals (such as cleaners) on how to collect and dispose of syringes safely;

- ☐ a depot for bulk waste; and
- ☐ a range of brochures advising “how to dispose safely of used syringes” and “what to do if you find a syringe”. (to be released soon)

BARRIERS AND CHALLENGES

The issues confronting the ACT NSP over the past 12 years are similar to those experienced in other jurisdictions. The primary issue provoking community angst has been the disposal of needles and syringes. The ACT has responded to public concern by strengthening its strategies around disposal. A related issue has been the call for one-for-one exchange of syringes (i.e. that supply of a needle is contingent on the return of a used needle). This has been resisted to date, and its continued resistance largely rests on the political will of the ACT Legislative Assembly.

An interesting historical event was the move towards the provision of a greater range of injecting paraphernalia in each pre-packed kit that was distributed. The rationale for this was the risks of transmission of hepatitis C in ways other than through sharing syringes. Some consumers did not like the new packs as they were too big and bulky. NSPs received strong negative comment about the packs and subsequently streamlined the packs.

It is expected that the issues of disposal and one-for-one exchange will continue to arise on a periodic basis. In addition, there has been some discussion about a move towards a user-pays system in the NSP’s primary and secondary outlets (as is already the case in community pharmacies).

The other issue that is of some import is after-hours access. Only one service (Calvary Hospital) provides FitPacks after 9pm and is not centrally located. ADDInc is currently negotiating with other potential hosts with a view to expanding the range of after-hours services.

NEW SOUTH WALES

SNAPSHOT

No of NSPs:	797
Syringes distributed in 1999-2000:	11,566,000
Approximate Cost:	$10,290,000

HISTORY

In 1987, the NSW government legalised possession of needles and syringes by amending the Drug Misuse and Trafficking Act 1985. A subsidised pharmacy program (the Pharmacy Distribution Scheme, later renamed the Pharmacy Fitpack Scheme) was funded in 1986 with the objectives of increasing access and encouraging the exchange of used syringes for new ones.

The public sector Needle and Syringe Program commenced in NSW in 1986 with a pilot program. In 1988, the program was rolled out across the state on an expanded pilot basis, with a focus on access, education, consumer involvement and the free supply and exchange of equipment.

Although possession of needles and syringes was legalised in 1987, possession of other items used to administer prohibited drugs, including other items of injecting equipment, remains illegal. Pursuant to the Act, the Director-General of Health is empowered to authorise a specified person or a specified class of persons to lawfully dispense needles and syringes (supply of which would otherwise be an offence). Two classes of agency can apply for approval to operate as an NSP. namely non-government organisations and public health sector

agencies. Approval is ultimately considered by the Chief Health Officer (NGO's) or Chief Executive Officer or Director (PHSA's).

Responsibility for statewide policy and planning of the NSP rests with the AIDS/Infectious Diseases Unit of the NSW Health Department. Responsibility for service delivery and operational matters is devolved largely to the state's 17 Area Health Services and, to a lesser extent, to a small number of community-based NGO's.

A recent significant event was the NSW Drug Summit, held in May 1999, which resulted in many progressive outcomes for the management and operation of drug-related programs in NSW. In the case of the NSP, the main benefit of the Drug Summit was that it demonstrated that there was broad support for the program and recognition of the need to expand the program commensurate with demand.

TYPES OF PROGRAMS

NSW has significant numbers of all types of NSPs. It has 34 primary outlets, 282 secondary outlets, 28 outreach services, 56 vending machines and 397 community pharmacy outlets. Within each of the 17 Area Health Services, there is usually at least one primary outlet, often incorporating an outreach component, and a range of secondary outlets which are usually located within community health services, hospital emergency departments and local NGOs This 'hub and spoke' type of approach to NSPs means that the local primary outlet provides support, training and supply of equipment to the secondary outlets within the same area.

There is a small number of primary NSPs operating through non-government organisations, some of which operate in a limited way or cater to a specific target population.

All primary, secondary and outreach NSPs provide needles and syringes, Fitpacks or other disposal containers and other injecting equipment (such as swabs, water, spoons and cotton balls) free of charge. Community pharmacies, operating as part of the Pharmacy Fitpack Scheme, supply Fitpacks that contain either 3, 5 or 10 syringes for a cost of around $3.30 or free if the customer has an exchange. The pharmacies are provided the equipment, through the NSP program at no cost. Vending machines mostly supply a 5 pack with water and swabs for a cost of up to $3 ($1 and $2 being most common).

Injecting equipment is also sold through about 400 non-participating pharmacies on a purely commercial basis. A 1 mL insulin syringe costs an average of $1.50 through these pharmacies, although prices of up to $4.50 for a single syringe have been reported.

All NSPs, including pharmacies, operating as part of the Pharmacy Fitpack Scheme, have disposal facilities provided as part of the program. In addition, a Needle Clean Up Hotline was introduced in 1997 in response to community disquiet generated through disposal of syringes. Whilst the initiative has had relatively modest resources committed, it appears to have significantly reduced visible community complaints about discarded needles. The program is now working on a strategy to further improve local responses to discarded needles by increasing the commitment and involvement of the local government sector.

BARRIERS AND CHALLENGES

The early days of the program in NSW were characterised by a significant level of community acceptance in many areas and support from a number of key sectors, most notably the police. In more recent times there has been an erosion of bipartisan political support and the emergence of concerted, organised opposition from particular groups including some local government bodies. During the recent increase in heroin use, street-based drug activity became much more visible in a number of areas, leading to a marked increase in community anxiety. The issue of needle and syringe disposal continues to be the major source of community dissatisfaction with the program.

As a result of the Drug Summit in 1999, a range of new initiatives are now being trialed, including the medically supervised injecting centre in Kings Cross. Following the summit, it seems likely that there will be support for

further development of harm reduction programs in NSW (in contrast to the fight to simply preserve the status quo evident in some other states).

Currently the NSP appears to be limiting injection-related transmission of HIV to a very low level and this seems likely to continue for the foreseeable future. The main challenge to this relates to the possibility of outbreaks of HIV infection among particular populations with high levels of risk behaviour, including some indigenous populations and prison inmates. Ensuring effective access to prevention services for high-risk groups of this kind is a priority at this time.

The second major challenge is to achieve a significant reduction in hepatitis C infections. The extent to which further development of the NSP will enable this to be achieved is difficult to predict. Community understanding, acceptance and support for the NSP will probably be a key factor in determining whether such development occurs. Therefore, a key challenge now is to work out how to achieve a much higher, sustainable level of community support.

NORTHERN TERRITORY

SNAPSHOT

No of NSPs:	19 (2 primary, 5 secondary, 12 pharmacies)
Syringes distributed in 1999-2000:	604,000 (Est)
Approximate Cost:	$373,000 per annum (Est.)

HISTORY

Needle and Syringe Programs have been operating in the Northern Territory since in 1989, when the Northern Territory AIDS Council (NTAC) and the AIDS Council of Central Australia (ACOCA) were established. In late 1990/early 1991, the distribution of needles and syringes through pharmacies in Darwin commenced. The Misuse of Drugs Act 1990 requires the licensing of positions within particular agencies to distribute needles and syringes. To date, 19 licenses have been granted. In addition, all medical practitioners and pharmacists are automatically authorised to distribute needles and syringes.

Injecting drug use in the Northern Territory differs significantly from other states and the ACT. The most commonly injected drugs in Darwin are prescribed opioids (particularly morphine) and amphetamines. Supply of morphine has recently shrunk due to the regulation of the distribution of morphine through General Practitioners. This pattern is different in Alice Springs where the supply of heroin is more common.

TYPES OF PROGRAMS

The two AIDS Councils (Darwin and Alice Springs) are the primary NSP outlets in the Northern Territory. Between them they distribute the majority of the 500,000 needles and syringes disseminated through NSPs in the NT each year. Supplementing the primary outlets are five secondary outlets operating through NT Health Service's sexual health clinics and Accident and Emergency Departments at each of the hospitals (located at Darwin, East Arnhem Land, Catherine, Tenant Creek and Alice Springs).

In addition to needles and syringes, primary outlets provide swabs and disposal units. Other items (such as condoms and injecting paraphernalia) may be provided on a cost-recovery basis through the core budget of the AIDS Councils. The services provided by the primary NSPs include education, advocacy, disposal, referral and support.

Needles and syringes are also distributed through commercial pharmacies. On commercial terms, pharmacies in the NT purchase "fit kits" from a third party source (either NT AIDS Council or suppliers from other states). These kits (consisting of 5 syringes and a disposal pack) are purchased for around $3 and sold through pharmacies for between $4 and $6. It is not known how many of these kits are sold annually, but it is estimated to account for an additional 5-10% per annum (perhaps as many as 50,000 syringes).

The NT AIDS Council and Darwin City Council have collaborated to provide 10 disposal units in the Darwin area. The first of these was installed into the Casuarina Library in 1998. Prior to that, the only disposal facilities were located at NTAC and the Darwin Airport (men's toilets). Disposal units are also located in the male and female toilets at Alice Springs Airport.

BARRIERS AND CHALLENGES

Like many other jurisdictions, NSPs in the Territory are a highly political issue and public perceptions have some influence over policy decisions. In recent times, increased cost recovery has been looked at and, accordingly, consideration is being given to strategies to increase the role of pharmacies as a major point of needle and syringe distribution. The other significant issue is that of disposal. On a regular basis the problem of needle and syringe disposal emerges and community debate ensues. The NT is currently developing a database to identify disposal hotspots. Contributing to the disposal issue is the fact that no pharmacy currently accepts used injecting equipment. Steps are being taken to provide bulk disposal facilities.

The legislative framework in the NT provides that needles and syringes can only be issued by licensed positions (as described above). This creates a difficulty when people in authorised positions are unavailable.

COAG funding has been provided for the establishment of an NSP in the suburb of Palmerston in Darwin. Prior to the NT Government issuing a license, it has required that the Palmerston Council support the new NSP. This support has not yet been forthcoming and, accordingly, the Palmerston NSP is yet to commence.

QUEENSLAND

SNAPSHOT

No of NSPs:	132 (+800 estimated pharmacies)
Syringes distributed in 1999-2000:	5,300,000 (+5,000,000 estimated through pharmacies)
Approximate Cost:	$1,678,000

HISTORY

The enabling legislation for Needle and Syringe Programs in Queensland was enacted in the Drugs Misuse Act (as amended) 1989. The amendment allows for the supply of needles and syringes to any person for any lawful purpose. It also allows for the supply of needles and syringes to any person by medical practitioners, pharmacists and persons approved by the Minister of Health for the purposes of illegal drug use. This requires the Minister of Health to approve all staff or staff positions involved in NSPs. To obtain approval, staff are required to attend and successfully complete a 4-hour training program, upon which their names or positions are submitted to the Minister for final approval.

Prior to 1989, single syringes were sold commercially through pharmacies. The original Queensland NSP (known then as the Statewide HIV/AIDS and IDU Program) was located within the Sexual Health Unit of Communicable Diseases. In 1997, following a review of needle and syringe availability, the management of NSPs was shifted to Alcohol, Tobacco and Other Drugs Services.

Distribution of needles and syringes has tripled over the past six years (1996-2001). There has been an explosion of amphetamine use over this time (there are now an estimated 85,000 amphetamine users in Queensland) and it is likely that many people who use amphetamines are injecting on a casual basis. Use of heroin is not as dominant as it is in some other states; even so it is estimated that Queensland has approximately 17,000 heroin-dependent persons.

TYPES OF PROGRAMS

Queensland has 132 needle and syringe programs currently operational, which distribute around 5,000,000 needles and syringes per annum. The program has two primary Queensland Health operated sites (Brisbane and Cairns) and 130 secondary and NGO sites. In addition, outreach services are provided through some of the NGOs. Brisbane City Council and Queensland Health are in the process of establishing an after-hours street-based service to distribute equipment within the Fortitude Valley area. Biala, located in Brisbane, is the largest NSP in Australia and accounts for approximately 34% of the total number of syringes distributed in Queensland each year. It is a 24hour/7days per week alcohol and drug service with a full range of services including a methadone clinic and an AIDS medical unit. It is Queensland Health policy that every health service district must provide at least one NSP within its area. In addition, Accident and Emergency Services in all publicly funded hospitals are strongly encouraged to operate NSPs. However, some hospitals do not operate NSPs, while others operate the program without uniformity of service or with little enthusiasm.

Like programs in all states, the range of activities that each service engages in is commensurate with their degree of specialisation. The primary sites, NGOs and larger secondary sites provide a comprehensive range of services (greater range of injecting paraphernalia, provision of condoms, BBV testing, referrals, support and information), whereas the smaller programs are more likely to only provide basic services.

In addition to the provision of equipment, all Queensland Health NSPs provide used sharps collection and disposal facilities. Brisbane City Council has also recently installed 300 disposal bins in and around the city. Many other local councils have already, or are in the process of installing sharps disposal bins in appropriate locations.

The legislation automatically authorises all pharmacists to sell syringes. There are approximately 1,000 retail pharmacies in Queensland and it is estimated that around 80% of them sell needles and syringes at a retail price of between $2 and $5 for a five pack (average price is $3). It is estimated that these pharmacies distribute about 5,000,000 syringes per annum (approximately the same number as the NSP). However, as this commercial operation takes place without any government involvement or regulation, it is difficult to determine exact figures. Queensland Health is in the process of talking directly with wholesalers of injecting equipment to obtain more accurate and detailed information.

BARRIERS AND CHALLENGES

From the outset of operating NSPs in Queensland, the program has experienced a significant level of public antagonism. This is well illustrated by the recent call from the Northern Queensland Local Government Association for the banning of NSPs. As a result, NSPs are continually defending their position and are operating from a position of reactivity rather than acting in accordance with sound evidence-based practice.

The major issue for action in Queensland, is the safe disposal of used needles and syringes. Although 80% of pharmacies sell syringes, almost none accept returned sharps. Queensland Health is currently exploring options with the Queensland Pharmacy Guild to encourage pharmacies to provide sharps disposal facilities. In addition to pharmacies, Queensland Health is working in partnership with local government to deal with disposal issues. This has resulted in the creation of a collaborative project between Queensland Health and the Local Government Association of Queensland within the framework of the Queensland Public Health Partnership Protocols. The first concrete result being the establishment of an 1800 State wide clean needle helpline.

SOUTH AUSTRALIA

SNAPSHOT

No of NSPs:	151
Syringes distributed in 1999-2000:	3,018,000
Approximate Cost:	$830,000

HISTORY

In South Australia, the "Clean Needle Program" (CNP) is operated by the Drug and Alcohol Services Council (DASC) within a licensing framework. The legislation (the Controlled Substances Act) provides for trained agency representatives to possess and distribute needles and syringes in compliance with agency protocols.

The Clean Needle Program commenced in South Australia in 1989. The first fixed-site services were located at SAVIVE (South Australia Voice for IntraVenous Education) and Warinilla (a drug treatment service). The pharmacy program commenced in SA in the early 1990's.

TYPES OF PROGRAMS

Of the 151 NSPs in South Australia, there is 1 primary outlet (SAVIVE), 66 secondary outlets (located in hospitals, community health centres and youth services) and 84 community pharmacies (from a potential pool of 380 pharmacies). An informal outreach service is provided by SAVIVE, via their peer educators' informal network. A vending machine was installed at SAVIVE, but was vandalised and is no longer operational. An issue was raised about the appropriateness of vending machines given that they only disseminate equipment and do not provide users with information about safe using.

Of the primary and secondary outlets, 32 are based in the metropolitan area of Adelaide and 34 are in rural areas (mainly in country hospitals). The absence of any metropolitan hospitals from the program means that there are no 24-hour services in Adelaide, although there is 24-hour access through the majority of country hospitals.

The community pharmacy program is run on a partial cost-recovery basis. The program supplies community pharmacies with Fitpacks at no cost and charges these pharmacies (at cost price) for syringes supplied ($0.1386 per syringe). Pharmacies sell Fitpacks to consumers at a cost of $5 for a 10 pack and $4 for a three pack. If consumers return a Fitpack, they receive a $2.00 discount on a new pack. Currently, the syringes for the Fitpacks are supplied in bulk through the program, with pharmacies being required to assemble the packs themselves. This element of the program has been identified as having an impact on pharmacists' willingness to participate in the program, and is currently being addressed.

In addition to pharmacies that participate in the Needle and Syringe Program, other pharmacies sell needles and syringes on a purely commercial basis. They order equipment (such as the SK-3) from pharmacy wholesales and retail them at whatever price they consider appropriate. As these transactions are outside the bounds of the program, there is very little information available about quantity and price.

Disposal facilities are provided through the program at all NSP sites, including community pharmacies. Some local councils also provide disposal facilities at various sites such as public toilets and council offices. SA has recently launched a Needle Clean Up Hotline, operated through the Alcohol and Drug Information Service (ADIS), whereby members of the public can report syringes found in public places.

BARRIERS AND CHALLENGES

Access to NSPs in some locations (some country areas have resisted the introduction of an NSP into their community) and on a 24/7 basis in Adelaide have been issues for the program over the past few years. Only two sites in Adelaide provide after-hours access (at Hindmarsh and Norwood) and neither of these sites are open 24 hours. Encouraging metropolitan hospitals to participate in the program is identified as a major challenge for the immediate future.

There have been several barriers to the involvement of pharmacies in the program, including issues relating to clients' behaviour in the pharmacy (people accessing the methadone program rather than the NSP are not differentiated) and the time-consuming process of assembling the Fitpacks. One of the objectives for the program in SA is to increase the number of community pharmacies participating in the program and work has already commenced through the Pharmacy Guild (SA Branch), to further that objective. An initiative that is being pursued through the community pharmacy program is the provision of information and linkages with other services.

There is a reasonable level of political and community goodwill towards the program and it seems to have been relatively immune from the attacks that the program has faced in some other states.

As distribution of equipment increases, so do the costs of running the program. There is a desire to increase the range of equipment that is available through the program (including spoons and filters), but it is recognised that this will further exacerbate the problem of rising costs.

TASMANIA

SNAPSHOT

No of NAPs:	88
Syringes distributed in 1999-2000:	1,381,000
Approximate Cost:	$622,000

The program in Tasmania is referred to as the "Needle Availability Program ("NAP").

HISTORY

In 1993, the Tasmanian Parliament passed the HIV/AIDS Preventive Measures Act, which established the regulatory framework for NAPs. Prior to this time, there had been some informal (and unlawful) dissemination of needles and syringes by individuals committed to preventing the transmission of HIV. The Act declares that permits are required to operate an NAP and identifies the basis upon which permits can be issued. Agencies wanting to run an NAP apply for a permit, nominate the individual (s) who will disseminate the equipment and have the conversation with the clients, attend education and training sessions etc. Once the permit is granted, the individual (permit holder) can delegate these powers to others, should they be unable to undertake them themselves.

The management of the NAP has always rested within the Sexual Health Branch under the Division of Health Advancement within the Department of Health and Human Services. There have been some suggestions that it should be moved to Drug and Alcohol Services within in the Department of Health and Human Services. This has, however, been resisted, on the basis that the focus of the program must be harm reduction and this is in sometimes in contrast with the philosophy surrounding the Alcohol and Drugs Services' approach to drugs.

The most commonly injected drugs in Tasmania vary between regions. Clients indicate, via a data-collecting tool administered to clients each time they access a NAP, that in Hobart the drugs mostly commonly injected are morphine or methadone; in Launceston it is morphine and amphetamines; and on the north-west coast, amphetamines are the drugs mostly injected. In 1999/00 only 4.3% of injecting drug users in Tasmania indicated that they mostly inject heroin.

TYPES OF PROGRAMS

Tasmania has no primary outlets (its largest NAP is located within the Tasmanian Council on AIDS and Related Diseases TasCAHRD), 28 secondary outlets (disseminating approximately 80% of all syringes) and 60 pharmacy-based outlets (disseminating approximately 20% of all syringes).

The secondary outlets are co-located with a range of services including hospitals, community health services, youth health and drug and alcohol services. There are a few 24/7 services, located within Accident and Emergency Departments in hospitals. Unfortunately, this is not the case in either Hobart or Launceston, although in Hobart a 24/7 Alcohol and Drug Detoxification Unit is involved in the program.

The secondary outlets order injecting equipment from a Medical Supply Company with which the Tasmanian Department of Health and Human Services ("DHHS") has a contract. The equipment is available to the NAP at no cost, but the outlets do not receive funding to provide the NAP service nor any ancillary services (such as disposal, referral, information, staff etc).

Pharmacy-based outlets provide equipment to clients for a fee. They order equipment in the same manner as secondary outlets and, similarly, are not charged for the supply of this equipment. Unfortunately, due to a range of factors, the variety of equipment available through the pharmacy-based outlets is usually limited to 1mL Fitpacks.

The standard prices that are charged for equipment are $6 for a 10 pack (10 syringes, 10 swabs, 10 water ampoules, Fitpack10 disposal unit) and $4 for the 3 pack (3 syringes, 3 swabs, 3 water ampoules and FitPack3 disposal unit). If the client returns a FitPack they are able to purchase a 10 pack for $3 and a 3 pack for $2. If however, they cannot afford to pay, they will not be refused equipment. The Department of Health and Human Services has an agreement with pharmacies that was written 10 years ago. A Memorandum of Understanding is now being negotiated between the Department of Health and Human Services and the Pharmacy Guild of Australia (Tasmanian Branch) to clearly document the roles and responsibilities of both Government and Pharmacies in the NAP. All pharmacy-based outlets provide disposal facilities for returned equipment at their own cost.

NAPs provide an extensive range of equipment including different types and sizes of needles (18-30 gauge), different types and sizes of syringes (1mL-20mL), a range of sharps containers (1.4-68 litre), water swabs, FitPacks (3 and 10), insulin syringes (27 and 29 gauge). Outlets are able to order whatever equipment they believe their clients need, and package it in appropriate ways. In other words, there is no standard "pack" provided through Tasmanian NAPs. Secondary outlets do not charge consumers for any of the equipment that they supply through the NAP.

In addition to the pharmacy-based outlets participating in the NAP (approximately 50% of all pharmacies in Tasmania), other pharmacies sell syringes on a purely commercial basis.

BARRIERS AND CHALLENGES

The biggest barrier that has been faced in Tasmania, like most jurisdictions, is the political sensitivity of needle and syringe programs. As a result, the government has chosen to take a low key, discrete approach to the operation of NAPs.

Some government health agencies, although well positioned to host an NAP (such as large metropolitan hospitals and community health centres), have declined invitations to be involved. They appear not to have been further encouraged by respective Ministers.

Another issue has been the trend away from bipartisan political support for the NAP in Tasmania since the last change of government. This has led to the NAP being used for political purposes, rather than being recognised as an important public health initiative. It is anticipated that this push to dampen down public health initiatives could have significant future consequences for the program.

As a result of the continual increase in the number of needles and syringes distributed, the costs of running the Program are also increasing. This has led to varying suggestions for strategies including a review of the program and a user-pays program.

VICTORIA

SNAPSHOT

No of NSPs:	215
Syringes distributed in 1999-2000:	6,177,000
Approximate Cost:	$4,767,000

HISTORY

Needle and Syringe Programs commenced in Victoria with four pilot programs in 1987. In 1988, the program was expanded state-wide. NSPs are governed by the Drugs, Poisons and Controlled Substances Act 1981 (Vic) which provides an exclusion for people authorised by that Act from the laws governing aiding and abetting illicit drug use. The Act enables the authorisation of agencies wanting to host an NSP (and records the agency name, positions, roles and hours of operation), as well as a general authorisation for pharmacists and pharmacy assistants. Once authorised by the Minister for Health, any changes within a service (eg hours of operation) must be notified to the Department. The requests for authorisation are considered by the Department, prior to making recommendations to the Minister. The Department gives consideration to such issues as consultation with the local community, the proposed location, training and general understanding of the operations of an NSP.

Until the most recent change of government (in December 1999), the NSP administration was located within Communicable Diseases Branch of Public Health. It is now located within the Drugs Policy and Services Branch of Victorian Department of Human Services.

Reports indicate that the main drug being used in Victoria is heroin, although in its absence (such as during the recent heroin drought), amphetamine use increases.

TYPES OF PROGRAMS

Of the 215 registered programs in Victoria, 14 are primary outlets (fully funded through the program), 180 are secondary outlets (which provide consumables, written resources and training) and 22 are pharmacy-based programs. In addition, there are 3 enhanced secondary outlets, located in drug hotspots, which are funded for some staff time, some disposal facilities and some community education and promotion.

Pharmacy involvement in the government-auspiced program is fairly limited, but other pharmacies do sell equipment. Those participating in the NSP program receive consumables for free and are supposed to provide it

to users free of charge (which removes the profit incentive for pharmacy involvement). The Department operates the ordering and supply process for all NSPs.

The equipment supplied through the NSPs includes needles, syringes (in a variety of sizes), swabs, condoms, lube, disposal units, plastic bags and printed materials. They do not currently supply water, spoon and filters, but consideration is being given to the possible provision of these items. There is some variation in what is supplied in a standard kit from NSPs as some choose to make up their own packs.

With respect to outreach services, there are two foot patrols, one of which operates on a fixed route. One foot patrol works within the CBD during the day and night. Another foot patrol, in the Springvale area, operates during the day only. In metropolitan Melbourne, there are also seven car-based outreach programs operating each evening, seven days a week, until about 11 pm. There are two car-based outreach programs operating during the day.

Another outreach project in Victoria is the steroid project, which employs a worker to provide information about safe using and consumables to users of anabolic steroids. This project was piloted in 1996 and has been running since 1997.

All needle and syringe programs have disposal facilities supplied and maintained by Departmental funding. A Helpline has also been established to provide advice and referral to people who find a syringe that has been disposed of. If there is an agency in the area that has a collection service, the referral is made to that agency (eg Fitzroy NSP or a local government authority). Otherwise the person is given information about how to pick up the syringe safely. In addition, many local councils have installed disposal units in locations such as public toilets.

BARRIERS AND CHALLENGES

Like most jurisdictions, disposal incidents have plagued the operations of the NSP. The highest profile of these incidents involved a well-known triathlete who received a needle stick injury from a discarded needle on St Kilda beach. This resulted in huge media coverage and public outrage. There is a constant level of public concern about NSPs and a lack of understanding about the rationale for such programs. Like other states, the name of the program was changed (by the deletion of the word ‘exchange’) to reduce expectations that NSPs achieved or should achieve a 1-for-1 exchange of equipment.

The Minister for Health launched a ‘Safe Needle Disposal Strategy’ in January 2001 that includes the establishment of the HelpLine and a number of strategies relating to surveillance, best practice disposal and retrieval and working with local government to improve and expand retrieval services. A Monitoring Group with representation from NSPs, users, local government, pharmacies, public transport, police and epidemiology has been established to oversee the implementation of the strategy.

The role of NSPs within mainstream health services has also been a constant issue. It is not widely accepted that the provision of an NSP is part of the public health role of these services, and so the introduction of NSPs has been resisted by some health workers.

One issue that has not been raised in other jurisdictions is that of planning issues for local government that arise when application is made for planning approval for a needle and syringe program. Negotiations are currently underway to develop guidelines that will clarify this process.

Current debates and anticipated future issues in Victoria include the call for the use of retractable needles, educating the public about the real risks of transmission of communicable diseases as a result of a non-occupational needle-stick injury (to reduce concerns about needle and syringe disposal) and an apparent increase in the intensity of media sensationalism about NSPs.

Another issue of concern in Victoria relates to the requirement for the legalisation of possession of used equipment. Whilst it is not illegal to possess used equipment, the equipment and its contents can be seized,

tested and used in evidence to support other charges. This is reported by NSPs as being a barrier to appropriate disposal such as carrying a container of used syringes to an NSP and is likely to contribute to the immediate disposal of equipment at the location of its use (i.e. inappropriate disposal).

WESTERN AUSTRALIA

SNAPSHOT

No of NSPs:	80 (plus pharmacies)
Syringes distributed in 1999-2000:	3,209,000
Approximate Cost:	$3,576,000

HISTORY

The Poisons Act 1964, was amended in 1994 to create a legal defence for persons participating in approved needle and syringe programs. The power to approve applications from organisations wishing to be authorised pursuant to this Act is vested in the Commissioner of Health, Health Department of WA. Legislatively, responsibility for the administration of needle and syringe programs in WA is carried by the Chief Pharmacist (Environmental Health: Drugs Poisons and Therapeutic Goods Control Section), Health Department of Western Australia. However, the Sexual Health Program, Communicable Disease Control Branch, Health Department of Western Australia plays a major role in liaison with service providers and providing statewide coordination of the program.

Until 1994, the provision of injecting equipment to people who were injecting drugs illicitly was illegal, and providers of needles and syringes could, as the law stood, have been charged with an offence under the Western Australian Criminal Code. Despite this, pharmacists and other services provided sterile needles and syringes to injecting drug users as a critical public health strategy in the prevention of further transmission of HIV. The Poisons Act amendments of 1994 led to an increase in the number of needles and syringes distributed and the number of outlets. NSPs first commenced in WA in 1987, with the Health Department of Western Australia administering a program providing sterile injecting equipment to injecting drug users (IDUs) in “SS5” kits (consisting of a disposal container, 5 sterile needles and syringes, condoms, and information pamphlets). These were distributed through the Western Australian AIDS Council and other agencies, and retailed through pharmacies. In July of 1992, Fitpacks⊇ were introduced to replace the SS5 kits.

TYPES OF PROGRAMS

WA has one dedicated fixed-site primary needle and syringe exchange, three mobile outreach exchange services, 75 secondary outlets, and one vending machine. The primary site is operated by Western Australian Substance Users’ Association (WASUA) and is located in Northbridge (Perth). The Western Australian AIDS Council (WAAC) operates two mobile services from a range of different sites in Perth and a new mobile program operated by WASUA has been established in the regional centre of Bunbury (funded through COAG Diversion Initiative). Secondary outlets are provided through other services such as hospitals (41 outlets), health units, nursing posts and community health centres. They account for less than 5% of total needle distribution in WA.

Two vending machines had been installed at a drug and alcohol service operated by the Health Department, but they are currently non-operational due to vandalism. When operational, the vending machines were disseminating just over 1% of all needles and syringes. A trial of a vending machine commenced operation at Kalgoorlie Regional Hospital in March 2001. In the first eight weeks of operation, similar numbers of Fitpacks⊇ have been vended through the machine at cost of $3.00 as were given out at no cost over the counter of the

Accident and Emergency Department for same time period last year. The machine has to date not been vandalised or broken into.

The operations of NSPs in WA are significantly different from those in other states in several significant ways. Firstly, approximately sixty-five per cent of needles and syringes distributed in WA are sold through pharmacies. Secondly, the two services that offer exchange do so on a cost-recovery basis, that is, a charge of 25c per needle if there is no exchange.

The pharmacy program is commercial in nature (equipment is ordered directly from the pharmaceutical wholesaler and sold at a price determined by the pharmacy). The Health Department of WA supplies information labels that are attached to the Fitpacks⊇ at the point of manufacture and packing (i.e. prior to dispatch to the pharmaceutical wholesalers). Under a collective licence held by the Pharmaceutical Council of WA, pharmacists are licensed to sell only Fitpacks⊇, Fitpack⊇ Plus and Sharpkitz, and most commonly retail a combination of these products. Fitpacks⊇ consist of 5 syringes in a disposal unit (average price 5.50), whilst Fitpacks⊇ Plus contain 3 syringes, water, swabs and spoons in a disposal unit (average price $6.50). Prices for each product vary significantly between outlets (including across location and hours of operation). There are no return discount or exchange, nor disposal facilities provided through pharmacies. There are a few pharmacists that have applied for individual licenses to allow them to sell loose needles and syringes with a disposal container.

The Health Department of WA includes safe disposal messages on all Fitpack⊇ labels, and has produced a pamphlet to inform the general public on how to appropriately dispose of used needles and syringes they may find discarded. Further, HDWA has made available to local government authorities needle and syringe disposal bins for installation in their public amenities. Maintenance of the bins is the responsibility of the local government authority. The WASUA and WAAC NSPs provide disposal facilities for used equipment.

A demonstration project involving WA Drug Abuse Strategy Office, WA Police Service, HDWA, local governments (Town of Vincent and City of Perth) and WASUA has developed strategies and resources to educate both the general public and people who inject drugs in the practice of safe disposal of used injecting equipment.

Barriers and Challenges

Historically, public and political support for the program has been one of the most difficult barriers to overcome. There has been a level of recognition of the need for the program from the government, but this has fallen short of making public statements to that effect. The media occasionally seizes on events such as needle and syringe disposal and makes a significant public issue out of it. There is a minority within the community who voice marked opposition to the program.

The major issues in WA for the NSP are those of access and equity. The sheer size of the state and the distribution of the population across the state make access for many people problematic. There is now a reasonable coverage of NSPs and/or participating pharmacies across WA, but there are some areas (such as some mining towns) where injecting drug use is known to be occurring but the supply of injecting equipment is limited. 24-hour access also poses an issue, as there is only one 24-hour pharmacy based in Perth and some of the major regional centres do not have 24-hour outlets (NSPs or pharmacies). Historically, there was an informal agreement between the Health Department and the Pharmacy Guild (WA Branch) that the Department would not establish an NSP in an area during pharmacy trading hours. This agreement is now being revisited as it is restricting access in some locations.

Equity of access is an issue in WA more so than in any other state as it is the only state that operates its NSPs largely on a cost-recovery basis and its pharmacy program is commercial. This means that people in areas not serviced by an NSP can only access Fitpacks⊇, at full retail price (around $5 to $6 in rural areas and $5 to $7 in metropolitan areas) and this is likely to have an impact on people with lower incomes. As noted above, the only 24-hour service in Perth is located in a pharmacy. This raises an issue of after-hours access not only for people on lower incomes and others who are price sensitive, but also for those who live in outer metropolitan areas, and

does not allow for choice in mode of service provision. These issues are currently being considered in WA, whilst recognising the costs involved in adding more NSPs to the current service system.

The WA Health Department is conscious of the advantages of having such a successful pharmacy program at a very low cost, but also recognises the dilemmas that have been created through the agreement with the Pharmacy Guild at the inception of the program. This issue is in the process of being explored with a view to widening access and the availability of needles and syringes.

APPENDIX B

DISCUSSION OF THE METHODOLOGY USED IN THE ECOLOGICAL STUDY

The effectiveness of NSPs reducing HIV incidence was estimated by first fitting for each city a linear regression line, on a logit scale, to give an estimate of the overall average annual rate of change of prevalence for each city. Effectiveness of NSPs was then estimated by comparing those cities which ever had NSPs with those cities which never had NSPs.

The justification for this approach lies in the fact that an estimate for HIV incidence in a given city can be taken approximately to be the change in prevalence. Fitting a simple linear regression line, over the entire available time period, for each city therefore gives an estimate of the average annual HIV incidence for that city over the whole available time period. Regression lines were fitted on a logit scale to avoid problems with fitted lines becoming negative or greater than one. There are several advantages to this approach:

1. This methodology was adopted in a peer-reviewed publication in a top-ranking medical journal, and as such represents the most widely accepted standard approach (Hurley et al. 1997).
2. The approach is simple, transparent, unbiased and clearly defined. Fitting a simple linear regression line can be done for each city in a completely consistent fashion without introducing any arbitrary decisions into the fitting procedure which would need either further data or assumptions to be made, and which could introduce biases. The approach also gives a well-defined, single summary statistic, namely the rate of change in HIV prevalence (the slope of the regression line) which can be compared between cities with and without NSPs.
3. The approach is robust in the sense that a simple linear regression gives an overall average estimate of annual change in HIV prevalence over the entire time period considered.
4. The approach is conservative in that by comparing cities which never had NSPs with cities which ever had NSPs, the effectiveness of NSPs will tend to be underestimated.

There are however some possible problems with this approach. First, the estimated annual rate of change in HIV prevalence for a given city will depend upon where in the HIV-epidemic natural history HIV-prevalence surveys were first conducted. In cities which started surveys after a HIV-epidemic was established among injecting drug users (IDUs), HIV prevalence would tend to be high initially, and fitted slopes therefore somewhat low. In cities which started surveys before an epidemic was established, there is the potential for much larger fitted slopes. Furthermore, the effectiveness of NSPs in reducing established HIV-epidemics might not apply to the effectiveness of NSPs in preventing HIV-epidemics among IDUs. In Australia, NSPs were introduced while HIV-prevalence among IDUs was low. The extent to which estimates of NSP effectiveness based on changes in HIV-seroprevalence from all cities, including those cities with HIV-epidemics which were well established, should be applied to Australia is arguable. In order to address this point, secondary analyses estimating the effectiveness of NSPs based only on those cities which had an initial HIV-seroprevalence estimate below 10% were conducted, corresponding broadly to the state of the HIV-epidemic among IDUs in Australia. These analyses gave qualitatively very consistent results to the overall analysis including all cities (see Table 3.1.2), suggesting that the results are relatively robust to this point.

A second criticism of the approach is that fitting simple linear regression lines to each city’s HIV-seroprevalence survey estimates is unnecessarily simplistic. It may be that there is a general form to the HIV-epidemic natural history among IDUs, with a rapid increase in seroprevalence early in an epidemic, followed by a plateau as prevalence attains high rates, and possible even a gradual decline as other preventive measures, such as general HIV-education programs, are introduced. A much better fit to the data in each city might be achieved by fitting a more flexible regression curve, for example introducing a quadratic term. An estimate of the rate of change in HIV-prevalence for each city could then be obtained by choosing a time-period, for each city, which corresponded to the epidemic in Australia, and using the fitted curve at the start and end of this period to estimate an average annual rate of change during the period. There are two disadvantages to this procedure. First, the choice of curve fitted to each city becomes somewhat arbitrary. Second, the choice of time period for each city corresponding to the epidemic in Australia would have to depend on further data (which in this case were unavailable) or further assumptions, which could be used to introduce bias into analyses. For these reasons, estimates of NSP effectiveness on reducing HIV incidence were based on the simple linear regression approach described above. However, more complex curve fitting procedures might be the subject of future research.

APPENDIX C

DETAILED TABLES OF EFFECTS OF NSPS ON HIV AND HCV

Table 3.4.1 Estimated number of people living with HIV/AIDS and total deaths with NSP introduction[1]

Year	HIV/AIDS[2]	HIV CD4 >500	HIV CD4 <500	AIDS	Total deaths[3]
1981	2	2	0	0	0
1982	7	7	0	0	0
1983	23	22	1	0	0
1984	60	57	2	1	0
1985	121	112	8	1	0
1986	201	180	18	3	1
1987	284	241	36	7	3
1988	355	281	63	12	7
1989	409	293	97	18	13
1990	445	284	135	26	21
1991	466	260	171	34	33
1992	474	229	202	43	49
1993	471	195	225	50	68
1994	465	168	240	57	91
1995	456	147	247	61	116
1996	452	133	256	64	137
1997	449	123	262	65	157
1998	449	116	267	66	176
1999	450	112	272	66	193
2000	453	109	279	65	208
2001	433	84	285	63	223
2002	413	62	289	62	238
2003	394	43	290	61	253
2004	375	29	286	59	267
2005	356	19	279	58	281
2006	338	12	269	56	295
2007	320	8	258	55	308
2008	303	5	245	53	321
2009	286	3	232	51	334
2010	271	2	220	49	346
2011	255	1	208	46	357
2012	241	1	196	44	368
2013	227	0	184	42	379
2014	213	0	173	40	389
2015	201	0	163	38	399
2016	189	0	153	35	408

Year	HIV/AIDS[2]	HIV CD4 >500	HIV CD4 <500	AIDS	Total deaths[3]
2017	177	0	144	33	417
2018	166	0	135	31	425
2019	156	0	126	29	432
2020	146	0	118	28	439
2021	137	0	111	26	446
2022	128	0	104	24	452
2023	120	0	97	23	458
2024	112	0	90	21	464
2025	104	0	84	20	469
2026	97	0	78	19	474
2027	90	0	73	17	479
2028	84	0	67	16	483
2029	77	0	62	15	487
2030	72	0	58	14	490
2031	66	0	53	13	494
2032	61	0	49	12	497
2033	56	0	45	11	500
2034	51	0	41	10	503
2035	47	0	38	9	505
2036	43	0	34	9	507
2037	39	0	31	8	510
2038	35	0	28	7	511
2039	32	0	25	7	513
2040	28	0	22	6	515
2041	25	0	20	5	516
2042	23	0	18	5	518
2043	20	0	16	4	519
2044	18	0	14	4	520
2045	15	0	12	3	521
2046	13	0	10	3	522
2047	11	0	9	3	523
2048	10	0	7	2	523
2049	8	0	6	2	524
2050	7	0	5	2	524
2051	6	0	4	1	525
2052	5	0	4	1	525
2053	4	0	3	1	526

Year	HIV/AIDS[2]	HIV CD4 >500	HIV CD4 <500	AIDS	Total deaths[3]
2054	3	0	2	1	526
2055	3	0	2	1	526
2056	2	0	1	1	526
2057	2	0	1	0	526
2058	1	0	1	0	527
2059	1	0	1	0	527
2060	1	0	0	0	527
2061	0	0	0	0	527
2062	0	0	0	0	527
2063	0	0	0	0	527
2064	0	0	0	0	527
2065	0	0	0	0	527
2066	0	0	0	0	527
2067	0	0	0	0	527
2068	0	0	0	0	527
2069	0	0	0	0	527
2070	0	0	0	0	527

1. NSP introduction in 1988
2. HIV/AIDS=number of people living with either HIV (CD4>500+CD4<500) or AIDS
3. Total deaths=cumulative deaths

Table 3.4.2 Estimated number of people living with HIV/AIDS and deaths without NSP introduction

Year	HIV/AIDS1	HIV CD4 >500	HIV CD4 <500	AIDS	Total deaths2
1981	2	2	0	0	0
1982	7	7	0	0	0
1983	23	22	1	0	0
1984	60	57	2	1	0
1985	121	112	8	1	0
1986	201	180	18	3	1
1987	284	241	36	7	3
1988	414	339	63	12	7
1989	592	471	101	19	13
1990	845	663	153	30	22
1991	1208	940	223	44	36
1992	1724	1339	321	65	58
1993	2459	1906	458	94	89
1994	3498	2709	654	135	134
1995	4959	3835	932	192	199
1996	7026	5398	1371	256	269
1997	9894	7542	2010	343	354
1998	13825	10434	2933	458	456
1999	19123	14262	4255	606	575
2000	26126	19206	6130	790	711
2001	25619	15935	8675	1009	885
2002	25063	12353	11430	1280	1104
2003	24450	8971	13897	1582	1376
2004	23774	6150	15732	1893	1706
2005	23040	4056	16796	2188	2101
2006	22245	2644	17153	2448	2561
2007	21397	1712	17028	2657	3080
2008	20513	1101	16615	2798	3650
2009	19602	703	16021	2878	4258
2010	18679	447	15335	2898	4894
2011	17757	282	14605	2870	5546
2012	16841	177	13858	2806	6202
2013	15942	110	13116	2716	6853
2014	15068	69	12391	2609	7492
2015	14224	43	11691	2490	8111
2016	13413	26	11021	2366	8707

Year	HIV/AIDS1	HIV CD4 >500	HIV CD4 <500	AIDS	Total deaths2
2017	12639	16	10382	2241	9277
2018	11903	10	9776	2118	9818
2019	11202	6	9198	1998	10331
2020	10538	4	8651	1883	10816
2021	9907	2	8132	1773	11274
2022	9310	1	7641	1668	11705
2023	8745	1	7176	1568	12111
2024	8209	0	6735	1474	12493
2025	7702	0	6317	1385	12853
2026	7220	0	5920	1300	13190
2027	6764	0	5544	1219	13507
2028	6330	0	5187	1143	13805
2029	5919	0	4848	1071	14084
2030	5528	0	4525	1002	14346
2031	5157	0	4219	937	14591
2032	4805	0	3929	876	14821
2033	4471	0	3654	817	15036
2034	4153	0	3392	761	15236
2035	3852	0	3144	709	15423
2036	3566	0	2908	658	15597
2037	3294	0	2684	611	15759
2038	3037	0	2471	566	15910
2039	2793	0	2270	523	16050
2040	2561	0	2079	482	16179
2041	2342	0	1898	444	16298
2042	2134	0	1727	407	16409
2043	1938	0	1566	372	16510
2044	1753	0	1414	339	16603
2045	1578	0	1270	308	16688
2046	1414	0	1135	279	16766
2047	1261	0	1010	252	16836
2048	1118	0	892	226	16900
2049	985	0	783	202	16957
2050	861	0	682	179	17009
2051	747	0	589	158	17055
2052	643	0	504	138	17096
2053	548	0	427	120	17132

Year	HIV/AIDS1	HIV CD4 >500	HIV CD4 <500	AIDS	Total deaths2
2054	462	0	358	104	17164
2055	386	0	297	89	17192
2056	318	0	243	75	17216
2057	259	0	196	63	17236
2058	208	0	156	52	17254
2059	164	0	122	43	17268
2060	128	0	94	34	17281
2061	98	0	71	27	17291
2062	74	0	53	21	17299
2063	55	0	39	16	17306
2064	40	0	28	12	17311
2065	28	0	20	8	17315
2066	19	0	13	6	17319
2067	12	0	9	4	17321
2068	7	0	5	2	17323
2069	3	0	2	1	17324
2070	0	0	0	0	17325

1. HIV/AIDS=number of people living with either HIV (CD4>500+CD4<500) or AIDS
2. Total deaths=cumulative deaths

Table 3.4.3 Estimated number of HIV/AIDS cases and deaths prevented through NSP introduction[1]

Year	HIV/AIDS[2]	HIV CD4>500	HIV CD4<500	AIDS	Total deaths[3]
1981	0	0	0	0	0
1982	0	0	0	0	0
1983	0	0	0	0	0
1984	0	0	0	0	0
1985	0	0	0	0	0
1986	0	0	0	0	0
1987	0	0	0	0	0
1988	59	59	0	0	0
1989	183	178	4	1	0
1990	401	379	18	4	1
1991	742	680	52	10	3
1992	1250	1109	118	23	9
1993	1988	1711	233	44	21
1994	3033	2541	414	78	44
1995	4503	3688	685	131	83
1996	6573	5266	1115	192	132
1997	9445	7419	1748	278	196
1998	13376	10318	2666	393	280
1999	18674	14150	3983	540	382
2000	25673	19096	5851	725	503
2001	25186	15851	8390	946	662
2002	24650	12291	11141	1219	866
2003	24056	8927	13608	1521	1124
2004	23400	6120	15446	1834	1439
2005	22684	4037	16517	2130	1820
2006	21908	2631	16884	2392	2266
2007	21077	1704	16771	2602	2772
2008	20211	1095	16370	2746	3329
2009	19316	700	15788	2828	3925
2010	18409	445	15115	2849	4549
2011	17502	281	14397	2824	5189
2012	16600	176	13662	2762	5834
2013	15715	110	12932	2674	6474
2014	14854	68	12217	2569	7103
2015	14023	43	11528	2453	7712
2016	13225	26	10868	2331	8299

Year	HIV/AIDS[2]	HIV CD4>500	HIV CD4<500	AIDS	Total deaths[3]
2017	12462	16	10238	2208	8860
2018	11737	10	9641	2086	9394
2019	11046	6	9072	1968	9899
2020	10391	4	8532	1855	10377
2021	9770	2	8021	1747	10828
2022	9182	1	7537	1644	11253
2023	8625	1	7079	1546	11653
2024	8097	0	6645	1453	12029
2025	7597	0	6233	1365	12383
2026	7123	0	5842	1281	12716
2027	6674	0	5472	1202	13029
2028	6247	0	5120	1127	13322
2029	5841	0	4785	1056	13598
2030	5456	0	4468	988	13856
2031	5091	0	4166	924	14098
2032	4744	0	3880	864	14324
2033	4415	0	3609	806	14536
2034	4102	0	3351	751	14733
2035	3805	0	3106	699	14918
2036	3524	0	2874	650	15090
2037	3256	0	2653	603	15250
2038	3001	0	2443	558	15398
2039	2761	0	2245	516	15536
2040	2533	0	2057	476	15664
2041	2316	0	1878	438	15782
2042	2112	0	1710	402	15891
2043	1918	0	1550	368	15991
2044	1735	0	1400	336	16083
2045	1563	0	1258	305	16167
2046	1401	0	1125	276	16244
2047	1250	0	1001	249	16314
2048	1108	0	885	224	16377
2049	976	0	777	200	16434
2050	854	0	677	178	16485
2051	741	0	585	157	16530
2052	638	0	501	137	16571
2053	544	0	424	119	16607

Year	HIV/AIDS[2]	HIV CD4>500	HIV CD4<500	AIDS	Total deaths[3]
2054	459	0	356	103	16638
2055	383	0	295	88	16666
2056	316	0	241	75	16690
2057	257	0	195	63	16710
2058	207	0	155	52	16727
2059	163	0	121	42	16742
2060	127	0	93	34	16754
2061	97	0	70	27	16764
2062	73	0	52	21	16772
2063	55	0	39	16	16779
2064	40	0	28	12	16784
2065	28	0	20	8	16788
2066	19	0	13	6	16792
2067	13	0	9	4	16794
2068	8	0	6	2	16796
2069	3	0	2	1	16798
2070	0	0	0	0	16799

1. NSP introduction in 1988
2. HIV/AIDS=number of people living with either HIV (CD4>500+CD4<500) or AIDS
3. Total deaths=cumulative deaths

Table 3.4.5 Estimated number of HIV/AIDS cases prevented through NSP introduction[1] by disease stage and diagnosis category[2]

Year	HIV/AIDS[3]	CD4 >500 (diagnosed)	CD4 >500 (undiagnosed)	CD4 <500 (diagnosed)	CD4 <500 (undiagnosed)	AIDS
1981	0	0	0	0	0	0
1982	0	0	0	0	0	0
1983	0	0	0	0	0	0
1984	0	0	0	0	0	0
1985	0	0	0	0	0	0
1986	0	0	0	0	0	0
1987	0	0	0	0	0	0
1988	59	47	12	0	0	0
1989	183	142	36	4	0	1
1990	401	303	76	16	2	4
1991	742	544	136	47	5	10
1992	1250	887	222	107	12	23
1993	1988	1369	342	210	23	44
1994	3033	2033	508	373	41	78
1995	4503	2950	738	616	68	131
1996	6573	4213	1053	1004	112	192
1997	9445	5935	1484	1573	175	278
1998	13376	8255	2064	2399	267	393
1999	18674	11320	2830	3585	398	540
2000	25673	15277	3819	5266	585	725
2001	25186	12681	3170	7551	839	946
2002	24650	9833	2458	10027	1114	1219
2003	24056	7142	1785	12247	1361	1521
2004	23400	4896	1224	13901	1545	1834
2005	22684	3230	807	14865	1652	2130
2006	21908	2105	526	15196	1688	2392
2007	21077	1363	341	15094	1677	2602
2008	20211	876	219	14733	1637	2746
2009	19316	560	140	14210	1579	2828
2010	18409	356	89	13603	1511	2849
2011	17502	225	56	12958	1440	2824
2012	16600	141	35	12296	1366	2762
2013	15715	88	22	11638	1293	2674
2014	14854	55	14	10996	1222	2569
2015	14023	34	9	10375	1153	2453

Year	HIV/AIDS[3]	CD4 >500 (diagnosed)	CD4 >500 (undiagnosed)	CD4 <500 (diagnosed)	CD4 <500 (undiagnosed)	AIDS
2016	13225	21	5	9781	1087	2331
2017	12462	13	3	9214	1024	2208
2018	11737	8	2	8677	964	2086
2019	11046	5	1	8165	907	1968
2020	10391	3	1	7679	853	1855
2021	9770	2	0	7219	802	1747
2022	9182	1	0	6784	754	1644
2023	8625	1	0	6371	708	1546
2024	8097	0	0	5980	664	1453
2025	7597	0	0	5610	623	1365
2026	7123	0	0	5258	584	1281
2027	6674	0	0	4925	547	1202
2028	6247	0	0	4608	512	1127
2029	5841	0	0	4307	479	1056
2030	5456	0	0	4021	447	988
2031	5091	0	0	3750	417	924
2032	4744	0	0	3492	388	864
2033	4415	0	0	3248	361	806
2034	4102	0	0	3016	335	751
2035	3805	0	0	2796	311	699
2036	3524	0	0	2587	287	650
2037	3256	0	0	2388	265	603
2038	3001	0	0	2199	244	558
2039	2761	0	0	2021	225	516
2040	2533	0	0	1851	206	476
2041	2316	0	0	1690	188	438
2042	2112	0	0	1539	171	402
2043	1918	0	0	1395	155	368
2044	1735	0	0	1260	140	336
2045	1563	0	0	1132	126	305
2046	1401	0	0	1013	113	276
2047	1250	0	0	901	100	249
2048	1108	0	0	796	88	224
2049	976	0	0	699	78	200
2050	854	0	0	609	68	178
2051	741	0	0	526	58	157
2052	638	0	0	451	50	137

Year	HIV/AIDS[3]	CD4 >500 (diagnosed)	CD4 >500 (undiagnosed)	CD4 <500 (diagnosed)	CD4 <500 (undiagnosed)	AIDS
2053	544	0	0	382	42	119
2054	459	0	0	320	36	103
2055	383	0	0	265	29	88
2056	316	0	0	217	24	75
2057	257	0	0	175	19	63
2058	207	0	0	139	15	52
2059	163	0	0	109	12	42
2060	127	0	0	84	9	34
2061	98	0	0	63	7	27
2062	74	0	0	47	5	21
2063	54	0	0	35	4	16
2064	40	0	0	25	3	12
2065	28	0	0	18	2	8
2066	19	0	0	12	1	6
2067	12	0	0	8	1	4
2068	7	0	0	5	1	2
2069	3	0	0	2	0	1
2070	0	0	0	0	0	0

1. NSP introduction in 1988
2. Proportions diagnosed are 80% for CD4>500, 90% for CD4<500, and 100% for AIDS.
3. HIV/AIDS=number of people living with either HIV (CD4>500+CD4<500) or AIDS

Table 3.5.1 Estimates of people living with HCV by disease stage and HCV-related deaths with NSPs

Year	Total living with HCV	Total chronic	Total 0/1	Total 2/3	Total cirrhosis	HCC incidence	Liver failure incidence	Total deaths	HCV deaths	Other deaths
1961	917	688	674	14	0	0	0	0	0	0
1962	2123	1592	1547	45	0	0	0	1	1	0
1963	3578	2683	2586	96	1	0	0	2	2	1
1964	5251	3938	3765	169	3	0	0	4	3	1
1965	7117	5338	5064	266	7	0	0	6	5	2
1966	9157	6868	6466	389	13	0	1	9	7	2
1967	11355	8516	7958	537	22	0	1	13	10	3
1968	13697	10273	9528	711	34	0	1	18	13	4
1969	16174	12131	11168	913	49	0	2	23	17	6
1970	18778	14083	12872	1142	69	1	3	30	22	7
1971	21434	16076	14584	1398	94	1	3	37	28	9
1972	24166	18125	16321	1679	124	1	4	45	34	11
1973	26997	20248	18100	1987	161	2	6	55	41	14
1974	29949	22462	19938	2321	203	2	7	65	49	16
1975	35723	26792	23818	2721	253	2	9	78	59	20
1976	38952	29214	25757	3148	310	3	11	93	70	23
1977	42373	31780	27803	3602	375	4	13	111	83	28
1978	46021	34516	29980	4087	448	4	16	130	98	33
1979	49918	37439	32302	4605	532	5	19	152	114	38
1980	54085	40564	34781	5157	626	6	22	175	131	44
1981	58545	43909	37432	5747	730	7	25	201	151	50
1982	63324	47493	40269	6377	847	8	29	229	172	57
1983	68445	51334	43307	7050	977	10	34	260	195	65

Year	Total living with HCV	Total chronic	Total 0/1	Total 2/3	Total cirrhosis	HCC incidence	Liver failure incidence	Total deaths	HCV deaths	Other deaths
1984	73882	55412	46524	7768	1120	11	39	293	220	73
1985	79301	59476	49670	8529	1277	13	44	328	246	82
1986	84716	63537	52757	9330	1451	14	50	366	275	92
1987	90126	67594	55787	10167	1640	16	57	406	305	102
1988	95579	71684	58796	11041	1846	18	64	449	337	112
1989	101056	75792	61773	11949	2070	20	71	495	371	124
1990	106596	79947	64746	12889	2313	23	79	543	407	136
1991	112730	84548	68101	13873	2573	25	88	594	445	148
1992	119459	89594	71837	14904	2854	28	98	648	486	162
1993	126815	95111	75970	15986	3154	31	108	706	530	177
1994	134836	101127	80522	17129	3476	34	119	769	576	192
1995	143566	107674	85517	18337	3820	38	130	835	626	209
1996	153049	114787	90979	19620	4188	41	143	907	680	227
1997	163338	122504	96940	20983	4580	45	156	983	738	246
1998	174493	130870	103435	22436	4999	49	170	1066	799	266
1999	186573	139930	110498	23986	5445	54	185	1155	866	289
2000	199649	149737	118172	25644	5922	58	201	1251	938	313
2001	197976	148482	114874	27179	6429	63	218	1349	1012	337
2002	196338	147254	111691	28599	6964	68	236	1450	1088	363
2003	194737	146053	108619	29909	7524	74	255	1555	1166	389
2004	193162	144871	105651	31115	8105	80	274	1665	1249	416
2005	191611	143708	102782	32222	8704	86	294	1779	1334	445
2006	190084	142563	100010	33235	9318	92	315	1900	1425	475
2007	188586	141440	97335	34160	9944	98	335	2027	1520	507

Year	Total living with HCV	Total chronic	Total 0/1	Total 2/3	Total cirrhosis	HCC incidence	Liver failure incidence	Total deaths	HCV deaths	Other deaths
2008	187112	140334	94753	35000	10580	104	356	2162	1622	541
2009	185657	139243	92258	35761	11223	110	377	2305	1729	576
2010	184234	138175	89857	36448	11870	117	399	2457	1843	614
2011	182834	137126	87542	37064	12520	123	420	2618	1964	655
2012	181453	136090	85309	37612	13169	129	441	2790	2093	698
2013	180104	135078	83164	38099	13815	136	462	2974	2231	744
2014	178778	134083	81101	38526	14456	142	483	3170	2378	793
2015	177480	133110	79121	38899	15090	148	504	3379	2534	845
2016	176216	132162	77225	39221	15715	154	524	3602	2702	901
2017	174841	131131	75336	39470	16324	160	544	3845	2884	961
2018	173353	130015	73454	39646	16914	166	563	4109	3082	1027
2019	171753	128815	71580	39753	17482	172	581	4396	3297	1099
2020	170028	127521	69710	39788	18023	177	598	4707	3530	1177
2021	168171	126128	67841	39751	18536	182	615	5045	3783	1261
2022	166182	124637	65974	39645	19017	187	630	5409	4057	1352
2023	164057	123043	64109	39470	19463	191	644	5803	4352	1451
2024	161786	121339	62243	39224	19872	195	657	6228	4655	1572
2025	159370	119528	60375	38911	20241	199	668	6683	4963	1720
2026	156804	117603	58506	38529	20568	202	678	7171	5276	1895
2027	154094	115571	56638	38083	20850	205	687	7692	5594	2098
2028	151236	113427	54768	37572	21087	207	694	8246	5915	2331
2029	148229	111172	52897	36998	21276	209	699	8834	6239	2595
2030	145079	108809	51028	36364	21417	210	703	9455	6565	2889
2031	141783	106337	49159	35670	21508	211	705	10109	6893	3217

Year	Total living with HCV	Total chronic	Total 0/1	Total 2/3	Total cirrhosis	HCC incidence	Liver failure incidence	Total deaths	HCV deaths	Other deaths
2032	138349	103762	47293	34921	21548	211	706	10797	7221	3576
2033	134776	101082	45429	34116	21537	211	704	11518	7549	3969
2034	131069	98302	43569	33259	21474	211	701	12271	7876	4395
2035	127234	95426	41714	32353	21358	209	697	13057	8201	4856
2036	123274	92455	39868	31400	21187	208	690	13876	8524	5353
2037	119200	89400	38031	30405	20964	205	682	14727	8843	5884
2038	115016	86262	36206	29370	20687	203	672	15608	9158	6449
2039	110735	83051	34395	28299	20358	199	661	16517	9468	7049
2040	106361	79771	32600	27195	19976	196	647	17456	9772	7683
2041	101902	76426	30822	26062	19542	191	632	18422	10070	8352
2042	97365	73024	29064	24903	19056	187	615	19415	10360	9055
2043	92765	69574	27330	23723	18521	181	597	20432	10642	9790
2044	88124	66093	25625	22528	17939	176	577	21471	10915	10555
2045	83434	62576	23947	21319	17310	170	556	22531	11179	11352
2046	78714	59036	22299	20099	16637	163	533	23610	11432	12177
2047	73984	55488	20688	18876	15924	156	509	24703	11675	13028
2048	69251	51938	19114	17650	15173	149	484	25808	11906	13902
2049	64538	48404	17583	16431	14389	141	458	26921	12125	14796
2050	59827	44871	16091	15204	13575	133	430	28039	12332	15707
2051	55203	41402	14655	14010	12737	125	403	29156	12526	16631
2052	50653	37990	13273	12836	11881	116	374	30268	12707	17561
2053	46191	34643	11947	11685	11011	108	346	31369	12874	18495
2054	41835	31376	10679	10564	10133	99	317	32456	13029	19427
2055	37609	28207	9475	9477	9254	90	288	33522	13170	20353

Year	Total living with HCV	Total chronic	Total 0/1	Total 2/3	Total cirrhosis	HCC incidence	Liver failure incidence	Total deaths	HCV deaths	Other deaths
2056	33539	25154	8339	8433	8382	82	259	34561	13297	21264
2057	29649	22237	7276	7438	7524	74	231	35565	13412	22153
2058	25957	19468	6286	6495	6687	65	204	36529	13514	23015
2059	22487	16865	5374	5612	5880	57	178	37446	13603	23843
2060	19265	14449	4543	4794	5112	50	154	38307	13681	24626
2061	16307	12230	3794	4046	4390	43	131	39107	13748	25360
2062	13621	10215	3127	3369	3720	36	110	39843	13804	26039
2063	11216	8412	2540	2765	3107	30	91	40509	13852	26658
2064	9101	6826	2034	2236	2556	25	74	41103	13891	27212
2065	7264	5448	1601	1778	2068	20	59	41625	13922	27703
2066	5699	4274	1239	1390	1645	16	46	42075	13947	28128
2067	4393	3295	943	1068	1284	12	35	42456	13967	28489
2068	3316	2487	702	803	982	10	26	42774	13982	28792
2069	2451	1838	513	591	734	7	19	43033	13993	29040
2070	1759	1319	363	423	533	5	13	43242	14001	29241
2071	1205	904	246	289	369	4	9	43411	14007	29405
2072	776	582	157	185	240	2	5	43544	14010	29533
2073	447	335	89	106	140	1	3	43646	14012	29634
2074	197	147	39	47	62	1	0	43725	14013	29712
2075	0	0	0	0	0	0	0	43787	14013	29773

Note: Deaths are cumulative following cirrhosis

Table 3 5.2 Estimates of people living with HCV by disease stage and HCV-related deaths without NSP introduction

Year	Total living with HCV	Total chronic	Total 0/1	Total 2/3	Total cirrhosis	HCC incidence	Liver failure incidence	Total deaths	HCV deaths	Other deaths
1961	917	688	674	14	0	0	0	0	0	0
1962	2123	1592	1547	45	0	0	0	1	1	0
1963	3578	2683	2586	96	1	0	0	2	2	1
1964	5251	3938	3765	169	3	0	0	4	3	1
1965	7117	5338	5064	266	7	0	0	6	5	2
1966	9157	6868	6466	389	13	0	1	9	7	2
1967	11355	8516	7958	537	22	0	1	13	10	3
1968	13697	10273	9528	711	34	0	1	18	13	4
1969	16174	12131	11168	913	49	0	2	23	17	6
1970	18778	14083	12872	1142	69	1	3	30	22	7
1971	21434	16076	14584	1398	94	1	3	37	28	9
1972	24166	18125	16321	1679	124	1	4	45	34	11
1973	26997	20248	18100	1987	161	2	6	55	41	14
1974	29949	22462	19938	2321	203	2	7	65	49	16
1975	35723	26792	23818	2721	253	2	9	78	59	20
1976	38952	29214	25757	3148	310	3	11	93	70	23
1977	42373	31780	27803	3602	375	4	13	111	83	28
1978	46021	34516	29980	4087	448	4	16	130	98	33
1979	49918	37439	32302	4605	532	5	19	152	114	38
1980	54085	40564	34781	5157	626	6	22	175	131	44
1981	58545	43909	37432	5747	730	7	25	201	151	50
1982	63324	47493	40269	6377	847	8	29	229	172	57
1983	68445	51334	43307	7050	977	10	34	260	195	65

Year	Total living with HCV	Total chronic	Total 0/1	Total 2/3	Total cirrhosis	HCC incidence	Liver failure incidence	Total deaths	HCV deaths	Other deaths
1984	73882	55412	46524	7768	1120	11	39	293	220	73
1985	79301	59476	49670	8529	1277	13	44	328	246	82
1986	84716	63537	52757	9330	1451	14	50	366	275	92
1987	90126	67594	55787	10167	1640	16	57	406	305	102
1988	96588	72441	59538	11056	1846	18	64	450	337	112
1989	103463	77597	63527	11999	2071	20	71	496	372	124
1990	110395	82796	67489	12993	2314	23	79	545	409	136
1991	117933	88449	71822	14050	2577	25	88	598	448	150
1992	126094	94571	76539	15170	2862	28	98	655	491	164
1993	134927	101196	81668	16359	3168	31	108	716	536	179
1994	144484	108363	87240	17625	3498	34	119	781	585	196
1995	154819	116115	93288	18974	3853	38	131	851	638	213
1996	165994	124496	99847	20414	4235	42	144	927	695	232
1997	178073	133555	106958	21952	4644	46	158	1009	756	253
1998	191127	143345	114663	23598	5084	50	173	1097	822	275
1999	205232	153924	123007	25361	5556	55	189	1193	894	299
2000	220470	165352	132040	27251	6061	60	206	1296	972	324
2001	218567	163926	128321	29001	6603	65	224	1402	1051	351
2002	216703	162527	124730	30620	7177	71	244	1510	1132	378
2003	214876	161157	121263	32114	7779	76	264	1623	1217	406
2004	213086	159814	117917	33491	8406	83	285	1740	1305	435
2005	211333	158499	114687	34757	9055	89	306	1863	1396	466
2006	209616	157212	111571	35918	9723	96	329	1991	1493	498
2007	207944	155958	108571	36981	10405	102	351	2125	1594	532

Year	Total living with HCV	Total chronic	Total 0/1	Total 2/3	Total cirrhosis	HCC incidence	Liver failure incidence	Total deaths	HCV deaths	Other deaths
2008	206308	154731	105680	37951	11100	109	374	2268	1700	567
2009	204704	153528	102890	38833	11805	116	397	2418	1813	605
2010	203147	152360	100211	39633	12517	123	421	2577	1932	645
2011	201628	151221	97633	40356	13232	130	444	2745	2058	687
2012	200141	150106	95151	41005	13950	137	468	2924	2193	731
2013	198702	149026	92773	41586	14667	144	491	3115	2335	779
2014	197301	147975	90490	42104	15381	151	515	3317	2487	830
2015	195945	146958	88305	42563	16090	158	538	3532	2649	883
2016	194640	145980	86220	42968	16793	165	561	3761	2821	941
2017	193220	144915	84143	43292	17480	172	583	4010	3007	1003
2018	191683	143762	82076	43538	18148	178	604	4281	3210	1071
2019	190028	142521	80019	43708	18795	184	625	4575	3431	1144
2020	188244	141183	77968	43799	19416	191	645	4894	3670	1224
2021	186323	139742	75920	43813	20009	196	664	5239	3929	1310
2022	184263	138197	73876	43751	20570	202	682	5613	4210	1404
2023	182059	136544	71835	43613	21096	207	699	6017	4513	1505
2024	179700	134775	69793	43399	21584	212	714	6453	4839	1614
2025	177189	132892	67751	43110	22031	216	728	6921	5175	1746
2026	174517	130888	65707	42746	22434	220	740	7423	5516	1907
2027	171690	128768	63665	42312	22791	224	751	7960	5864	2096
2028	168704	126528	61621	41807	23100	227	761	8531	6215	2316
2029	165556	124167	59576	41231	23360	229	768	9139	6571	2568
2030	162251	121688	57532	40589	23568	231	774	9782	6930	2852
2031	158785	119089	55487	39879	23723	233	779	10462	7291	3171

Year	Total living with HCV	Total chronic	Total 0/1	Total 2/3	Total cirrhosis	HCC incidence	Liver failure incidence	Total deaths	HCV deaths	Other deaths
2032	155164	116373	53443	39107	23824	234	781	11178	7654	3524
2033	151387	113540	51399	38272	23869	234	782	11930	8017	3913
2034	147456	110592	49358	37378	23856	234	780	12719	8381	4338
2035	143378	107534	47320	36426	23787	233	777	13544	8743	4801
2036	139153	104365	45288	35420	23656	232	772	14407	9103	5304
2037	134792	101094	43264	34364	23466	230	765	15306	9461	5846
2038	130298	97724	41249	33259	23216	228	755	16241	9814	6427
2039	125681	94261	39245	32110	22905	224	744	17211	10163	7048
2040	120947	90710	37255	30920	22534	221	731	18215	10506	7709
2041	116099	87074	35280	29692	22102	217	716	19254	10843	8411
2042	111147	83360	33321	28430	21609	212	699	20327	11172	9155
2043	106103	79578	31383	27138	21057	206	680	21431	11492	9938
2044	100991	75743	29472	25823	20448	200	659	22564	11804	10760
2045	95802	71851	27585	24486	19780	194	636	23727	12105	11622
2046	90554	67915	25728	23129	19058	187	612	24916	12395	12521
2047	85269	63952	23905	21761	18285	179	585	26127	12674	13454
2048	79955	59966	22119	20385	17463	171	558	27359	12940	14420
2049	74640	55980	20375	19008	16596	162	529	28606	13192	15414
2050	69306	51979	18671	17619	15689	154	498	29866	13431	16434
2051	64041	48031	17024	16258	14748	144	467	31131	13656	17475
2052	58840	44130	15435	14914	13780	135	435	32396	13866	18530
2053	53720	40290	13906	13593	12791	125	402	33655	14061	19594
2054	48703	36527	12441	12300	11786	115	369	34903	14240	20663
2055	43820	32865	11046	11044	10776	105	335	36131	14404	21727

Year	Total living with HCV	Total chronic	Total 0/1	Total 2/3	Total cirrhosis	HCC incidence	Liver failure incidence	Total deaths	HCV deaths	Other deaths
2056	39106	29329	9727	9834	9769	95	302	37332	14553	22779
2057	34590	25943	8490	8678	8775	86	270	38495	14687	23809
2058	30298	22724	7337	7582	7805	76	239	39615	14805	24810
2059	26259	19694	6275	6553	6866	67	208	40681	14910	25771
2060	22505	16879	5306	5600	5972	58	180	41684	15001	26683
2061	19056	14292	4433	4728	5131	50	153	42617	15079	27538
2062	15922	11942	3654	3938	4350	42	128	43475	15145	28329
2063	13107	9831	2968	3231	3632	35	106	44255	15201	29055
2064	10628	7971	2374	2611	2986	29	86	44951	15246	29705
2065	8478	6358	1868	2075	2415	24	68	45563	15283	30280
2066	6647	4986	1445	1621	1919	19	53	46090	15312	30778
2067	5121	3841	1099	1244	1497	15	41	46535	15335	31200
2068	3864	2898	818	935	1144	11	31	46906	15352	31554
2069	2854	2140	597	688	855	8	22	47208	15365	31843
2070	2048	1536	423	492	621	6	16	47452	15375	32077
2071	1402	1051	286	336	430	4	10	47649	15381	32268
2072	903	677	182	215	279	3	6	47804	15386	32418
2073	519	389	104	123	162	2	3	47923	15388	32535
2074	228	171	45	54	72	1	0	48014	15389	32625
2075	0	0	0	0	0	0	0	48086	15389	32697

Note: Deaths are cumulative following cirrhosis

Table 3.5.3 Number of HCV infections and HCV-related deaths prevented through introduction of NSP

Year	Living with HCV	Chronic	Stage 0/1	Stage 2/3	Cirrhosis	HCC incidence	Liver failure incidence	Total deaths	HCV deaths
1961	0	0	0	0	0	0	0	0	0
1962	0	0	0	0	0	0	0	0	0
1963	0	0	0	0	0	0	0	0	0
1964	0	0	0	0	0	0	0	0	0
1965	0	0	0	0	0	0	0	0	0
1966	0	0	0	0	0	0	0	0	0
1967	0	0	0	0	0	0	0	0	0
1968	0	0	0	0	0	0	0	0	0
1969	0	0	0	0	0	0	0	0	0
1970	0	0	0	0	0	0	0	0	0
1971	0	0	0	0	0	0	0	0	0
1972	0	0	0	0	0	0	0	0	0
1973	0	0	0	0	0	0	0	0	0
1974	0	0	0	0	0	0	0	0	0
1975	0	0	0	0	0	0	0	0	0
1976	0	0	0	0	0	0	0	0	0
1977	0	0	0	0	0	0	0	0	0
1978	0	0	0	0	0	0	0	0	0
1979	0	0	0	0	0	0	0	0	0
1980	0	0	0	0	0	0	0	0	0
1981	0	0	0	0	0	0	0	0	0
1982	0	0	0	0	0	0	0	0	0
1983	0	0	0	0	0	0	0	0	0
1984	0	0	0	0	0	0	0	0	0
1985	0	0	0	0	0	0	0	0	0
1986	0	0	0	0	0	0	0	0	0
1987	0	0	0	0	0	0	0	0	0
1988	1009	757	742	15	0	0	0	0	0
1989	2407	1805	1754	50	0	0	0	1	1
1990	3798	2849	2743	105	1	0	0	2	2
1991	5202	3902	3721	177	4	0	0	4	3
1992	6635	4977	4703	266	8	0	0	6	4
1993	8112	6084	5698	373	14	0	1	9	6
1994	9647	7235	6718	496	22	0	1	12	9
1995	11254	8440	7771	636	33	0	1	16	12

Year	Living with HCV	Chronic	Stage 0/1	Stage 2/3	Cirrhosis	HCC incidence	Liver failure incidence	Total deaths	HCV deaths
1996	12945	9709	8868	794	47	0	2	20	15
1997	14734	11051	10018	969	64	1	2	26	19
1998	16634	12476	11228	1162	85	1	3	31	23
1999	18659	13994	12509	1375	110	1	4	38	28
2000	20820	15615	13868	1607	140	1	5	45	33
2001	20592	15444	13447	1823	174	2	6	53	39
2002	20364	15273	13039	2022	213	2	8	60	45
2003	20139	15104	12644	2205	255	3	9	68	50
2004	19924	14943	12266	2376	301	3	11	76	56
2005	19721	14791	11905	2535	351	3	12	83	62
2006	19532	14649	11562	2683	405	4	14	91	68
2007	19358	14518	11236	2822	461	5	16	98	73
2008	19196	14397	10926	2951	520	5	18	106	79
2009	19047	14285	10632	3072	582	6	20	113	84
2010	18913	14185	10354	3185	646	6	22	120	90
2011	18794	14095	10091	3292	713	7	24	127	95
2012	18688	14016	9842	3392	781	8	27	134	100
2013	18598	13948	9609	3487	852	8	29	140	105
2014	18523	13892	9389	3578	925	9	31	147	110
2015	18465	13848	9185	3664	1000	10	34	153	114
2016	18424	13818	8994	3746	1077	11	36	159	119
2017	18379	13784	8807	3822	1155	11	39	165	123
2018	18329	13747	8622	3892	1234	12	42	172	128
2019	18276	13707	8439	3955	1313	13	44	179	134
2020	18217	13662	8258	4011	1393	14	47	186	139
2021	18152	13614	8079	4061	1473	14	49	195	146
2022	18080	13560	7902	4105	1553	15	52	204	153
2023	18002	13501	7726	4143	1633	16	55	214	160
2024	17915	13436	7550	4174	1712	17	57	225	184
2025	17819	13364	7376	4199	1789	18	60	238	212
2026	17712	13284	7201	4217	1866	18	62	252	240
2027	17596	13197	7027	4229	1941	19	65	268	270
2028	17468	13101	6853	4234	2013	20	67	286	300
2029	17327	12995	6679	4233	2084	20	69	305	332
2030	17172	12879	6504	4225	2151	21	71	328	365
2031	17002	12751	6327	4209	2215	22	74	353	398
2032	16815	12611	6150	4186	2275	22	75	381	433

Year	Living with HCV	Chronic	Stage 0/1	Stage 2/3	Cirrhosis	HCC incidence	Liver failure incidence	Total deaths	HCV deaths
2033	16611	12458	5970	4156	2331	23	77	412	469
2034	16387	12291	5789	4119	2383	23	79	447	505
2035	16144	12108	5606	4073	2428	24	80	487	542
2036	15879	11909	5421	4020	2468	24	81	531	579
2037	15592	11694	5233	3959	2502	25	82	579	618
2038	15282	11461	5044	3889	2529	25	83	634	656
2039	14946	11210	4851	3811	2548	25	84	693	695
2040	14585	10939	4656	3725	2558	25	84	759	734
2041	14197	10648	4457	3630	2560	25	84	832	773
2042	13782	10336	4256	3527	2553	25	83	912	812
2043	13338	10004	4053	3415	2536	25	83	999	850
2044	12867	9651	3847	3295	2509	25	82	1093	888
2045	12367	9275	3639	3166	2470	24	80	1196	926
2046	11839	8880	3429	3030	2421	24	78	1306	963
2047	11285	8464	3217	2886	2361	23	76	1425	999
2048	10705	8028	3004	2735	2289	22	74	1551	1034
2049	10102	7576	2792	2578	2207	22	71	1685	1067
2050	9478	7109	2580	2415	2114	21	68	1827	1100
2051	8838	6629	2369	2248	2011	20	64	1975	1130
2052	8187	6140	2162	2079	1899	19	60	2128	1159
2053	7529	5646	1959	1908	1779	17	56	2286	1186
2054	6868	5151	1761	1736	1653	16	52	2447	1211
2055	6211	4659	1570	1567	1521	15	48	2609	1235
2056	5567	4175	1387	1401	1387	14	43	2771	1256
2057	4941	3706	1214	1240	1252	12	39	2931	1275
2058	4341	3256	1052	1087	1118	11	34	3086	1292
2059	3772	2829	901	941	987	10	30	3235	1307
2060	3240	2430	763	806	861	8	26	3377	1320
2061	2749	2062	638	682	742	7	22	3509	1331
2062	2302	1726	527	569	630	6	19	3632	1341
2063	1891	1418	427	466	525	5	15	3746	1349
2064	1528	1146	341	375	430	4	12	3848	1355
2065	1214	910	267	297	346	3	10	3938	1361
2066	948	711	206	231	274	3	8	4014	1365
2067	728	546	156	177	213	2	6	4079	1368
2068	548	411	116	133	162	2	4	4132	1370
2069	403	302	84	97	121	1	3	4175	1372

Year	Living with HCV	Chronic	Stage 0/1	Stage 2/3	Cirrhosis	HCC incidence	Liver failure incidence	Total deaths	HCV deaths
2070	288	216	60	69	87	1	2	4210	1374
2071	197	148	40	47	60	1	1	4238	1375
2072	126	95	26	30	39	0	1	4260	1375
2073	73	54	14	17	23	0	0	4277	1376
2074	32	24	6	8	10	0	0	4289	1376
2075	0	0	0	0	0	0	0	4299	1376

Note: Deaths are cumulative following cirrhosis

Table 3.5.5 Number of cases of chronic HCV by disease stage and diagnosis and HCV-deaths prevented by NSPs

Year	Chronic	Stage 0/1 diagnosed	Stage 0/1 undiagnosed	Stage2/3 diagnosed	Stage 2/3 undiagnosed	Cirrhosis diagnosed	Cirrhosis undiagnosed	HCC	Liver failure	HCV deaths
1988	757	445	297	11	4	0	0	0	0	0
1989	1805	1053	702	38	13	0	0	0	0	1
1990	2849	1646	1097	78	26	1	0	0	0	2
1991	3902	2233	1489	132	44	3	1	0	0	3
1992	4977	2822	1881	200	67	6	2	0	0	4
1993	6084	3419	2279	279	93	11	3	0	1	6
1994	7235	4031	2687	372	124	18	4	0	1	9
1995	8440	4663	3108	477	159	26	7	0	1	12
1996	9709	5321	3547	595	198	37	9	0	2	15
1997	11051	6011	4007	727	242	51	13	1	2	19
1998	12476	6737	4491	872	291	68	17	1	3	23
1999	13994	7505	5004	1031	344	88	22	1	4	28
2000	15615	8321	5547	1205	402	112	28	1	5	33
2001	15444	8068	5379	1367	456	139	35	2	6	39
2002	15273	7823	5216	1516	505	170	43	2	8	45
2003	15104	7586	5058	1654	551	204	51	3	9	50
2004	14943	7359	4906	1782	594	241	60	3	11	56
2005	14791	7143	4762	1901	634	281	70	3	12	62
2006	14649	6937	4625	2012	671	324	81	4	14	68
2007	14518	6741	4494	2116	705	369	92	5	16	73
2008	14397	6556	4370	2213	738	416	104	5	18	79
2009	14285	6379	4253	2304	768	465	116	6	20	84
2010	14185	6212	4142	2389	796	517	129	6	22	90
2011	14095	6054	4036	2469	823	570	143	7	24	95
2012	14016	5905	3937	2544	848	625	156	8	27	100
2013	13948	5765	3843	2616	872	682	170	8	29	105
2014	13892	5634	3756	2683	894	740	185	9	31	110
2015	13848	5511	3674	2748	916	800	200	10	34	114
2016	13818	5397	3598	2810	937	862	215	11	36	119
2017	13784	5284	3523	2867	956	924	231	11	39	123
2018	13747	5173	3449	2919	973	987	247	12	42	128
2019	13707	5063	3375	2966	989	1051	263	13	44	134
2020	13662	4955	3303	3008	1003	1115	279	14	47	139
2021	13614	4847	3232	3046	1015	1179	295	14	49	146
2022	13560	4741	3161	3079	1026	1243	311	15	52	153
2023	13501	4635	3090	3107	1036	1306	327	16	55	160

Year	Chronic	Stage 0/1 diagnosed	Stage 0/1 undiagnosed	Stage2/3 diagnosed	Stage 2/3 undiagnosed	Cirrhosis diagnosed	Cirrhosis undiagnosed	HCC	Liver failure	HCV deaths
2024	13436	4530	3020	3131	1044	1369	342	17	57	184
2025	13364	4425	2950	3149	1050	1432	358	18	60	212
2026	13284	4321	2881	3163	1054	1493	373	18	62	240
2027	13197	4216	2811	3172	1057	1552	388	19	65	270
2028	13101	4112	2741	3176	1059	1611	403	20	67	300
2029	12995	4007	2671	3175	1058	1667	417	20	69	332
2030	12879	3902	2601	3168	1056	1721	430	21	71	365
2031	12751	3796	2531	3157	1052	1772	443	22	74	398
2032	12611	3690	2460	3140	1047	1820	455	22	75	433
2033	12458	3582	2388	3117	1039	1865	466	23	77	469
2034	12291	3474	2316	3089	1030	1906	477	23	79	505
2035	12108	3364	2242	3055	1018	1943	486	24	80	542
2036	11909	3253	2168	3015	1005	1975	494	24	81	579
2037	11694	3140	2093	2969	990	2002	500	25	82	618
2038	11461	3026	2017	2917	972	2023	506	25	83	656
2039	11210	2911	1940	2859	953	2038	510	25	84	695
2040	10939	2793	1862	2794	931	2047	512	25	84	734
2041	10648	2674	1783	2723	908	2048	512	25	84	773
2042	10336	2554	1703	2645	882	2042	511	25	83	812
2043	10004	2432	1621	2561	854	2029	507	25	83	850
2044	9651	2308	1539	2471	824	2007	502	25	82	888
2045	9275	2183	1456	2375	792	1976	494	24	80	926
2046	8880	2057	1371	2272	757	1937	484	24	78	963
2047	8464	1930	1287	2164	721	1889	472	23	76	999
2048	8028	1803	1202	2051	684	1832	458	22	74	1034
2049	7576	1675	1117	1933	644	1766	441	22	71	1067
2050	7109	1548	1032	1811	604	1691	423	21	68	1100
2051	6629	1422	948	1686	562	1609	402	20	64	1130
2052	6140	1297	865	1559	520	1519	380	19	60	1159
2053	5646	1176	784	1431	477	1423	356	17	56	1186
2054	5151	1057	705	1302	434	1322	331	16	52	1211
2055	4659	942	628	1175	392	1217	304	15	48	1235
2056	4175	832	555	1051	350	1110	277	14	43	1256
2057	3706	728	486	930	310	1001	250	12	39	1275
2058	3256	631	421	815	272	894	224	11	34	1292
2059	2829	541	360	706	235	789	197	10	30	1307
2060	2430	458	305	605	202	689	172	8	26	1320

Year	Chronic	Stage 0/1 diagnosed	Stage 0/1 undiagnosed	Stage2/3 diagnosed	Stage 2/3 undiagnosed	Cirrhosis diagnosed	Cirrhosis undiagnosed	HCC	Liver failure	HCV deaths
2061	2062	383	255	511	170	593	148	7	22	1331
2062	1726	316	211	427	142	504	126	6	19	1341
2063	1418	256	171	349	116	420	105	5	15	1349
2064	1146	204	136	281	94	344	86	4	12	1355
2065	910	160	107	223	74	277	69	3	10	1361
2066	711	124	82	173	58	219	55	3	8	1365
2067	546	94	62	133	44	170	43	2	6	1368
2068	411	70	46	99	33	130	32	2	4	1370
2069	302	51	34	73	24	97	24	1	3	1372
2070	216	36	24	52	17	70	17	1	2	1374
2071	148	24	16	35	12	48	12	1	1	1375
2072	95	15	10	23	8	31	8	0	1	1375
2073	54	9	6	13	4	18	5	0	0	1376
2074	24	4	3	6	2	8	2	0	0	1376
2075	0	0	0	0	0	0	0	0	0	1376

Note: Deaths are cumulative following cirrhosis

APPENDIX D

DETAILED TABLES ON FINANCIAL EFFECTS AND NPV OF NSPS

		1991	1992	1993	1994	1995	1996	1997	1998	1999	2000
**NSP Expenditure**											
Total Govt NSP Expend		-$8,482,805	-$9,229,559	-$9,830,915	-$10,588,542	-$11,127,279	-$12,341,186	-$15,005,665	-$16,353,751	-$17,505,049	-$19,673,115
All NSP Expend		-$9,573,954	-$10,412,793	-$11,438,420	-$12,493,700	-$12,992,474	-$13,896,572	-$17,048,446	-$18,979,078	-$20,434,694	-$22,674,291
**HIV Effects - Cases Avoided**											
CD4 >500	Diagnosed	544	887	1,369	2,033	2,950	4,213	5,935	8,255	11,320	15,277
CD4 <500	Diagnosed	47	107	210	373	616	1,004	1,573	2,399	3,585	5,266
AIDS	Diagnosed	10	23	44	78	131	192	278	393	540	725
**Total Diag. HIV Cases Avoided**		601	1,017	1,623	2,484	3,697	5,409	7,786	11,047	15,445	21,268
**Annual Treatment Costs-HIV**											
CD4>500		$1,520,716	$2,479,550	$3,826,950	$5,683,119	$8,246,532	$11,777,166	$30,683,950	$42,678,350	$58,524,400	$78,982,090
CD4<500		$211,430	$481,341	$944,687	$1,677,944	$2,771,082	$4,516,505	$13,612,742	$20,760,946	$31,024,590	$45,571,964
AIDS		$837,771	$1,926,874	$3,686,194	$6,534,616	$10,974,805	$16,085,210	$13,882,473	$19,625,223	$26,965,955	$36,204,291
**Total HIV Costs Avoided**		$2,569,918	$4,887,765	$8,457,831	$13,895,679	$21,992,419	$32,378,880	$58,179,165	$83,064,519	$116,514,945	$160,758,345
**Net Govt Expenditure/Savings**		-$5,912,888	-$4,341,794	-$1,373,084	$3,307,137	$10,865,140	$20,037,694	$43,173,500	$66,710,768	$99,009,896	$141,085,230
**Net All Expend/Savings**		-$7,004,036	-$5,525,028	-$2,980,589	$1,401,979	$8,999,946	$18,482,308	$41,130,719	$64,085,440	$96,080,251	$138,084,054

**HIV**	**NPV Govt Expend @ 5%**	**All Years**	**$2,276,548,829**	**NPV All Expend @ 5%**	**All Years**	**$2,261,898,701**
		**To 2000**	**$241,702,597**		**To 2000**	**$227,052,469**
	**NPV Govt Expend @ 3%**	**All Years**	**$3,414,541,497**	**NPV All Expend @ 3%**	**All Years**	**$3,398,079,560**
		**To 2000**	**$286,631,780**		**To 2000**	**$270,169,843**
	**NPV Govt Expend @ 0%**	**All Years**	**$6,895,585,578**	**NPV All Expend @ 0%**	**All Years**	**$6,875,779,022**
		**To 2000**	**$372,561,599**		**To 2000**	**$352,755,044**

		1991	1992	1993	1994	1995	1996	1997	1998	1999	2000
**HCV Effects - Cases Avoided**											
Stage 0/1	Diagnosed	2,233	2,822	3,419	4,031	4,663	5,321	6,011	6,737	7,505	8,321
Stage 2/3	Diagnosed	132	200	279	372	477	595	727	872	1,031	1,205
Cirrhosis	Diagnosed	3	6	11	18	26	37	51	68	88	112
HCC	Diagnosed	0	0	0	0	0	0	1	1	1	1
Liver Failure	Diagnosed	0	0	1	1	1	2	2	3	4	5
**Total Diag. HCV Cases Avoided**		2,368	3,028	3,710	4,422	5,167	5,955	6,792	7,681	8,629	9,644
**Whole of HCV Episode Costs**		$123,272	$157,517	$192,752	$229,499	$267,914	$308,362	$351,207	$396,607	$444,925	$496,527
**Annual Treatment Costs-HCV**											
Stage 0/1		$453,299	$572,866	$694,057	$818,293	$946,589	$1,080,163	$1,220,233	$1,367,611	$1,523,515	$1,689,163
Stage 2/3		$26,796	$40,600	$56,637	$75,516	$96,831	$120,785	$147,581	$177,016	$209,293	$244,615
Cirrhosis		$1,140	$2,280	$4,180	$6,840	$9,880	$14,060	$19,380	$25,840	$33,440	$42,560
HCC		$0	$0	$0	$0	$0	$0	$100,770	$100,770	$100,770	$100,770
Liver Failure		$0	$0	$167,109	$167,109	$167,109	$334,219	$334,219	$501,328	$668,438	$835,547
**Total HCV Costs Avoided**		$604,507	$773,263	$1,114,736	$1,297,258	$1,488,324	$1,857,589	$2,173,390	$2,569,172	$2,980,381	$3,409,182
**Total Costs Avoided HIV + HCV**		$3,174,425	$5,661,028	$9,572,567	$15,192,937	$23,480,743	$34,236,469	$60,352,555	$85,633,691	$119,495,325	$164,167,527
**Net Govt Expenditure/Savings**		-$5,308,381	-<br>$3,568,531	-$258,349	$4,604,395	$12,353,463	$21,895,283	$45,346,890	$69,279,940	$101,990,277	$144,494,413
**Net All Expend/Savings**		-$6,399,529	-<br>$4,751,765	-$1,865,853	$2,699,237	$10,488,269	$20,339,897	$43,304,109	$66,654,612	$99,060,631	$141,493,236

**HIV+HCV**	**NPV Govt Expend @ 5%**	**All Years**	**$2,401,575,004**	**NPV All Expend @ 5%**	**All Years**	**$2,386,924,875**
		**To 2000**	**$254,859,826**		**To 2000**	**$240,209,698**
	**NPV Govt Expend @ 3%**	**All Years**	**$3,653,324,247**	**NPV All Expend @ 3%**	**All Years**	**$3,636,862,310**
		**To 2000**	**$301,576,093**		**To 2000**	**$285,114,155**
	**NPV Govt Expend @ 0%**	**All Years**	**$7,678,155,690**	**NPV All Expend @ 0%**	**All Years**	**$7,658,349,135**
		**To 2000**	**$390,829,399**		**To 2000**	**$371,022,844**

		2001	2002	2003	2004	2005	2006	2007	2008	2009	2010
**NSP Expenditure**											
Total Govt NSP Expend											
All NSP Expend											
**HIV Effects - Cases Avoided**											
CD4 >500	Diagnosed	12,681	9,833	7,142	4,896	3,230	2,105	1,363	876	560	356
CD4 <500	Diagnosed	7,551	10,027	12,247	13,901	14,865	15,196	15,094	14,733	14,210	13,603
AIDS	Diagnosed	946	1,219	1,521	1,834	2,130	2,392	2,602	2,746	2,828	2,849
**Total Diag. HIV Cases Avoided**		21,178	21,079	20,910	20,631	20,225	19,693	19,059	18,355	17,598	16,808
**Annual Treatment Costs-HIV**											
CD4>500		$65,560,770	$50,836,610	$36,924,140	$25,312,320	$16,699,100	$10,882,850	$7,046,710	$4,528,920	$2,895,200	$1,840,520
CD4<500		$65,346,354	$86,773,658	$105,985,538	$120,299,254	$128,641,710	$131,506,184	$130,623,476	$127,499,382	$122,973,340	$117,720,362
AIDS		$47,240,358	$60,873,146	$75,954,106	$91,584,372	$106,365,711	$119,449,192	$129,935,953	$137,126,874	$141,221,704	$142,270,380
**Total HIV Costs Avoided**		$178,147,482	$198,483,414	$218,863,784	$237,195,946	$251,706,521	$261,838,226	$267,606,139	$269,155,176	$267,090,244	$261,831,262

		2001	2002	2003	2004	2005	2006	2007	2008	2009	2010
**HCV Effects - Cases Avoided**											
Stage 0/1	Diagnosed	8,068	7,823	7,586	7,359	7,143	6,937	6,741	6,556	6,379	6,212
Stage 2/3	Diagnosed	1,367	1,516	1,654	1,782	1,901	2,012	2,116	2,213	2,304	2,389
Cirrhosis	Diagnosed	139	170	204	241	281	324	369	416	465	517
HCC	Diagnosed	2	2	3	3	3	4	5	5	6	6
Liver Failure	Diagnosed	6	8	9	11	12	14	16	18	20	22
**Total Diag. HCV Cases Avoided**		9,582	9,519	9,456	9,396	9,340	9,291	9,247	9,208	9,174	9,146
**Whole of HCV Episode Costs**		$491,784	$486,780	$481,620	$476,460	$471,404	$466,452	$461,657	$457,070	$452,587	$448,313
**Annual Treatment Costs-HCV**											
Stage 0/1		$1,637,804	$1,588,069	$1,539,958	$1,493,877	$1,450,029	$1,408,211	$1,368,423	$1,330,868	$1,294,937	$1,261,036
Stage 2/3		$277,501	$307,748	$335,762	$361,746	$385,903	$408,436	$429,548	$449,239	$467,712	$484,967
Cirrhosis		$52,820	$64,600	$77,520	$91,580	$106,780	$123,120	$140,220	$158,080	$176,700	$196,460
HCC		$201,539	$201,539	$302,309	$302,309	$302,309	$403,079	$503,848	$503,848	$604,618	$604,618
Liver Failure		$1,002,656	$1,336,875	$1,503,985	$1,838,203	$2,005,313	$2,339,532	$2,673,751	$3,007,969	$3,342,188	$3,676,407
**Total HCV Costs Avoided**		$3,664,105	$3,985,612	$4,241,154	$4,564,176	$4,721,738	$5,148,830	$5,577,447	$5,907,075	$6,338,743	$6,671,801
**Total Costs Avoided HIV + HCV**		$181,811,587	$202,469,026	$223,104,938	$241,760,122	$256,428,259	$266,987,056	$273,183,586	$275,062,251	$273,428,987	$268,503,064

		2011	2012	2013	2014	2015	2016	2017	2018	2019	2020
**NSP Expenditure**											
Total Govt NSP Expend											
All NSP Expend											
**HIV Effects - Cases Avoided**											
CD4 >500	Diagnosed	225	141	88	55	34	21	13	8	5	3
CD4 <500	Diagnosed	12,958	12,296	11,638	10,996	10,375	9,781	9,214	8,677	8,165	7,679
AIDS	Diagnosed	2,824	2,762	2,674	2,569	2,453	2,331	2,208	2,086	1,968	1,855
**Total Diag. HIV Cases Avoided**		16,007	15,199	14,400	13,620	12,862	12,133	11,435	10,771	10,138	9,537
**Annual Treatment Costs-HIV**											
CD4>500		$1,163,250	$728,970	$454,960	$284,350	$175,780	$108,570	$67,210	$41,360	$25,850	$15,510
CD4<500		$112,138,532	$106,409,584	$100,715,252	$95,159,384	$89,785,250	$84,644,774	$79,737,956	$75,090,758	$70,659,910	$66,454,066
AIDS		$141,021,956	$137,925,865	$133,531,413	$128,288,033	$122,495,347	$116,403,038	$110,260,793	$104,168,485	$98,275,924	$92,633,048
**Total HIV Costs Avoided**		$254,323,738	$245,064,419	$234,701,625	$223,731,767	$212,456,377	$201,156,382	$190,065,959	$179,300,603	$168,961,684	$159,102,624

		2011	2012	2013	2014	2015	2016	2017	2018	2019	2020
**HCV Effects - Cases Avoided**											
Stage 0/1	Diagnosed	6,054	5,905	5,765	5,634	5,511	5,397	5,284	5,173	5,063	4,955
Stage 2/3	Diagnosed	2,469	2,544	2,616	2,683	2,748	2,810	2,867	2,919	2,966	3,008
Cirrhosis	Diagnosed	570	625	682	740	800	862	924	987	1,051	1,115
HCC	Diagnosed	7	8	8	9	10	11	11	12	13	14
Liver Failure	Diagnosed	24	27	29	31	34	36	39	42	44	47
**Total Diag. HCV Cases Avoided**		9,124	9,109	9,100	9,097	9,103	9,116	9,125	9,133	9,137	9,139
**Whole of HCV Episode Costs**		$444,248	$440,391	$436,846	$433,510	$430,487	$427,777	$424,858	$421,783	$418,499	$415,059
**Annual Treatment Costs-HCV**											
Stage 0/1	Diagnosed	$1,228,962	$1,198,715	$1,170,295	$1,143,702	$1,118,733	$1,095,591	$1,072,652	$1,050,119	$1,027,789	$1,005,865
Stage 2/3	Diagnosed	$501,207	$516,432	$531,048	$544,649	$557,844	$570,430	$582,001	$592,557	$602,098	$610,624
Cirrhosis	Diagnosed	$216,600	$237,500	$259,160	$281,200	$304,000	$327,560	$351,120	$375,060	$399,380	$423,700
HCC	Diagnosed	$705,388	$806,158	$806,158	$906,927	$1,007,697	$1,108,467	$1,108,467	$1,209,236	$1,310,006	$1,410,776
Liver Failure	Diagnosed	$4,010,626	$4,511,954	$4,846,173	$5,180,392	$5,681,720	$6,015,939	$6,517,267	$7,018,595	$7,352,814	$7,854,142
**Total HCV Costs Avoided**		$7,107,030	$7,711,149	$8,049,680	$8,490,380	$9,100,481	$9,545,763	$10,056,364	$10,667,350	$11,110,586	$11,720,166
**Total Costs Avoided HIV + HCV**		$261,430,769	$252,775,568	$242,751,305	$232,222,147	$221,556,858	$210,702,145	$200,122,323	$189,967,953	$180,072,270	$170,822,790

		2021	2022	2023	2024	2025	2026	2027	2028	2029	2030
**NSP Expenditure**											
Total Govt NSP Expend											
All NSP Expend											
**HIV Effects - Cases Avoided**											
CD4 >500	Diagnosed	2	1	1	0	0	0	0	0	0	0
CD4 <500	Diagnosed	7,219	6,784	6,371	5,980	5,610	5,258	4,925	4,608	4,307	4,021
AIDS	Diagnosed	1,747	1,644	1,546	1,453	1,365	1,281	1,202	1,127	1,056	988
**Total Diag. HIV Cases Avoided**		8,968	8,429	7,918	7,433	6,975	6,539	6,127	5,735	5,363	5,009
**Annual Treatment Costs-HIV**											
CD4>500		$10,340	$5,170	$5,170	$0	$0	$0	$0	$0	$0	$0
CD4<500		$62,473,226	$58,708,736	$55,134,634	$51,750,920	$48,548,940	$45,502,732	$42,620,950	$39,877,632	$37,272,778	$34,797,734
AIDS		$87,239,857	$82,096,351	$77,202,530	$72,558,393	$68,163,941	$63,969,237	$60,024,218	$56,278,946	$52,733,423	$49,337,710
**Total HIV Costs Avoided**		$149,723,423	$140,810,257	$132,342,334	$124,309,313	$116,712,881	$109,471,969	$102,645,168	$96,156,578	$90,006,201	$84,135,444

		2021	2022	2023	2024	2025	2026	2027	2028	2029	2030
**HCV Effects - Cases Avoided**											
Stage 0/1	Diagnosed	4,847	4,741	4,635	4,530	4,425	4,321	4,216	4,112	4,007	3,902
Stage 2/3	Diagnosed	3,046	3,079	3,107	3,131	3,149	3,163	3,172	3,176	3,175	3,168
Cirrhosis	Diagnosed	1,179	1,243	1,306	1,369	1,432	1,493	1,552	1,611	1,667	1,721
HCC	Diagnosed	14	15	16	17	18	18	19	20	20	21
Liver Failure	Diagnosed	49	52	55	57	60	62	65	67	69	71
**Total Diag. HCV Cases Avoided**		9,135	9,130	9,119	9,104	9,084	9,057	9,024	8,986	8,938	8,883
**Whole of HCV Episode Costs**		$411,410	$407,605	$403,539	$399,317	$394,783	$390,092	$385,088	$379,875	$374,350	$368,512
**Annual Treatment Costs-HCV**											
Stage 0/1	Diagnosed	$983,941	$962,423	$940,905	$919,590	$898,275	$877,163	$855,848	$834,736	$813,421	$792,106
Stage 2/3	Diagnosed	$618,338	$625,037	$630,721	$635,593	$639,247	$642,089	$643,916	$644,728	$644,525	$643,104
Cirrhosis	Diagnosed	$448,020	$472,340	$496,280	$520,220	$544,160	$567,340	$589,760	$612,180	$633,460	$653,980
HCC	Diagnosed	$1,410,776	$1,511,545	$1,612,315	$1,713,085	$1,813,854	$1,813,854	$1,914,624	$2,015,394	$2,015,394	$2,116,164
Liver Failure	Diagnosed	$8,188,361	$8,689,689	$9,191,017	$9,525,236	$10,026,565	$10,360,783	$10,862,112	$11,196,330	$11,530,549	$11,864,768
**Total HCV Costs Avoided**		$12,060,846	$12,668,640	$13,274,778	$13,713,041	$14,316,884	$14,651,321	$15,251,347	$15,683,244	$16,011,699	$16,438,634
**Total Costs Avoided HIV + HCV**		$161,784,269	$153,478,897	$145,617,112	$138,022,355	$131,029,765	$124,123,291	$117,896,515	$111,839,822	$106,017,900	$100,574,078

		2031	2032	2033	2034	2035	2036	2037	2038	2039	2040
**NSP Expenditure**											
Total Govt NSP Expend											
All NSP Expend											
**HIV Effects - Cases Avoided**											
CD4 >500	Diagnosed	0	0	0	0	0	0	0	0	0	0
CD4 <500	Diagnosed	3,750	3,492	3,248	3,016	2,796	2,587	2,388	2,199	2,021	1,851
AIDS	Diagnosed	924	864	806	751	699	650	603	558	516	476
**Total Diag. HIV Cases Avoided**		4,674	4,356	4,054	3,767	3,495	3,237	2,991	2,757	2,537	2,327
**Annual Treatment Costs-HIV**											
CD4>500		$0	$0	$0	$0	$0	$0	$0	$0	$0	$0
CD4<500		$32,452,500	$30,219,768	$28,108,192	$26,100,464	$24,196,584	$22,387,898	$20,665,752	$19,030,146	$17,489,734	$16,018,554
AIDS		$46,141,745	$43,145,528	$40,249,184	$37,502,652	$34,905,930	$32,459,020	$30,111,983	$27,864,820	$25,767,468	$23,769,990
**Total HIV Costs Avoided**		$78,594,245	$73,365,296	$68,357,376	$63,603,116	$59,102,514	$54,846,918	$50,777,735	$46,894,966	$43,257,202	$39,788,544

		2031	2032	2033	2034	2035	2036	2037	2038	2039	2040
**HCV Effects - Cases Avoided**											
Stage 0/1	Diagnosed	3,796	3,690	3,582	3,474	3,364	3,253	3,140	3,026	2,911	2,793
Stage 2/3	Diagnosed	3,157	3,140	3,117	3,089	3,055	3,015	2,969	2,917	2,859	2,794
Cirrhosis	Diagnosed	1,772	1,820	1,865	1,906	1,943	1,975	2,002	2,023	2,038	2,047
HCC	Diagnosed	22	22	23	23	24	24	25	25	25	25
Liver Failure	Diagnosed	74	75	77	79	80	81	82	83	84	84
**Total Diag. HCV Cases Avoided**		8,821	8,747	8,664	8,571	8,466	8,348	8,218	8,074	7,917	7,743
**Whole of HCV Episode Costs**		$362,414	$356,003	$349,175	$342,086	$334,580	$326,709	$318,422	$309,769	$300,752	$291,213
**Annual Treatment Costs-HCV**											
Stage 0/1		$770,588	$749,070	$727,146	$705,222	$682,892	$660,359	$637,420	$614,278	$590,933	$566,979
Stage 2/3		$640,871	$637,420	$632,751	$627,067	$620,165	$612,045	$602,707	$592,151	$580,377	$567,182
Cirrhosis		$673,360	$691,600	$708,700	$724,280	$738,340	$750,500	$760,760	$768,740	$774,440	$777,860
HCC		$2,216,933	$2,216,933	$2,317,703	$2,317,703	$2,418,473	$2,418,473	$2,519,242	$2,519,242	$2,519,242	$2,519,242
Liver Failure		$12,366,096	$12,533,206	$12,867,424	$13,201,643	$13,368,753	$13,535,862	$13,702,972	$13,870,081	$14,037,190	$14,037,190
**Total HCV Costs Avoided**		$17,030,262	$17,184,232	$17,602,899	$17,918,001	$18,163,202	$18,303,948	$18,541,523	$18,674,262	$18,802,935	$18,759,667
**Total Costs Avoided HIV + HCV**		$95,624,507	$90,549,527	$85,960,275	$81,521,117	$77,265,717	$73,150,866	$69,319,258	$65,569,228	$62,060,137	$58,548,211

		2041	2042	2043	2044	2045	2046	2047	2048	2049	2050
**NSP Expenditure**											
Total Govt NSP Expend											
All NSP Expend											
**HIV Effects - Cases Avoided**											
CD4 >500	Diagnosed	0	0	0	0	0	0	0	0	0	0
CD4 <500	Diagnosed	1,690	1,539	1,395	1,260	1,132	1,013	901	796	699	609
AIDS	Diagnosed	438	402	368	336	305	276	249	224	200	178
**Total Diag. HIV Cases Avoided**		2,128	1,941	1,763	1,596	1,437	1,289	1,150	1,020	899	787
**Annual Treatment Costs-HIV**											
CD4>500		$0	$0	$0	$0	$0	$0	$0	$0	$0	$0
CD4<500		$14,625,260	$13,318,506	$12,072,330	$10,904,040	$9,796,328	$8,766,502	$7,797,254	$6,888,584	$6,049,146	$5,270,286
AIDS		$21,872,386	$20,074,655	$18,376,799	$16,778,816	$15,230,771	$13,782,599	$12,434,301	$11,185,878	$9,987,391	$8,888,778
**Total HIV Costs Avoided**		$36,497,646	$33,393,161	$30,449,129	$27,682,856	$25,027,099	$22,549,101	$20,231,555	$18,074,462	$16,036,537	$14,159,064

		2041	2042	2043	2044	2045	2046	2047	2048	2049	2050
**HCV Effects - Cases Avoided**											
Stage 0/1	Diagnosed	2,674	2,554	2,432	2,308	2,183	2,057	1,930	1,803	1,675	1,548
Stage 2/3	Diagnosed	2,723	2,645	2,561	2,471	2,375	2,272	2,164	2,051	1,933	1,811
Cirrhosis	Diagnosed	2,048	2,042	2,029	2,007	1,976	1,937	1,889	1,832	1,766	1,691
HCC	Diagnosed	25	25	25	25	24	24	23	22	22	21
Liver Failure	Diagnosed	84	83	83	82	80	78	76	74	71	68
**Total Diag. HCV Cases Avoided**		7,554	7,349	7,130	6,893	6,638	6,368	6,082	5,782	5,467	5,139
**Whole of HCV Episode Costs**		$281,310	$270,990	$260,252	$249,098	$237,578	$225,642	$213,393	$200,884	$188,061	$175,082
**Annual Treatment Costs-HCV**											
Stage 0/1		$542,822	$518,462	$493,696	$468,524	$443,149	$417,571	$391,790	$366,009	$340,025	$314,244
Stage 2/3		$552,769	$536,935	$519,883	$501,613	$482,125	$461,216	$439,292	$416,353	$392,399	$367,633
Cirrhosis		$778,240	$775,960	$771,020	$762,660	$750,880	$736,060	$717,820	$696,160	$671,080	$642,580
HCC		$2,519,242	$2,519,242	$2,519,242	$2,519,242	$2,418,473	$2,418,473	$2,317,703	$2,216,933	$2,216,933	$2,116,164
Liver Failure		$14,037,190	$13,870,081	$13,870,081	$13,702,972	$13,368,753	$13,034,534	$12,700,315	$12,366,096	$11,864,768	$11,363,440
**Total HCV Costs Avoided**		$18,711,574	$18,491,670	$18,434,174	$18,204,109	$17,700,958	$17,293,496	$16,780,313	$16,262,435	$15,673,267	$14,979,143
**Total Costs Avoided HIV + HCV**		$55,209,219	$51,884,831	$48,883,303	$45,886,965	$42,728,057	$39,842,597	$37,011,869	$34,336,897	$31,709,803	$29,138,207

		2051	2052	2053	2054	2055	2056	2057	2058	2059	2060
**NSP Expenditure**											
Total Govt NSP Expend											
All NSP Expend											
**HIV Effects - Cases Avoided**											
CD4 >500	Diagnosed	0	0	0	0	0	0	0	0	0	0
CD4 <500	Diagnosed	526	451	382	320	265	217	175	139	109	84
AIDS	Diagnosed	157	137	119	103	88	75	63	52	42	34
**Total Diag. HIV Cases Avoided**		683	588	501	423	353	292	238	191	151	118
**Annual Treatment Costs-HIV**											
CD4>500		$0	$0	$0	$0	$0	$0	$0	$0	$0	$0
CD4<500		$4,552,004	$3,902,954	$3,305,828	$2,769,280	$2,293,310	$1,877,918	$1,514,450	$1,202,906	$943,286	$726,936
AIDS		$7,840,102	$6,841,363	$5,942,497	$5,143,506	$4,394,452	$3,745,272	$3,146,028	$2,596,722	$2,097,352	$1,697,856
**Total HIV Costs Avoided**		$12,392,106	$10,744,317	$9,248,325	$7,912,786	$6,687,762	$5,623,190	$4,660,478	$3,799,628	$3,040,638	$2,424,792

		2051	2052	2053	2054	2055	2056	2057	2058	2059	2060
**HCV Effects - Cases Avoided**											
Stage 0/1	Diagnosed	1,422	1,297	1,176	1,057	942	832	728	631	541	458
Stage 2/3	Diagnosed	1,686	1,559	1,431	1,302	1,175	1,051	930	815	706	605
Cirrhosis	Diagnosed	1,609	1,519	1,423	1,322	1,217	1,110	1,001	894	789	689
HCC	Diagnosed	20	19	17	16	15	14	12	11	10	8
Liver Failure	Diagnosed	64	60	56	52	48	43	39	34	30	26
**Total Diag. HCV Cases Avoided**		4,801	4,454	4,103	3,749	3,397	3,050	2,710	2,385	2,076	1,786
**Whole of HCV Episode Costs**		$162,000	$148,864	$135,886	$122,959	$110,345	$98,148	$86,421	$75,370	$64,998	$55,407
**Annual Treatment Costs-HCV**											
Stage 0/1		$288,666	$263,291	$238,728	$214,571	$191,226	$168,896	$147,784	$128,093	$109,823	$92,974
Stage 2/3		$342,258	$316,477	$290,493	$264,306	$238,525	$213,353	$188,790	$165,445	$143,318	$122,815
Cirrhosis		$611,420	$577,220	$540,740	$502,360	$462,460	$421,800	$380,380	$339,720	$299,820	$261,820
HCC		$2,015,394	$1,914,624	$1,713,085	$1,612,315	$1,511,545	$1,410,776	$1,209,236	$1,108,467	$1,007,697	$806,158
Liver Failure		$10,695,002	$10,026,565	$9,358,127	$8,689,689	$8,021,252	$7,185,705	$6,517,267	$5,681,720	$5,013,282	$4,344,845
**Total HCV Costs Avoided**		$14,114,740	$13,247,041	$12,277,058	$11,406,200	$10,535,353	$9,498,678	$8,529,878	$7,498,815	$6,638,938	$5,684,018
**Total Costs Avoided HIV + HCV**		$26,506,845	$23,991,358	$21,525,384	$19,318,987	$17,223,115	$15,121,867	$13,190,356	$11,298,443	$9,679,576	$8,108,811

		2061	2062	2063	2064	2065	2066	2067	2068	2069	2070
**NSP Expenditure**											
Total Govt NSP Expend											
All NSP Expend											
**HIV Effects - Cases Avoided**											
CD4 >500	Diagnosed	0	0	0	0	0	0	0	0	0	0
CD4 <500	Diagnosed	63	47	35	25	18	12	8	5	2	0
AIDS	Diagnosed	27	21	16	12	8	6	4	2	1	0
**Total Diag. HIV Cases Avoided**		90	68	51	37	26	18	12	7	3	0
**Annual Treatment Costs-HIV**											
CD4>500		$0	$0	$0	$0	$0	$0	$0	$0	$0	$0
CD4<500		$545,202	$406,738	$302,890	$216,350	$155,772	$103,848	$69,232	$43,270	$17,308	$0
AIDS		$1,348,298	$1,048,676	$798,991	$599,243	$399,496	$299,622	$199,748	$99,874	$49,937	$0
**Total HIV Costs Avoided**		$1,893,500	$1,455,414	$1,101,881	$815,593	$555,268	$403,470	$268,980	$143,144	$67,245	$0

		2061	2062	2063	2064	2065	2066	2067	2068	2069	2070
**HCV Effects - Cases Avoided**											
Stage 0/1	Diagnosed	383	316	256	204	160	124	94	70	51	36
Stage 2/3	Diagnosed	511	427	349	281	223	173	133	99	73	52
Cirrhosis	Diagnosed	593	504	420	344	277	219	170	130	97	70
HCC	Diagnosed	7	6	5	4	3	3	2	2	1	1
Liver Failure	Diagnosed	22	19	15	12	10	8	6	4	3	2
**Total Diag. HCV Cases Avoided**		1,516	1,272	1,045	845	673	527	405	305	225	161
**Whole of HCV Episode Costs**		$46,598	$38,728	$31,535	$25,280	$19,963	$15,481	$11,832	$8,809	$6,463	$4,587
**Annual Treatment Costs-HCV**											
Stage 0/1		$77,749	$64,148	$51,968	$41,412	$32,480	$25,172	$19,082	$14,210	$10,353	$7,308
Stage 2/3		$103,733	$86,681	$70,847	$57,043	$45,269	$35,119	$26,999	$20,097	$14,819	$10,556
Cirrhosis		$225,340	$191,520	$159,600	$130,720	$105,260	$83,220	$64,600	$49,400	$36,860	$26,600
HCC		$705,388	$604,618	$503,848	$403,079	$302,309	$302,309	$201,539	$201,539	$100,770	$100,770
Liver Failure		$3,676,407	$3,175,079	$2,506,641	$2,005,313	$1,671,094	$1,336,875	$1,002,656	$668,438	$501,328	$334,219
**Total HCV Costs Avoided**		$4,835,215	$4,160,774	$3,324,439	$2,662,847	$2,176,375	$1,798,176	$1,326,709	$962,493	$670,593	$484,039
**Total Costs Avoided HIV + HCV**		$6,728,715	$5,616,188	$4,426,321	$3,478,440	$2,731,643	$2,201,646	$1,595,689	$1,105,637	$737,838	$484,039

		2071	2072	2073	2074	2075	Total
**NSP Expenditure**							
Total Govt NSP Expend							-$130,137,868
All NSP Expend							-$149,944,423
**HIV Effects - Cases Avoided**							
CD4 >500	Diagnosed						96,422
CD4 <500	Diagnosed						344,734
AIDS	Diagnosed						71,410
**Total Diag. HIV Cases Avoided**							512,566
**Annual Treatment Costs-HIV**							
CD4>500							$470,016,453
CD4<500							$2,973,533,547
AIDS							$3,582,173,445
**Total HIV Costs Avoided**							$7,025,723,445
**Net Govt Expenditure/Savings**							$6,895,585,578
**Net All Expend/Savings**							$6,875,779,022

		2071	2072	2073	2074	2075	Total
**HCV Effects - Cases Avoided**							
Stage 0/1	Diagnosed	24	15	9	4	0	285,367
Stage 2/3	Diagnosed	35	23	13	6	0	150,916
Cirrhosis	Diagnosed	48	31	18	8	0	79,585
HCC	Diagnosed	1	0	0	0	0	975
Liver Failure	Diagnosed	1	1	0	0	0	3,248
**Total Diag. HCV Cases Avoided**		109	70	40	18	0	520,091
**Whole of HCV Episode Costs**		$3,075	$1,981	$1,147	$521	$0	$22,740,552
**Annual Treatment Costs-HCV**							
Stage 0/1		$4,872	$3,045	$1,827	$812	$0	$57,929,501
Stage 2/3		$7,105	$4,669	$2,639	$1,218	$0	$30,635,948
Cirrhosis		$18,240	$11,780	$6,840	$3,040	$0	$30,242,300
HCC		$100,770	$0	$0	$0	$0	$98,250,451
Liver Failure		$167,109	$167,109	$0	$0	$0	$542,771,360
**Total HCV Costs Avoided**		$301,171	$188,584	$12,453	$5,591	$0	$782,570,112
**Total Costs Avoided HIV + HCV**		$301,171	$188,584	$12,453	$5,591	$0	$7,808,293,558
**Net Govt Expenditure/Savings**		$301,171	$188,584	$12,453	$5,591	$0	$7,678,155,690
**Net All Expend/Savings**		$301,171	$188,584	$12,453	$5,591	$0	$7,658,349,135

APPENDIX E

DETAILED TABLES ON QUALITY OF LIFE EFFECTS OF NSPS

Year		1991	1992	1993	1994	1995	1996	1997	1998	1999	2000
**HIV**											
**Survivors without HIV**		786	1,309	2,070	3,153	4,684	6,828	9,797	13,853	19,304	26,488
**Survivors with HIV**											
CD4 >500	Diagnosed	544	887	1,369	2,033	2,950	4,213	5,935	8,255	11,320	15,277
CD4 >500	Undiagnosed	136	222	342	508	738	1,053	1,484	2,064	2,830	3,819
CD4 <500	Diagnosed	47	107	210	373	616	1,004	1,573	2,399	3,585	5,266
CD4 <500	Undiagnosed	5	12	23	41	68	112	175	267	398	585
AIDS	Diagnosed	10	23	44	78	131	192	278	393	540	725
**Total**		742	1,251	1,988	3,033	4,503	6,574	9,445	13,378	18,673	25,672
**Life Years Gained**		44	58	82	120	181	254	352	475	631	816
**Quality Adjusted Life Years**											
CD4 >500	Diagnosed	473	772	1,191	1,769	2,567	3,665	5,163	7,182	9,848	13,291
CD4 >500	Undiagnosed	128	209	321	478	694	990	1,395	1,940	2,660	3,590
CD4 <500	Diagnosed	36	81	160	283	468	763	1,195	1,823	2,725	4,002
CD4 <500	Undiagnosed	5	11	21	37	61	101	158	240	358	527
AIDS	Diagnosed	6	14	27	48	81	119	172	244	335	450
**Total**		648	1,087	1,720	2,615	3,871	5,638	8,084	11,429	15,926	21,859
**HIV QALYs Gained**		138	222	350	538	814	1,190	1,713	2,424	3,378	4,629

Year		1991	1992	1993	1994	1995	1996	1997	1998	1999	2000
**HCV**											
**Survivors without HCV**		5,204	6,637	8,114	9,650	11,256	12,949	14,738	16,639	18,663	20,826
**Survivors with HCV**											
Not Chronic		1,300	1,658	2,028	2,412	2,814	3,236	3,683	4,158	4,665	5,205
Stage 0/1	Diagnosed	2,233	2,822	3,419	4,031	4,663	5,321	6,011	6,737	7,505	8,321
Stage 0/1	Undiagnosed	1,489	1,881	2,279	2,687	3,108	3,547	4,007	4,491	5,004	5,547
Stage 2/3	Diagnosed	132	200	279	372	477	595	727	872	1,031	1,205
Stage 2/3	Undiagnosed	44	67	93	124	159	198	242	291	344	402
Cirrhosis	Diagnosed	3	6	11	18	26	37	51	68	88	112
Cirrhosis	Undiagnosed	1	2	3	4	7	9	13	17	22	28
HCC	Diagnosed	0	0	0	0	0	0	1	1	1	1
Liver Failure	Diagnosed	0	0	1	1	1	2	2	3	4	5
**Total**		5,202	6,636	8,112	9,648	11,254	12,943	14,734	16,634	18,659	20,820
**Life Years Gained**		2	1	2	2	2	6	4	5	4	6
**Quality Adjusted Life Years**											
Not Chronic		1,300	1,658	2,028	2,412	2,814	3,236	3,683	4,158	4,665	5,205
Stage 0/1	Diagnosed	1,831	2,314	2,804	3,305	3,824	4,363	4,929	5,524	6,154	6,823
Stage 0/1	Undiagnosed	1,400	1,768	2,142	2,526	2,922	3,334	3,767	4,222	4,704	5,214
Stage 2/3	Diagnosed	108	164	229	305	391	488	596	715	845	988
Stage 2/3	Undiagnosed	41	63	87	117	149	186	227	274	323	378
Cirrhosis	Diagnosed	2	4	7	13	19	26	36	47	61	78
Cirrhosis	Undiagnosed	1	2	3	3	6	8	11	14	18	24
HCC	Diagnosed	0	0	0	0	0	0	0	0	0	0
Liver Failure	Diagnosed	0	0	0	0	0	1	1	1	1	2
**Total**		4,683	5,973	7,300	8,681	10,124	11,642	13,249	14,955	16,773	18,712
**HCV QALYs Gained**		520	664	814	969	1,132	1,307	1,489	1,683	1,891	2,114
**All Life Years Gained**		45	59	84	122	184	260	356	479	636	821
**All QALYs Gained**		658	886	1,164	1,507	1,945	2,497	3,202	4,107	5,269	6,742
**HIV**	**NPV All Years @5%**		**138,072**	**HCV**	**NPV All Years @5%**		**32,207**	**HIV+HCV**	**NPV All Years @5%**		**170,279**
	**NPV All Years @3%**		**248,364**		**NPV All Years @3%**		**50,041**		**NPV All Years @3%**		**298,406**
	**NPV All Years @0%**		**715,245**		**NPV All Years @0%**		**119,992**		**NPV All Years @0%**		**835,237**

Year		2001	2002	2003	2004	2005	2006	2007	2008	2009	2010
**HIV**											
**Survivors without HIV**		26,190	25,898	25,608	25,317	25,035	24,756	24,477	24,208	23,944	23,691
**Survivors with HIV**											
CD4 >500	Diagnosed	12,681	9,833	7,142	4,896	3,230	2,105	1,363	876	560	356
CD4 >500	Undiagnosed	3,170	2,458	1,785	1,224	807	526	341	219	140	89
CD4 <500	Diagnosed	7,551	10,027	12,247	13,901	14,865	15,196	15,094	14,733	14,210	13,603
CD4 <500	Undiagnosed	839	1,114	1,361	1,545	1,652	1,688	1,677	1,637	1,579	1,511
AIDS	Diagnosed	946	1,219	1,521	1,834	2,130	2,392	2,602	2,746	2,828	2,849
**Total**		25,187	24,651	24,056	23,400	22,684	21,907	21,077	20,211	19,317	18,408
**Life Years Gained**		1,003	1,247	1,552	1,917	2,351	2,849	3,400	3,997	4,627	5,283
**Quality Adjusted Life Years**											
CD4 >500	Diagnosed	11,032	8,555	6,214	4,260	2,810	1,831	1,186	762	487	310
CD4 >500	Undiagnosed	2,980	2,311	1,678	1,151	759	494	321	206	132	84
CD4 <500	Diagnosed	5,739	7,621	9,308	10,565	11,297	11,549	11,471	11,197	10,800	10,338
CD4 <500	Undiagnosed	755	1,003	1,225	1,391	1,487	1,519	1,509	1,473	1,421	1,360
AIDS	Diagnosed	587	756	943	1,137	1,321	1,483	1,613	1,703	1,753	1,766
**Total**		21,093	20,244	19,367	18,502	17,673	16,877	16,100	15,341	14,593	13,858
**HIV QALYs Gained**		5,097	5,654	6,241	6,815	7,362	7,879	8,376	8,867	9,351	9,833

Year		2001	2002	2003	2004	2005	2006	2007	2008	2009	2010
**HCV**											
**Survivors without HCV**		20,597	20,370	20,145	19,930	19,727	19,538	19,363	19,202	19,053	18,919
**Survivors with HCV**											
Not Chronic		5,148	5,091	5,035	4,981	4,930	4,883	4,840	4,799	4,762	4,728
Stage 0/1	Diagnosed	8,068	7,823	7,586	7,359	7,143	6,937	6,741	6,556	6,379	6,212
Stage 0/1	Undiagnosed	5,379	5,216	5,058	4,906	4,762	4,625	4,494	4,370	4,253	4,142
Stage 2/3	Diagnosed	1,367	1,516	1,654	1,782	1,901	2,012	2,116	2,213	2,304	2,389
Stage 2/3	Undiagnosed	456	505	551	594	634	671	705	738	768	796
Cirrhosis	Diagnosed	139	170	204	241	281	324	369	416	465	517
Cirrhosis	Undiagnosed	35	43	51	60	70	81	92	104	116	129
HCC	Diagnosed	2	2	3	3	3	4	5	5	6	6
Liver Failure	Diagnosed	6	8	9	11	12	14	16	18	20	22
**Total**		20,592	20,364	20,139	19,923	19,721	19,533	19,357	19,196	19,047	18,913
**Life Years Gained**		5	6	6	7	6	5	6	6	6	6
**Quality Adjusted Life Years**											
Not Chronic		5,148	5,091	5,035	4,981	4,930	4,883	4,840	4,799	4,762	4,728
Stage 0/1	Diagnosed	6,616	6,415	6,221	6,034	5,857	5,688	5,528	5,376	5,231	5,094
Stage 0/1	Undiagnosed	5,056	4,903	4,755	4,612	4,476	4,348	4,224	4,108	3,998	3,893
Stage 2/3	Diagnosed	1,121	1,243	1,356	1,461	1,559	1,650	1,735	1,815	1,889	1,959
Stage 2/3	Undiagnosed	429	475	518	558	596	631	663	694	722	748
Cirrhosis	Diagnosed	97	118	142	168	197	226	258	291	325	362
Cirrhosis	Undiagnosed	29	36	43	50	59	68	77	87	97	108
HCC	Diagnosed	0	0	0	0	0	0	1	1	1	1
Liver Failure	Diagnosed	2	3	3	4	4	4	5	6	6	7
**Total**		18,498	18,284	18,072	17,869	17,678	17,499	17,330	17,176	17,031	16,900
**HCV QALYs Gained**		2,099	2,086	2,073	2,061	2,049	2,039	2,033	2,026	2,022	2,018
**All Life Years Gained**		1,008	1,253	1,558	1,924	2,357	2,854	3,406	4,002	4,633	5,289
**All QALYs Gained**		7,196	7,740	8,313	8,876	9,411	9,918	10,409	10,893	11,373	11,852

Year		2011	2012	2013	2014	2015	2016	2017	2018	2019	2020
**HIV**											
**Survivors without HIV**		23,455	23,234	23,037	22,874	22,755	22,693	22,625	22,553	22,470	22,380
**Survivors with HIV**											
CD4 >500	Diagnosed	225	141	88	55	34	21	13	8	5	3
CD4 >500	Undiagnosed	56	35	22	14	9	5	3	2	1	1
CD4 <500	Diagnosed	12,958	12,296	11,638	10,996	10,375	9,781	9,214	8,677	8,165	7,679
CD4 <500	Undiagnosed	1,440	1,366	1,293	1,222	1,153	1,087	1,024	964	907	853
AIDS	Diagnosed	2,824	2,762	2,674	2,569	2,453	2,331	2,208	2,086	1,968	1,855
**Total**		17,503	16,600	15,715	14,856	14,024	13,225	12,462	11,737	11,046	10,391
**Life Years Gained**		5,952	6,634	7,322	8,018	8,731	9,468	10,163	10,816	11,424	11,989
**Quality Adjusted Life Years**											
CD4 >500	Diagnosed	196	123	77	48	30	18	11	7	4	3
CD4 >500	Undiagnosed	53	33	21	13	8	5	3	2	1	1
CD4 <500	Diagnosed	9,848	9,345	8,845	8,357	7,885	7,434	7,003	6,595	6,205	5,836
CD4 <500	Undiagnosed	1,296	1,229	1,164	1,100	1,038	978	922	868	816	768
AIDS	Diagnosed	1,751	1,712	1,658	1,593	1,521	1,445	1,369	1,293	1,220	1,150
**Total**		13,143	12,442	11,764	11,111	10,482	9,880	9,307	8,764	8,247	7,757
**HIV QALYs Gained**		10,312	10,792	11,273	11,763	12,274	12,813	13,318	13,789	14,223	14,623

Year		2011	2012	2013	2014	2015	2016	2017	2018	2019	2020
**HCV**											
**Survivors without HCV**		18,799	18,693	18,603	18,528	18,469	18,428	18,384	18,334	18,281	18,222
**Survivors with HCV**											
Not Chronic		4,699	4,672	4,650	4,631	4,617	4,606	4,595	4,582	4,569	4,555
Stage 0/1	Diagnosed	6,054	5,905	5,765	5,634	5,511	5,397	5,284	5,173	5,063	4,955
Stage 0/1	Undiagnosed	4,036	3,937	3,843	3,756	3,674	3,598	3,523	3,449	3,375	3,303
Stage 2/3	Diagnosed	2,469	2,544	2,616	2,683	2,748	2,810	2,867	2,919	2,966	3,008
Stage 2/3	Undiagnosed	823	848	872	894	916	937	956	973	989	1,003
Cirrhosis	Diagnosed	570	625	682	740	800	862	924	987	1,051	1,115
Cirrhosis	Undiagnosed	143	156	170	185	200	215	231	247	263	279
HCC	Diagnosed	7	8	8	9	10	11	11	12	13	14
Liver Failure	Diagnosed	24	27	29	31	34	36	39	42	44	47
**Total**		18,794	18,687	18,598	18,523	18,466	18,425	18,380	18,330	18,276	18,218
**Life Years Gained**		5	6	5	5	3	3	4	4	5	4
**Quality Adjusted Life Years**											
Not Chronic		4,699	4,672	4,650	4,631	4,617	4,606	4,595	4,582	4,569	4,555
Stage 0/1	Diagnosed	4,964	4,842	4,727	4,620	4,519	4,426	4,333	4,242	4,152	4,063
Stage 0/1	Undiagnosed	3,794	3,701	3,612	3,531	3,454	3,382	3,312	3,242	3,173	3,105
Stage 2/3	Diagnosed	2,025	2,086	2,145	2,200	2,253	2,304	2,351	2,394	2,432	2,467
Stage 2/3	Undiagnosed	774	797	820	840	861	881	899	915	930	943
Cirrhosis	Diagnosed	399	437	477	518	559	603	647	690	736	780
Cirrhosis	Undiagnosed	120	131	143	155	168	181	194	207	221	234
HCC	Diagnosed	1	1	1	1	1	1	1	1	1	1
Liver Failure	Diagnosed	8	9	9	10	11	12	12	13	14	15
**Total**		16,783	16,675	16,585	16,506	16,443	16,395	16,343	16,287	16,227	16,163
**HCV QALYs Gained**		2,016	2,018	2,018	2,022	2,026	2,034	2,040	2,048	2,054	2,059
**All Life Years Gained**		5,957	6,640	7,327	8,023	8,734	9,472	10,167	10,820	11,429	11,994
**All QALYs Gained**		12,328	12,809	13,292	13,785	14,300	14,847	15,358	15,837	16,277	16,682

Year		2021	2022	2023	2024	2025	2026	2027	2028	2029	2030
**HIV**											
**Survivors without HIV**		22,279	22,170	22,050	21,917	21,772	21,610	21,432	21,234	21,015	20,773
**Survivors with HIV**											
CD4 >500	Diagnosed	2	1	1	0	0	0	0	0	0	0
CD4 >500	Undiagnosed	0	0	0	0	0	0	0	0	0	0
CD4 <500	Diagnosed	7,219	6,784	6,371	5,980	5,610	5,258	4,925	4,608	4,307	4,021
CD4 <500	Undiagnosed	802	754	708	664	623	584	547	512	479	447
AIDS	Diagnosed	1,747	1,644	1,546	1,453	1,365	1,281	1,202	1,127	1,056	988
**Total**		9,770	9,183	8,626	8,097	7,598	7,123	6,674	6,247	5,842	5,456
**Life Years Gained**		12,509	12,987	13,424	13,820	14,174	14,487	14,758	14,987	15,173	15,317
**Quality Adjusted Life Years**											
CD4 >500	Diagnosed	2	1	1	0	0	0	0	0	0	0
CD4 >500	Undiagnosed	0	0	0	0	0	0	0	0	0	0
CD4 <500	Diagnosed	5,486	5,156	4,842	4,545	4,264	3,996	3,743	3,502	3,273	3,056
CD4 <500	Undiagnosed	722	679	637	598	561	526	492	461	431	402
AIDS	Diagnosed	1,083	1,019	959	901	846	794	745	699	655	613
**Total**		7,293	6,855	6,439	6,043	5,671	5,316	4,981	4,662	4,359	4,071
**HIV QALYs Gained**		14,986	15,316	15,612	15,874	16,101	16,294	16,451	16,572	16,656	16,703

Year		2021	2022	2023	2024	2025	2026	2027	2028	2029	2030
**HCV**											
**Survivors without HCV**		18,158	18,087	18,009	17,923	17,828	17,723	17,608	17,481	17,342	17,189
**Survivors with HCV**											
Not Chronic		4,538	4,520	4,501	4,479	4,455	4,428	4,399	4,367	4,332	4,293
Stage 0/1	Diagnosed	4,847	4,741	4,635	4,530	4,425	4,321	4,216	4,112	4,007	3,902
Stage 0/1	Undiagnosed	3,232	3,161	3,090	3,020	2,950	2,881	2,811	2,741	2,671	2,601
Stage 2/3	Diagnosed	3,046	3,079	3,107	3,131	3,149	3,163	3,172	3,176	3,175	3,168
Stage 2/3	Undiagnosed	1,015	1,026	1,036	1,044	1,050	1,054	1,057	1,059	1,058	1,056
Cirrhosis	Diagnosed	1,179	1,243	1,306	1,369	1,432	1,493	1,552	1,611	1,667	1,721
Cirrhosis	Undiagnosed	295	311	327	342	358	373	388	403	417	430
HCC	Diagnosed	14	15	16	17	18	18	19	20	20	21
Liver Failure	Diagnosed	49	52	55	57	60	62	65	67	69	71
**Total**		18,152	18,081	18,002	17,915	17,819	17,713	17,595	17,469	17,327	17,171
**Life Years Gained**		6	6	7	8	9	10	13	12	15	18
**Quality Adjusted Life Years**											
Not Chronic		4,538	4,520	4,501	4,479	4,455	4,428	4,399	4,367	4,332	4,293
Stage 0/1	Diagnosed	3,975	3,888	3,801	3,715	3,629	3,543	3,457	3,372	3,286	3,200
Stage 0/1	Undiagnosed	3,038	2,971	2,905	2,839	2,773	2,708	2,642	2,577	2,511	2,445
Stage 2/3	Diagnosed	2,498	2,525	2,548	2,567	2,582	2,594	2,601	2,604	2,604	2,598
Stage 2/3	Undiagnosed	954	964	974	981	987	991	994	995	995	993
Cirrhosis	Diagnosed	826	870	914	958	1,002	1,046	1,086	1,128	1,168	1,205
Cirrhosis	Undiagnosed	248	261	275	287	301	313	326	339	350	361
HCC	Diagnosed	1	2	2	2	2	2	2	2	2	2
Liver Failure	Diagnosed	16	17	18	18	19	20	21	21	22	23
**Total**		16,093	16,018	15,936	15,847	15,749	15,644	15,528	15,405	15,269	15,119
**HCV QALYs Gained**		2,065	2,069	2,074	2,076	2,079	2,079	2,080	2,076	2,073	2,070
**All Life Years Gained**		12,515	12,994	13,432	13,828	14,183	14,497	14,771	14,999	15,187	15,336
**All QALYs Gained**		17,051	17,385	17,686	17,950	18,180	18,373	18,531	18,648	18,729	18,772

Year		2031	2032	2033	2034	2035	2036	2037	2038	2039	2040
**HIV**											
**Survivors without HIV**		20,510	20,223	19,911	19,571	19,204	18,805	18,371	17,904	17,409	16,874
**Survivors with HIV**											
CD4 >500	Diagnosed	0	0	0	0	0	0	0	0	0	0
CD4 >500	Undiagnosed	0	0	0	0	0	0	0	0	0	0
CD4 <500	Diagnosed	3,750	3,492	3,248	3,016	2,796	2,587	2,388	2,199	2,021	1,851
CD4 <500	Undiagnosed	417	388	361	335	311	287	265	244	225	206
AIDS	Diagnosed	924	864	806	751	699	650	603	558	516	476
**Total**		5,091	4,744	4,415	4,102	3,806	3,524	3,256	3,001	2,762	2,533
**Life Years Gained**		15,419	15,479	15,496	15,469	15,398	15,281	15,115	14,903	14,647	14,341
**Quality Adjusted Life Years**											
CD4 >500	Diagnosed	0	0	0	0	0	0	0	0	0	0
CD4 >500	Undiagnosed	0	0	0	0	0	0	0	0	0	0
CD4 <500	Diagnosed	2,850	2,654	2,468	2,292	2,125	1,966	1,815	1,671	1,536	1,407
CD4 <500	Undiagnosed	375	349	325	302	280	258	239	220	203	185
AIDS	Diagnosed	573	536	500	466	433	403	374	346	320	295
**Total**		3,798	3,539	3,293	3,059	2,838	2,627	2,427	2,237	2,058	1,887
**HIV QALYs Gained**		16,712	16,685	16,618	16,512	16,366	16,177	15,943	15,667	15,350	14,986

Year		2031	2032	2033	2034	2035	2036	2037	2038	2039	2040
**HCV**											
**Survivors without HCV**		17,021	16,836	16,634	16,413	16,171	15,909	15,624	15,315	14,981	14,622
**Survivors with HCV**											
Not Chronic		4,251	4,204	4,153	4,096	4,036	3,970	3,898	3,821	3,736	3,646
Stage 0/1	Diagnosed	3,796	3,690	3,582	3,474	3,364	3,253	3,140	3,026	2,911	2,793
Stage 0/1	Undiagnosed	2,531	2,460	2,388	2,316	2,242	2,168	2,093	2,017	1,940	1,862
Stage 2/3	Diagnosed	3,157	3,140	3,117	3,089	3,055	3,015	2,969	2,917	2,859	2,794
Stage 2/3	Undiagnosed	1,052	1,047	1,039	1,030	1,018	1,005	990	972	953	931
Cirrhosis	Diagnosed	1,772	1,820	1,865	1,906	1,943	1,975	2,002	2,023	2,038	2,047
Cirrhosis	Undiagnosed	443	455	466	477	486	494	500	506	510	512
HCC	Diagnosed	22	22	23	23	24	24	25	25	25	25
Liver Failure	Diagnosed	74	75	77	79	80	81	82	83	84	84
**Total**		17,002	16,816	16,610	16,388	16,144	15,880	15,592	15,282	14,947	14,585
**Life Years Gained**		19	20	24	25	27	29	32	33	34	37
**Quality Adjusted Life Years**											
Not Chronic		4,251	4,204	4,153	4,096	4,036	3,970	3,898	3,821	3,736	3,646
Stage 0/1	Diagnosed	3,113	3,026	2,937	2,849	2,758	2,667	2,575	2,481	2,387	2,290
Stage 0/1	Undiagnosed	2,379	2,312	2,245	2,177	2,107	2,038	1,967	1,896	1,824	1,750
Stage 2/3	Diagnosed	2,589	2,575	2,556	2,533	2,505	2,472	2,435	2,392	2,344	2,291
Stage 2/3	Undiagnosed	989	984	977	968	957	945	931	914	896	875
Cirrhosis	Diagnosed	1,240	1,275	1,306	1,335	1,361	1,384	1,402	1,417	1,427	1,434
Cirrhosis	Undiagnosed	372	382	391	401	408	415	420	425	428	430
HCC	Diagnosed	2	2	2	2	2	2	3	3	3	3
Liver Failure	Diagnosed	24	24	25	25	26	26	26	27	27	27
**Total**		14,959	14,785	14,592	14,386	14,161	13,919	13,656	13,375	13,072	12,746
**HCV QALYs Gained**		2,062	2,052	2,042	2,027	2,010	1,989	1,967	1,940	1,909	1,875
**All Life Years Gained**		15,438	15,500	15,520	15,494	15,425	15,309	15,146	14,936	14,681	14,378
**All QALYs Gained**		18,774	18,736	18,660	18,539	18,376	18,166	17,911	17,608	17,260	16,862

Year		2041	2042	2043	2044	2045	2046	2047	2048	2049	2050
**HIV**											
**Survivors without HIV**		16,302	15,697	15,053	14,376	13,662	12,916	12,144	11,347	10,526	9,689
**Survivors with HIV**											
CD4 >500	Diagnosed	0	0	0	0	0	0	0	0	0	0
CD4 >500	Undiagnosed	0	0	0	0	0	0	0	0	0	0
CD4 <500	Diagnosed	1,690	1,539	1,395	1,260	1,132	1,013	901	796	699	609
CD4 <500	Undiagnosed	188	171	155	140	126	113	100	88	78	68
AIDS	Diagnosed	438	402	368	336	305	276	249	224	200	178
**Total**		2,316	2,112	1,918	1,736	1,563	1,402	1,250	1,108	977	855
**Life Years Gained**		13,986	13,585	13,135	12,640	12,099	11,514	10,894	10,239	9,549	8,834
**Quality Adjusted Life Years**											
CD4 >500	Diagnosed	0	0	0	0	0	0	0	0	0	0
CD4 >500	Undiagnosed	0	0	0	0	0	0	0	0	0	0
CD4 <500	Diagnosed	1,284	1,170	1,060	958	860	770	685	605	531	463
CD4 <500	Undiagnosed	169	154	140	126	113	102	90	79	70	61
AIDS	Diagnosed	272	249	228	208	189	171	154	139	124	110
**Total**		1,725	1,573	1,428	1,292	1,163	1,043	929	823	725	634
**HIV QALYs Gained**		14,577	14,124	13,626	13,084	12,499	11,873	11,215	10,524	9,801	9,054

Year		2041	2042	2043	2044	2045	2046	2047	2048	2049	2050
**HCV**											
**Survivors without HCV**		14,235	13,820	13,377	12,906	12,405	11,876	11,321	10,740	10,135	9,511
**Survivors with HCV**											
Not Chronic		3,549	3,446	3,334	3,216	3,092	2,959	2,821	2,677	2,526	2,369
Stage 0/1	Diagnosed	2,674	2,554	2,432	2,308	2,183	2,057	1,930	1,803	1,675	1,548
Stage 0/1	Undiagnosed	1,783	1,703	1,621	1,539	1,456	1,371	1,287	1,202	1,117	1,032
Stage 2/3	Diagnosed	2,723	2,645	2,561	2,471	2,375	2,272	2,164	2,051	1,933	1,811
Stage 2/3	Undiagnosed	908	882	854	824	792	757	721	684	644	604
Cirrhosis	Diagnosed	2,048	2,042	2,029	2,007	1,976	1,937	1,889	1,832	1,766	1,691
Cirrhosis	Undiagnosed	512	511	507	502	494	484	472	458	441	423
HCC	Diagnosed	25	25	25	25	24	24	23	22	22	21
Liver Failure	Diagnosed	84	83	83	82	80	78	76	74	71	68
**Total**		14,197	13,783	13,338	12,867	12,368	11,837	11,284	10,707	10,102	9,478
**Life Years Gained**		38	37	39	39	37	39	37	33	33	33
**Quality Adjusted Life Years**											
Not Chronic		3,549	3,446	3,334	3,216	3,092	2,959	2,821	2,677	2,526	2,369
Stage 0/1	Diagnosed	2,193	2,094	1,994	1,893	1,790	1,687	1,583	1,478	1,374	1,269
Stage 0/1	Undiagnosed	1,676	1,601	1,524	1,447	1,369	1,289	1,210	1,130	1,050	970
Stage 2/3	Diagnosed	2,233	2,169	2,100	2,026	1,948	1,863	1,774	1,682	1,585	1,485
Stage 2/3	Undiagnosed	854	829	803	775	744	712	678	643	605	568
Cirrhosis	Diagnosed	1,435	1,431	1,422	1,406	1,385	1,358	1,325	1,285	1,238	1,185
Cirrhosis	Undiagnosed	430	429	426	422	415	407	396	385	370	355
HCC	Diagnosed	3	3	3	3	2	2	2	2	2	2
Liver Failure	Diagnosed	27	27	27	26	26	25	24	24	23	22
**Total**		12,398	12,029	11,631	11,212	10,771	10,301	9,813	9,305	8,773	8,226
**HCV QALYs Gained**		1,837	1,791	1,745	1,693	1,634	1,575	1,508	1,434	1,362	1,285
**All Life Years Gained**		14,024	13,622	13,174	12,679	12,136	11,553	10,931	10,271	9,583	8,866
**All QALYs Gained**		16,414	15,915	15,371	14,777	14,133	13,449	12,723	11,958	11,163	10,339

Year		2051	2052	2053	2054	2055	2056	2057	2058	2059	2060
**HIV**											
**Survivors without HIV**		8,845	8,001	7,164	6,344	5,553	4,800	4,090	3,433	2,838	2,305
**Survivors with HIV**											
CD4 >500	Diagnosed	0	0	0	0	0	0	0	0	0	0
CD4 >500	Undiagnosed	0	0	0	0	0	0	0	0	0	0
CD4 <500	Diagnosed	526	451	382	320	265	217	175	139	109	84
CD4 <500	Undiagnosed	58	50	42	36	29	24	19	15	12	9
AIDS	Diagnosed	157	137	119	103	88	75	63	52	42	34
**Total**		741	638	543	459	382	316	257	206	163	127
**Life Years Gained**		8,104	7,363	6,621	5,885	5,171	4,484	3,833	3,227	2,675	2,178
**Quality Adjusted Life Years**											
CD4 >500	Diagnosed	0	0	0	0	0	0	0	0	0	0
CD4 >500	Undiagnosed	0	0	0	0	0	0	0	0	0	0
CD4 <500	Diagnosed	400	343	290	243	201	165	133	106	83	64
CD4 <500	Undiagnosed	52	45	38	32	26	22	17	14	11	8
AIDS	Diagnosed	97	85	74	64	55	47	39	32	26	21
**Total**		549	473	402	339	282	233	189	151	120	93
**HIV QALYs Gained**		8,295	7,528	6,762	6,005	5,271	4,567	3,901	3,282	2,718	2,212

Year		2051	2052	2053	2054	2055	2056	2057	2058	2059	2060
**HCV**		8,869	8,216	7,556	6,893	6,235	5,588	4,960	4,358	3,787	3,253
**Survivors without HCV**											
**Survivors with HCV**		2,209	2,047	1,883	1,717	1,552	1,392	1,235	1,085	943	810
Not Chronic		1,422	1,297	1,176	1,057	942	832	728	631	541	458
Stage 0/1	Diagnosed	948	865	784	705	628	555	486	421	360	305
Stage 0/1	Undiagnosed	1,686	1,559	1,431	1,302	1,175	1,051	930	815	706	605
Stage 2/3	Diagnosed	562	520	477	434	392	350	310	272	235	202
Stage 2/3	Undiagnosed	1,609	1,519	1,423	1,322	1,217	1,110	1,001	894	789	689
Cirrhosis	Diagnosed	402	380	356	331	304	277	250	224	197	172
Cirrhosis	Undiagnosed	20	19	17	16	15	14	12	11	10	8
HCC	Diagnosed	64	60	56	52	48	43	39	34	30	26
Liver Failure	Diagnosed	8,838	8,187	7,530	6,868	6,210	5,567	4,940	4,342	3,771	3,241
**Total**		31	29	26	25	25	21	20	16	16	12
**Life Years Gained**											
**Quality Adjusted Life Years**		2,209	2,047	1,883	1,717	1,552	1,392	1,235	1,085	943	810
Not Chronic		1,166	1,064	964	867	772	682	597	517	444	376
Stage 0/1	Diagnosed	891	813	737	663	590	522	457	396	338	287
Stage 0/1	Undiagnosed	1,383	1,278	1,173	1,068	964	862	763	668	579	496
Stage 2/3	Diagnosed	528	489	448	408	368	329	291	256	221	190
Stage 2/3	Undiagnosed	1,129	1,066	999	928	854	779	703	628	554	485
Cirrhosis	Diagnosed	338	319	299	278	255	233	210	188	165	144
Cirrhosis	Undiagnosed	2	2	2	2	2	1	1	1	1	1
HCC	Diagnosed	20	19	18	17	15	14	12	11	10	8
Liver Failure	Diagnosed	7,666	7,097	6,524	5,946	5,373	4,814	4,269	3,751	3,255	2,797
**Total**		1,203	1,119	1,032	946	862	774	691	608	532	457
**HCV QALYs Gained**											
**All Life Years Gained**		8,134	7,392	6,647	5,910	5,196	4,505	3,853	3,243	2,691	2,190
**All QALYs Gained**		9,498	8,648	7,794	6,952	6,133	5,341	4,592	3,889	3,250	2,669

Year		2061	2062	2063	2064	2065	2066	2067	2068	2069	2070
**HIV**											
**Survivors without HIV**		1,842	1,450	1,120	853	635	454	308	188	90	0
**Survivors with HIV**											
CD4 >500	Diagnosed	0	0	0	0	0	0	0	0	0	0
CD4 >500	Undiagnosed	0	0	0	0	0	0	0	0	0	0
CD4 <500	Diagnosed	63	47	35	25	18	12	8	5	2	0
CD4 <500	Undiagnosed	7	5	4	3	2	1	1	1	0	0
AIDS	Diagnosed	27	21	16	12	8	6	4	2	1	0
**Total**		97	73	55	40	28	19	13	8	3	0
**Life Years Gained**		1,745	1,377	1,065	813	607	435	295	180	87	0
**Quality Adjusted Life Years**											
CD4 >500	Diagnosed	0	0	0	0	0	0	0	0	0	0
CD4 >500	Undiagnosed	0	0	0	0	0	0	0	0	0	0
CD4 <500	Diagnosed	48	36	27	19	14	9	6	4	2	0
CD4 <500	Undiagnosed	6	5	4	3	2	1	1	1	0	0
AIDS	Diagnosed	17	13	10	7	5	4	2	1	1	0
**Total**		71	53	40	29	20	14	9	6	2	0
**HIV QALYs Gained**		1,771	1,397	1,080	824	615	440	298	182	87	0

Year		2061	2062	2063	2064	2065	2066	2067	2068	2069	2070
**HCV**											
**Survivors without HCV**		2,760	2,311	1,899	1,534	1,219	952	731	550	405	290
**Survivors with HCV**											
Not Chronic		687	576	473	382	304	237	182	137	101	72
Stage 0/1	Diagnosed	383	316	256	204	160	124	94	70	51	36
Stage 0/1	Undiagnosed	255	211	171	136	107	82	62	46	34	24
Stage 2/3	Diagnosed	511	427	349	281	223	173	133	99	73	52
Stage 2/3	Undiagnosed	170	142	116	94	74	58	44	33	24	17
Cirrhosis	Diagnosed	593	504	420	344	277	219	170	130	97	70
Cirrhosis	Undiagnosed	148	126	105	86	69	55	43	32	24	17
HCC	Diagnosed	7	6	5	4	3	3	2	2	1	1
Liver Failure	Diagnosed	22	19	15	12	10	8	6	4	3	2
**Total**		2,747	2,302	1,890	1,527	1,214	948	728	547	404	288
**Life Years Gained**		13	9	9	7	5	4	3	3	1	2
**Quality Adjusted Life Years**											
Not Chronic		687	576	473	382	304	237	182	137	101	72
Stage 0/1	Diagnosed	314	259	210	167	131	102	77	57	42	30
Stage 0/1	Undiagnosed	240	198	161	128	101	77	58	43	32	23
Stage 2/3	Diagnosed	419	350	286	230	183	142	109	81	60	43
Stage 2/3	Undiagnosed	160	133	109	88	70	55	41	31	23	16
Cirrhosis	Diagnosed	417	354	296	243	195	154	120	92	69	50
Cirrhosis	Undiagnosed	124	106	88	72	58	46	36	27	20	14
HCC	Diagnosed	1	1	1	0	0	0	0	0	0	0
Liver Failure	Diagnosed	7	6	5	4	3	3	2	1	1	1
**Total**		2,369	1,984	1,628	1,315	1,045	815	626	470	347	247
**HCV QALYs Gained**		391	327	271	219	174	137	105	80	58	42
**All Life Years Gained**		1,758	1,386	1,074	820	612	439	298	183	88	2
**All QALYs Gained**		2,162	1,724	1,351	1,043	789	577	404	263	145	42

Year		2071	2072	2073	2074	2075	Total
**HIV**							
**Survivors without HIV**							1,162,533
**Survivors with HIV**							
CD4 >500	Diagnosed						96,422
CD4 >500	Undiagnosed						24,103
CD4 <500	Diagnosed						344,734
CD4 <500	Undiagnosed						38,302
AIDS	Diagnosed						71,410
**Total**							574,971
**Life Years Gained**							587,562
**Quality Adjusted Life Years**							
CD4 >500	Diagnosed						83,887
CD4 >500	Undiagnosed						22,657
CD4 <500	Diagnosed						261,998
CD4 <500	Undiagnosed						34,472
AIDS	Diagnosed						44,274
**Total**		0	0	0	0	0	447,288
**HIV QALYs Gained**			0	0	0	0	715,245

Year		2071	2072	2073	2074	2075	Total
**HCV**							
**Survivors without HCV**		198	127	73	32	0	1,036,257
**Survivors with HCV**							
Not Chronic		49	31	19	8	0	258,775
Stage 0/1	Diagnosed	24	15	9	4	0	285,367
Stage 0/1	Undiagnosed	16	10	6	3	0	190,245
Stage 2/3	Diagnosed	35	23	13	6	0	150,916
Stage 2/3	Undiagnosed	12	8	4	2	0	50,307
Cirrhosis	Diagnosed	48	31	18	8	0	79,585
Cirrhosis	Undiagnosed	12	8	5	2	0	19,898
HCC	Diagnosed	1	0	0	0	0	975
Liver Failure	Diagnosed	1	1	0	0	0	3,248
**Total**		196	126	74	33	0	1,035,093
**Life Years Gained**		2	1	-1	-1	0	1,164
**Quality Adjusted Life Years**							
Not Chronic		49	31	19	8	0	
Stage 0/1	Diagnosed	20	12	7	3	0	234,001
Stage 0/1	Undiagnosed	15	9	6	3	0	178,830
Stage 2/3	Diagnosed	29	19	11	5	0	123,751
Stage 2/3	Undiagnosed	11	8	4	2	0	47,289
Cirrhosis	Diagnosed	34	22	13	6	0	55,768
Cirrhosis	Undiagnosed	10	7	4	2	0	16,714
HCC	Diagnosed	0	0	0	0	0	98
Liver Failure	Diagnosed	0	0	0	0	0	1,039
**Total**		168	108	64	29	0	916,265
**HCV QALYs Gained**		30	19	9	3	0	119,992
**All Life Years Gained**		2	1	-1	-1	0	588,726
**All QALYs Gained**		30	19	9	3	0	835,237

APPENDIX F

REFERENCES

REFERENCES TO STUDIES USED IN THE ECOLOGICAL ANALYSIS

Aitken C, Brough R, Crofts N. *Injecting drug use and blood-borne viruses: a comparison of rural and urban Victoria, 1990-95*. Drug & Alcohol Review 1999; 18(1):47-52.

Alfonso GR, Hurtado N, I, Espacio CA, Santos RG, Tomas DS. *Risk behaviours and seroprevalence to HIV, HBV and HCV in patients of the AIDS information and prevention center in Valencia, Spain*. Gaceta Sanitaria 1999; 13(1):16-21.

Australian National Study of AIDS and Injecting Drug Use Study, (ANAIDUS) (1991). *Neither a borrower nor a lender be: First report of the Australian National AIDS and injecting drug use study, 1989 data collection*. National Centre in HIV Social Research, University of New South Wales, Sydney.

Azevedo-Neto RS, RC Bueno, F Mesquita, et al. *HIV Seroprevalence in IDUs from Santos: General Trends and Gender Analysis*. XI International Conference on AIDS, Vancouver, 1996; 7/7-14, Abstract Tu.C.2493.

Baozhang T, Kaining Z, Jinxing K, Ruchang X, Ming L, Caixia Z, Li T. *Infection with human immunodeficiency virus and hepatitis viruses in Chinese drug addicts*. Epidemiology & Infection 1997; 119(3):343-347.

Battjes RJ, Pickens RW, Brown LS (1995). *HIV infection and AIDS risk behaviours among injecting drug users entering Methadone Treatment: An update*. J Acqu Immune Def Syndr & Human Retro, 10: 90-96.

Bell J, Batey RG, Farrell GC, Crewe EB, Cunningham AL, Byth K. *Hepatitis C virus in intravenous drug users.* Medical Journal of Australia 1990; 153(5):274-276.

Blacker P, Tindall B, Wodak A, Cooper D (1986). *Exposure of intravenous drug users to AIDS retrovirus, Sydney, 1985*. Aust & NZ J of Med, 16: 686-690.

Bluthenthal RN, Kral AH, Lorvick JJ. *Risk behaviours among clients of tolerated and underground syringe exchange programs in the San Francisco Bay area*. 1996

Bolumar F, Hernandez-Aguado I, Ferrer L, Ruiz I, Avino MJ, Rebagliato M. *Prevalence of antibiotics to hepatitis C in a population of intravenous drug users in Valencia, Spain, 1990-1992*. International Journal of Epidemiology 1996; 25(1):204-209.

Brajachand-Singh N, Ibotomba-Singh Y. *Absence of Serological Evidence of HIV in Manipur, a Border State of India.* Virus Information Exchange Newsletter. 1987; 4(3): 81.

Broers B, Junet C, Bourquin M, Deglon JJ, Perrin L, Hirschel B. *Prevalence and incidence rate of HIV, hepatitis B and C among drug users on methadone maintenance treatment in Geneva between 1988 and 1995*. AIDS 1998; 12(15):2059-2066.

Brunton C, Kemp R, Raynel P, Harte D, Baker M. *Cumulative incidence of hepatitis C seroconversion in a cohort of seronegative injecting drug users*. New Zealand Medical Journal 2000; 113(1106):98-101.

Byrne A, Wodak A. *Census of patients receiving methadone treatment in a general practice*. Addiction Research 1996; 3(4):341-349.

Byrne A (2000). *Nine year follow-up of 86 consecutive patients treated with methadone in general practice, Sydney, Australia*. Drug & Alcohol Rev, 19: 153-158.

Cahn P, Perez H, Casiro A, et al. *Analysis of Spontaneous Attendance at an AIDS Consulting Centre in the City of Buenos Aires*. Medicina (Buenos Aires) 1988; 48: 125-131.

Cao K, Mizokami M, Orito E, Ding X, Ge XM, Huang GY, Ueda R. *TT virus infection among IVDUs in south western China*. Scandinavian Journal of Infectious Diseases 1999; 31(1):21-25.

Carruthers S, Loxley W. *Hepatitis C and young drug users: are they about to join the epidemic?* Australian Journal of Public Health 1995; 19(4):421-424

Carter H, Robinson G, Hanlon C, Hailwood C, Massarotto A. *Prevalence of hepatitis B and C infection in a methadone clinic population: implications for hepatitis B vaccination*. New Zealand Medical Journal 2001; 114(1136):324-326.

Celentano DD, Jittiwutikorn J, Hodge MJ, et al. *Epidemiology of HIV-1 Infection in Opiate Users in Northern Thailand*. Journal of Acquired Immune Deficiency Syndromes and Human Retrovirology. 1997; 17(1): 73-78.

Chamot E, de Saussure P, Hirschel B, Deglon JJ, Perrin LH. *Incidence of hepatitis C, hepatitis B and HIV infections among drug users in a methadone-maintenance programme*. AIDS 1992; 6(4):430-431.

Chamot E, Hirschel B, Wintsch J, Robert CF, Gabriel V, Deglon JJ, Yerly S, Perrin L. *Loss of antibodies against hepatitis C virus in HIV-seropositive intravenous drug users*. AIDS 1990; 4(12):1275-1277.

Chang CJ, Ko YC, Liu HW. *Serum alanine aminotransferase levels in relation to hepatitis B and C virus infections among drug abusers in an area hyperendemic for hepatitis B.* Digestive Diseases & Sciences 2000; 45(10):1949-1952.

Chen DS, Kuo GC, Sung JL, Lai MY, Sheu JC, Chen PJ, Yang PM, Hsu HM, Chang MH, Chen CJ. *Hepatitis C virus infection in an area hyperendemic for hepatitis B and chronic liver disease: the Taiwan experience*. Journal of Infectious Diseases 1990; 162(4):817-822.

Chetwynd J, Brunton C, Blank M, Plumridge E, Baldwin D. *Hepatitis c seroprevalence amongst injecting drug users attending a methadone programme*. New Zealand Medical Journal 1995; 108(1007):364-366.

Choopanya K, Des Jarlais DC, Vanichseni S, et al. *Risk Factors for HIV Seroprevalence among IDUs in Bangkok, 1989 vs. 1993*. Tenth International Conference on AIDS, Yokohama, Japan. 1994; 8/7-12, Abstract P.C.0125.

Coppola RC, Masia G, di Martino ML, Carboni G, Muggianu E, Piro R, Manconi PE. *Sexual behaviour and multiple infections in drug abusers*. European Journal of Epidemiology 1996; 12(5):429-435.

Corona R, Prignano G, Mele A, Gentili G, Caprilli F, Franco E, Ferrigno L, Giglio A, Titti F, Bruno C. *Heterosexual and homosexual transmission of hepatitis C virus: relation with hepatitis B virus and human immunodeficiency virus type 1*. Epidemiology & Infection 1991; 107(3):667-672.

Crofts N, Aitken CK. *Incidence of blood borne virus infection and risk behaviours in a cohort of injecting drug users in Victoria, 1990-1995*. Medical Journal of Australia 1997; 167(1):17-20.

Crofts N. *Surveillance for HIV Infection and AIDS.* World Health Organization, Global Programme on AIDS, Kuala Lumpur, Malaysia, 7-27 October 1991, mission report. 1992.

Crofts N, Hopper JL, Bowden DS, Breschkin AM, Milner R, Locarnini SA. *Hepatitis C virus infection among a cohort of Victorian injecting drug uses*. Medical Journal of Australia 1993; 159(4): 237-241

Crofts N, Hopper JL, Milner R, Breschkin AM, Bowden DS, Locarnini SA. *Blood-borne virus infections among Australian injecting drug users: implications for spread of HIV.* European Journal of Epidemiology 1994; 10(6):687-694.

Crofts N, Stewart T, Hearne P, Ping XY, Breshkin AM, Locarnini SA. *Spread of blood borne viruses among Australian prison entrants*. BMJ 1995; 310(6975):285-288.

Crofts N, Nigro L, Oman K, Stevenson E, Sherman J. *Methadone maintenance and hepatitis C virus infection among injecting drug users*. Addiction 1997; 92(8):999-1005.

Dan M, Rock M, Bar-Shany S. *Prevalence of Antibodies to Human Immunodeficiency Virus among Intravenous Drug Users in Israel - Association with Travel Aboard*. International Journal of Epidemiology. 1989; 18(1): 239-241.

Dan M. *HIV Infection and Intravenous Drug Abuse: World Perspective and Epidemiology in Israel.* Israel Journal of Medical Sciences. 1993; 29(10) (Suppl.): 11-14.

Darke S, Baker A, Dixon J, Wodak A, Heather N. *Drug use and HIV-risk taking among clients in methadone maintenance treatment*. Drug & Alcohol Dependence, 1992a 29: 263-268.

de Carvalho HB, Mesquita F, Massad E, Bueno RC, Lopes GT, Ruiz MA, Burattini MN. *HIV and infections of similar transmission patterns in a drug injectors community of Santos, Brazil.* Journal of Acquired Immune Deficiency Syndromes 1996; 12(1):84-92.

Delgado-Iribarren A, Calvo M, Perez A, del Alamo M, Cercenado S*. Intravenous drug users serologic control: what may be prevented?*. [Spanish]. Enfermedades Infecciosas y Microbiologia Clinica 2000; 18(1):2-5.

Denham I & Hayes P. *Human immunodeficiency virus antibody testing at the Melbourne Sexually Transmitted Diseases Centre 1984-1989. Six years and 17,382 tests.* Venerology, 1990, 3: 4-11.

Denis B, Dedobbeleer M, Collet T, Petit J, Jamoulle M, Hayani A, Brenard R. *High prevalence of hepatitis C virus infection in Belgian intravenous drug users and potential role of the "cotton-filter" in transmission: the GEMT Study*. Acta Gastroenterologica Belgica 2000; 63(2):147-153.

Des Jarlais DC., Subhachaturas W, Vanichseni S, et al. *Sexual Risk Behaviors of Injecting Drug Users in Bangkok, Thailand.* 12th World AIDS Conference, Geneva. 1998; 6/28-7/3, Abstract 23154.

Diamantis I, Bassetti S, Erb P, Ladewig D, Gyr K, Battegay M. *High prevalence and coinfection rate of hepatitis G and C infections in intravenous drug addicts*. Journal of Hepatology 1997; 26(4):794-797.

Diaz T, Des J, Vlahov D, Perlis TE, Edwards V, Friedman SR, Rockwell R, Hoover D, Williams IT, Monterroso ER. *Factors associated with prevalent hepatitis C: differences among young adult injection drug users in lower and upper Manhattan, New York City*. American Journal of Public Health 2001; 91(1):23-30.

Dimitrakopoulos A, Takou A, Haida A, Molangeli S, Gialeraki A, Kordossis T. *The prevalence of hepatitis B and C in HIV-positive Greek patients: relationship to survival of deceased AIDS patients.* Journal of Infection 2000; 40(2):127-131.

Donovan B, Finlayson RJ, Mutimer K. *HIV infection in sexually transmissible disease practice in Sydney: the effects of legislation, public education and changing clinical spectrum*. Int J of STD & AIDS, 1990, 1: 21-27.

Donahue JG, Nelson KE, Munoz A, Vlahov D, Rennie LL, Taylor EL, Saah AJ, Cohn S, Odaka NJ, Farzadegan H. *Antibody to hepatitis C virus among cardiac surgery patients, homosexual men, and intravenous drug users in Baltimore, Maryland*. American Journal of Epidemiology 1991; 134(10):1206-1211.

Dourado I, Andrade T, Montes JC, et al. *Human Retrovirus in a Brazilian City with a Population Predominantly of African Origin: Evidences for High Prevalence.* XI International Conference on AIDS, Vancouver. 1996; 7/7-14, Poster Pub.C.1132.

Duburcq A, Charpak Y, Blin P, Madec L. *Two years follow-up of heroin users treated by GPs with high dosage buprenorphine: the SPESUB study (pharmaco-epidemiological follow-up of high dosage buprenorphine in general practice)* [French]. Revue d Epidemiologie et de Sante Publique 2000; 48(4):363-373.

Dwyer D, Bell J, Batey R, et al. *Low prevalence of human immunodeficiency virus infection in methadone program attenders and pregnant intravenous drug users in the western metropolitan region of Sydney.* Aus & NZ J of Med, 1989, 19: 407-408.

Edeh J, Spalding P. *Screening for HIV, HBV and HCV markers among drug users in treatment in rural south-east England*. Journal of Public Health Medicine 2000; 22(4):531-539.

Eicher AD, Crofts N, Benjamin S, Deutschmann P, Rodger AJ. *A certain fate: spread of HIV among young injecting drug users in Manipur, North-East India*. AIDS Care 2000; 12(4):497-504.

European Centre for the Epidemiological Monitoring of AIDS (ECDMA). *HIV/AIDS surveillance in Europe. Mid Year report 2001*. 2001 No. 65.

European Monitoring Centre for Drugs and Drug Addiction (EMCDDA) *Annual report on the state of the drugs problem in the European Union, 2000*. Office for Official Publications of the European Communities, Luxembourg.

Fairley CK, Leslie DE, Nicholson S, Gust ID. *Epidemiology and hepatitis C virus in Victoria*. Medical Journal of Australia 1990; 153(5):271-273.

Ferreira A, Arevalo E, Fernandes NEL, et al. *HIV Infection among Users and Non Users of Injected Drugs.* VIII International Conference on AIDS, Amsterdam, 1992; 7/19-24, Abstract PuC 8072.

Fingerhood MI, Jasinski DR, Sullivan JT. *Prevalence of Hepatitis C in a chemically dependent population.* Archives of Internal Medicine 1993; 153(17):2025-2030.

Fisher DG, Fenaughty AM, Paschane AA, Paschane DM, Cagle HH, Orr SM. *Hepatitis c virus infection among Alaskan drug users*. American Journal of Public Health 1997; 87(10):1722-1724.

Francisci D, Baldelli F, Papili R, Stagni G, Pauluzzi S. *Prevalence of HBV, HDV and HCV hepatitis markers in HIV-positive patients*. European Journal of Epidemiology 1995; 11(2):123-126.

Friedman SR, Des Jarlais DC. *HIV among drug injectors: the epidemic and the response*. AIDS Care 1991,3 (3) 239-250

Friedman SR, Jose B, Deren S, Des Jarlais DC, *Neagigus for the National AIDS Research Consortium. Risk factors for human immunodeficiency virus seroconversion among out-of-treatment drug injectors in high and low seroprevalence cities.* 1995, 142,(8):864-874.

Frischer M, Bloor M, Green S, Goldberg D, Covell R, McKeganey, Taylor. *Reduction in needle sharing among community wide samples of injecting drug users.* International Journal of STD & AIDS 1992; 3: 288-290.

Freeman AJ, Zekry A, Whybin LR, Harvey CE, van Beek IA, de Kantzow SL, Rawlinson WD, Boughton CR, Robertson PW, Marinos G, Lloyd AR. *Hepatitis C prevalence among Australian injecting drug users in the 1970s and profiles of virus genotypes in the 1970s and 1990s*. Medical Journal of Australia 2000; 172(12):588-591.

Fuglsang T, Fouchard JR, Ege PP. *Prevalence of HIV and hepatitis B and C among drug addicts in the city of Copenhagen*. [Danish]. Ugeskrift for Laeger 2000; 162(27):3860-3864.

Galeazzi B, Tufano A, Barbierato E, Bortolotti F. *Hepatitis C virus infection in Italian intravenous drug users: epidemiological and clinical aspects.* Liver 1995; 15(4):209-212.

Garfein RS, Vlahov D, Galai N, Doherty MC, Nelson KE. *Viral infections in short-term injection drug users: the prevalence of hepatitis c hepatitis B, human immunodeficiency and human T-lymphotropic viruses*. American Journal of Puclic Health 1996; 86 (5): 655-661.

Garfein RS, Doherty MC, Monterroso ER, Thomas DL, Nelson KE, Vlahov D. *Prevalence and incidence of hepatitis C virus infection among young adult injection drug users.* Journal of Acquired Immune Deficiency Syndromes 1998; 18 Suppl 1:S11-S19.

Garner JJ, Gaughwin M, Dodding J, Wilson K. *Prevalence of hepatitis C infection in pregnant women in South Australia.* Medical Journal of Australia 1997; 167(9):470-472.

Gaughwin M, Dodding J, Ali R. *Hepatitis C and the history of injecting drug use in South Australia*. [letter; comment]. Medical Journal of Australia 1994; 161(4):286.

Gaughwin MD & Ali R. *HIV infection among injecting drug users in the South Australian methadone program.* Med J Aust, 1995, 162: 242-244.

Gianna MC, Chequer P, Castilho E, et al. *Profile of IVDU in an AIDS Reference Center, Sao Paulo, Brazil*, *1988*. VI International Conference on AIDS, San Francisco, 1990; 6/20-24, Poster Th.C.713.

Girardi E, Zaccarelli M, Tossini G, Puro V, Narciso P, Visco G. *Hepatitis C virus infection in intravenous drug users: prevalence and risk factors. Scandinavian Journal of Infectious Diseases 1990*; 22(6):751-752.

Giuliani M, Caprilli F, Gentili G, Maini A, Lepri AC, Prignano G, Palamara G, Giglio A, Crescimbeni E, Rezza G. *Incidence and determinants of hepatitis c virus infection among individuals at risk of sexually transmitted diseases attending a human immunodeficiency virus type 1 testing program*. Sexually Transmitted Diseases 1997; 24(9):533-537.

Guimaraes ML, Bastos FI, Telles PR, Galvao-Casto B, Diaz RS, Bongertz V, Morgado MG. *Retrovirus infections in a sample of injecting drug users in Rio de Janeiro City, Brazil: prevalence of HIV-1 subtypes, and co-infection with HTLV-I/II*. Journal of Clinical Virology, 2001; 21: 143-151.

Goldberg D, Burns S, Taylor A, Cameron S, Hargreaves D, Hutchinson S. *Trends in HCV prevalence among injecting drug users in Glasgow and Edinburgh during the era of needle/syringe exchange*. Scandinavian Journal of Infectious Diseases 2001a; 33(6):457-461.

Goldberg D, Cameron S, McMenamin J. *Hepatitis C virus antibody prevalence among injecting drug users in Glasgow has fallen but remains high*. Communicable Disease & Public Health 1998; 1(2):95-97.

Goldberg D, Cameron S, Sharp G, Burns S, Scott G, Molyneaux P, Scoular A, Downie A, Taylor A. *Hepatitis C virus among genitourinary clinic attenders in Scotland: unlinked anonymous testing*. International Journal of STD & AIDS 2001b; 12(1):17-21.

Goldberg D, McIntyre PG, Smith R, Appleyard K, Dunlop J, Taylor A, Hutchinson S. *Hepatitis C virus among high and low risk pregnant women in Dundee: unlinked anonymous testing.* British Journal of Obstetrics & Gynaecology 2001c; 108(4):365-370.

Gore SM, Brettle RP, Burns SM, Lewis SC. *Pilot study to estimate survivors to 1995 of 1983-1984 prevalent hepatitis c infections in lothian patients who tested positive or negative for hepatitis b surface antigen in 1983-1984*. Journal of Infection 1998; 37(2):159-165.

Guadagnino V, Zimatore G, Rocca A, Montesano F, Masciari R, Caroleo B, Izzi A, Morabito D, Naso E, Scicchitano R. *Anti-hepatitis C antibody prevalence among intravenous drug addicts in the Catanzaro area.* Archives of Virology - Supplementum 1992; 4:335-336.

Hagan H, Jarlais DC, Friedman SR, Purchase D, Alter MJ. *Reduced risk of hepatitis B and hepatitis C among injection drug users in the Tacoma syringe exchange program.* American Journal of Public Health 1995; 85(11):1531-1537.

Hagan H, McGough JP, Thiede H, Weiss NS, Hopkins S, Alexander ER. *Syringe exchange and risk of infection with hepatitis B and C viruses.* American Journal of Epidemiology 1999; 149(3):203-213.

Hagan H, Thiede H, Weiss NS, Hopkins SG, Duchin JS, Alexander ER. *Sharing of drug preparation equipment as a risk factor for hepatitis C*. American Journal of Public Health 2001; 91(1):42-46.

Hahn RA, Onorato IM, Jones TS, Dougherty J. *Prevalence of HIV infection among intravenous drug users in the United States.* JAMA, 1989; Vol 261, No. 18: 2677-2684.

Hahn JA, Page-Shafer K, Lum PJ, Ochoa K, Moss AR. *Hepatitis C virus infection and needle exchange use among young injection drug users in San Francisco*. Hepatology 2001; 34(1): 180-187.

Hamers FF, Batter V, Downs AM, Alix J, Cazein F, Brunet JB. *The HIV epidemic associated with injecting drug use in Europe: geographic and time trends*. AIDS 1997, 11:1365-1374.

Harsch HH, Pankiewicz J, Bloom AS, Rainey C, Cho JK, Sperry L, Stein EA. *Hepatitis C virus infection in cocaine users - a silent epidemic*. Community Mental Health Journal 2000; 36(3):225-233.

Hart GJ, Woodward N, Johnson AM, Tighe J, Parry JV, Adler MW. *Prevalence of HIV, hepatitis B and associated risk behaviors in clients of a needle-exchange in central London*. AIDS 1992, 5:543-547.

Health Canada. *Inventory of HIV incidence and prevalence studies in Canada*. April 2000.

Htoon MT, Lwin HH, San KO, et al. *HIV/AIDS in Myanmar.* AIDS. 1994; 8(suppl. 2): S105-S109.

Hwang LY, Ross MW, Zack C, Bull L, Rickman K, Holleman M. *Prevalence of sexually transmitted infections and associated risk factors among populations of drug abusers*. Clinical Infectious Diseases 2000; 31(4):920-926.

Ichimura H, Kurimura O, Tamura I, Tsukue I, Tsuchie H, Kurimura T. *Prevalence of blood-borne viruses among intravenous drug users and alcoholics in Hiroshima, Japan*. International Journal of STD & AIDS 1995; 6(6):441-443.

Inciardi JA, Surratt H, Telles P, et al. *Risks for HIV-1 Infection and Seropositivity Rates among Cocaine Users in Rio de Janeiro, Brazil*. XI International Conference on AIDS, Vancouver. 1996; 7/7-14, Session Th.C.425.

Inciardi J, McBride D, Surratt H, et al. *Reducing HIV Risk among Cocaine Users in Rio de Janeiro, Brazil*. 12th World AIDS Conference, Geneva. 1998; 6/28 - 7/3, Poster 33407.

Iqbal N. *Substance dependence - A hospital based survey.* Saudi Medical Journal 2000; 21(1):51-57.

Iguchi MY, Bux DA, Lidz V, Kushner H, French JF, Platt JJ. *Interpreting HIV seroprevalence data from a street-based outreach program.* Journal of Acquired Immune Deficiency Syndromes 1994, 7:491-499.

Ismail R. *HIV/AIDS in Malaysia*. AIDS. 1998; 12 (Suppl B):S33-S41.

Jiang JJ, Dubois F, Driss F, Carnot F, Thepot V, Pol S, Berthelot P, Brechot C, Nalpas B. *Clinical impact of drug addiction in alcoholics.* Alcohol & Alcoholism 1995; 30(1):55-60.

Johnson Z, O'Connor M, Pomeroy L, Johnson H, Barry J, Scully M, Fitzpatrick E. *Prevalence of HIV and associated risk behavior in attendees at a Dublin needle exchange*. Addiction 1994; 89 (5): 603-607.

Kelen GD, Green GB, Purcell RH, Chan DW, Qaqish BF, Sivertson KT, Quinn TC. *Hepatitis B and hepatitis C in emergency department patients*. New England Journal of Medicine 1992; 326(21):1399-1404.

Kemp R, Miller J, Lungley S, Baker M. *Injecting behaviours and prevalence of hepatitis B, C and D markers in New Zealand injecting drug user populations*. New Zealand Medical Journal 1998; 111(1060):50-53.

Kitayaporn D, Vanichseni S, Mastro TD, et al. Infection with HIV-1 *Subtypes B and E in Injecting Drug Users Screened for Enrollment into a Prospective Cohort in Bangkok.* Journal of Acquired Immune Deficiency Syndromes and Human Retrovirology. 1998; 19(3): 289-295.

Krook A, Albert J, Andersson S, Biberfeld G, Blomberg J, Eklund I, Engstrom A, Julander I, Kall K, Martin C, Stendahl P, Struve J, Sonnerborg A. *Prevalence and risk factors for HTLV-II infection in 913 injecting drug users in Stockholm, 1994.* Journal of Acquired Immune Deficiency Syndromes 1997; 15(5):381-386.

Lal S. *AIDS - A Priority Health Problem in India*. Swasth Hind. 1991; 35: 277.

Lamden KH, Kennedy N, Beeching NJ, Lowe D, Morrison CL, Mallinson H, Mutton KJ, Syed Q. *Hepatitis B and hepatitis C virus infections: Risk factors among drug users in Northwest England*. Journal of Infection 1998; 37(3):260-269.

Laskus T, Radkowski M, Werezynska T, Horban A, Lupa E, Cianciara J, Slusarczyk J. *Occurrence of antibodies against hepatitis C virus (HCV) among drug addicts*. [Polish]. Polskie Archiwum Medycyny Wewnetrznej 1992; 87(1):8-13.

Latt NC, Spencer JD, Beeby PJ, McCaughan GW, Saunders JB, Collins E, Cossart YE. *Hepatitis C in injecting drug-using women during and after pregnancy.* Journal of Gastroenterology & Hepatology 2000; 15(2):175-181.

Lee SD, Chan CY, Wang YJ, Wu JC, Lai KH, Tsai YT, Lo KJ. *Seroepidemiology of hepatitis C virus infection in Taiwan*. Hepatology 1991; 13(5):830-833.

Lestrem, MD, Fainboim H, Mendez N, et al. *HIV-1 Infection in Intravenous Drug Abusers with Clinical Manifestation of Hepatitis in the City of Buenos Aires.* In: AIDS Profile of an Epidemic, PAHO, Scientific Publication. 1989; 514: 51-60.

Li D, Zheng X, Zhang G. *Prevalence of HIV and HCV among injecting drug users (IDUs) in Yunnan, China.* [Chinese]. Chung-Hua Liu Hsing Ping Hsueh Tsa Chih Chinese Journal of Epidemiology 1994; 15(2):74-75.

Libonatti O, Lima E, Peruga A, et al. *Role of Drug Injection in the Spread of HIV in Argentina and Brazil.* International Journal of STD and AIDS. 1993; 4: 135-141.

Lima ES, Azevedo RCS, Manfrinatti MB, et al. *Risk Behaviors for HIV-1 Seroprevalence in a Sample of Injecting Drug Users (IDUS) and Crack Smokers (CSS) in Campinas, Brazil.* XI International Conference on AIDS, Vancouver. 1996; 7/7-14, Abstract Pub.C.1133.

Lindan CP, Lieu TX, Giang LT, et al. *Rising HIV Infection Rates in Ho Chi Minh City Herald Emerging AIDS Epidemic in Vietnam.* AIDS. 1997; 11(supp. 1): S5-S13.

Lohsomboon P, Young NL, Weniger BG, et al. *Nondetection of HTLV-I/II and HIV-2 in Thailand, 1991-1992.* Journal of Acquired Immune Deficiency Syndromes. 1994; 7(9): 992-994.

Lopez-Zetina J, Ford W, Weber M, Barna S, Woerhle T, Kerndt P, Monterroso E. *Predictors of syphilis seroreactivity and prevalence of HIV among street recruited injection drug users in Los Angeles County, 1994-6.* Sexually Transmitted Infections 2000; 76:462-469.

Lorvick J, Kral AH, Seal K, Gee L, Edlin BR. *Prevalence and duration of hepatitis C among injection drug users in San Francisco, Calif.* American Journal of Public Health 2001; 91(1):46-47.

Love A, Stanzeit B. *Hepatitis C virus infection in Iceland: a recently introduced blood-borne disease.* Epidemiology & Infection 1994; 113(3):529-536.

Loxley WM, Marsh AM, Hawks DV, Quigley AJ). *HIV risk behaviour among injecting drug users in Perth: the Australian National AIDS and injecting drug use study.* Med J Aust, 1992, 156: 687-692.

Loxley W, Ovenden C. *Friends and lovers: needle sharing in young people in Western Australia.* AIDS Care 1995; 7(3):337-351.

Lucidarme D, Foutrein P, Creusy C, Forzy G, Foutrein-Comes MC, Muyssen A, Bailly D, Parquet PJ, Filoche B. *Prevalence of hepatitis C, B and D markers and histopathological aspects in a group of intravenous drug addicts.* [French]. Gastroenterologie Clinique et Biologique 1994; 18(11):964-968.

Luksamijarulkul P, Plucktaweesak S. *High hepatitis C seroprevalence in Thai intravenous drug abusers and qualitative risk analysis*. Southeast Asian Journal of Tropical Medicine & Public Health 1996; 27(4):654-658.

Maayan S, Shufman EN, Engelhard D, Shouval D. *Exposure to hepatitis B and C and to HTLV-1 and 2 among Israeli drug abusers in Jerusalem*. Addiction 1994; 89(7):869-874.

MacDonald M, Sullivan P, Locke A, Wodak A, Kaldor J. *HIV and HCV prevalence among trawler crew.* Australian & New Zealand Journal of Public Health 1998; 22(7):829-831.

Majid A, Holmes R, Desselberger U, Simmonds P, McKee TA. *Molecular epidemiology of hepatitis C virus infection amongst intravenous drug users in rural communities.* Journal of Medical Virology 1995; 46(1):48-51.

Mansson AS, Moestrup T, Nordenfelt E, Widell A. *Continued transmission of hepatitis B and C viruses, but no transmission of human immunodeficiency virus among intravenous drug users participating in a syringe/needle exchange program*. Scandinavian Journal of Infectious Diseases 2000; 32(3):253-258.

Marmor M, Des Jarlaid DC, Cohen H, Friedman SR, Beatrice ST, Dubin N, El-Sadr W, Mildvan D, Yancovitz S, Mathur U, Holzman R. AIDS 1987, 1:39-44.

McDonald AM, Whyte BM, Jacobs D, et al. *Voluntary HIV antibody testing among STD clinic patients: A pilot study.* Med J Aust, 1990, 153: 12-14.

McIntyre PG, Hill DA, Appleyard K, Taylor A, Hutchinson S, Goldberg DJ. *Prevalence of antibodies to hepatitis C virus, HIV and human T-cell leukaemia/lymphoma viruses in injecting drug users in Tayside, Scotland*, 1993-7. Epidemiology & Infection 2001; 126(1):97-101.

McKenna JG, Evans G, Lyttle PH, Couper A. *Hepatitis C virus seroprevalence in patients attending a sexual health centre.* New Zealand Medical Journal 1994; 107(970):8-10.

Mesquita F, Moss AR, Reingold AL, et al. *Pilot Study of HIV Antibody Seroprevalence among IVDUs in the City of Santos Sao Paulo State, Brazil*. VII International Conference on AIDS, Florence, Italy, 1991; 6/16-21, Poster M.C.3008.

Metzger DS, Woody GE, McLellan T, O'Brien CP, Druley P, Navaline H, DePhilippis D, Stolley P, Abrutyn E. *Human immunodeficiency virus seroconversion among intravenous drug users in-and out-of-treatment: an 18 month prospective follow up*. Journal of Acquired Immune Deficiency Syndromes 1993; 6:1049-1056.

Ministry of Public Health Thailand. *National Sentinel Seroprevalence Survey*. 1991; (Oct. 28; Feb. 21), 1992 (June), 1993 (June & December), 1994 (June & December), 1996 (June), 1997 (June), 1998 (June), Unpublished tables.

Montoya ID, Atkinson JS. *Determinants of HIV seroprevalence rates among sites participating in a community-based study of drug users.* Journal of Acquired Immune Deficiency Syndromes and Human Retrovirology 1996: 13: 169-176.

Moriarty H, Kemp R, Robinson G. *Hepatitis services at an injecting drug user outreach clinic.* New Zealand Medical Journal 2001; 114(1128):105-106.

Morlet A, Darke S, Guinan J, Wolk J, Gold J). *Intravenous drug users who present to the Albion Street (AIDS) Centre for diagnosis and management of Human Immunodeficiency Virus infection.* Med J Aust. 1990, 152: 78-80.

Moss AR, Vranizan K, Gorter R, Bacchetti, Watters J, Osmond D. *HIV seroconversion in intravenous drug users in San Francisco, 1985 – 1990*. AIDS 1994, 8:223-231.

Muchinik GR, Picchio GR, Bouzas MB, et al. *Seroepidemiology of Human Immunodeficiency Virus in Low and High Risk Groups in Buenos Aires, Argentina.* AIDS-Forschung 1988; 3(2): 89-93.

National Centre in HIV Epidemiology and Clinical Research (NCHECR)). *Annual Surveillance Report 2000:* HIV/AIDS, hepatitis C and sexually transmissible infections in Australia. 2001

Neuwald C, Pont J, Tomasits J, Bauer K. *Antibody prevalence for hepatitis C and other parenterally transmissible viral diseases in i.v. drug dependent patients.* [German]. Acta Medica Austriaca 1992; 19(2):47-48.

Oliveira ML, Bastos FI, Telles PR, Yoshida CF, Schatzmayr HG, Paetzold U, Pauli G, Schreier E. *Prevalence and risk factors for HBV, HCV and HDV infections among injecting drug users from Rio de Janeiro, Brazil*. Brazilian Journal of Medical & Biological Research 1999; 32(9):1107-1114.

Osmond DH, Padian NS, Sheppard HW, Glass S, Shiboski SC, Reingold A. *Risk factors for hepatitis C virus seropositivity in heterosexual couples*. JAMA 1993; 269(3):361-365.

Osztrogonacz H, Gerevich J, Horvath G, Tolvaj G, David K. *Prevalence of chronic viral hepatitis in drug abusers.* [Hungarian]. Orvosi Hetilap 2000; 141(14):715-718.

Pal SC, Sarkar S, Naik TN, et al. *Explosive Epidemic of HIV Infection in North Eastern States of India, Manipur and Nagaland.* CARC Calling. 1990; 3(3): 2-6.

Panda S, Sarkar S, Mandal BK, et al. *Epidemic of Herpes Zoster Following HIV Epidemic in Manipur, India.* Journal of Infection. 1994; 28: 167-173.

Patti AM, Santi AL, Pompa MG, Giustini C, Vescia N, Mastroeni I, Fara GM. *Viral hepatitis and drugs: a continuing problem*. International Journal of Epidemiology 1993; 22(1):135-139.

Payeras CA, Socias MM, Forteza-Rei BJ, Besalduch VJ. *Hepatitis C viral infection and the consumption of intravenous drugs*. [Spanish]. Revista de Sanidad e Higiene Publica 1992; 66(3-4):233-237.

Peak A, Rana S, Maharjan SH, et al. *Declining Risk for HIV among Injecting Drug Users in Kathmandu, Nepal: The Impact of a Harm-Reduction Programme.* AIDS. 1995; 9(9): 1067-1070.

Piribauer F, Duer W. *Trends in HIV seroprevalence, AIDS and prevention policy among intravenous drug users and men who have sex with men, before and after 1990 in Austria*. European Journal of Epidemiology 1998, 14:635-643.

Pont J, Neuwald C, Salzner G. *Antibody prevalence of parenterally transmitted viruses (HIV-1, HTLV-I, HBV, HCV) in Austrian intravenous drug users*. Infection 1991; 19(6):427-430.

Quaranta JF, Delaney SR, Alleman S, Cassuto JP, Dellamonica P, Allain JP. *Prevalence of antibody to hepatitis C virus (HCV) in HIV-1-infected patients (nice SEROCO cohort)*. Journal of Medical Virology 1994; 42(1):29-32.

Ramsay ME, Balogun MA, Collins M, Balraj V. *Laboratory surveillance of hepatitis C virus infection in England and Wales: 1992 to 1996*. Communicable Disease & Public Health 1998; 1(2):89-94.

Rezza G, Sagliocca L, Zaccarelli M, Nespoli M, Siconolfi M, Baldassarre C. *Incidence rate and risk factors for HCV seroconversion among injecting drug users in an area with low HIV seroprevalence.* Scandinavian Journal of Infectious Diseases 1996; 28(1):27-29.

Ribeiro TT, Brites C, Moreira ED, et al. *Serologic Validation of HIV Infection in a Tropical Area.* Journal of Acquired Immune Deficiency Syndromes. 1993; 6(3): 319-322.

Richardson C, Ancelle-Park R, Papaevangelou G for the European Community Study Group on HIV in Injecting Drug Users. *Factors associated with HIV seropositivity in European injecting users*. AIDS 1993, 7:1485-1491.

Robinson GM, Reynolds JN, Robinson BJ. *Hepatitis C prevalence and needle/syringe sharing behaviours in recent onset injecting drug users.* New Zealand Medical Journal 1995; 108(996):103-105.

Rocha M, Lima M, Tanibata P, et al. *Seroprevalence of the Markers the Hepatitis B Virus and Syphilis in Heterosexual Drug Users*. VIII International Conference on AIDS, Amsterdam, 1992 ; 7:19-24, Abstract PoC 4696.

Rodriguez OES, Gil MLM, Santana JFH, Canal JML, Sanchez AMM. *Prevalence of serologic markers of hbv, hdv, hcv and hiv in noninjection drug users compared to injection drug users in Gran Canaria, Spain.* European Journal of Epidemiology 1998; 14(6):555-561.

Rollag H, Thorvaldsen J, Kittelsen P, Bjoro K, Froland S. *Hepatitis C virus infections among persons attending special clinics dealing with problems related to social conditions*. [Norwegian]. Tidsskrift for Den Norske Laegeforening 1993; 113(19):2397-2400.

Sarkar S, Das N, Panda S, et al. *Rapid Spread of HIV among Injecting Drug Users in North-Eastern States of India.* Bulletin on Narcotics. 1993; XLV(1): 91-105.

Sarkar S, Panda S, Sarkar K, et al. *A Cross-Sectional Study on Factors Including HIV Testing and Counselling Determining Unsafe Injecting Practices among*…Indian Journal of Public Health. 1995; 39(3): 86-92.

Saha MK, Chakrabarti S, Panda S, Naik TN, Manna B, Chatterjee A, Detels R, Bhattacharya SK. *Prevalence of HCV & HBV infection amongst HIV seropositive intravenous drug users & their non-injecting wives in Manipur, India*. Indian Journal of Medical Research 2000; 111:37-39.

San Francisco Department of Health. *HIV/AIDS Epidemiology annual report 2000*. HIV/AIDS seroepidemiology and AIDS Surveillance Section.

Schmitt C, Bertel J, Jacob C. *Incidence of serological markers of hepatitis B and C viruses and HIV in a population of drug abusers hospitalized from 1990 to 1992*. [French]. Annales de Medecine Interne 1994; 145(1):7-12.

Selvey LA, Denton M, Plant AJ. *Incidence and prevalence of hepatitis c among clients of a Brisbane methadone clinic - factors influencing hepatitis c serostatus*. Australian & New Zealand Journal of Public Health 1997; 21(1):102-104.

Shah NG, Celentano DD, Vlahov D, Stambolis V, Johnson L, Nelson KE, Strathdee SA. *Correlates of enrollment in methadone maintenance treatment programs differ by HIV-serostatus.* AIDS 2000, 14:2035-2043.

Shirin T, Ahmed T, Iqbal A, Islam M, Islam MN. *Prevalence and risk factors of hepatitis B virus, hepatitis C virus, and human immunodeficiency virus infections among drug addicts in Bangladesh.* Journal of Health, Population & Nutrition 2000; 18(3):145-150.

Shrestha SM, Shrestha S, Tsuda F, Sawada N, Tanaka T, Okamoto H, Miyakawa Y, Mayumi M. *Infection with GB virus C and hepatitis C virus in drug addicts, patients on maintenance hemodialysis, or with chronic liver disease in Nepal*. Journal of Medical Virology 1997; 53(2):157-161.

Singh S, Crofts N. *HIV Infection among Injecting Drug Users in North-East Malaysia*. AIDS Care. 1993; 5(3): 273-281.

Sinniah M, Ooi BG*. Hepatitis C - the Malaysian story*. Singapore Medical Journal 1993; 34(2):132-134.

Smiatacz T, Wlasiuk M, Paszkiewicz J, Zielinska W. *HBV, HCV and HIV infections among drug addicts-residents of rehabilitation and social adjustment centers in the Gdańsk district*. [Polish]. Przeglad Epidemiologiczny 1991; 45(4):351-355.

Smyth BP, Keenan E, O'Connor JJ. *Bloodborne viral infection in Irish injecting drug users*. Addiction 1998; 93(11):1649-1656.

Smyth BP, Keenan E, O'Connor JJ. *Evaluation of the impact of Dublin's expanded harm reduction programme on prevalence of hepatitis C among short-term injecting drug users*. Journal of Epidemiology & Community Health 1999; 53 (7):434-435.

Smyth R, Keenan E, Dorman A, O'Connor J. Hepatitis *C infection among injecting drug users attending the National Drug Treatment Centre*. Irish Journal of Medical Science 1995; 164(4):267-268.

Somaini B, Wang J, Perozo M, Kuhn F, Meili D, Grob P, Flepp M. *A continuing concern: HIV and hepatitis testing and prevalence among drug users in substitution programmes in Zurich, Switzerland*. AIDS Care 2000; 12(4):449-460.

Spencer JD, Latt N, Beeby PJ, Collins E, Saunders JB, McCaughan GW, Cossart YE. *Transmission of hepatitis C virus to infants of human immunodeficiency virus-negative intravenous drug-using mothers: rate of infection and assessment of risk factors for transmission.* Journal of Viral Hepatitis 1997; 4(6):395-409.

Stark K, Schreier E, Muller R, Wirth D, Driesel G, Bienzle U. *Prevalence and determinants of anti-HCV seropositivity and of HCV genotype among intravenous drug users in Berlin*. Scandinavian Journal of Infectious Diseases 1995; 27(4):331-337.

Stark K, Muller R, Bienzle U, Guggenmoos-Holzmann I. *Frontloading: a risk factor for HIV and hepatitis C virus infection among injecting drug users in Berlin.* AIDS 1996; 10(3):311-317.

Stark K, Bienzle U, Vonk R, Guggenmoos-Holzmann I. *History of syringe sharing in prison and risk of hepatitis B virus, hepatitis C virus, and human immunodeficiency virus infection among injecting drug users in Berlin.* International Journal of Epidemiology 1997; 26(6):1359-1366.

Steffen T, Christen S, Blattler R, Gutzwiller F. *Infectious diseases and public health: Risk-taking behavior during participation in the Swiss program for a medical prescription of narcotics (PROVE)*. Substance Use & Misuse 2001; 36(1-2):71-89.

Stein MD, Maksad J, Clarke J. *Hepatitis C disease among injection drug users: knowledge, perceived risk and willingness to receive treatment*. Drug & Alcohol Dependence 2001; 61(3):211-215.

Strathdee SA, Patrick DM, Currie SL, Cornelisse PGA, Rekart ML, Montaner JSG, Schechter MT, Oshaughnessy MV. *Needle exchange is not enough - lessons from the Vancouver injecting drug use study*. AIDS 1997; 11(8):F.

Subhachaturas W, Desjarcais D, Chooranya K, et al. *New Injectors in the HIV Epidemic among Injecting Drug Users in Bangkok, Thailand.* 5th International Congress on AIDS in Asia and the Pacific, Kuala Lumpur, Malaysia. 1999; 10/20-27, Abstract PTCD061.

Suganuma N, Ikeda S, Taketa K, Wang DH, Yamamoto H, Phornphukutkul K, Peerakome S, Sitvacharanum K, Jittiwutikarn J. *Risk analysis of the exposure to GB virus C/hepatitis G virus among populations of intravenous drug users, commercial sex workers and male outpatients at STD clinic in Chiang Mai, Thailand: a cross-sectional case-control study*. Acta Medica Okayama 1998; 52(3):161-167.

Telles PR, Bastos FI, Guydish J, et al. *Risk Behavior and HIV Seroprevalence among Injecting Drug Users in Rio de Janeiro, Brazil.* AIDS. 1997; 11(Suppl. 1): S35-S42.

Thiede H, Hagan H, Murrill CS. *Methadone treatment and HIV and hepatitis B and C risk reduction among injectors in the Seattle area*. Journal of Urban Health 2000; 77(3):331-345.

Thio CL, Nolt KR, Astemborski J, Vlahov D, Nelson KE, Thomas DL. *Screening for hepatitis C virus in human immunodeficiency virus-infected individuals*. Journal of Clinical Microbiology 2000; 38(2):575-577.

Thomas G. *AIDS in India: A Country-wide Threat*. AIDS in India, Rawat Publications, New Delhi. 1994; 151-168.

Thomson JA, Rodger AJ, Thompson SC, Jolley D, Byrne A, Best SJ, Crofts N. *The prevalence of hepatitis C in patients admitted with acute hepatitis to Fairfield Infectious Diseases Hospital, 1971-1975*. Medical Journal of Australia 1998; 169(7):360-363.

Thongcharoen P, Wasi C, Louisirirotchanakul S, et al. *Human Immunodeficiency Virus Infection in Thailand,* Mahidol University, Bangkok. 1989; ISBN 974-586-526-5.

Thorpe LE, Ouellet LJ, Levy JR, Williams IT, Monterroso ER. *Hepatitis C virus infection: prevalence, risk factors, and prevention opportunities among young injection drug users in Chicago, 1997-1999*. Journal of Infectious Diseases 2000; 182(6):1588-1594.

Tor J, Llibre JM, Carbonell M, Muga R, Ribera A, Soriano V, Clotet B, Sabria M, Foz M. *Sexual transmission of hepatitis C virus and its relation with hepatitis B virus and HIV.* BMJ 1990; 301(6761):1130-1133.

Trisler Z, Seme K, Poljak M, Celan-Lucu B, Sakoman S. *Prevalence of hepatitis C and G virus infections among intravenous drug users in Slovenia and Croatia.* Scandinavian Journal of Infectious Diseases 1999; 31(1):33-35.

Trmal J, Kellerova J, Koblizkova R, Krpalkova H, Holasova J. *Effect of needle and syringe exchange programs on the spread of viral hepatitis C*. [Czech]. Epidemiologie, Mikrobiologie, Imunologie 1999; 48(4):171-178.

Trubner K, Polywka S, Puschel K, Laufs R. *Hepatitis C in deceased drug addicts*. International Journal of Legal Medicine 1991; 104(5):251-254.

Ungchusak K, Sriprapandh S, Pinichapongsa S, et al. *First National Sentinel Seroprevalence Survey of HIV-1 Infection in Thailand: June 1989*. Thai AIDS Journal. 1989; 1(2): 57-74.

Unlinked Anonymous HIV surveys steering group. *Prevalence of HIV in the United Kingdom, Data to end 1998. London.* Department of Health, Public Health Laboratory Service, Institute of Child Health (London). Annual report of the unlinked anonymous prevalence monitoring programme. 1998 Prevalence of HIV in the United Kingdom.

Utsumi T, Hashimoto E, Okumura Y, Takayanagi M, Nishikawa H, Kigawa M, Kumakura N, Toyokawa H. *Heterosexual activity as a risk factor for the transmission of hepatitis C virus.* Journal of Medical Virology 1995; 46(2):122-125.

Vanbeek I, Buckley R, Stewart M, MacDonald M, Kaldor J. *Risk factors for hepatitis c virus infection among injecting drug users in Sydney.* Genitourinary Medicine 1994; 70(5):321-324.

Vanbeek I, Dwyer R, Dore GJ, Luo KH, Kaldor JM. *Infection with HIV and hepatitis C virus among injecting drug users in a prevention setting - retrospective cohort study*. BMJ 1998; 317(7156):433-437.

Vanderschueren S, Van Renterghem L, Plum J, Verhofstede C, Mak R, Vincke J. *Hepatitis C among risk groups for HIV and hepatitis B.* International Journal of STD & AIDS 1991; 2(3):185-187.

Vanichseni S, Wright N, Akarasewi P, et al. *Case Control Study of HIV Positivity among Male Intravenous Drug Addicts (IVDA) in Bangkok*. V International Conference on AIDS, Montreal. 1989; 6/4-9, Poster W.G.P. 19.

Vanichseni S, Sakuntanaga P, et al. *Results of Three Seroprevalence Survey for HIV in IVDU in Bangkok.* VI International Conference on AIDS, San Francisco. 1990; 6/20-24, Session F.C.105.

Vanichseni S, Choopanya K, Friedmann P, et al. *Estimated HIV Incidence among Injecting Drug Users in Bangkok Implication for a Vaccine Trial*. 3rd International Conference on AIDS in Asia and the Pacific, Chiang Mai, Thailand. 1995; 9/17-21, Poster PA813.

Vanichseni S, Kitayaporn D, Mastro TD, Mock PA, Raktham S, Des J, Sujarita S, Srisuwanvilai LO, Young NL, Wasi C, Subbarao S, Heyward WL, Esparza L, Choopanya K. *Continued high HIV-1 incidence in a vaccine trial preparatory cohort of injection drug users in Bangkok, Thailand*. AIDS 2001; 15(3):397-405.

Verbaan H, Andersson K, Eriksson S. *Intravenous drug abuse--the major route of hepatitis C virus transmission among alcohol-dependent individuals?* Scandinavian Journal of Gastroenterology 1993; 28(8):714-718.

Villano SA, Vlahov D, Nelson KE, Lyles CM, Cohn S, Thomas DL. *Incidence and risk factors for hepatitis C among injection drug users in Baltimore, Maryland*. Journal of Clinical Microbiology 1997; 35(12):3274-3277.

Vlahov D, Munoz A, Anthony JC, Cohn S, Celentano DD, Nelson KE. *Association of drug injection patterns with antibody to human immunodeficiency virus type 1 among intravenous drug users in Baltimore, Maryland.* American Journal of Epidemiology 1990, 132:5;847-856.

Vlahov D, Anthony JC, Munoz A, Margolick J, Nelson KE, Celentano DD, Solomon L, Polk BF. *The alive study: A longitudinal study of HIV-1 infection in intravenous drug users: description and methods.* Journal of Drug Issues 1991; 21(4), 759-776.

Wada K, Greberman SB, Konuma K, Hirai S. *HIV and HCV infection among drug users in Japan.* Addiction 1999; 94(7):1063-1069.

Waddell RG. *Hepatitis C and the history of injecting drug use in South Australia.* [letter; comment]. Medical Journal of Australia 1994; 161(4):286.

Weinstock HS, Bolan G, Reingold AL, Polish LB. *Hepatitis C virus infection among patients attending a clinic for sexually transmitted diseases.* JAMA 1993; 269(3):392-394.

Weniger BG, Limpakarnjanarat K, Ungchusak K, et al. *The Epidemiology of HIV Infection and AIDS in Thailand.* AIDS. 1991; 5 (suppl 2): S71-S85.

Westh H, Worm AM, Jensen BL, Kroon S, Kvinesdal B, Nielsen CM, Wantzin P. *Hepatitis C virus antibodies in homosexual men and intravenous drug users in Denmark.* Infection 1993; 21(2):115-117.

Widell A, Hansson BG, Berntorp E, Moestrup T, Johansson HP, Hansson H, Nordenfelt E. *Antibody to a hepatitis C virus related protein among patients at high risk for hepatitis B*. Scandinavian Journal of Infectious Diseases 1991; 23(1):19-24.

Wiebel WW, Jimenez A, Johnson W, Ouellet L, Jovanovic B, Lampinen T, Murray J, O'Brien MU. *Risk behaviour and HIV seroincidence among out-of-treatment injection drug users: a four-year prospective study*. Journal of acquired immune deficiency syndromes and Human Retrovirology 1996, 12:282-289.

Will T. *Early decline in an epidemic: evolution of the prevalences of HIV, hepatitis B, hepatitis C and aminotransferases in intravenous drug abusers in Strasbourg between 1980 and 1990.* [French]. Revue Medicale de la Suisse Romande 1999; 119(4):329-334.

Woodfield DG, Harness M, Rix-Trott K, Tsuda F, Okamoto H, Mayumi M. *Identification and genotyping of hepatitis C virus in injectable and oral drug users in New Zealand.* Australian & New Zealand Journal of Medicine 1994; 24(1):47-50.

Wolk J, Wodak A, Morlet A, Guinan JJ, Gold J). *HIV-related risk-taking behaviour, knowledge and serostatus of intravenous drug users in Sydney*. Med J Aust, 1990, 152: 453-458.

World Health Organisation Programme on Substance Abuse Collaborative Study Group (WHO PSA)). *Multi-City Study on Drug Injecting and Risk of Infection.* WHO/PSA. 1994, 94.4, Geneva.

Worm AM, Gottschau. *No change in incidence and prevalence of HIV among intravenous drug users in Copenhagen from 1985 to 1990*. Journal of acquired immune deficiency Syndromes, 1993; 6:845-848.

Wormser GP, Forseter G, Joline C, Tupper B, O'Brien TA. *Hepatitis C in HIV-infected intravenous drug users and homosexual men in suburban New York City.* JAMA 1991; 265(22):2958.

Wright NH, Vanichseni S, Akarasewi P, et al. *Was the 1988 HIV Epidemic among Bangkok's Injecting Drug Users a Common Source Outbreak?* AIDS. 1994; 8(4): 529-532.

Xinhua S, Junhua N, Qili G. *The Growing Problem of AIDS in Asia.* VI International Conference on AIDS, San Francisco, 1990; 6/24, Closing Ceremony, vol. 3, p. 93.

Ye S. *Prevalence of antibody to hepatitis C virus in 177 drug addicts*. [Chinese]. Chung-Hua Liu Hsing Ping Hsueh Tsa Chih Chinese Journal of Epidemiology 1993; 14(1):45-48.

Zeldis JB, Jain S, Kuramoto IK, Richards C, Sazama K, Samuels S, Holland PV, Flynn N. *Seroepidemiology of viral infections among intravenous drug users in northern California.* Western Journal of Medicine 1992; 156(1):30-35.

OTHER REFERENCES

ABS (Australia Bureau of Statistics)(1995). Deaths, Australia 1994. ABS Catalogue No. 3302.0. AGPS, Canberra, 1995.

Alcabes P, Munoz A, Vlahov D and Freidlan GH (1993). *Incubation period of human immunodeficiency virus*. Epidemiol Rev, 15: 303-318

Becker NG, Watson LF and Carlin JB (1991). *A method of non-parametric back-projection and its application to AIDS data. Stat Med,* 10: 1527-1542

Bennett WG, Inoue Y, Beck JR, et al (1997). *Estimates of the cost-effectiveness of a single course of interferon-alpha2B in patients with histologically mild chronic hepatitis C*. Ann Intern Med, 127: 855-865.

Bonkovsky HL, Woolley JM and the Consensus Interferon Study Group (1999). *Reduction of health-related quality of life in chronic hepatitis C and improvement with interferon therapy*. Hepatology, 29: 264-270.

Bruneau J, Lamothe F, Franco E, et al (1997). *High rates of HIV infection among injecting drug users participating in needle exchange programs in Montreal: results of a cohort study*. Am J Epidemiol, 145: 994-1002.

Des Jarlais DC, Hagan H, Friedman S, et al (1995). *Maintaining low HIV seroprevalence in populations of injecting drug users.* JAMA, 274: 1226-1231.

Drucker E, Lurie P, Wodak A and Alcabes P (1998). *Measuring harm reduction: the effects of needle and syringe exchange programs and methadone maintenance on the ecology of HIV*. AIDS, 12 (Suppl A): S217-S230.

Drummond et al (1997). *Methods for the Economic Evaluation of Health Care Programmes*; 2nd edition, Oxford University Press

English D, Holman CDJ, Milne E, et al (1995). *The quantification of drug caused morbidity and mortality in Australia, 1995 edition*. Commonwealth Department of Human Services and Health, Canberra, 1995.

Fattovich G, Giustina G, Degos F, et al (1997). *Morbidity and mortality in compensated cirrhosis type C: a retrospective follow-up study of 384 patients.* Gastroenterol, 112: 463-472.

Freeman A, Dore GJ, Law MG, Thorpe M, Von Overbeck J, Lloyd A, Marinos G and Kaldor JM (2001). *Estimating progression to cirrhosis in chronic hepatitis C.* Hepatology, 34: 809-816.

Grulich AE, Wan X, Law MG, Coates M and Kaldor JM (1999). *Risk of cancer in people with AIDS*. AIDS, 13: 839-843.

Guydish J, Bucardo J, Young M, Woods W, Grinstead O and Clark W (1993). *Evaluating needle exchange: Are there negative effects?* AIDS, 7: 871-876.

Hagan H, McGough JP, Thiede H, et al (1999). *Syringe exchange and risk of infection with hepatitis B and C viruses.* Am J Epidemiol, 149: 203-213.

Hall WD, Ross JE, Lynskey MT, Law MG and Degenhardt LJ (2000). *How many dependent heroin users are there in Australia?* Med J Aust, 173: 528-531.

Holtgrave DR and Pinkerton SD (1997). *Updates of cost of illness and quality of life estimates for use in economic evaluations of HIV prevention programs*. JAIDS, 16: 54-62.

Hurley SF, Jolley DJ and Kaldor JM (1997). *Effectiveness of needle-exchange programmes for prevention of HIV infection*. Lancet, 349: 1797-1800.

Hurley SF, Kaldor JM, Carlin JB, et al (1995). *The usage and costs of health services for HIV infection in Australia*. AIDS, 9: 777-785.

Law MG on behalf of the Hepatitis C Virus Projections Working Group (1999). *Modelling the hepatitis C virus epidemic in Australia.* J Gastro Hepatol, 14:1100-1107.

MacDonald M, Crofts N and Kaldor J (1996). *Transmission of hepatitis C: Rates, routes and cofactors*. Epidemiol Rev, 18: 137-148.

Marschner IC and Watson LF (1992). *An improved EMS algorithm for back-projection of AIDS incidence data.* Research Report 92/1, Department of Statistics, La Trobe University, Melbourne.

NCHECR (2001). *HIV/AIDS, viral hepatitis and sexually transmissible infections in Australia Annual Surveillance Report 2001*. National Centre in HIV Epidemiology and Clinical Research (Ed), Sydney, 2001.

Rodger AJ, Jolley D, Thompson SC, Lanigan A and Crofts N (1999). *The impact of diagnosis of hepatitis C virus on quality of life*. Hepatology, 30: 1299-1301.

Rosenberg PS, Gail MH and Carroll RJ (1992). *Estimating HIV prevalence and projecting AIDS incidence in the United States: a model that accounts for therapy and changes in the surveillance definition of AIDS.* Stat Med, 11: 1633-1655.

Schoenbaum EE, Hartel DM and Gourevitch MN (1996). *Needle exchange use among a cohort of injecting drug users*. AIDS, 10: 1729-1734.

Shiell A, Briggs A and Farrell G (1994). *The cost effectiveness of alpha interferon in the treatment of chronic active hepatitis C*. Med J Aust, 160: 268-272.

Tengs, Tammy O, Wallace, Amy, *One Thousand Health-Related Quality-of-Life Estimates*, Medical Care, 2000; Vol 38 No. 6: 583-637

Thorley A (1981). *Longitudinal studies of drug dependence. In: Drug problems in Britain: a review of ten years*. Eds: Edwards G, Busch C. Academic Press.

US Census Bureau & UNAIDS (2000). *HIV/AIDS Surveillance Data Base.* Health Studies Branch, International Programs Center Population Division, US Census Bureau, Washington.

van Ameijden EJC, van den Hoek JAR and Coutinho RA (1994). *Injecting risk behaviour among drug users in Amsterdam 1986 to 1992, and its relationship to AIDS prevention programs.* Am J Pub Health, 84: 275.

Watters JK, Estilo MJ, Clark GL and Lorvick J (1994). *Syringe and needle exchange as HIV/AIDS prevention for injecting drug users*. Journal of American Medical Association, 271: 115-120.

Wolk J, Wodak A, Guinan JJ, Macaskill P and Simpson JM (1990). *The effect of a needle and syringe exchange on a methadone maintenance unit. British Journal of Addiction*, 85. 1445-1450.
